# Supplementary material for: Omicron COVID-19 immune correlates analysis of a third dose of mRNA-1273 in the COVE trial
Source: Nat Commun. 2024 Sep 11;15:7954. doi: 10.1038/s41467-024-52348-9 (PMC11390939; doi:10.1038/s41467-024-52348-9)
Supplement: Supplementary file 1 — Supplementary Information [file 41467_2024_52348_MOESM1_ESM.pdf]

Supplementary Information for

Omicron COVID-19 Immune Correlates Analysis of a Third Dose of mRNA-1273 in the COVE Trial

Bo Zhang, Youyi Fong, Jonathan Fintzi, Eric Chu, Holly E. Janes, Avi Kenny, Marco Carone, David Benkeser, Lars van der Laan, Weiping Deng, Honghong Zhou, Xiaowei Wang, Yiwen Lu, Chenchen Yu, Bhavesh Borate, Haiyan Chen, Isabel Reeder, Lindsay N. Carpp, Christopher R. Houchens, Karen Martins, Lakshmi Jayashankar, Chuong Huynh, Carl J. Fichtenbaum, Spyros Kalams, Cynthia L. Gay, Michele P. Andrasik, James G. Kublin, Lawrence Corey, Kathleen M. Neuzil, Frances Priddy, Rituparna Das, Bethany Girard, Hana M. El Sahly, Lindsey R. Baden, Ruben O. Donis, Richard A. Koup, Peter B. Gilbert, Dean Follmann*, on behalf of the United States Government (USG) COVID-19 Immune Assays Team; Moderna, Inc. Team; Coronavirus Vaccine Prevention Network (CoVPN)/Coronavirus Efficacy (COVE) Team; and USG/CoVPN Biostatistics Team
Correspondence to: dfollmann@niaid.nih.gov

Contents:

Supplementary Tables 1-15
Supplementary Figures 1-37
Supplementary References
Statistical Analysis Plan

United States Government (USG) COVID-19 Immune Assays Team

(PubMed listed, and ordered alphabetically by affiliation)

Affiliation	Team Members
Biomedical Advanced Research and Development Authority (BARDA), Washington, DC	Oleg Borisov, Flora Castellino, Brett Chromy, Mark Delvecchio, Ruben O. Donis, Tremel Faison, Corey Hoffman, Christopher Houchens, Tom Hu, Chuong Huynh, Pennie Hylton, Lakshmi Jayashankar, Aparna Kolhekar, James Little, Karen Martins, Jeanne Novak, Carol Sabourin, Evan Sturtevant, Xiaomi Tong, John Treanor, Danielle Turley, Leah Watson
Boston Consulting Group, Boston, MA	Gian King, Andrew Li, Najaf Shah, Smruthi Suryaprakash, Jue Xiang Wang
Division of AIDS, NIAID, NIH, Bethesda, MD	Patricia D'Souza
Division of MID (Microbiology and Infectious Diseases), NIAID, NIH, Bethesda, MD	Janie Russell
Duke University, Durham, NC	David Beaumont, Kendall Bradley, Jiayu Chen, Xiaoju Daniell, Thomas Denny, Elizabeth Domin, Amanda Eaton, Kelsey Engel, Wenhong Feng, Juanfei Gao, Hongmei Gao, Kelli Greene, Sarah Hiles, Leihua Liu, Kristy Long, Kellen Lund, Charlene McDanal, David C. Montefiori, Marcella Sarzotti-Kelsoe, Francesca Suman, Haili Tang, Jin Tong, Olivia Widman
The Tauri Group, an LMI company - Contract Support for U.S. Department of Defense (DOD) Joint Program Executive Office for Chemical, Biological, Radiological and Nuclear Defense (JPEO-CBRND) Joint Project Manager for Chemical, Biological, Radiological, and Nuclear Medical (JPM CBRN Medical), Fort Detrick, MD, USA	Christopher S. Badorrek, Gregory E. Rutkowski
Vaccine Research Center, NIAID, NIH, Bethesda, MD	Akua Abrah, Obrimpong Amoa-Awua, Manjula Basappa, Robin Carroll, Erykah Coe, Jevone Fentress, Britta Flach, Suprabhath Gajjala, Nazaire Jean-Baptiste, Richard A. Koup, Bob C. Lin, Adrian McDermott, Christopher Moore, Mursal Naisan, Muhammed Naqvi, Sandeep Narpala, Sarah O'Connell, Abhinaya Srikanth, Clare Whittaker, Weiwei Wu

Moderna, Inc. Team

(PubMed listed, ordered alphabetically)

Affiliation	Team Members
Moderna, Inc., Cambridge, MA	Weiping Deng, Shu Hahn, Jacqueline Miller, Rolando Pajon, Honghong Zhou

Coronavirus Vaccine Prevention Network (CoVPN)/Coronavirus Efficacy (COVE) Team

(PubMed listed, and ordered alphabetically by institution affiliation)

Affiliation/Funding*	Study Group	Location
AB Clinical Trials	Atoya Adams, MD, MBA, Eric Miller	Las Vegas, NV
Accel Research Sites	Bruce G. Rankin DO, John Hill MD, Steven Shinn MD, Marshall Nash MD	DeLand, FL
Advanced Clinical Research	Sinikka L. Green MD, Colleen Jacobsen, Jayasree Krishnankutty, Sikhongi Phungwayo	Cedar Park, TX
Alliance for Multispecialty Research	Richard M. Glover, II MD, Drs. Stacy Slechta, Troy Holdeman, Robyn Hartvickson, Amber Grant	Newton, KS
Alliance for Multispecialty Research	Terry L. Poling MD, Terry D. Klein MD, Thomas C. Klein MD, Tracy R. Klein MD	Wichita, KS
Alliance for Multispecialty Research	William B. Smith MD, Richard L. Gibson MD, Jennifer Winbigler MD, Elizabeth Parker PA	Knoxville,TN
Baptist Health Center for Clinical Research	Priyantha N. Wijewardane, MD, Eric Bravo MD, Jeffrey Thessing MD, Michelle Maxwell APRN, Amanda Horn APRN	Little Rock, AR
Baylor College of Medicine, NIAID 1UM1AI148575-01S2	Hana El Sahly MD, Jennifer Whitaker MD, Catherine Mary Healy MD, Christine Akamine MD	Houston, TX
Benchmark Research	Laurence Chu, MD, R. Michelle Chouteau, MD	Austin, TX
Benchmark Research	Michael J. Cotugno MD, George H. Bauer, Jr. MD	Metairie, LA
Benchmark Research	Greg Hachigian MD, Masaru Oshita MD, Michael Cancilla NP, Deborah Murray NP, Kristen Kiersey NP	Sacramento, CA
Benchmark Research	William Seger MD, Mohammed Antwi, Allison Green, Anthony Kim	Fort Worth, TX
Brigham and Women's Hospital, NIAID UM1AI069412, NCATS UL1RR025758	Lindsey R Baden MD, Michael Desjardins MD, Jennifer A Johnson MD, Amy Sherman MD, Stephen R Walsh MD	Boston, MA
Carolina Institute for Clinical Research	Judith Borger DO, Ryan Starr DO, Scott Syndergaard DO, Nafisa Saleem MD	Fayetteville, NC
Centex Studies	Joel Solis MD, Martha Carmen Medina PA-C, Westly Keating PA-C, Edgar Garcia PA-C, Cynthia Bueno PA-C	McAllen, TX
Clinical Research Atlanta	Nathan Segall MD, Nathan Segall, Jon Finley, Mildred Stull	Stockbridge, GA
Clinical Trials of Texas	Douglas Scott Denham DO, Thomas Weiss MD, Ayoade Avworo DNP, Parke Hedges MD	San Antonio, TX
Coastal Carolina Research Center	Cynthia Becher Strout MD, Rica Santiago, Yvonne Davis, Patty Howenstine, Alison Bondell	Mount Pleasant, SC
Cornell Clinical Trials Unit - Weill Cornell Uptown & Weill Cornell Chelsea, NIAID UM1AI068619, NCAT UL1TR002384	Kristin Marks MS MD, Grant Ellsworth, MS, MD, Tina Wang, MD, Timothy Wilkin, MD, MPH, Mary Vogler, MD, Carrie Johnston, MD, MS	New York, NY
Covid19 Prevention Network (CoVPN, NIAID-NIH)	Michele P Andrasik, Jessica G Andriesen, Gail Broder, Lawrence Corey, Niles Eaton, Kathleen M Neuzil, Huub G Gelderblom, James G Kublin, Rachael McClennen, Nelson Michael, Merlin Robb, Carrie Sopher	Seattle, WA
DM Clinical Research	Vicki E. Miller MD, MPH, Fredric Santiago MD, Blanca Gomez FNP-C, Insiya Valika PA-C, Amy Starr FNP-C	Tomball, TX
Emory University – Ponce de Leon Clinical Research Site, NIAID 3UM1AI068614-14S1	Colleen Kelley MD MPH, Valeria D Cantos MD, Sheetal Kandiah MD MPH, Carlos del Rio MD	Atlanta, GA
Emory University – Hope Clinic, NIAID 1UM1AI148576-01	Nadine Rouphael MD, Paulina Rebolledo, Srilatha Edupuganti, Daniel Sans Graciaa	Decatur, GA
Emory University School of Medicine, NIAID 1UM1AI148576-01	Evan J Anderson MD, Andres Camacho-Gonzalez MD, Satoshi Kamidani MD, Christiana A Rostad MD, Meghan Teherani MD	Atlanta, GA
George Washington University, NIAID UM1AI068619	David Joseph Diemert MD, Elissa Malkin, Marc Siegel, Afsoon Roberts, Gary Simon	Washington, DC
Hackensack University Medical Center	Bindu Balani MD, Carolene Stephenson, Steven Sperber, Cristina Cicogna	Hackensack, NJ
Henry Ford Health System	Marcus J. Zervos MD, Paul Kilgore MD, MPH, Mayur Ramesh MD, Erica Herc MD, Kate Zenlea MPH	Detroit, MI
Hope Research Institute	Abram Burgher MD, Ann Marie Milliken	Phoenix, AZ
Hope Research Institute	Joseph D. Davis MD, Brendan Levy, Sandra Kelman	Chandler, AZ
Hope Research Institute	Matthew W. Doust MD, Denise Sample, Sandra Erickson	Phoenix, AZ
J. Lewis Research	Shane Glade Christensen MD, Christopher Matich, James Longe, John Witbeck	Salt Lake City, UT
J. Lewis Research	James Todd Peterson MD, Alexander Clark, Gerald Kelty, Issac Pena-Renteria	Salt Lake City, UT

Affiliation/Funding*	Study Group	Location
Jacksonville Center for Clinical Research	Michael J. Koren MD, Darlene Bartilucci MD, Jeffery Jacqmein MD, Alpa Patel MD, Carolyn Tran MD	Jacksonville, FL
Javara	Christina Kennelly MD, Robert Brownlee, Jacob Coleman, Hala Webster	Charlotte, NC
Johnson County Clin-Trials	Carlos A. Fierro MD, Natalia Leistner, Amy Thompson, Celia Gonzalez	Lenexa, KS
Kaiser Permanente Washington Health Research Institute, NIAID 1UM1AI148373-01	Lisa A Jackson MD MPH, Janice Suyehira MD	Seattle, WA
Laguna Clinical Research Associates	Milton Haber MD, Maria M. Regalado MD, Veronica Procasky RN JD, Alisha Lutat	Laredo, TX
Lynn Health Science Institute	Carl P. Griffin MD, Raymond Cornelison, William Schnitz, Shanda Gower	Oklahoma City, OK
Lynn Institute of the Rockies	Ripley R. Hollister MD, Jeremy Brown DO, Melody Ronk PA-C	Colorado Springs, CO
M3 Wake Research	Wayne Lee Harper MD, Lisa Cohen DO, Lynn Eckert PA-C, Matthew Hong MD	Raleigh, NC
MediSync Clinical Research Hattiesburg Clinic	Rambod Rouhbakhsh MD, MBA, Elizabeth Danford MD, John Johnson MD, Richard Calderone MD	Petal, MS
Meridian Clinical Research	Shishir Kumar Khetan MD, Oyebisi Olanrewaju AC-CRNP, Nan Zhai NP-C, Kimberly Nieves AC-CRNP, Allison O'Brien AC-CRNP	Rockville, MD
Meridian Clinical Research	Paul Simon Bradley MD, Amanda Lilienthal MSN NP-C, Jim Callis PA-C	Savannah, GA
Meridian Clinical Research	Adam Benson Brosz MD, Andrea Clement PA, Whitney West APRN, Luke Friesen PA, Paul Cramer APRN	Grand Island, NE
Meridian Clinical Research	Frank Steven Eder MD, Ryan Little FNP, Victoria Engler FNP, John Tarbox FNP, Heather Rattenbury-Shaw DO	Binghamton, NY
Meridian Clinical Research	David Jon Ensz MD, Tavane Harrison, Allie Oplinger	Dakota Dunes, SD
Meridian Clinical Research	Brandon James Essink MD, Jay Meyer MD, Frederick Raiser, III MD, Kimberly Mueller APRN, Roni Gray PA	Omaha, NE
Meridian Clinical Research	Keith William Vrbicky MD, Charles Harper MD, Chelsie Nutsch MD, Wendell Lewis III MD, Cathy Laflan MD	Norfolk, NE
Meridian Clinical Research	Jordan L. Whatley MD, Nicole Harrell MD, Amie Shannon MD, Crystal Rowell APRN, FNP-C, Christopher Dedon APRN, FNP-C	Baton Rouge, LA
NIH	Mamodikoe Makhene MD MPH	Bethesda, MD
New Horizons Clinical Research	Gregory Mark Gottschlich MD, Kate Harden PA-C, Melissa Gottschlich PA-C, Mary Smith MSN, FNP-C, Richard Powell MD	Cincinnati, OH
Optimal Research	Murray A. Kimmel DO, Simmy Pinto MD	Melbourne, FL
Optimal Research	Timothy P. Vachris MD, Mark Hutchens MD, Stephen Daniels DO, Margaret Wells MD	Austin, TX
Optimal Research	Mimi Van Der Leden MD, PhD, Peta Gay Jackson Booth MD	Rockville, MD
Palm Beach Research Center	Mira Baron MD, Pamela Kane DO, Shannen Seversen PA-C, Mara Kryvicky PA-C, Julia Lord PA-C	West Palm Beach, FL
Paradigm Clinical Research Center	Jamshid Saleh MD, Matthew Miles, Rafael Lupercio	Redding, CA
Quality of Life Medical & Research Centers	John W. McGettigan Jr. MD, Walter Patton MD, Riemke Brakema MD, Karin Choquette MSN, ABNP-C, Jonlyn McGettigan MSN, RN	Tucson, AZ
Rancho Paseo Medical Group	Judith L. Kirstein MD, Marcia Bernard NP	Banning, CA
Rapid Medical Research	Mary Beth Manning MD, Joan Rothenberg MD, Toby Briskin MD, Denise Roadman PAC, Sharita Tedder-Edwards FNP	Cleveland, OH
Research Centers of America	Howard I. Schwartz MD, Surisday Mederos, Barbara Corral, Jennifer Schwartz, Nelia Sanchez-Crespo	Hollywood, FL
Rutgers New Jersey Medical School, NIAID UM1AI068619	Shobha Swaminathan MD, Amesika Nyaku MD MS, Tilly Varughese MD, Michelle DallaPiazza MD	Newark, NJ
Saint Louis University, NIAID 1UM1AI148685-01	Sharon E Frey MD, Irene Graham MD, Getahun Abate MD PhD MSc, Daniel Hoft MD PhD	St. Louis, MO
St. Vincent's Health System	Leland N. Allen III MD, Leslie Anne Edwards MSN, CRNP, William Simpson Davis Jr., MS PA-C, Jessica Maria Mena, PA	Birmingham, AL
Suncoast Research Group	Mark E. Kutner MD, Jorge Caso MD, CPI, Maria Hernandez Moran APRN, Marianela Carvajal APRN, Janet Mendez APRN	Miami, FL
Sundance Clinical Research	Larkin T. Wadsworth III MD, Horacio Marafioti, Lyly Dang, Jennifer Berry, Lauren Clement	St. Louis, MO
Synexus Clinical Research	Michael Ryan Adams MD, Leslie Iverson PA	Murray, UT
Synexus Clinical Research	Joseph Lee Newberg MD, Laura Pearlman MS, MD, MBA	Chicago, IL
Synexus Clinical Research	Paul Joseph Nugent DO, Leonard Singer	Cincinnati, OH

Affiliation/Funding*	Study Group	Location
Synexus Clinical Research	Michele Diane Reynolds MD, Jennifer Bashour MD, Robert Schmidt MD	Dallas, TX
Synexus Clinical Research	Neil Parmanand Sheth MD, Kenneth Steil DO	Glendale, AZ
Synexus Clinical Research	Ramy Joseph Toma MD, William Kirby MD, Pink Folmar MD, Samantha Williams NP	Birmingham, AL
Synexus Clinical Research	Judith White MD, Robert Meyer MD, Sejal Patel MD, Prity Patel APRN	Orlando, FL
Tekton Research	Paul Pickrell MD, Stefanie Mott FNP-C, Carol Ann Linebarger MD, Hussain Malbari MD,David Pampe MD	Austin, TX
Texas Center for Drug Development	Veronica G. Fragoso MD, Lisa Holloway MD, Cecilia McKeown-Bragas MD, Teresa BeckerMD, Vicki Miller MD	Houston, TX
Trial Management Associates	Barton G. Williams MD, William H. Jones MD	Wilmington, NC
VA Greater Los Angeles Healthcare System	Michael Lewis MD, Elham Ghadishah, Joseph Yusin, Mai Pham	Los Angeles, CA
University of California Los Angeles, NIAID UM1AI068619	Jesse L Clark MD, Steven Shoptaw PhD, Michele Vertucci PA, NP, Will Hernandez NP	Los Angeles, CA
University of California San Diego, NIAID UM1AI068636	Stephen A. Spector MD, Amaran Moodley MD, Jill Blumenthal MD, Lisa Stangl NP, KarenDeutsch NP	La Jolla, CA
University of Chicago	Kathleen M. Mullane DO PharmD, David Pitrak MD, Cheryl Nuss FNP, Judy Pi PharmD	Chicago, IL
University of Cincinnati, NIAID UN1AI068619	Carl Fichtenbaum MD, Margaret Powers-Fletcher PhD, Michelle Saemann RN, SharonKohrs RN	Cincinnati, OH
University of Colorado Denver, Anschutz Medical Campus, NIAID UM1AI068636	Thomas B. Campbell MD, Andrew Lauria, Jose Castillo Mancilla, Hillary Dunlevy	Aurora, CO
University of Illinois at Chicago – Project WISH, NIAID UM1AI068619	Richard M Novak MD, Andrea Wendrow, Scott Borgetti, Ben Ladner	Chicago, IL
University of Maryland School of Medicine, NIAID 1UM1AI148689-01	Karen L Kotloff MD, Matthew Laurens, Milagritos Tapia, Lisa Chrisley, Cheryl Young	Baltimore, MD
University of Miami, NIAID 3UM1AI068614-14S1	Susanne Doblecki-Lewis MD, Maria Luisa Alcaide, Jose Gonzales-Zamora, Stephen Morris	Miami, FL
University of North Carolina at Chapel Hill, NIAID UM1AI068619	Cynthia Gay MD MPH, Erin Hoffman, Susan Pedersen, Maria Bullis, Mandy Tipton, Carolina Pastrana Medina, Catherine Kronk, Nicole Maponga, Julie Nelson, Becky Straub, Amy James Loftis, David Wohl MD, Joseph Eron, Jr. MD	Chapel Hill, NC
University of Pennsylvania, NIAID 3UM1AI068614-14S1	Ian Frank MD, Debora Dunbar, David Metzger, Florence Momplaisir	Philadelphia, PA
University of Pittsburgh Medical Center, NIAID 1UM1AI148452-01	Judith Martin MD, Alejandro Hoberman MD, Timothy Shope MD MPH, Gysella Muniz MD	Pittsburgh, PA
University of Texas Medical Branch, NIAID 1UM1AI148575-01	Richard Rupp MD, Amber Stanford PA-C, Megan Berman MD, Laura Porterfield MD	Galveston, TX
VA Greater Los Angeles Healthcare System	Michael Lewis MD, Elham Ghadishah, Joseph Yusin, Mai Pham	Los Angeles, CA
Vanderbilt University Medical Center, NIAID 1UM1AI148452-01	Clarence Buddy Creech II MD, Shannon Walker MD, Stephanie Rolsma MD PhD, RobertSamuels, Isaac Thomsen MD	Nashville, TN
Vanderbilt University Medical Center, NIAID 3UM1AI068614-14S1	Spyros Andrews Kalams MD, Greg Wilson MD	Nashville, TN
Velocity Clinical Research	Gregg H. Lucksinger MD, Kevin Parks MD, Ryan Israelsen MD, Jaleh Ostovar FNP-C, KaryKelly FNP-C	Medford, OR
Velocity Clinical Research, San Diego	Jeffrey Scott Overcash MD, Hanh Chu, Kia Lee, Karla Zepeda	La Mesa, CA
VitaLink Research	Luis I. De La Cruz MD, Steve Clemons, Elizabeth Everette, Suzanna Studdard	Greenville, SC
VitaLink Research	Gowdhami Mohan MD, Stefanie Tyson, Alyssa-Kay Peay, Danyel Johnson	Anderson, SC
VitaLink Research-Spartanburg	Gregory J. Feldman MD, May-Yin Suen, Jacqueline Muenzner, Joseph Boscia, FarhanSiddiqui	Spartanburg, SC
Wake Forest University Health Sciences	John Sanders MD,PhD, James Peacock MD, Julio Nasim MD	Winston Salem, NC
WR-Clinical Research Center of Nevada	Michael L. Levin MD, Julie Hussey MSN APRN FNP-C, Marcy Kulic MD	Las Vegas, NV
WR-ClinSearch	Mark Montgomery McKenzie MD, Teresa Deese, Erica Osmundsen, Christy Sweet	Chattanooga, TN
WR-Global Medical Research	Valentine Mbepson Ebuh MD MA MSc, Elwaleed Elnagar MD, Georgette Ebuh DNP APRNFNP-C, Genevieve Iwuala FNP	Dallas, TX
WR-Medical Center for Clinical Research	Laurie J. Han-Conrad MD, Todd Simmons MD, Denis Tarakjian MD	San Diego, CA

*Funding of institutions by the National Institute of Allergy and Infectious Diseases (NIAID) and/or research support by the National Center for Advancing Translational Science (NCATS) as indicated. All other institutions were funded by Office of the Assistant Secretary for Preparedness and Response, Biomedical Advanced Research and Development Authority. The content of this publication is solely the responsibility of the authors and does not necessarily represent the official views of the funding sources.

CoVPN/COVE Team (cont'd): COVE Trial Investigators and Study Teams

Principal Investigator	Study Team	Institution	Location
Atoya Adams, MD, MBA	Miriah Campbell, Eric Miller, Daisy Langarica, Alia Bober, Diana Giraldo	AB Clinical Trials	Las Vegas, NV
Michael Ryan Adams, MD	Leslie Iverson, Andryelle Toledo, Melinda Bullington, Alicia Hanten, Carolyn Taylor, Shannon Wright, Chase Carnahan, Rachel Law, Natalie Smith, Julie Taylor, Jared-Robert Blake, Stefanie Vasconez, Courtney Jensen	Synexus Clinical Research	Murray, UT
Leland N. Allen III, MD	Leslie Anne Edwards, William Simpson Davis, Jr., Ronald Meza, Jordan Stauffer, John Farringer, Faith Holmes, Rhonda Buzbee, Cristina Velez, Huse Lisa, Lisa Huse, Camelia Speegle, Gregory Prestage, Mary Perez, Jessica Space, Matthew Todd, Jessica McDowell, Marha Bunnell-Pollak, Jackie Ziegler, Jasmine Ali, Dumitru Sirbu, Kellie Williams, Logan Sawyer, Richelle Chambliss, Samantha Blackmon, Stephanie Brennan, Tiffany Gibbs, Alexandria Anderson, Caitlin Roll, Candace Robinson, Zachary McCoy, Jessica Bartlett, Kimberly Cornelison, Chris Bovell, Vincent Baglini, Christy Greenhalgh, Jessica Maria Mena, David House, Matt Honold, Esteban Zurita	St. Vincent's Health System	Birmingham, AL
Evan J. Anderson, MD	Kathleen Stephens, Francine Dyer, Maya Stagg, Aaliyah Carron, Austin Lu, Julia Barton, Sy Tran, Leisa Bower, Esther Park, Jianguo Xu, Rebecca Gonzalez, Vy Ngo, Mike Shepard, Lezly Roxxette Zepeda, Karen Sytsma, Sandra Rojas-Honan, Felicia Glover, Susan Rogers, Theda Gibson, Christina A. Rostad, Andres Camacho-Gonzalez, Teresa Ball, Satoshi Kamidani, Mehgan Farah Teherani, Vikash Patel, Etza Peters, Peggy Kettle, Lisa Macoy, Cindy Lubbers, Amber Samuel, Laila Hussaini, Kathryn Zaks, Caroline Ciric, Meg Taylor, Oliver Smith, Amy Muchinsky, Sydney Biccum, Laura Clegg, Dean Kleinhenz, Angelle Ijeoma, Hannah Huston	Emory University School of Medicine	Atlanta, GA
Lindsey Baden, MD	Xhoi Mitre, Jon Gothing, Bruce Bausk, Jessica Cauley, Natalie Izaguirre, Lewis Novack, Michael Seaman, Katherine Yanosick, Henry Rutherford, Junghyun Kim, Dominique Betterbed, Kathleen Garvey, Lauren Clore, Alexander Mills, Deepesh Duwadi, Alessandra Setaro, Kyl Bowman, Kevin McManus, Sidali Beriane, Fadi Ghantous, Christy Lavine, Jasper Ophel, Joseph Sapiente, Jessica Dorning, Tessa Speidel, Lauren Garneau, Robert Dannemiller, Kirquenique Rolle, Mulika Chhorn, Bailey McCarthy, Hana Flaxman, Milenko Tanasijevic, Cameron Nutt, Javier Barria, Andre Avila-Paz, Buteau Malhaika, Tong Alexandra, Tenaizus Woods, Bethany Evans, Hannah Jin, LaKeisha Gandy, Stephanie St. Pierre, Carolyn Darcy, Michael Corrado, James Maguire, Adetoun Okenla, Tamara Roldon Sevilla, David Kubiak, Cassandre Titus, Movita Harrigan, Maria Alvarado, Rose Theodat, Amy Sherman, Laura Platt, Kirsten Goodman, Laura Nicholson, Wilfredo Matias, Emily Koleske, Ruth Rodriguez, Nicole Taikeff, Jun Bai Park Chang, Julia Klopfer, Phoebe Cunningham, Elizabeth Sampson, Karen Magsipoc, Maureen Macgowan, Lauren Donahue, Haley Schram, Noah Abasciano, Megan Powell, Janet Morgan, Yazed Alsowaida, Olivia Riccardi, Neha Limaye, Virginia Loudermilk, Austin Kim, Kevin Zinchuk, Caitlin Grant, Charles Kelly, David Mellace, Jamie Myers, Erika Gribb, Jose Licona, Monica Feeley, Stephen R Walsh, Jennifer A Johnson, Ann Woolley, Alexis Liakos, Jane Kleinjan, Jon Gothing, Nicolas Issa, Michael Desjardins, Raphael Dolin, Alka Patel, Opeyemi Talabi, Christin Price, Paulette Chandler, Elizabeth W Karlson, Allison P Moriarty	Brigham and Women's Hospital	Boston, MA
Bindu Balani, MD	Smith Kerowyn, Sergio Garcia, Charo Valdez, Shelly Chin, Caitlin DiBello, Silvia Lara, Chika Ekweghariri, Abena Roberts, Abimbola Coker, Marie-Therese Estanbouli, Greg Eskinazi, Michael Tortoriello, Jay Elkareh, Meral Karakoc, Olga Spathis, Patrice Hassoun, Carolene Stephenson, Steven Sperber, Kaur Harveen, Cristina Cicogna, Ciaran Mannion	Hackensack University Medical Center	Hackensack, NJ
Mira Baron, MD	Pamela Kane, Maria Bermudez, Shannen Seversen, Mara Kryvicky, Julia Lord, Terri Barr, Daisy Acevedo, Elena Acosta, Delta Anderson, Alexandra Arango, Anne Bauer, Joshua Egbehor, Tim Flanary, Audrey Haber, Carol Henao, Patti Isaacson, Peter Jacob, Sakaiya Jackson, Karen Kodes, Ludovic La-Branche, Kimarie Lee-Russell, Carol Liso, Cristina Liso, Stephanie Morse, Michelle Navarrette, Christy Norcross, Nora Norcross, Annette Pitts, Mary Sergalis, David Scott, Tytiana Spearman, Danielle Theodore, Brian Thomas, Jennifer Torres	Palm Beach Research Center	West Palm Beach, FL
Judith Borger, DO	Jennifer Angell, Nicole Austin, Deanna Benz, Lucian Cappoli, Nicole Davis, Lynn Eckert, Kathryn Hostetter, Stephanie Keating, Jeanette Mangual-Coughlin, Avia McClain-Stocker, Ifeanyi Momodu, Cheryl Norris, Brennan Opanasenko, Stacey Saldua, Nafisa Saleem, Amy Sheets, Ryan Starr, Scott Syndergaard, Jennifer Thomas, Michelle Wallace, Jeffery Pemberton, Mitchell Arildsen, Dan Tomita	Carolina Institute for Clinical Research	Fayetteville, NC
Paul Simon Bradley, MD	Taja Adams, Stephanie Ailey, Kira Bell, Shanice Bennett, Vincent Bernades, Jim Callis, Bounphone Chanthavong. Taryn Collett, Anne Crouch, Shannon Davis, Morgan Deal, Mimi Duncan, Brandon Essink, Laura Falcone, Debra Gabrielson, Brooke Halpern, Anyfa Hanna, Cassie Heisey, Dawn Kalloniatis, Andrew Kimball, Jeanette Lee, Amanda Lilienthal, Ginny McNew, Crystal Neely, Kay Lynn Olmsted, Nicole Osborn, Chevon Roberts, Pechoka Sanders, Cynthia Seedorf, Kathryn Stoddard, Jonathan Whelan, Stella Yoon	Meridian Clinical Research	Savannah, GA

Principal Investigator	Study Team	Institution	Location
Adam Benson Brosz, MD	Rhonda Richter, Debra Gabrielson, Kayla Flege, Ashley Bell, Karen Jo Johnson, Paul Cramer, Jessica Stanton, Andrea Clement, Whitney West, Laura Falcone, Amanda Friesz, Kathy Osborne, Summer Tophoj, Kimber Breeden, Susan Newman, Douglas Herbek, Lindsey Mettenbrink, Luke Friesen, Alison Pierce	Meridian Clinical Research	Grand Island, NE
Abram Burgher, MD	Stephanie Catanzaro, Shauna Harrell, Magen Hess, Nate Alderson, Bettie D'Nise Corcoran, Norma Frederick, Adrian Alejo, Brian DeCraene, Karen Wakefield, Scarlett Hammett, Susan DeCraene, Ann Marie Milliken, Neil Pearson, Donald Terral Harper	Hope Research Institute	Phoenix, AZ
Thomas B. Campbell, MD	Andrew Lauria, Jenelynn Kimble, Steven Johnson, Matin Krsak, Andrew Monte, Patrisha Adkins, Michelle Barron, Suzanne Fiorillo, Amy Harrison, Anderson Victoria, Nga Le, Sara Berech, Jose Castillo-Mancilla, Kristine Erlandson, Laurel Ware, Josie Marshall, Stephen Bartlett, Hillary Dunlevy	University of Colorado Denver, Anschutz Medical Campus	Aurora, CO
Shane Glade Christensen, MD	Christopher Mickelson, Jessica Shaw, Emily Raming, Amy Nelson, Gabrielle Lewis, Jenessa Folsom, Mikaela Jones, Dylan Owen, Rachel Pugmire, Jennifer Bradley, Annjanette Kemp, Krista Marti, Allyson Christensen, Madison Ellis, Holly Anderson, Emily Bloomquist, Ross Brunetti, Thomas Conner. Jr., Gina Cox, Diana Grazulis, Wesley Lewis, James Longe, Christopher Matich, Bryan Nelson, Sarah Scott, John Witbeck, Stephen Wood	J. Lewis Research	Salt Lake City, UT
Laurence Chu, MD	Jennifer Bacchi, Maria Barrientes, Lamar Box, Christian Casas, R. Michelle Chouteau, Katherine Davis, Tambra Dora, Cindy Duran, Pamela Fidler, Ruth Fitch, Brooke Harris, Isaiah Knight, Jennifer Leyva, Michelle Listz, Jennifer Montes, Javier Perez, Jessica Ruff, Dean Skiles, Sean Turnbow, Francesca Vigil, Breana Wade, Kelly Weber	Benchmark Research	Austin, TX
Jesse L. Clark, MD	Sandy MacNicoll, Somaieh Talebi, Timothy Hall, Steven Shoptaw, Emery Chang, Michael Li, David Goodman, Paul Adamson, Oladunni Adeyiga, Inez Bentancourt, Susan Reed, Christopher Blades, Jasmin Tavares, Demetria Villanueva, Simone Riley, Jonathan Veloz, Schuyler Thomas, Will Hernandez, Jennifer Baughman, Mitchell Stern, Michele Vertucci	University of California, Los Angeles	Los Angeles, CA
Michael J. Cotugno, MD	Kyra Lawson, Kim Harper, Edwin Adamson, George H. Bauer Jr., Julie Bilich, Brenda Lawson, Brandon Illickal, Lois Eaglin, Heather Salisbury, Jeff Segner	Benchmark Research	Metairie, LA
Clarence Buddy Creech II, MD	Shanda Phillips, Naomi Kown, Katherine Sokolow, Wendy Winn, Katherine Wright, Shannon Walker, Stephanie Rolsma, Anna Gallion, April Hanlotxomphou, Deborah Myers, Robert Adkisson, Natalia Jimenez, Cindy Trimmer, Roberta Winfrey, Matthew Donio, John Oleis, Donna Torr, Shelly McGehee, Robert Samuels, Sandra Yoder, Eric Brady, Isaac Thomsen, Madeleine Guy, Emma Alexander, Lana Howard, Krisha Alexander, Shane Moore, Tacora Wright, Tara Evans, Ursula Powell, Jenna Caserta, Valerie Mitchell, Meryk Moore, Melissa Lehman, Diane Anders, Constance Dotye, Crystal Rice, Lamar Bowman, Sherri Hails, Monique Bennett, Nicki Soper, Leigh Howard	Vanderbilt University Medical Center	Nashville, TN
Joseph D. Davis, MD	Sandra Kelman, Sandra Braden, Sabrina Bolland, Mia Munoz, Jose Barocio, Brendan Levy, Dhwani Shah, Neil Pearson, Stephanie Catanzaro, Nathan Alderson, Susan DeCraene, Maureen Godfrey, Skyla Clark	Hope Research Institute	Chandler, AZ
Luis I. De La Cruz, MD	Amy Ford, Taylor Wilson, Cindy Smith, Austin Lambert, Erin Zeiler, Kaelyn Rowland, Marlee Smith, Suzanna Studdard, Zandra Hamilton, Meredith Benfield, Sara Poff, David Godwin, Elizabeth Everette, Steven Clemons, Kayla Peay, Stephanie Gilreath	VitaLink Research	Greenville, SC
Douglas Scott Denham, DO	Thomas Weiss, Parke Hedges, Ayoade Avworo, Kay Scroggins, Leisel Koerber, Antonio Gutierrez, Nathan Cortez, Andrea Gomez, Darlington Akahara, Michelle Smith, Kristy Trevino, Beatriz Herrera, Shaiane Dickerson, Kerry de Jesus, Matthew Korte, Cynthia Ramos, Reanna Martinez, Erica Leal, Shakera Flores, Paul Esparza, Brian Hemming, Melinda Axton, D'Andre White, Terri Perez, Carolina Coronado, Rebecca Many, Clayton Stone, Kimberly Evans, Anshumaan Maharaj, Stephen Brick, Steffanie Barrera, Staci Poettgen, Dawn Killian, Gerardo Pena, Karol Perez, Victoria Hernandez, Kevin Martinez, Amy Griffith, Nolan Payton, Quincey Hogue, Jamie Padilla, Emily Mendez, Lily Hays, Maristelle Co, Nicholas Trinidad, Ismael Rodriguez, Amy Lewis, Cindi Nellis, Lele Simmons, Marissa Johnson	Clinical Trials of Texas	San Antonio, TX
David Joseph Diemert, MD	Linda Witkin, Aimee Desrosiers, DeEnna Wedding, Bertran Walton, LaKeisha Queen, Ryan Mouton, Caroline Thoreson, Manya Magnus, Jennifer Wald, Erika Faust, Nicholas Heredia, Robbie Kattappuram, Hira Qadir, Chelsea Ware, Hannah Yellin, Kegan Dasher, Daniel Mullen, Jeanne Jordan, Taylor Ladson, Madison Lintner, Kaitlyn Macnair, Bitana Saintilma, Kelly Thomas, Samantha Walker, Neha Rampally, Madhu Balachandran, Elissa Malkin, David Parenti, Hana Akselrod, Marc Siegel, Gary Simon, Afsoon Roberts, Aileen Chang	George Washington University	Washington, DC

Principal Investigator	Study Team	Institution	Location
Susanne Doblecki-Lewis, MD	Maria Luisa Alcaide, Jose Gonzalez-Zamora, Stephen Morris, Yimy Puerto, Annie Salvarrey, Claudia Balgas, Claudia Santos, Katherine King, Brahian Steven Erazo, Mayra Fernandez, Leopoldo Cordova-Garcia, Elisa Corzo-Sanchez, Edgar Fernandez, Loreta Padron, Stefani Ann Butts, Kenia Moreno, Juan Casuso, Maria de Pilar Valanzasca, Thomas Tanner, Marilyn Fernandez, Mary Aloise, Inza Patton, Vivian Pastrana, Sendy Puerto, Irma Barreto Ojeda, Junlin Long, Barbara Huang, Gilianne Narcisse, Vanessa Perez	University of Miami	Miami, FL
Matthew W. Doust, MD	Denise Sample, Sandra Erickson, Nate Alderson, Adrian Alejo, Stephanie Catanzaro, Susan DeCraene, Cassie Enricco, Sandra Erickson, Alex Guereque, Shauna Harrell, Shana Harshell, Stephanie Junker, Stephanie Laufenberg, Madison Mikulak, Makayla Morra, Nicole Olson, Neil Pearson, Jasmin Redden, Monique Romo, Denise Sample, Dhwani Shah, Sahara Vega, Emma Kar	Hope Research Institute	Phoenix, AZ
Valentine Mbepson Ebuh, MD	Elwaleed Elnagar, Georgette Ebuh, Genevieve Iwuala, Catina Adams, Marissa Cervenka, Ezgar Del Real, Shraddha Dubal, Elwaleed Elnagar, Jenifer Fiatte, Kathy Harrell, Genevieve Iwuala, Vicki Martinez, Robert Miranda, Brennan Opanasenko, Destiny Robinson, Liz Ruiz, Amy Sheets, Shoniece Wallace	WR-Global Medical Research	Dallas, TX
Frank Steven Eder, MD	Ryan Little, Victoria Engler, John Tarbox, Heather Rattenbury-Shaw, Deborah Hubish, Jessie Taylor, Debra Gabrielson, Jessica Fellows, Jennifer Molstead, Kathe Olmstead, Ashley Conover, Tammy Kohn, Chelsea Briar, Corrine Young, Collen McVannan, Kelli Quick, Shaylynne Hubanks, Kimber Breeden, Ann Marie Sampson, Traci Hull, Tarin Gordon, Susan Owen, Kate Macarak, Tonya Rackett, Jacob Blattstein, Partidge Jane Aton, Nicole Croft, Carolyn Grausgruber, Rebecca Miller, Ryan Little, Victoria Engler, John Tarbox, Heather Rattenbury-Shaw, Nathan Kimball, Courtney Heisey, Ginny McNew, Abigail Wine, Cindi VanKuren, Jared Frick, Tammy Dennis, Andrew Kimball	Meridian Clinical Research	Binghamton, NY
Hana M. El Sahly, MD	Jennifer A. Whitaker, C. Mary Healy, Christine Akamine, Wendy A Keitel, Robert L Atmar, Annette Nagel, Sandra Francisco, Thea Marie Cordero, Janet Brown, Jennifer Christensen, Caroline Doughty-Skierski, Connie Rangel, Carrie Kibler, Coni Cheesman, Lisreina Toro, Chanei Henry, Chianti Wade Bowers, Pedro Piedra, Kathy Bosworth, Kayla Burrell, Jesus Banay, Tykel Eddy, Trent Davis, Shetel Anassi, Yvette Rugeley, Olga Rybina-Willis	Baylor College of Medicine	Houston, TX
David Jon Ensz, MD	Pamela Allen, Taylor Bergh, Kimber Breeden, Avery Dunn, Brandon Essink, Debra Gabrielson, Rylea Gulick, Tavane Harrison, Courtney Heisey, Andrew Kimball, Shelby Klaschen, Jessica Knight, Makayla Langston, Meagan Miller, Allie Oplinger, Heather Persinger, Alison Pierce, Kathryn Stoddard, Kayla Sturgeon, Jamie Thompson, Melissa Wiseman	Meridian Clinical Research	Dakota Dunes, SD
Brandon James Essink, MD	Jay Meyer, Frederick Raiser, Kimberly Mueller, Roni Gray, Riley Brockman, Tabitha Campbell, Carrie Essink, Laura Falcone, Roni Gray, Linda Layton, Jay Meyer, Kimberly Mueller, Tiffany Nemecek, Frederick "Fritz" Raiser, III, Jessica Satorie, Chelsea Steinmetz, Nicole Osborn, Cassie Heisey, Maria Nguyen	Meridian Clinical Research	Omaha, NE
Gregory J. Feldman, MD	May-Yin Suen, Brittany Cooksey, Madison Fowler, Sarah Chynoweth, Gary Clemons, Laura Jolly, Charlie Jordan, Heather Allison, Steve Clemons, Amber Brittany Belcher, Allison Kelly, Marsha Gossett, Wendy Taylor, Amy Witt, Kendal Nelson, Jeffrey Witt, Jacqueline Muenzner, Elizabeth Everette, Supinder Channa, Allison Ayers, Joseph Boscia, Farhan Siddiqui	VitaLink Research-Spartanburg	Spartanburg, SC
Carl J. Fichtenbaum, MD	Maggie Powers-Fletcher, Michelle Saemann, Sharon Kohrs, Kimberly Mullins, Lindsay Davis, Moises Huaman, Angela Snyder, Kristin Weghorn, Brenda Miller, Elizabeth Costea, Lisa Schira, Romana Saeed, Helen Shelton, Kathleen Ballman, Laura Browning-Cho, Sherry Donaworth, Chris Goddard, Jeanine Goodin, Elizabeth Niederegger, Lisa Hachey, Tamara Maus, Pam Fletcher, Makayla Bishop, Victoria Straughn, Shaina Horner, Carrie Christofield, Dana Burns, Jason Mayes, Kelly Windholtz, Lisa Proffitt, Faizan Qureshi, Michelle O'Neil, Arustamyan Lisa, Sarah Trentman, Eva Whitehead, Jennifer Baer, Linda Hinds, Jaasiel Chapman, D'Vaughn House, Gary Frazier, Judy Houston, Lisa Altenau, Mary Burns, Dorice Smith, Justin Ragle, Eric Mueller, Cynthia Nypaver, Jaime Robertson, Anissa Moussa, Geronimo Feria Garzon, Sierra Bennett, Marlena Petrie	University of Cincinnati	Cincinnati, OH
Carlos A. Fierro, MD	Mazen Zari, Celia Gonzalez, Natalia Leistner, Mary Easley, Mary Provost, Krista Estrada, Ann Geier, Amy Thompson, Heather Barker, Karol Moore, Kelly Moen, Monica Atwood, Amber Wolf, Brandi Dickerson, Manyvohn Rinehart, Dina Hammine, Angela Eichler, Casey Johnson, Nathan Arthur	Johnson County Clin-Trials	Lenexa, KS
Veronica G. Fragoso, MD	Lisa Holloway, Cecilia McKeown-Bragas, Teresa Becker, Vicki Miller, Leena Mir, Elton Oliveira, Moez Talpur, Enya Rentas-Sherman, Gabriela Maria Becerra, Dewayne Hicks, Robert Krbashyan, Shakira Barr, Ashraf Jafri, Herman Ortiz, Zohair Harianawala, Chandra Tobin, Norma Gonzalez, Saji Perinjelil, Khorshid Amirkhosravi, Tracy Kowalski, Biman Goswami, Waheeda Sureshbabu, Amy Anderson, Berenice Ferrero, Simeen Khan, Chen-Ho Yang, Nazanin Zarinkamar, Scott Ward, Crystal Reese, Miyosha Lewis, Olga Konshina, Lorrian Yates.Joel Cano, Quiana Wilson, Kara	Texas Center for Drug Development, Inc.	Houston, TX

Principal Investigator	Study Team	Institution	Location
	Sikes, Diana Chehab, Joanna Quezan, Maryam Rabbani, Sadaf Batla, Abbyssinia Moges. Diego Carrington, Matthew Joseph, Laura Grissanty, Dean Jang, Dustin McFadden, Misbah Baloch, Elisa Moralez, Abdeali Dalal, Frances Saubon, William Fernandez, Jenny Toress, Blessing Felix, Zain Rizvi		
Ian Frank, MD	Annet Davis, Eileen Donaghy, Nicole Sundo, Juan Ramirez, Laura Schankel, Dana Brown, Katharine Bar, Dana Brown, Christopher Chianese, Gillain Constantino, Dovie Watson, Kathleen Degnan, Helen Koenig, William Short, Petra Alexander, Eileen Mergliano, Jie Ho, Michele Wisniewski, Debora Dunbar, Liani Santini-Lopez, Rosemarie Kappes, Angela Cabassa, Tammy Chen, Berry SotoVega, Deborah Kim, Devon Cliett, Kate Kearns, Jillian Baron, Vivian Leung, Florence Momplaisir, Sarah Wood, Tameka Matthews, David Metzger, Richard Tustin	University of Pennsylvania	Philadelphia, PA
Sharon E. Frey, MD	Irene Graham, Getahun Abate, Daniel Hoft, Heather Douds, Cassandra Zehenny, Joan Siegner, Helay Hassas, Kim Cooper, Shirley Dettlebach, Sabrina DiPiazza, Carol Duane, Linda Eggemeyer-Sharpe, Lauren Foreman, Jerry Hutter, Ryan Kerr, Kate Liefer, Tracy Montauk, Karla Mosby, Janice Tennant, Nicole Purcell, Kiana Wilder, Kathleen Chirco, Sharon Irby-Moore, Kathleen Koehler, Melissa Loyet, Thomas Pacatte, Susan Stewart, Azra Blazevic, Tamara Blevins, Chase Colbert, Christopher Eickhoff, Lainey Mejia Jauregui, Keith Meyer, Krystal Meza, Amanda Nethington, Huan Ning, Brittany Williams, Mei Xia, Yinyi Yu, Stanley Doublin, Mary Pat Eastman, Eric Eggemeyer, Mikayla Frye, Michelle Harris, Aleshia McCoy, Donna Duncan, Gwendolyn Tatum, Nicole Purcell, Kiana Wilder, Tammy Grant, Claudia Castillo Paredes, Rong Hou, Jin Wang, Qian Wang, Sarah George	Saint Louis University	St. Louis, MO
Cynthia Gay, MD	David Wohl, Joseph Eron, Jr., , Janette Goins, Ulrike Adam, Ekundayo Nylander-Thompson, Anna Furlong, XinHong Ao, Kathy Guerrero, Melinda Hart, Kathleen Loeven, Rachael Mossey, Esther Speight, Rachel White, Chloe Twomey, Kristen Gray, , Patti Vasquez, April Welch, Camille O'Reilly, Maureen Furlong, Noshima Darden-Tabb, Elizabeth DuBose, Marie Oriol, Dynesha Perry, Maria Stetson, Maria Bullis, Shelby Turner, Ebony Harrington, Alexander Bradley, Susan Pedersen, Becky Straub,Sandra Barnhart, Tevnan Keller, MandyTipton, Abigail Riddick, Kristi Kirkland, Maggie Harman, Tania Hossain, Centhla Washington, Erin Hoffman, Carolina Pastrana Medina, William Johnson, Samantha Earnhardt, Amy James Loftis, Catherine Kronk, Yaa Ofori-Marfoh, Julie A Nelson, Nicole Maponga, Lina Rosengren-Hovee, William Zhao, Jennifer Thompson, Sarah Law, Holly Milner, Jonathan Oakes, Rachel Cook, Erin Cardot, Oesa Vinesett, Victoria Rucinski, Joy Wannamaker, Tanailly Giralt Smith, Eliza DuBose, Chidinma Okafor	University of North Carolina at Chapel Hill	Chapel Hill, NC
Richard M. Glover, II, MD	Stacy Slechta, Troy Holdeman, Robyn Hartvickson, Amber Grant, Jennifer Bennett, Lindsey Brewer, Janelle Brown, Kelsey Burden, Melissa Burton, Brianna Burton, Jordan Danby, Sheri Duncan, Amber Grant, Robyn Hartvickson, Lisa Hemmelgarn, Sherry Henning, Jeri King, Riley King, Colton King, April Kitterman, Shannen Lassiter, Cayla Lawless, Janna Martinez, Ragene Moore, Marissa Mueller, Aaron Nguyen, Justin Phillips, Jordan Reheis, Rebecca Ring, Katherine Saengerhausen, Shannon Thomas, Dylan Thomas, Cindy Thome, Denae Villines, Amber Wenzel, Eilleen Wilbert, Avi Woods, Caressa Presley, Brianna Newport, Olivia Allen, Miranda Santiago, Cheryl Sauerwein, Jill Longstaff, Sadie Allen, Candace Heckart	Alliance for Multispecialty Research	Newton, KS
Gregory Mark Gottschlich, MD	Melissa Gottschlich, Steven Anderson, Gregory Mark Gottschlich II, Mary Woeste, Kate Harden, Cindy Young, Michael Pordy, Audrius Ruksenas, Lacy Baird, Kim Krogman, Lori Stanton, Melissa Fuson, Mason Urban, Christine Watson, Richard Powell, Mary Smith, Jacob Sekinger, Diamond Russell, Nicole Lim, Mylene Asmar-Rios, Yusef Museitif, Craig Mitchell, Tarik Whitham, Zachary Rutledge, Troy Porter, Andrea Newlands, Jami Ramsey, Mary Frances Curry, Nishay Holloman, Crystal Barket, Michelle Spear, Shelley Mahan, Taeleigha Greene, Zachary Eardley, Gen Moussa, Mary Ann Gottschlich	New Horizons Clinical Research	Cincinnati, OH
Sinikka L. Green, MD	Julie Hamilton, Alex Fuller, Jeanette Dickhaus, Colleen Jacobson, Triny Cooper, Michelle Jackson, Taylor Evans, Tabitha Judd, Kathryn Alexander, Megan Rosallo, Sikhongi Phungwayo, Robin Dotson, Dana Finley, Michael Vasquez, Cyndi Foster, Gregg Lucksinger, Sarah Smiley, Jayasree Krishnankutty, Ray Coon, Grishma Dhimmer, Melanie Wilkerson, Tatum Shawver, Marcedes Coffman, Devin Teal, Laura Crenshaw	Advanced Clinical Research	Cedar Park, TX
Carl P. Griffin, MD	William Schnitz, Andrea Romero, Kim Hamilton, Raymond Cornelison, Angela Genovese, Shelly Brunson, April Green, Lacey Dietz, Kim Calloway, Chris Hyatt, Destiny Heinzig-Cartwright, Chalimar Rojo, Sharee Wright, Kathi Shaw, Michael Pojezny, Avery Keller, Krystal Hightower, Dalia Tovar, Shanda Gower	Lynn Health Science Institute	Oklahoma City, OK
Milton Haber, MD	Maria Candelario, Martha Bunnell-Pollak, Lauren Wade, Jackie Ziegler, Deena Ramirez, Perla Avalos, Maria Drada, Jasmine Ali, Jessica McDowell, Kehinde Busari, Patricia Church, Ronald Meza, Marco Vela, Esteban Zurita, Chris Connolly, Ruben	Laguna Clinical Research Associates	Laredo, TX

Principal Investigator	Study Team	Institution	Location
	Del Bosque, Alisha Lutat, Chelsea Fleming, Brett Potthoff, Anita Suri, Cynthia Priester, Brenda Hernandez, Veronica Procasky, Eva Cerreta, Matt Honold, Melinda Rodriguez, Maria Regalado, Jordan Stauffer		
Greg Hachigian, MD	Michael Cancilla, Ricardo Castellanos, Angela Cuellar, Yaman Darmarathne, Shaila Faulker, Yana Gordeyeva, Michelle Hisey, Ashley Jungsten, Kristin Kiersey, Pawandeep Nagra, Nav Nagra-Kooner, Jazmin Nauta, Masaru Oshita, Kenneth Quick, Julie Raygoza, Amanny Sadek, Melisa Tinder, Jhoana Torres, Deborah Murray, Kristen Kiersey	Benchmark Research	Sacramento, CA
Laurie J. Han-Conrad, MD	Brandon Baldwin, Lucian Cappoli, Tenisha Garcia, Ella Grach, Brenda Grande, Nicolle Mendez, Natalie Moy, Matthew Musikanth, Karen Mylerberg, Brennan Opanasenko, Mark Pulera, Patti Sanchez-Emery, Mireles Sarah, Todd Simmons, Denis Tarakjian	WR-Medical Center for Clinical Research	San Diego, CA
Wayne Lee Harper, MD	Toni Bland, Lori Bridges, Lucian Cappoli, Lisa Cohen, Leah Corts, Annie Craft, James Earnhardt, Lynn Eckert, Aubrey Farray, Laura Hoer, Matthew Hong, Chris Hoyle, Jenee Jiggetts, Brian Joseph, Bradley Killebrew, Kendra Lisec, Lucie Mangala, David Musante, Adnan Nasir, Amanda Olsen, Brennan Opanasenko, Marci Parks, Marion Peoples, Katherine Schuch, Judith Shand, Sabine Ucik, Douglas Wadeson, Barbara Wheeler	M3 Wake Research	Raleigh, NC
Ripley R. Hollister, MD	Jeremy Brown, Brandy Ball, Jeremy Brown, Valerie Dyster, Dalia Jeronimo, Shelby Pickle, Michael Pojezny, Melody Ronk, Kathi Shaw, Bobbi Shofner, Jami Wagner, Meghan York, Jill York	Lynn Institute of the Rockies	Colorado Springs, CO
Lisa A. Jackson, MD, MPH	Marilyn Nguyen, Maya Dunstan, Barbara Carste, Sarah Friend, Diana McFeters, Lynn Gross, Mohamed Ajenah, Jana Ffitch, Audra Mccoy, David Skatula, Susan Lasicka, Kimberly Brinker, Karen Sherwin, Melissa Scheer, Paula Lins, Roger Calvert, Roxanne Erolin, Stella Lee, Vi Tran, Stephanie Pimienta, Bruce Douglas, Lee Barr, Colin Fields, Erika Kiniry, Joe Choe, Janice Suyehira, Joyce Benoit, Michael Witte, Rebecca Lau	Kaiser Permanente Washington Health Research Institute	Seattle, WA
Spyros Andrews Kalams, MD	Greg Wilson, Kyle Rybczyk, Katie Crumbo, Carly Griffin, Latoya Hannah, Amy Kerrigan, Valerie Mitchell, Jenna Caserta, Mary Downey, Nicole Swindle, Shonda Sumner, Amber Massey, Trudy Sullivan, Rita Smith, Cindy Nochowicz, Eric Olson, Christian Warren, Josh Simmons, Dana King, Gwendolyn Rees, Matt Donio, Jesse Case, Keith Richardson, Jarissa Greenard	Vanderbilt University Medical Center	Nashville, TN
Colleen Kelley, MD, MPH	Valeria D. Cantos, Sheetal Kandiah, Carlos del Rio, Christina Bacher, Hannah Huston, Juliet Brown, Divya Bhamidipati, Nithin Gopalsamy, Brittany Lynn Speigel, Elizabeth (Betsy) Hall, Brandon Spratt, Kiran Dhillon, Caitlin Moran, Michael Chung, Felecia Wright, Marcia Peters, Rondell Jaggers, Vanessa Soliman, Ron Gaston, Christopher Foster, Sarah Wiatrek, Bezuayehu Mandefro, Pamela Weizel, Pamela Lankford-Turner, Anandi Sheth, John Gharbin, Catherine Abrams, Philip Powers, Paulina Rebolledo, Christin Root, Tiraje Lester, Sha Yi, Damien Swearing, Fred Ede, Isaac Perez, Kelly Likos, Meen Dhir, Aastha KC, Gabriela Georgial, Tucker Colvin, Nabeel Yar Khan, Valarie Hunter, D'Jamel Young, Felecia Atkinson	Emory University<br>Emory University – Ponce de Leon Clinical Research Site	Atlanta, GA
Christina Kennelly, MD	Jacob Coleman, Brittany Bundeff, Melissa C. Hennessey, Kenneth Owen, Caroline Wilds Wilds, Jennifer Womack, Susan Martello, Chiedza Hooker, Robert Brownlee, Melissa James, Deborah Wesley-Farrington, Lori Whiteheart, Hala Webster, David Framm, Cortney Fretz, Gwyn Gibson, Susan Donahue, Kelly Woodell, Linda McCarty, Jim Vesely, Scott Chatterton, Andrew Ottesen, Enrico Belgrave, Krishna Shah, James Chester Alexander, Brittain Callahan	Javara	Charlotte, NC
Shishir Kumar Khetan, MD	Taja Adams, Tanya Alexander, Tanya Alexaner, Sydney Barmoy, Jake Bart, Kira Bell, Ira Berger, Jemario Blackwell, Priscilla Buahin, Bounphone Chanthavong, Juliana DeVito, Azure Erskine, Brandon Essink, Laura Falcone, Debra Gabrielson, Beau Garland, Barb Geiger, Tiana Oliver, Courtney Heisey, Sucharita Katikala, Andrew Kimball, Heather Lang, Jeanette Lee, Asefa Mekonnen, Devan Myers, Kimberly Nieves, Allison O'Brien, Oyebisi Olanrewaju, Nicole Osborn, Adunola Oshiyoye, Rahul Patel, Alan Pollack, April Poole, Collin Smith, Kathryn Stoddard, Chao Wang, Sean Whelan, Jonathan Whelan, Graciela Zapata, Nan Zhai	Meridian Clinical Researc	Rockville, MD
Murray A. Kimmel, DO	Alexa Diec, Ann Riley, Bette Denmat, Bram Swarr, Christina Raidl, Dania Billman, Denise Dixon, Donald Dawson, Elaine Crudo, James Crowley, Katrina Carlson, Kaylie Worzick, Laura Worth, Lisbeth Gordon, Marion Oliver, Robert Holt, Simmy Pinto, Taylor Atkinson, Traci Mitchell, Lana Ghomrawi, Norma Rokoff	Optimal Research	Melbourne, FL
Judith L. Kirstein, MD	Jared Bradshaw, Krista Forster, Jeanette Dickhaus, Marcia Bernard, Erica Sanchez, Nikki Abels, Cynthia Kunakom, Vanessa Vandergoot, Jessica Fisher, Carol Remigio, Jourdan Manfred, Frederick Lloyd, Tiffany Williams, Clarisse Baudelaire, Lovette Cherelle, Nolan Mackey, Alan Valenzuela, Theodore Wyman, Alyssa Taber, Karen Myers, Craig Koch	Rancho Paseo Medical Group	Banning, CA
Michael J. Koren, MD	Shannon Trull, Amanda Elwood, Mary Strickland, Ivy Gulliermo, Chistopher Ganzhorn, Sonia Gerardo, Taylor Johnson, Victoria Kaposchansky, Cassie Lawler, Laura Little, Amanda Pratt, Sheldon Warren, Andrea West, Emery Noles, Nathanial	Jacksonville Center for Clinical Research	Jacksonville, FL

Principal Investigator	Study Team	Institution	Location
	Grant, Jillian Agnew, Lori Alexander, Brenda Anderson, Deirdre Arrington, Sara Benner, Lisa Carl, Allison Crain, Nafisa Ishaku, Robert Nix, Sharon Smith, Amber Devries, Sandy Salceiro, Opara Chukwudi, Mikaela Karney-Trull, Ramil Castillo, David Graham, Gail Lowe, Alexander Hill, Carolyn Tran, Jeffry Jacqmein, Darlene Bartilucci, Alpa Patel, Janet Garvey, Mitchell Rothstein, Kenneth Aung-Din, Margaret Gannaway, Arman Mughal, Sandra Fuit, Jolenne Wolfer, Erin Schelhorn, Jacob Wolfer, Madison Martinez, Melissa Parks, Patricia Neal		
Karen L. Kotloff, MD	Matthew Laurens, Milagritos Tapia, Lisa Chrisley, Cheryl Young, Barbara Albert, Robin Barnes, Shernel Barrett, Andrea Berry, Melissa Billington, Shannon Bittner, Colleen Boyce, Faith Pa'Ahana Brown, James Campbell, Regina Carpenter, Jamonie Carter, Ginny Cummings, Brenda Dorsey, Jorge Flores, DeAnna Friedman-Klabanoff, Shirley George, Nancy Greenberg, Hassan Haji, Elizabeth Hammershaimb, Susan Holian, Leslie Howe, Myounghee Lee, Alyson Kwon, Kirsten Lyke, Alma Valle Maldonado, Jennifer Marron, Kaitlin Mason, Monica McArthur, Rosa McBryde, Sherry McCammon, Sandra Molina, Kathleen Neuzil, Daniele Nitkowski , Justin Ortiz, Rekha Rapaka, Mardi Reymann, Toni Robinson, Wanda Somrajit, Mark Travassos	University of Maryland, School of Medicine	Baltimore, MD
Mark E. Kutner, MD	Amanda Colina, Isett Caro, Frances Beltran, Jessie Downs, Jonathan Fernandez, Mariete Renden, Mirnaya Mujica-Alabaci, Susel Figueredo, Yanelis Dominguez, Jaime Blandon, Bryan Ruiz, Leidy Montoya, Edgardo Rodriguez, Jessie Downs, Jason Rothscheld, Janett Acle, Yaime Martinez, Soraya Ricardo, Maria Hernandez Moran, Eloisa Guerra, Heidie Perez, Claudia Rodriguez, Victoria Moreno, Vanessa Hechavarria, Saray Carvajal, Daniel Lopez, Carlos Ivircu, Neiner Enriquez, Paola Garcia, Chris Hoyle, Marianela Carvajal, Janet Mendez, Edisleidy Mesa, Marco Ramirez, Dalila Del Valle, Jennifer Ortega, Yeni Hernandez, Jhobana Vargas, Carmen Amador, Juan Delgado, Maury Santos, Meredith Arguelles, Leyanis Coello, Vanessa Ansorena, Jorge Caso, Stacy Machado, Raydel Valdes, Giann Lightbourn, Dayami Dovales, Alain Chang	Suncoast Research Group	Miami, FL
Mimi Van Der Leden, MD, PhD	Chrishea Harvey, Tricia Oyeyemi, Aicha Moutanni, Stephanie Melton, Peta-Gay Jackson Booth, Jennifer Yoon, Gloria Kim, Atanas Filev, Francis Uwandi, Meyling Lopez, Janice Spreitzer, Courtney Gennes, Xiangfei Cheng, Matthew Van Sickle, Nick Bart, Brianne Okunji, Frank Maloba	Optimal Research	Rockville, MD
Michael L. Levin, MD	Brennan Opanasenko, Yajaira Ramos, Shonda Lester, Rebecca Boucher, Shawn Harrell, Shon Boucher, Patti Sanchez, Nina Scharbach, Alex Sanchez, Shyane Raniello, Wendy Guerra, Krystal Tyner, Kimberly Temple, Ruby Ortiz, Daniel Terreault, Amy Kill, Jade Odynski, Adolfo DeLeon, Debbie Carter, Eduardo Rodriguez, Julia Gass, Sara Esparza, Sierra Dansbee, Tammy Harrison, Marcy Kulic, Lucian Cappoli, Mora KIm, Matthew Fenner, Heather Jimenez, Shraddha Dubal, Julie Hussey	WR-Clinical Research Center of Nevada	Las Vegas, NV
Michael Lewis, MD	Nancy Mohler, Mai Pham, Ron Waldorf, Elham Ghadishah, Samantha Feril, Stella Lee, Dzuyen Nguyen, Ruoxiang Wang, Justine Velandria, Benjamin Dreskin, Joseph Yusin, Lauren Vigil, Sara Wong, Suchi Tiwari, Joseph Pisegna, Sunita Dergalust, Wayman Lee, Krissa Caroff	VA Greater Los Angeles Healthcare System	Los Angeles, CA
Gregg H. Lucksinger, MD	Jaleh Ostovar, Craig Koch, Danuel Hamlin, Kelly Chase, Jeanette Dickhaus, Edward Kerwin, Frederick Forde, Allison Alvord, Dawn Stewart, Dan Hamlin, Kevin Parks, Ryan Israelsen, Kary Kelly, Tiffany Smith, Melissa Myers, Ryan Rackley, Audrey Kuehl, Savannah Peterson, Hannah Hall, Jay Weisbart, Alison Dodenhoff, Emily Kelly	Velocity Clinical Research	Medford, OR
Mary Beth Manning, MD	Carol Salango, Alec Ireland, Lisa Hoagland, Jeanette Dickhaus, Toby Briskin, Joan Rothenberg, Michael Gaston, Sharita Tedder-Edwards, Denise Roadman, Megan Sokolowski, Tina Shickluna, Katherine Bielanski, Samantha Hood, Talia Chandler, Brianna Arman, Melinda DeLong, Naqib Ahmad, Karly Tarase, Jade Svoboda, Lisle Merriman, Melisa Sebera, Emma Landskroner, Amy Maroun, Brooke Glivar, Jennifer Gaston, Sarah Dzigiel, Cassiandra Uminski, Karol Sabol, Devan Patel, Nick Zarbo, Briana Jackson, Brian Sharpe, Nicole, Baitt, Kaitlyn Duffy, Gabrielle Jacobs, Ann Czuprun, Tracee Cash, Diamond Ivey, Kaitlyn Rubell	Rapid Medical Research	Cleveland, OH
Kristen Marks, MD	Grant Ellsworth, Tina Wang, Timothy Wilkin, Mary Vogler, Carrie Johnston, Marshall Glesby, Roy Gulick, Ole Vielemeyer, Rebecca Fry, Todd Stroberg, Caitlin Rhoades, Noah Goss, Shaun Barcavage, Valery Hughes, Jonathan Berardi, Caroline Greene, Sarah Galloway, Caique Mello, Ashley Machado, Mia Crowley, Monique Williams, Katherine Fee, Elizabeth DeJesus, Andrew Yu, Minkyung Lee, Susan Herder, Mary Ann Zweibel, Patrice Weller, Antonio Rivera-Lopez, Edward Kenny, Hetal May, Natella Fridman, Parul Shah, Ruby Lee, Venus Fernandez, Victoria Lesina, Celine Arar, Byron Bullough, Kinge-Ann Marcelin, Brian Mangano, Jessenia Fuentes, Jiamin Li, Genessi Rodriguez, Catherine Jerry, Nadi Islam, Liqun Cai, Wayne Burns, Akinbayo Caulcrick, Andrika Thomas, Barbara Batog, Guoan He, Sara Yoder, Tamara Crowder, Gianna Resso, Sophia Alvarez, Tahera Begum, Elizabeth Connolly, Roxanne Rosario, Paul Kim, Steven Wang, Vasilika Koci	Cornell Clinical Trials Units - Weill Cornell Chelsea and Uptown	New York, NY

Principal Investigator	Study Team	Institution	Location
Judith Martin, MD	Alejandro Hoberman, Timothy Shope, Gysella Muniz, Sonika Bhatnagar, Kumaravel Rajakumar, Anne-Marie Rick, Peri Unligil, Jennifer Nagg, Melissa Andrasko, Mary Ann Sieber, Jennifer Opal, Lalicia Roman, Spenser Kinsey, Michelle Burke, Matthew Lee, Dominic Kramer, Linette Milkovich, Emily Dougherty, Emily Carney, Shannon Mance, Nader Shaikh, Diana Kearney, Jamie Fries, Lisa Vavro, Shayla Goller	UPMC University Center	Pittsburgh, PA
John W. McGettigan, Jr., MD	Walter Patton, Jennifer Schnider, Riemeka Brakema, Heeten Desai, Mikell Brett Karsten, Patricia Jalomo, Cindy Finch Benoy, Karin Choquette, Jonlyn McGettigan, Yvonne De Los Reyes, Melissa Cozzens, Amanda Hermosillo, Cindy Montgomery, Susan Tarwid, Annette Elzy, Tianna Young, Saysamone Banks, Cristina Fernandez, Damaris Atondo, Zoe Sesma, Norma Barrientos, Maggie Tono, Kisha Adams, JoAnn Wilkins, Arianna Bermudez, Carol Sayer, Julie McDowell, Angelina Navarro, Mercedes Sullivan, Crystal Mata, Sheldon Gingrich, Aaliyah Sestiaga, Gia Longo	Quality of Life Medical & Research Centers	Tucson, AZ
Mark Montgomery McKenzie, MD	Tiffany Jewell, Zackery Harmon, Michael Elizabeth, Christy Sweet, Teresa Deese, Catherine Schon, Misti Earwood, Lou Cappoli, Brennan Opanasenko, Lisa Guider, Michelle Forgey, Justian Jarrett, Rachel Scott, Elizabeth Michael, Erica Osmundsen, Andrew Wood, Shelly Brooks, Gisela Heintz, Lilian Nukuna	WR-ClinSearch	Chattanooga, TN
Vicki E. Miller, MD	Sajjad Naqvi, Soofia Masood, Fredric Santiago, Sonia Guerrero, Subhash Koneru, Nirja Shah, Andrea Torres, Ramani Gali, Talha Baig, Heather Leary, Afifah Ayub, Nayab Goher, Patti Tate, Reagen Reed, Muhammad Irfan, Amy Starr, Alefiyah Motiwala, Julia Kenny, Victoria Aguilar, Jessica Arguijo, Insiya Valika, Victoria Aguilar, Jagruti Patel, Anna Pena, Faryal Mahmood, Blanca Gomez, Nancy Torres, Kristyn Latil, Tarori Mark, Laura Djampou, Lindsey Kueng, Marianne Tadros, Mohammad Millwala, Monica Murray, Murtaza Marvi, Shivani Shah, Vanessa Gonzalez, Zohair Harianawala, Zainab Rizvi, Ambily Dileep, Jaquelyn Gonzales, Ragen Powell, Carolina Deandres, Syed Fahad Ali Kazmi, Sandra Natalia Perez, Shannon Amacker, Shiela Varghese	DM Clinical Research	Tomball, TX
Gowdhami Mohan, MD	Rodolfo Barrera, Emma Partin, Kelly White, Ashley Rochester, Charles Thompson, Stefanie Tyson, Ashten Sheriff, Alyssa-Kay Peay, Kayla Corn, Barbara A. Richardson, Kristin Miller, Steven Clemons, Cameron King, Emma Partin, Gary Clemons, Brianna Starr, Danyel Johnson, Taylor Davis, Niki Tyson	VitaLink Research	Anderson, SC
Kathleen M. Mullane, DO, PharmD	David L. Pitrak, Cheryl Nuss, Karen Cornelius, Randee Estes, Amy Luckett, Michelle Moore, Judi Pi, Stephen Schrantz, Jill Stetkevych	University of Chicago	Chicago, IL
Joseph Lee Newberg, MD	Mary Reyes, Nicole Leahy, Victoria Andriulis, Herbert Whinna, Patricia James, Lana Ghomrawi, Carole Kempfer, Miriam Arroyo, Maria Castro, Anna Maddox, Reuben Martinez, Jacquilyn McCormick-Burks, Laura Pearlman, Rosalinda Vazquez, Shaheera Suleiman, Neha Atal, Rosalind Vazquez	Synexus Clinical Research	Chicago, IL
Richard M. Novak, MD	Regina Harden, Maria Schwarber, Michael Pacini, Rebeca Gansari, Margie Villarreal, Stephanie Martin, Michelle Lee, Richard Morrissy, , Taylor Ellis, Samuel Rene, Tara Cobbs, Claudia Preciado, Scott Borgetti, Maximo Brito, Olamide Jarrett, Mahesh Patel, Tracy Cable, Charity Ball, Maryann Holtcamp, Rodrigo Burgos, Sarah Michienzi, Emily Drwiega, Mikayla Johnson, Fischer Herald, Benjamin Ladner, Minseung Chu, Carolyn Dickens, Alfredo Mena Lora, Stockton Mayer, Andrea Wendrow, Habiba Sultana, Nanu Nunwar, David Chan, Marla Schwarber, Khandaker Anwar, Mahmood Ghassemi, Md Ruhul Amin, Doris Carroll, Rosa Valencia, Michelle Agnoli, Elena Llinas, Samuel Rene, Liam Morrissy, Adrian Raygoza, Addis Mekkonnen, Lisa Lindemann, Daniel Meslar, Karen Pacini, Corey Ringhisen, Amy Kennedy-Krage, Claudia Miller, Lorna Sanchez McCann, Gizelle Alvarez, Nia Moragne-Oneal, Nusirat Williams, Ian Feather, Nikki Griffith, Wardrick Nealon, Renyce Powell, Nila Safaeian, Monica Gingell, Diana Bahena, Gerald Beck, Brad Farrington, Rod Reyes, Monica Wilson, Juline Wondrasek, Kimberly Shapiro, Shannon Whitted, Victoria Roehl, Braulio Carrasco, Michael Chen, Olivia Murray, Yasiel Lacalle, Tessa Eckley, Anna Schluckebier, Kevin Cao, Elise DeBruyn	University of Illinois at Chicago - Project WISH	Chicago, IL
Paul Joseph Nugent, DO	Leonard Singer, Jennifer Jones, April Smith, Georgettea Geuss, Lana Ghomrawi, Christine Bennett, Norma Blevins, Linda Brotherton, Michele Byrd, Krista Doss, Victoria Holden, Christine Hull, Jean Montgomery, Nancy Cipollone, Savanah Torline, Brandon Brown, Meagan Thomas, Katie Ziska, Dana Sias, Hannah Wagner	Synexus Clinical Research	Cincinnati, OH
Jeffrey Scott Overcash, MD	Hanh Chu, Kia Lee, Karla Zepeda, John Rodriguez, Adam Prince, Yashveer Dubbula, Elizabeth Tomatsu Michael Voskanian, Crystle Rajania, Stephanie Ramirez, Claudia Camacho, Lauren Arnett, Kecia Darbeau, Ashley Smith, Kimberly Quillin, Cesar Ramirez, Daniel Robitaille, Erica Sanchez, Allie Davis, Michael Waters, Pat Kappen, Valerie Horne, Thao Vuong, Andrew Dennis, Nikki Abels, Dominique Panis, Richard McQuaid, Whitley Harbison, Erika Trujillo, Andrea Garcia, Jose Jacob Esparza, Carlos Vera, Raquel Taitingfong, Cathy Meza, He Pu, Jackielynn Smith, Shandel Odom, Zahira Nieves, Ashliegh Lindsay, Ariana Nasatka, Jose Cazarez, Nora Martinez, Angela Hunt, Antonio Delgado, Linda Vega, Angela Anorve, Erica Martinelli, Melania Riordan, Sylvia Lindholm, Gina Ciezkowski, Grecia Perez, Jacob Pineda,	Velocity Clinical Research, San Diego	La Mesa, CA

Principal Investigator	Study Team	Institution	Location
	Nathan Tyler, Ranya Salem, Amara Yilmaz, Jessica Gonzales, Zabrina Ruiz, Laura Castillo, Yajaira Contreras, Angelica Guzman, Makenna Orel, Jeffery Alvarez, Gordon Bovee, Roxana Ramirez, Joan Esquivel		
James Todd Peterson, MD	Christopher Mickelson, Madeline Maldonado, Alison Charlton, Ashley Bragg, Sean Hansen, Emily Wilcox, Colby Bostock, Megan Henry, Pam Iwasaki, Bradley Young, Katelyn Walker, Joy Nguyen, Lindsey Bevan, Megan Grimmett, Madeline Grote, Heather Littell, Natalie Bee, Alexander Clark, Shana Eborn, Susan Edwards, Dan Henry, Heather Jackson, Gerald Kelty, Issac Pena-Renteria, Jacqueline Rohrer, Jack Taylor, Brooke Barrick, Ty Henry, Anna Dansie, Kenadie Hamblin	J. Lewis Research	Salt Lake City, UT
Paul Pickrell, MD	Susan Bonner, Blaire Graham, Staci Taggart, Hussain Malbari, Tiffany Lemuz, Ethan Shotton, Andrew Bell, Megan Malek, David Pampe, Carol Ann Linebarger, Michelle Peterson, Brandi Chalman, John Luna, Elizabeth Santellanes, Christina Martinez, Lisa Johnson, Lisa Savage, Melissa Winn, Wendi McKenzie, Eileen Euperio, Stefanie Mott, Paul Menefee, Katie Caballero, Darrell O'Brien, Morgan Schulle, Kate Jurek, Olivia Hapanowicz	Tekton Research, Inc.	Austin, TX
Terry L. Poling, MD	Meenakshi (Kavya) Natesan, Patricia Contreras, Denise Hole, Avi Woods, Jill Hiebert, Melissa Burton, Olivia Eagleson, Laura Holz, Terri Ford, Cindy Thome, Terry D Klein, Gregory Greer, Diandra Henriques, Tracy R Klein, Thomas C Klein, Christa Shue, Gina Young, Brenna Sprout	Alliance for Multispecialty Research	Wichita, KS
Bruce G. Rankin, DO	Jennifer Dittman, Lora Parahovnik, Crystal Paccione, Melissa Hodges, Katina Marchione, Matt Maxwell, Any Dominy, Diana Toney, Andrea Marrafino, Laura Isbell, Leandro Fernandez, Claxton Copeland, Michelle Tutt, Adam VanDeusen, Kevin Feldman, Clark Mason, Tifany Huertas, Over Seijas, Jennifer Cline, Christian Beierschmitt, Ryan Hobbick, Jessica Gilliam, Jeanette de Leon, Iman Mencia, Daniel Layish, Vienna Bauer, Shatonia Fields, Albert Garcia, Carrie Rycort, Tasha Brocato, Marshall Nash, Samantha Watts, Amy Houck-Dominy, Angela Hammerle, Teresa Logsdon, Erika Wierzbicki, Taylor Martin, Ranie Hutchins, Fadhel Alyunis, Gail Lavine, Jeffery Hood, Robert Duran, Michelle Jones, Ginny McClanahan, Heather Jackson, Leandra Fernandez, Douglas Winter, Antonio Rivera, Amber Vasquez, Thais Truffa, Daniel Campbell, Grace Newcomb, Elizabeth Orlando, Steven Shinn, John Hill, Christina Isbell, Dhaneshwar Oomrow, Alicia Cevera	Accel Research Sites	DeLand, FL
Michele Diane Reynolds, MD	Jennifer Bashour, Robert Schmidt, Cynthia Mayeux, Uvoka Huffman, Lisa Nicholson, Jacklyn Newton, Lynn Yauch, Cathy Monroe, Kathleen Carty, Angelica Banks, Taylor Werner, Pamela Echols, Pauline Jackson, Chana Hines, Lorine Cook, Cristina Puig, Patrick Brooks, Jennifer Ruiz, Deanna Bowman, Ladina Garcia	Synexus Clinical Research	Dallas, TX
Rambod Rouhbakhsh, MD, MBA	John Johnston, Richard Calderone, Tasha Stevenson, Tameka Fortune, Brandi Pace, Adreanna Pou, Jerrica Sullivan, Yolanda Lewis, April Rouse, Tiffany Jefferson, Elizabeth Danford, Jeff Repper, Mason Boutwell, Alexycia Washington, Krista Hirth, Meagan Grabel	MediSync Clinical Research Hattiesburg Clinic	Petal, MS
Nadine Rouphael, MD	Renata Dennis, Tigisty Girmay, Michelle Wiles, Sharon Curate-Ingram, Lauren Hewitt, Alexis Ahonen, Mari Hart, Sarah Bechnak, Erin Carter, Lauren Nolan, Daniel Sans Graciaa, Geoffrey Kamau, Easton Beshears, Sy Tran, Mary Atha, Mary Bower, Ghina Alaaedine, Brandy Johnson, Jacob Usher, Eileen Osinski, Erin Scherer, C. Tae Stallworth, Stephanie Ramer, Rose Pope, Esther Park, Francine Dyer, Laura Clegg, Rebecca Gonzalez, Stacey Wheeler, Susan Rogers, Vy Ngo, Vanessa Soliman, Kristen Unterberger, Bernadine Panganiban, Christopher Huerta, Juton Winston, Ali Alvarez, Jianguo Xu, Colleen Kelley, Paulina Rebolledo, Nicholas Scanlon, Jessica Traenkner, Matthew Collins, Hollie Macenczak, Cassie Grimsely-Ackerley, Tiffany Lee, Amy Anderson, Michele Paine McCullough, Hannah Huston, Daniella Carter, Lisa Harewood, Srilatha Edupuganti, Varun Phadke, Mindee Adamson, Jeanne Allen, Debbie Bartenfeld, Lily Berz, Amy Cromwell, Sergio Cruz, Fred Ede, Monica Godfrey, Evan Gutter, Angelle Ijeoma, Sara Jo Johnson, Vinit Karmali, Dean Kleinhenz, Jennifer Kleinhenz, Alexandra Koumanelis, Maranda Leary, Tiraje Lester, Juliet Alise Morales, Shashi Nagar, Julia Paine, Dilshad Rafi Ahmed, Brittany Robinson, Amanda Rosner, Renee Silver, Trevor William Simon, Talib Sirajud-Deen, Damien Swearing, Maliya Tolbert, Pamela Turner, Chia Uzuegbunam, Claire Wan, Dongli Wang, Erika Wimberly, Jean Winter, Joy Winters, Yong Xu, Sha Yi	Emory University - Hope Clinic	Decatur, GA
Richard Rupp, MD	Amber Stanford, Megan Berman, Laura Porterfield, Gerianne Casey, Hala Ghoson, Doreen Jones, Michael Willig, Cori Burkett, Robert Cox, Amy McMahan, Diane Barrett, Kristin Pollock	University of Texas Medical Branch	Galveston, TX
Jamshid Saleh, MD	Matthew Miles, Rafael Lupercio, Vicky Martin, Marla Clark, Matthew Pohlmeyer, Ruba Zanaid, Veronica Blevins, Tara Ulberg, Carlyee Chambers, Marisol Corrales, Emily Crews, Mohamed Yassin, Sarah Sandberg, Frank Chen, Mandy Swanson	Paradigm Clinical Research Center	Redding, CA
John W. Sanders, MD, MPH	Stacy Harpe-Hall, Jesse Hopkins, Ann Schweppe, Jaymous Fayssoux, Kathryn Bender, James Peacock, Katharine Pearsall, Brandy Snyder, Deidre Knox, Megan Thorpe, Melissa Ellingson, Brittany Bundeff, Lisa Ashworth, Meredith Hiatt, Ritu Rathee, Stacy Woodliff, Brian Strittmatter, Amanda Wright, Daisy DeWeese-Gatt,	Wake Forest University Health Sciences	Winston Salem, NC

Principal Investigator	Study Team	Institution	Location
	Caryn Morse, John Williamson, Samantha Wheeler, Lori Whiteheart, Susan Donahue, James Lovette, Kaitlyn Van Leuvan, Kelly Ledbetter, Scott Chatterton, Julio Nasim, Amie Sidberry, Ashley Davis, Carter Noecker, Chie Hooker, Johanna Breenan, Sam Cable, Anna Bowman, Stephanie Boothe, Shea Overcash		
Howard I. Schwartz, MD	Carlos Valladares, Jocelyn Morrera, Yulexis Amestoy, Tori Wallenburg, Thelma Beltran, Terry Piedra, Monica Garces, Alexandra Galvis, Wanda Delgado, Catherine Casas, Lesly Miguel Sosa, Vivian Rosales, Jose Fernando Henriquez, Mikael Yaniz, Beatriz Rivera, Peter Ventre, Gabriella Huyke, Maria Companioni, Jessie De Vega, Brianna Gamez, Stephanie Diaz, James Jean-Mary, Americo Padilla, Nikita Notise, Yorlina Luquetta, Monifa Wilson-Morris, Kenia Gutierrez, Roilan Garcia, Karla Pentzke, Leyda Valentin, Lazara Novas, Marilein Camacho, Jazmin Henfield, Laymis Alvarez, Myriam Rosado, Maxine Bryant, Maria Pinero, Laura Raucci, Francisco Ramirez, Angelic Gamez, Mailin Perez, Yasmin Baddour, Hary Leon Joseph, Yaquelin De la Cruz, Dunia Torres, Rosaidaliz Carreira, Chanella Garcia, Surisaday Mederos, Jose Muniz, Karenda Plotka, Sara Gomez, Maria Soto, Cathy Cruz, Nelia Sanchez-Crespo, Jennifer Schwartz, Barbara Corral, Matthew Muniz, Dayana Deltejo, Ana Castro, Reem Hassan	Research Centers of America	Hollywood, FL
Nathan Segall, MD	Michelle Sowell, Nancy Levine, Erynn McKinley, Hannah Smith, Karen Hickson, Elizabet West, Patrizia Greene, Jon Finley, Mildred Stull, Susan Jones, Jennifer LeBrun, Pamela Talbott, Kwannda Whatley, Jeffrey Jones, Michelle Binns, Donna Toepfer, Cynthia Steele, Grace Newville, Gillian Waite, Cynthia Pinckney, Karen Yangapatty, Kiara Tyner, Kimberly Cobb, Kourtney Richardson	Clinical Research Atlanta	Stockbridge, GA
William Seger, MD	Kimberly Pullen, Jean Seignon, Anthony Kim, Mohammed Antwi, Allison Green, Lizzy Seger, Elizabeth Boydston, Abdur Rafay Qadri, Deborah Devlin, Tasha Todd, Oluwatosin Akingbala, Alma Guel, Tisha Davis, Melody Dufrene, Samantha Loudermilk, Virginia Loudermilk, Crystal Starr, John Villegas, Ben Seger, Katherine Hollie	Benchmark Research	Fort Worth, TX
Neil Parmanand Sheth, MD	Kenneth Stell, David Beckett, Enitt Gonzalez, Donna McGunigal, Amanda Burns, Nancy Wood, Shelley Miceli, Christina Avila, Rebecca Baker, Laura Vigliotti, Sarah Kading, Samer Salama	Synexus Clinical Research	Glendale, AZ
William B. Smith, MD	Richard L Gibson, Jennifer Winbigler, Elizabeth Parker, Madison Watts, Suzann Cloninger, Talya Thomas	Alliance for Multispecialty Research	Knoxville,TN
Joel Solis, MD	Martha Carmen Medina, Xavier Morales, Hank Heller, Blake Torrence, Joanna Gurrola-Mahoney, Cynthia Bueno, Heather Holloway, Irving Salinas, Joel Perez, Paola Garcia, Erica Canales, Blanca Urbina, Brancisilio Gutierrez, Carolina Cantu, Chelsea Vargas, Cindy Vasquez, Cody McIntire, Gabriela Gutierrez, Hugo Sosa, Irvin Munoz, Jessica Estrada, Jonna Lopez, Kaegan Knox, Mirella Melendez, Natalia Valle, Natalie Echavarria, Nicole Litton, Amber Victor, Nancy Torrence, Madhu Shreya, Mathew Maran, Asfak Alam, Westly Keating, Tara Green, Devora Torrence, Gerardo Sedas, Shruti Konda, Prem Jangam, Mario Echavarria, Alejandro Silva, Anne McNulty, Daniel Contreras, Daniel Gomez, Edgar Garcia, Elizabeth Weber, Luis Lopez, Samuel Ramirez, Kayla Lopez, Pedro Penalo, Angel Salinas, Jaime Solis, Shannon Moyer, Aryana Ibarra, Guadalupe Gurrola, Jenna Anastasiades, Uchechi Ehiemua, Sara Solorzano	Centex Studies, Inc.	McAllen, TX
Stephen A. Spector, MD	Amaran Moodley, Jill Blumenthal, Baharin Abdullah, Christina Addington, Juan Carlos Alcantar, Deyna Arellano, Bernadette Cale, Brendan Costello, Tammelita Cotlon-Pineda, Fanny Delebecque, Karen Deutsch, Aram Dimayuga, Son Do, Yasmeen Esshaki, Aileen Everhart, Cindy Ewing, Veronica Figueroa, Medardo Gaytan, Crystal Groom, Carolyn Hernandez, Heather Huitema, Benjamin Hull, Sylvia Isaac, Jaclyn Jaskowiak, Cindy Knott, Leander Lazaro, Thuan Le, Megan Loughran, Michelle Madey, Rosalva Martha-Patten, Colleen McLellan, Jeff Ledford-Mills, Asami Mimura, Patty Moraes, Jennifer Morales, Jessica Nasca, Phirum Nguyen, Marielys Padilla-Martinez, Dennis Perpetua, Mike Pizza, Shannon Ransom, Emily Rizo, Carlos Rojas, Thaine Ross, Marie Sagrado, Eugene Sato, Lisa Stangl, Ji Sun, Nancy Tang, Mina Trivedi, Rodney Trout, Donna Voss, Lindsey Woronicz	University of California, San Diego	La Jolla, CA
Cynthia Becher Strout, MD	Rica Santiago, Yvonne Davis, Patty Howenstine, Alison Bondell, Jaime Robertson, Anissa Moussa, Geronimo Feria Garzon, Sierra Bennett, Marlena Petrie	Coastal Carolina Research Center	Mount Pleasant, SC
Shobha Swaminathan, MD	Amesika Nyaku, Tilly Varughese, Rondalya Deshields, Michelle L DallaPiazza, Elise Lewis, Jennifer Punsal, Mario Portilla, Malithi Desilva, Christina Daliani, Susana Rivera, Aidan Ziobro, Andressa Rebellatto, Brian Murloy, Christina Ninan, Ernest Pianim, Eunice Wang, Merit Henen, Muhammad Usman, Rebecca Kim, Shiao Wang, Gener Eric Cruz, Bethany Birago, Joyell Arscott, Dina Meawad, Christie Lyn Costanza, Francesca Escaleira, Zoraida Cruz-Barahona, Jared Khan, Valeria Cadorett, Jamir Tuten, Travis Love, Eric Asencio, Sukhwinder Singh	Rutgers New Jersey Medical School	Newark, NJ

Principal Investigator	Study Team	Institution	Location
Ramy Joseph Toma, MD	Olivia Graves, Josiah Robinson, Patricia Hammonds, Lana Ghomrawi, Kara Quinnelly, Shaun O'Conor, Michael Lambe, Rachell Stewart, William Kirby, Pink Folmar, Rachel Culbreth, Heidi Leblanc, Julie McDaniel, Rian Montgomery, Andrea Woodle, Samantha Williams, Hunter Russell, Shereen Lowe, Maureen Mayer, Hollis Ryan, Elaine Reese	Synexus Clinical Research	Birmingham, AL
Timothy P. Vachris, MD	Mark Hutchens, Stephen Daniels, Margaret Wells, Sandra Clancy, Rebecca Martinez, Jessica Buot, Merissa Daugherty, Julie Hamilton, Kimberly Hernandez, Ashli Alejandro, Amy Collins, Monique Gawlik, Patricia Johnson, Maria Moreno, Ashley Washington, Tina Rountree, Daniel Dore, Ravi Davuluri, Ashlee Brunaugh, Jorge Martinez, James Hermon, Vianai Carreno, Mia Rountree, Colleen Coelho	Optimal Research	Austin, TX
Keith William Vrbicky, MD	Charles Harper, Chelsie Nutsch, Wendell Lewis III, Cathy Laflan, Linden DeBoer, Kayla Andal, Misty Appeldorn, Jenniger Grebe, Russell Herstein, Catherine King, Samantha Wieseler, Alisha Kiepke, Christy Lee, Kelsey Kelley, Kelli James, Ashley Frisch, Courney Green, Taysha Hingst, Jeni Hoppe, Kimber Breeden, Debra Gabrielson, Ginny McNew	Meridian Clinical Research	Norfolk, NE
Larkin T. Wadsworth III, MD	Ashley Dale, Christy Schultz, Rebecca Munsch, Anya Penly, Liz Garner, Stephanie Tesson, George Cherniawski, Angie Kean, Dan Reed, Courtney Kubiak, Maureen Dempsey, Heather Cherniawski, Breanna Galibert, Kristin Branson, Laura Hartupee, Karen Knapp, Horacio Marafioti, Lyly Dang, Jennifer Berry, Lauren Clement, Megan Dandurand	Sundance Clinical Research	St. Louis, MO
Jordan L. Whatley, MD	Patricia Whatley, Christopher Dedon, Anika Payne, Amie Shannon, Kristen Losavio, Nicole Harrell, Mary Margaret Dobson, Lindsey Hall, Chaney Bennett, Crystal Rowell, Mimi Dimmick, Amy Thomassie, Kimber Breeden, Cody LaFleur, Makaylea Truitt, Taryn Collett, Emily Best, Alexandra Caillouet,	Meridian Clinical Research	Baton Rouge, LA
Judith White, MD	Amy Edridge, Chelsea Montalvo, Eugenia Clark, Lisa Russell, Zahra Somji, Lesli Leimer, Robert Meyer, Christine Murphy, Prity Patel, Sejal Patel, Ruben Moliere, Samantha Merveillard, Yarnick Mirjah, Bryn Walls, Joey Cruz, Aaron Cooper, Jessica Bienaime, Ashley Gilcrist, Alisa Petit, Tyler Knightly, Kimberly Stokes, Christina Rosario, Talhia Matos, Ilona Boggs, Nicholas Weber, Felix Busot, Linda Colon, Heather Gillenwater, Cristina Kaplun, Melissa Caputi, Shayna Siplin, Daminee Shah, Samuel Martin, Alexis Waldorf, Vihar Upadhyay, Adolfo Henriquez, Saskia Singh, Maria Roberts, John Caporelli, Shirley Salvador, Quevina Scarver, Vanessa Garcia, Taylor Moore, Jayasen Singh, Curshinda Galvin-Burch, Mary Kesner, Jasmin Gil, Shay Gray, Steven Monsegur, Michele Steinmetz, Michael Lambe, Heather Powell, Sandra Torres, Shaban Katbeh, Taylor Wilson	Synexus Clinical Research	Orlando, FL
Priyantha N. Wijewardane, MD	Natalie Johnson, Martha Evans, Sondra Wright, Richard Pellegrino, Lastida Burns, Natasha Williams, Haylee Rowe, Kayla Graham, Amanda Horn, Eric Bravo, Jeffrey Thessing, A. Michele Maxwell, Amy Cooper, Lauren Evans, Tonya Cato, Haylee Tucker, Lesa Gann, Hannah Jones, Amanda May, Tiffany Walker, A. LeiAn Diaz, Laura Khalil, Lydia Purcell, Timothy Campbell, Charlotte Garcia-Velez, Andrea Scarborough, Beatrice A. Miller, Keith Bracy, Aujania Thompson, Cassandra Johnson, Krishana Day, Freddie Hicks, Jamie Pettus	Baptist Health Center for Clinical Research	Little Rock, AR
Barton G. Williams, MD	Flo Abbott, Nicole Burton, Alice Cipollini, Madison Croucher, Philip Dattilo, Erin Harrelson, Kelsey Heston, James Ingram, William H Jones, Karla Lane, Brandy Lowman, Evan Lucas, Megan Marles, Morgan Mathis, Angie Northcott, Clyda Pasquantonio, Alyssa Valente, Ciara Winders, Stephanie Graham	Trial Management Associates	Wilmington, NC
Marcus J. Zervos, MD	Paul Kilgore, Mayur Ramesh, Jelena Verkler, Pardeep Pabla, Andrew Clark, Katrina Williams, Dee Dee Wang, Beverley Duthie, Samia Arshad, Alandra White, Anna Kern, Ashley Mattern, Bilqis Mosed, Dana Parke, Doreen Dankerlui, Dragana Spasevska, Hanah Woods, Helina Misikir, Howard Klausner, Janay Scott, Jessica Heinonen, John Zervos, Joseph Miller, Kate Zenlea, Kristin Eis, Marissa Vasquez, Maurice Slaughter, Meaghan Flynn, Michael Garcia, Michelle Sankah, Nina Paeilli, Philip Benson, Robert Devore, Stevanya Baho, Tony Eljallad, Tyler Prentiss, Yaman Ahmed, Sharon Mathys, Linda Kaljee, Jeffrey Van Laere, Claudia Hanni, Hassan Zafar, Mona Desai, Gina Maki, Mary Perri, Dora Vager, Shannon Thomas, Autumn Robinson, Isis Hamilton, Sonia Eliya, Jehan Jazrawi, Biljana Popovic, Sharon Zahul, Joshua Ruzzin, John Laguio, Ali Mathena, Bobby Cook Jr., Marlene Hesler, Rochelle Fleming, Terria Minniefield, John Simons, Sherese Henderson, Ashley Hopkins, Rebecca McFarlane, Raeshell Carson, Jonathan Williams, Katherine Reyes, Erica Herc, Indira Brar, Mayur Ramesh, John McKinnon, Lacquis Duncan, Tim Asmar, Margaret Beyer, Kaleem Chaudhry, Madison Lee, Jo-Ann Rammal, Karthik Sridasyam, Siddesh Veer, Angelique Buluran, Kimberlyn Lott, Jeremiah Rooker, Alayna Wilder, Kathleen Wilson, Allison Weinmann, Hassan Mourtada	Henry Ford Health System	Detroit, MI

United States Government (USG)/Coronavirus Prevention Network (CoVPN) Biostatistics Team

(PubMed listed, and ordered alphabetically by institution affiliation)

Affiliation	Team Members
Biomedical Advanced Research and Development Authority (BARDA), Washington, DC	Di Lu, James Zhou
Department of Biostatistics and Bioinformatics, Rollins School of Public Health, Emory University	David Benkeser
Vaccine and Infectious Disease Division, Fred Hutchinson Cancer Research Center, Seattle, WA	Jessica Andriesen, Bhavesh Borate, Lindsay N. Carpp, Andrew Fiore-Gartland, Youyi Fong*, Peter B. Gilbert*, Ying Huang*, Yunda Huang, Ollivier Hyrien, Holly E. Janes*, Michal Juraska, Yiwen Lu, April K. Randhawa, Lars W.P. van der Laan, Chenchen Yu, Bo Zhang
Biostatistics Research Branch, NIAID, NIH, Bethesda, MD	Michael P. Fay, Jonathan Fintzi, Dean Follmann, Martha Nason
Clinical Monitoring Research Program Directorate, Frederick National Laboratory for Cancer Research, Frederick, MD	Eric Chu
Department of Biostatistics, T.H. Chan School of Public Health, Harvard University	Nima S. Hejazi
Department of Biostatistics, University of Washington, Seattle, WA	Marco Carone, James Peng, Charlotte Talham
Department of Statistics, University of Washington, Seattle, WA	Alex Luedtke
Department of Population Health Sciences, Weill Cornell Medical College, New York, New York	Iván Díaz
Kaiser Permanente Washington Health Research Institute	Brian D. Williamson
Department of Biostatistics and Bioinformatics, Duke University; Global Health Institute, Duke University	Avi Kenny

*YF, PBG, YH, and HEJ are also affiliated with the Department of Biostatistics, University of Washington, Seattle, WA. PBG is also affiliated with the Public Health Sciences Division, Fred Hutchinson Cancer Research Center, Seattle, WA.

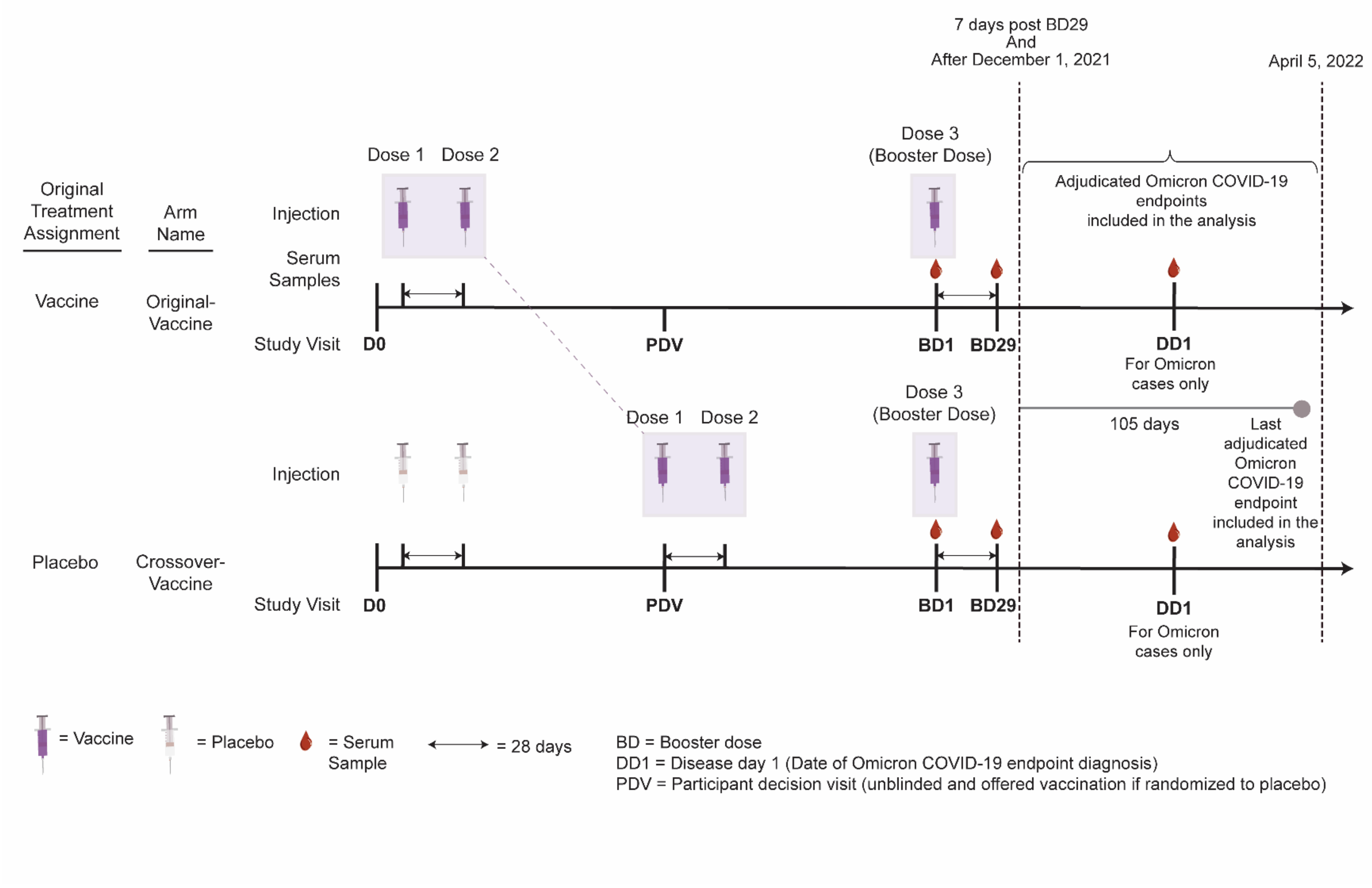

Supplementary Fig. 1. Timing of doses, study visits for serum sampling, and follow-up for Omicron COVID-19 endpoints included in the analysis. The median time interval between the second dose and the third (booster) dose was 12.9 months in the original-vaccine arm and 8.2 months in the crossover-vaccine arm.

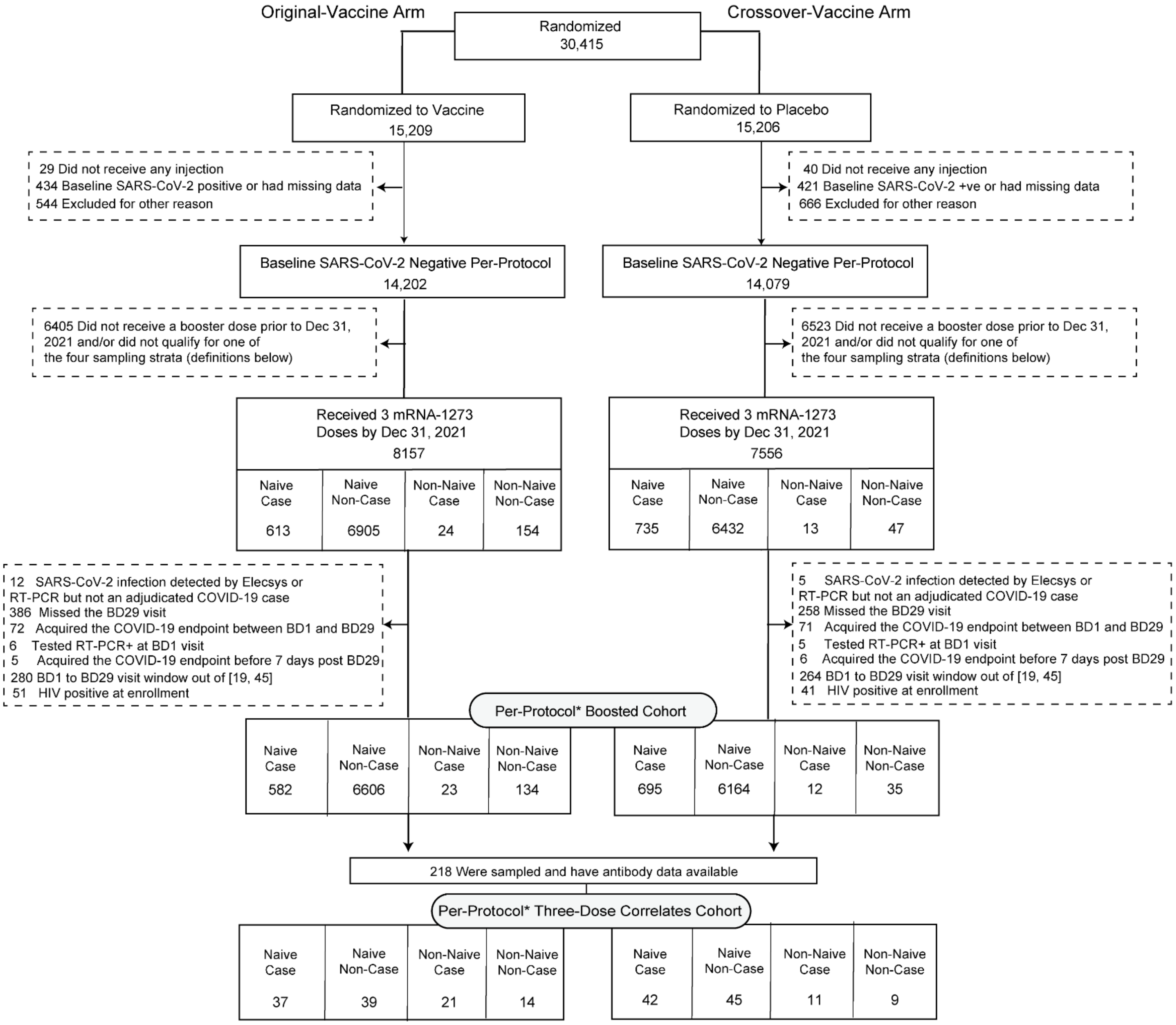

Sampling Strata Qualification:

Naive/Case:	No evidence of SARS-CoV-2 infection detected by Elecsys (Roche) or RT-PCR from enrollment through BD1; Omicron COVID-19 event in the interval [max(BD29 + 7, December 1, 2021), data cut-off date April 5, 2022].
Naive/ Non-Case:	No evidence of SARS-CoV-2 infection detected by Elecsys or RT-PCR from enrollment through BD1; No evidence of SARS-CoV-2 infection detected by Elecsys or RT-PCR in the interval (BD1, data cut-off date April 5, 2022).
Non-Naive/Case:	Any evidence of SARS-CoV-2 infection in the interval [Dose 2 of mRNA-1273 + 14, BD1]; SARS-CoV-2 infection detected by Elecsys or RT-PCR in the interval [max(BD29 + 7, December 1, 2021), data cut-off date April 5, 2022] and the Elecsys test was not positive at BD-D1 pre-booster.
Non-Naive/Non-Case:	Any evidence of SARS-CoV-2 infection in the interval [Dose 2 mRNA-1273 + 14, BD1]; No evidence of SARS-CoV-2 infection detected by Elecsys or RT-PCR in the interval (BD1, data cut-off date April 5, 2022].

*Here, "per-protocol" refers to additional criteria beyond the original per-protocol criteria during the blinded phase as in Gilbert et al. 2022.

Supplementary Fig. 2. Flowchart of study participants from enrollment in COVE through to the per-protocol three dose correlates cohort.

Supplementary Table 1. n = 218 sampled participants in the per-protocol three-dose correlates cohort (Fig. S2) by sampling stratum (N = SARS-CoV-2 naive and NN = Non-Naive) and time period of receipt of third (booster) dose.

	Time Period of Receipt of Third (Booster) Dose (BD1 Visit)								
	Sep 23 to Oct 15, 2021		Oct 16 to Oct 31, 2021		November, 2021		December, 2021		Total
Original-Vaccine Arm × Omicron Case	8N	8NN	11N	5NN	7N	7NN	11N	1NN	37N 21NN
Original-Vaccine Arm × Non-Case	8N	7NN	10N	2NN	8N	4NN	13N	1NN	39N 14NN
Crossover-Vaccine Arm × Omicron Case	9N	5NN	12N	1NN	10N	5NN	11N	0NN	42N 11NN
Crossover-Vaccine Arm × Non-Case	9N	4NN	12N	0NN	10N	4NN	14N	1NN	45N 9NN

Omicron Case= COVID-19 endpoint in the interval [≥ 7 days post BD29 AND ≥ December 1, 2021 to April 5, 2022 data cutoff date]. As described in the SAP (Appendix A) the COVID-19 endpoint is documented to be Omicron BA.1 if possible whereas for some non-naive COVID-19 endpoints there was not lineage data available to document the case to be Omicron BA.1. COVID-19 endpoints were hard-imputed as Omicron BA.1 if the COVID-19 diagnosis date was before January 15, 2022.

Non-case = Did not acquire COVID-19 (of any strain) in the interval [BD1, data cutoff date].

SARS-CoV-2 naive (N) = No evidence of SARS-CoV-2 infection from enrollment through to BD1; Non-naive (NN) = Any evidence of SARS-CoV-2 infection in the interval [≥ 14 days after the first two doses of mRNA-1273, BD1]

Supplementary Table 2. Demographic and clinical information of the per-protocol boosted cohort and the per-protocol three-dose correlates cohort (original-vaccine and crossover-vaccine arms combined)

Characteristics	Per-Protocol Boosted Cohort (Original Vaccine and Crossover Vaccine Combined)			Per-Protocol Three-Dose Correlates Cohort (Original Vaccine and Crossover Vaccine Combined)		
	SARS-CoV-2 Naive (N=14047)	Non-Naive (N=204)	Total (N=14251)	SARS-CoV-2 Naive (N=163)	Non-Naive (N=55)	Total (N=218)
**Age**						
Age < 65	9683 (68.9%)	155 (76.0%)	9838 (69.0%)	121 (74.2%)	44 (80.0%)	165 (75.7%)
Age ≥ 65	4364 (31.1%)	49 (24.0%)	4413 (31.0%)	42 (25.8%)	11 (20.0%)	53 (24.3%)
Mean (Range)	54.1 (18.0, 95.0)	51.6 (19.0, 84.0)	54.0 (18.0, 95.0)	54.4 (20.0, 80.0)	49.3 (21.0, 77.0)	53.1 (20.0, 80.0)
**BMI**						
Mean ± SD	29.3 ± 6.7	30.2 ± 7.8	29.3 ± 6.7	29.9 ± 7.7	29.8 ± 5.7	29.8 ± 7.2
**Risk for Severe COVID-19**						
At-risk	3359 (23.9%)	55 (27.0%)	3414 (24.0%)	43 (26.4%)	14 (25.5%)	57 (26.1%)
Not at-risk	10688 (76.1%)	149 (73.0%)	10837 (76.0%)	120 (73.6%)	41 (74.5%)	161 (73.9%)
**Age, Risk for Severe COVID-19**						
Age < 65 At-risk	2086 (14.9%)	37 (18.1%)	2123 (14.9%)	29 (17.8%)	10 (18.2%)	39 (17.9%)
Age < 65 Not at-risk	7597 (54.1%)	118 (57.8%)	7715 (54.1%)	92 (56.4%)	34 (61.8%)	126 (57.8%)
Age ≥ 65	4364 (31.1%)	49 (24.0%)	4413 (31.0%)	42 (25.8%)	11 (20.0%)	53 (24.3%)
**Sex Assigned at Birth**						
Female	6783 (48.3%)	100 (49.0%)	6883 (48.3%)	94 (57.7%)	23 (41.8%)	117 (53.7%)
Male	7264 (51.7%)	104 (51.0%)	7368 (51.7%)	69 (42.3%)	32 (58.2%)	101 (46.3%)
**Hispanic or Latino Ethnicity**						
Hispanic or Latino	2496 (17.8%)	39 (19.1%)	2535 (17.8%)	32 (19.6%)	12 (21.8%)	44 (20.2%)
Not Hispanic or Latino	11405 (81.2%)	165 (80.9%)	11570 (81.2%)	131 (80.4%)	43 (78.2%)	174 (79.8%)
Not reported and unknown	146 (1.0%)	0 (0%)	146 (1.0%)	0 (0%)	0 (0%)	0 (0%)
**Race**						
White	11189 (79.7%)	156 (76.5%)	11345 (79.6%)	136 (83.4%)	41 (74.5%)	177 (81.2%)
Black or African American	1383 (9.8%)	28 (13.7%)	1411 (9.9%)	14 (8.6%)	5 (9.1%)	19 (8.7%)
Asian	613 (4.4%)	8 (3.9%)	621 (4.4%)	4 (2.5%)	3 (5.5%)	7 (3.2%)
American Indian or Alaska Native	109 (0.8%)	1 (0.5%)	110 (0.8%)	1 (0.6%)	1 (1.8%)	2 (0.9%)
Native Hawaiian or Other Pacific Islander	29 (0.2%)	1 (0.5%)	30 (0.2%)	2 (1.2%)	0 (0%)	2 (0.9%)
Multiracial	310 (2.2%)	6 (2.9%)	316 (2.2%)	2 (1.2%)	3 (5.5%)	5 (2.3%)
Other	267 (1.9%)	4 (2.0%)	271 (1.9%)	3 (1.8%)	2 (3.6%)	5 (2.3%)

Not reported and unknown	134 (1.0%)	0 (0%)	134 (0.9%)	1 (0.6%)	0 (0%)	1 (0.5%)
**Underrepresented Minority Status**						
White Non-Hispanic	10133 (72.1%)	140 (68.6%)	10273 (72.1%)	115 (70.6%)	38 (69.1%)	153 (70.2%)
Communities of Color	3914 (27.9%)	64 (31.4%)	3978 (27.9%)	48 (29.4%)	17 (30.9%)	65 (29.8%)

This Supplementary Table ummarizes the per-protocol boosted cohort, which was randomly sampled within 12 strata defined by enrollment characteristics: Assigned treatment arm × Baseline SARS-CoV-2 naive vs. non-naive status (defined by serostatus and NAAT testing) × Randomization strata (Age < 65 and at-risk, Age < 65 and not at-risk, Age ≥ 65)× Minority status (Minority vs. Non-minority) defined by White Non-Hispanic vs. all others [same as in (*2*)]. "At Risk" refers to participants believed to be at increased risk of severe COVID-19 illness and comprised six self-reported health/comorbidities, as in (*2*). "Minority" includes Blacks or African Americans, Hispanics or Latinos, American Indians or Alaska Natives, Native Hawaiians, and other Pacific Islanders. Non-Minority includes all other races with observed race (Asian, Multiracial, White, Other) and observed ethnicity Not Hispanic or Latino. Numbers and percentages are based on inverse probability of sampling weighting.

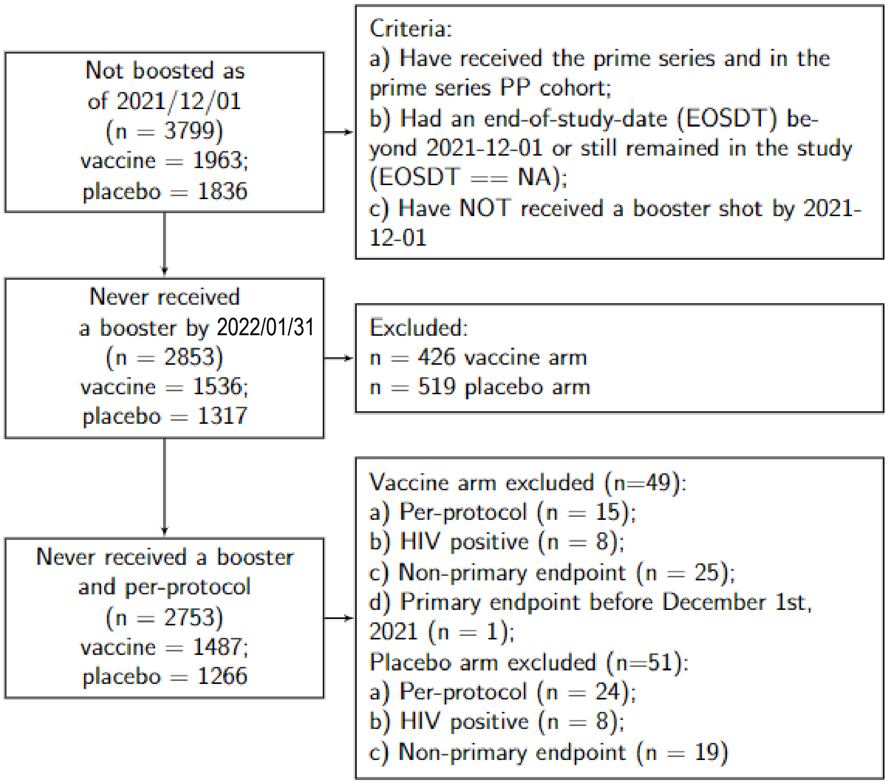

Supplementary Fig. 3. Flow of baseline-negative per-protocol (according to the definition in ref.[8]) participants who were still in the study and had not received a third (booster) dose as of December 1, 2022 through to inclusion in the exposure-proximal CoP analysis. These 2753 participants were used to enrich the analysis cohort for the exposure-proximal CoP analysis.

Supplementary Table 3. Assay limits for A) the PPD pseudovirus neutralizing antibody (nAb) assay and B) the PPD VAC123 MSD multiplex assay by antigen. Note that the Ancestral strain Spike used for pseudotyping in the nAb assay has the D614G mutation, whereas the Ancestral strain Spike used in the bAb assay does not (D614).

**A**	**Neutralizing antibody (nAb) assay**	
	Ancestral strain[1]	Omicron BA.1
LLOQ (AU/ml)	10	8
ULOQ (AU/ml)	281,600	24,503
**B**	**Binding antibody assay (Spike IgG)**	
	Ancestral strain	Omicron BA.1/B.1.1.529
LLOQ[2] (AU/ml)	69	102
ULOQ[3] (AU/ml)	14,400,000	1,180,000

[1]For ancestral strain nAbs, the units AU/ml can be transformed to International Units (IU50)/ml (see SAP).
[2]LLOQs were taken as the LLOQs for the lowest dilution (1:500).
[3]ULOQs were taken as the ULOQs for the highest dilution (1:500,000).
For all assays, values < LLOQ were set to LLOQ/2 and for the nAb assay, values > ULOQ were set to ULOQ. AU = arbitrary units.

Supplementary Table 4. BD1 Ancestral strain neutralizing antibody (nAb) and BD1 Spike IgG-Ancestral strain binding antibody (bAb) response rates and geometric means stratified by Omicron COVID-19 case vs. non-case and by SARS-CoV-2 naive vs. non-naive status in the per-protocol boosted cohort, pooled across the original-vaccine and crossover-vaccine arms

			Omicron Cases[1]			Non-Cases[2]			Comparison	
**Status[3]**	**Marker**	**Measurement**	**N[4]**	**Response Rate[5] (95% CI)**	**GMC or GMT (AU/ml)[6] (95% CI)**	**N[4]**	**Response Rate[5] (95% CI)**	**GMC or GMT (AU/ml)[6] (95% CI)**	**Response Rate Difference (Omicron Cases-Non-Cases) (95% CI)**	**Ratio of GM (Omicron Cases/Non-Cases) (95% CI)**
SARS-CoV-2 naive	Ancestral strain nAbs	BD1	79	100% (100%, 100%)	124 (89.7, 170)	84	99.7% (98.1%, 100%)	114 (75.9, 173)	0.003 (0, 0.019)	1.08 (0.64, 1.82)
SARS-CoV-2 naive	Spike IgG-Ancestral strain bAbs	BD1	79	100% (100%, 100%)	19100.77 (13777.90, 26480.05)	84	100% (100%, 100%)	18213.80 (14060.12, 23594.56)	0 (0, 0)	1.05 (0.69, 1.59)
Non-Naive	Ancestral strain nAbs	BD1	32	100% (100%, 100%)	437 (247, 770)	23	100% (100%, 100%)	148 (82.3, 264)	0 (0, 0)	2.96 (1.31, 6.68)
Non-Naive	Spike IgG-Ancestral strain bAbs	BD1	32	100% (100%, 100%)	51318 (30442, 86512)	23	100% (100%, 100%)	24521 (16224, 37062)	0 (0, 0)	2.09 (1.08, 4.07)

[1]Omicron case = COVID-19 Omicron BA.1 endpoint that occurred in the interval [≥ 7 days post BD29 AND ≥ December 1, 2021 to April 5, 2022 data cutoff].

[2]Non-case = No acquirement of COVID-19 (of any strain) in the interval [BD1, April 5, 2022 data cutoff].

[3]SARS-CoV-2 naive = No evidence of SARS-CoV-2 infection from enrollment through to BD1; Non-naive = Any evidence of SARS-CoV-2 infection in the interval [≥ 14 days after the original two-dose series, BD1]

[4]N is the number of cases sampled into the subcohort within baseline covariate strata.

[5]Definitions of "responder" for each BD1 marker: positive (quantifiable) response defined as BD1 Ancestral strain nAb ≥ 10 AU/ml; positive response defined as BD1 Spike IgG-Ancestral strain bAb ≥ 69 AU/ml.

[6]For ancestral strain nAbs, the units AU/ml can be transformed to International Units (IU50)/ml (see SAP).

AU/ml, arbitrary units/ml; CI: confidence interval; GMC: geometric mean concentration; GMT: geometric mean titer.

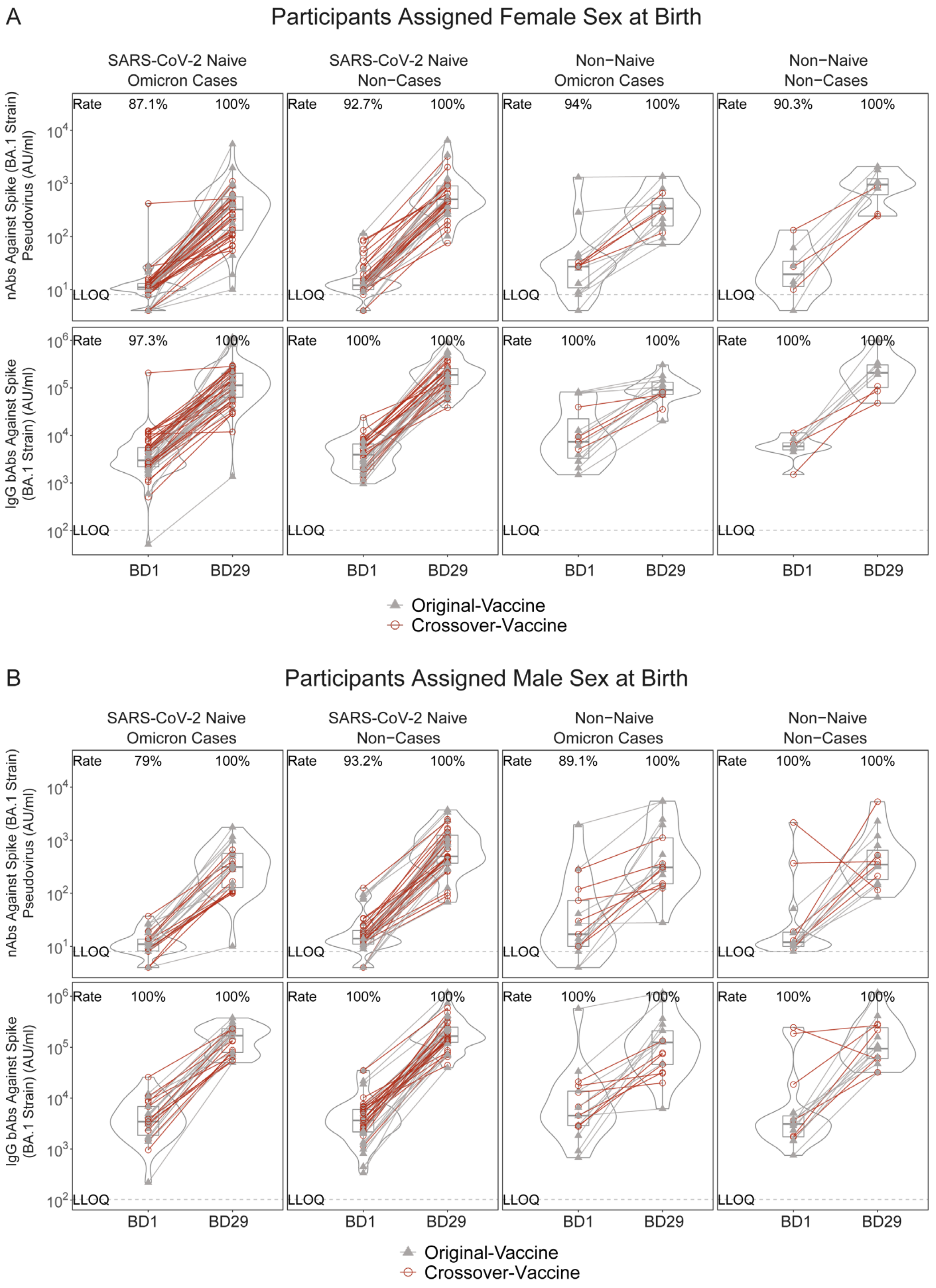

Supplementary Fig. 4. Distributions of BD1 and BD29 neutralizing antibody (nAb) titer against Spike (BA.1 strain) pseudovirus and IgG binding antibody (bAb) concentration against Spike (BA.1 strain), stratified by Omicron COVID-19 case vs. non-case status and by SARS-CoV-2 naive vs. non-naive status, shown separately in participants assigned (A)

female or (B) male sex at birth. Data points are from per-protocol boosted participants in the original-vaccine (filled triangles) or crossover-vaccine (open circles) arm, with lines (gray: original-vaccine arm; red: crossover-vaccine arm) connecting the BD1 and BD29 data points for an individual participant. Numbers of participants shown are: (A) (Female participants; all numbers are the same for both nAb BA.1 and Spike IgG BA.1): Naive Omicron cases: BD1 N=53, BD29=53; Naive non-cases: BD1 N=41, BD29 N=41; Non-naive Omicron cases: BD1 N=15, BD29 N=15; Non-naive non-case: BD1 N=8, BD29 N=8. (B) (Male participants; all numbers are the same for both nAb BA.1 and Spike IgG BA.1): Naive Omicron cases: BD1 N=26, BD29=26; Naive non-cases: BD1 N=43, BD29 N=43; Non-naive Omicron cases: BD1 N=17, BD29 N=17; Non-naive non-case: BD1 N=15, BD29 N=15. The violin plots contain interior box plots with upper and lower horizontal edges representing the 25th and 75th percentiles of antibody level and middle line representing the 50th percentile. The vertical bars represent the distance from the 25th (or 75th) percentile of antibody level and the minimum (or maximum) antibody level within the 25th (or 75th) percentile of antibody level minus (or plus) 1.5 times the interquartile range. Each side shows a rotated probability density (estimated by a kernel density estimator with a default Gaussian kernel) of the data. Positive response rates were computed with inverse probability of sampling weighting. LLOQ, lower limit of quantification. AU/ml, arbitrary units/ml. LLOQ = 8 AU/ml for nAb BA.1 and 102 AU/ml for Spike IgG BA.1. Positive (quantifiable) response for BA.1 nAb at a given timepoint was defined by value ≥ LLOQ at that timepoint. Positive response for Spike IgG-BA.1 bAb at a given timepoint was defined by value ≥ LLOQ at that timepoint. Omicron Case = COVID-19 endpoint in the interval [≥ 7 days post BD29 AND ≥ December 1, 2021 to April 5, 2022 (data cutoff date)]. Non-case = Did not acquire COVID-19 (of any strain) in the interval [BD1 to April 5, 2022]. SARS-CoV-2 naive = No evidence of SARS-CoV-2 infection from enrollment through to BD1; Non-naive = Any evidence of SARS-CoV-2 infection in the interval [≥ 14 days after the first two doses of mRNA-1273, BD1].

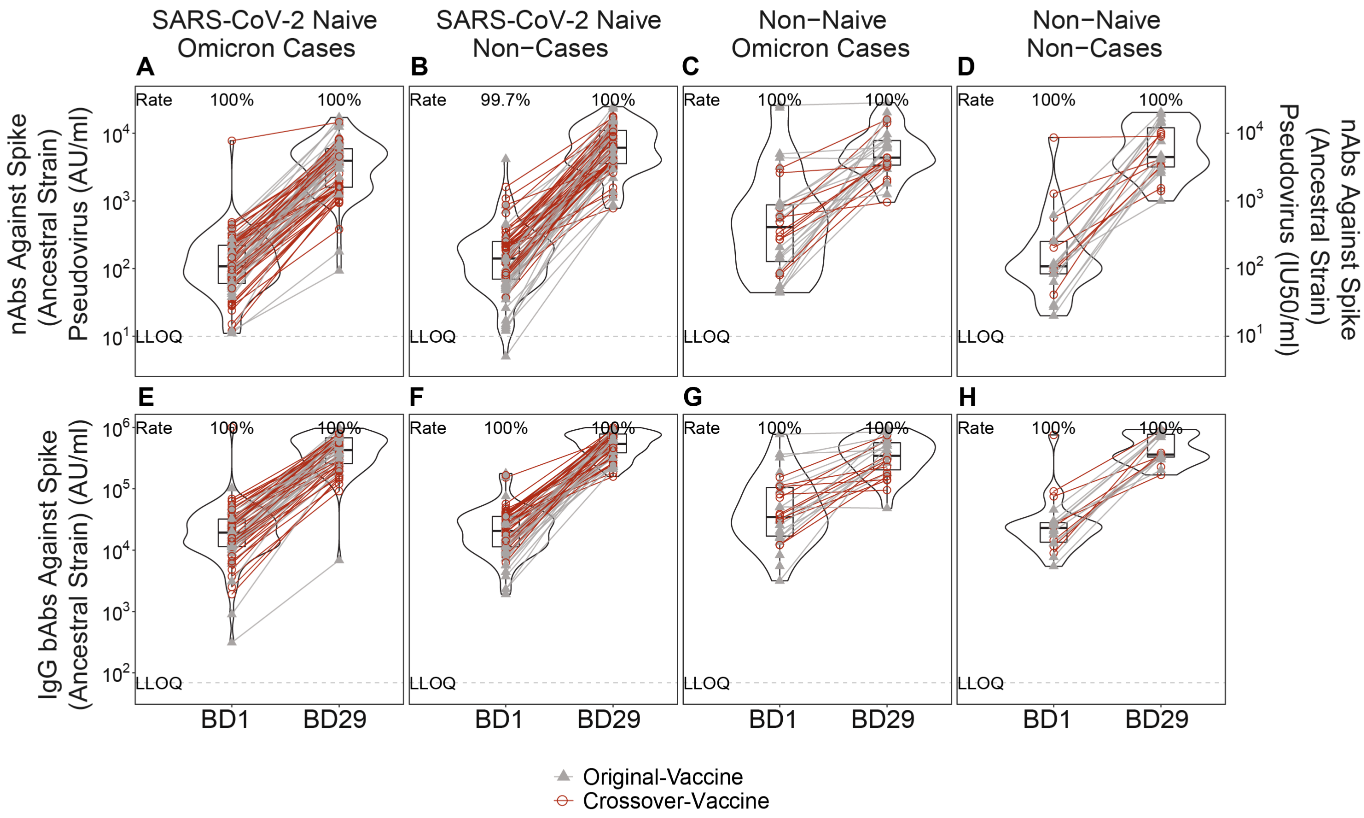

Supplementary Fig. 5. Distributions of BD1 and BD29 (A-D) Ancestral strain neutralizing antibody (nAb) titer and (E-H) Spike IgG-Ancestral strain binding antibody (bAb) concentration, stratified by Omicron COVID-19 case vs. non-case status and by SARS-CoV-2 naive vs. non-naive status. Data points are from per-protocol boosted participants in the original-vaccine (filled triangles) or crossover-vaccine (open circles) arm, with lines (gray: original-vaccine arm; red: crossover-vaccine arm) connecting the BD1 and BD29 data points for an individual participant (A, E: n=79; B, F: n=84; C, G: 32; D, H: n=23). The violin plots contain interior box plots with upper and lower horizontal edges representing the 25th and 75th percentiles of antibody level and middle line representing the 50th percentile. The vertical bars represent the distance from the 25th (or 75th) percentile of antibody level and the minimum (or maximum) antibody level within the 25th (or 75th) percentile of antibody level minus (or plus) 1.5 times the interquartile range. Each side shows a rotated probability density (estimated by a kernel density estimator with a default Gaussian kernel) of the data. Positive response rates were computed with inverse probability of sampling weighting. LLOQ, lower limit of quantification. LLOQ = 10 AU/ml for Ancestral strain nAbs and 69 AU/ml for Spike IgG-Ancestral strain bAbs. Positive response for Ancestral strain nAbs at a given timepoint was defined by value ≥ LLOQ at that timepoint. Positive response for Spike IgG-Ancestral strain bAbs at a given timepoint was defined by value ≥ LLOQ at that timepoint. Omicron Case = COVID-19 endpoint in the interval [≥ 7 days post BD29 AND ≥ December 1, 2021 to April 5, 2022 data cutoff date]. Non-case =

Did not acquire COVID-19 (of any strain) in the interval [BD1 to April 5, 2022]. SARS-CoV-2 naive = No evidence of SARS-CoV-2 infection from enrollment through to BD1; Non-naive = Any evidence of SARS-CoV-2 infection in the interval [≥ 14 days after the first two doses of mRNA-1273, BD1]. For ancestral strain nAbs, the units AU/ml can be transformed to International Units (IU50)/ml (see SAP), shown on the right-hand y-axis labels.

Supplementary Table 5. BD29 and Fold-Rise Ancestral strain neutralizing antibody (nAb) and Spike IgG-Ancestral strain binding antibody (bAb) response rates and geometric means by Omicron COVID-19 case vs. non-case status and by SARS-CoV-2 naive vs. non-naive status in the per-protocol boosted cohort, pooled across the original-vaccine and crossover-vaccine arms

			Omicron Cases[1]			Non-Cases[2]			Comparison	
Status[3]	Marker	Measurement	N[4]	Response Rate[5] (95% CI)	GMC or GMT (AU/ml)[6] (95% CI)	N[4]	Response Rate[5] (95% CI)	GMC or GMT (AU/ml) (95% CI)[6]	Response Rate Difference (Omicron Cases-Non-Cases) (95% CI)	Ratio of GM (Omicron Cases/Non-Cases) (95% CI)
SARS-CoV-2 Naive	Ancestral Strain nAbs	BD29	79	100% (100%, 100%)	3234 (2385, 4387)	84	100% (100%, 100%)	5492 (3866, 7802)	0 (0, 0)	0.59 (0.37, 0.94)
SARS-CoV-2 Naive	Spike IgG-Ancestral Strain bAbs	BD29	79	100% (100%, 100%)	467178 (364292, 599121)	84	100% (100%, 100%)	652950 (516281, 825799)	0 (0, 0)	0.72 (0.51, 1.01)
SARS-CoV-2 Naive	Ancestral Strain nAbs	Fold-Rise	79	-	26.2 (21.1, 32.4)	84	-	48.0 (37.4, 61.6)	-	0.54 (0.39, 0.76)
SARS-CoV-2 Naive	Spike IgG-Ancestral Strain bAbs	Fold-Rise	79	-	24.5 (20.0, 30.0)	84	-	35.9 (29.4, 43.7)	-	0.68 (0.51, 0.91)
Non-Naive	Ancestral Strain nAbs	BD29	32	100% (100%, 100%)	5536 (4012, 7637)	23	100% (100%, 100%)	5759 (3526, 9407)	0 (0, 0)	0.96 (0.53, 1.73)
Non-Naive	Spike IgG-Ancestral Strain bAbs	BD29	32	100% (100%, 100%)	419795 (301040, 585399)	23	100% (100%, 100%)	619269 (441303, 869004)	0 (0, 0)	0.68 (0.42, 1.09)
Non-Naive	Ancestral Strain nAbs	Fold-Rise	32	-	12.7 (7.5, 21.6)	23	-	39.0 (23.9, 63.9)	-	0.32 (0.16, 0.67)
Non-Naive	Spike IgG-Ancestral Strain bAbs	Fold-Rise	32	-	8.2 (4.7, 14.1)	23	-	25.3 (16.7, 38.2)	-	0.32 (0.16, 0.64)

[1]Omicron case = COVID-19 Omicron BA.1 endpoint that occurred in the interval [≥ 7 days post BD29 AND ≥ December 1, 2021 to April 5, 2022 data cutoff].
[2]Non-case = No acquirement of COVID-19 (of any strain) in the interval [BD1, April 5, 2022 data cutoff].
[3]SARS-CoV-2 naive = No evidence of SARS-CoV-2 infection from enrollment through to BD1; Non-naive = Any evidence of SARS-CoV-2 infection in the interval [≥ 14 days after the original two-dose series, BD1]
[4]N is the number of cases sampled into the subcohort within baseline covariate strata.
[5]Definitions of "responder" for the BD29 markers: positive (quantifiable) response defined as BD29 Ancestral strain nAbs ≥ 10 AU/ml; positive response defined as BD29 Spike IgG-Ancestral strain bAbs ≥ 69 AU/ml.
[6]For ancestral strain nAbs, the units AU/ml can be transformed to International Units (IU50)/ml (see SAP).
AU/ml, arbitrary units/ml; CI: confidence interval; GMC: geometric mean concentration; GMT: geometric mean titer.

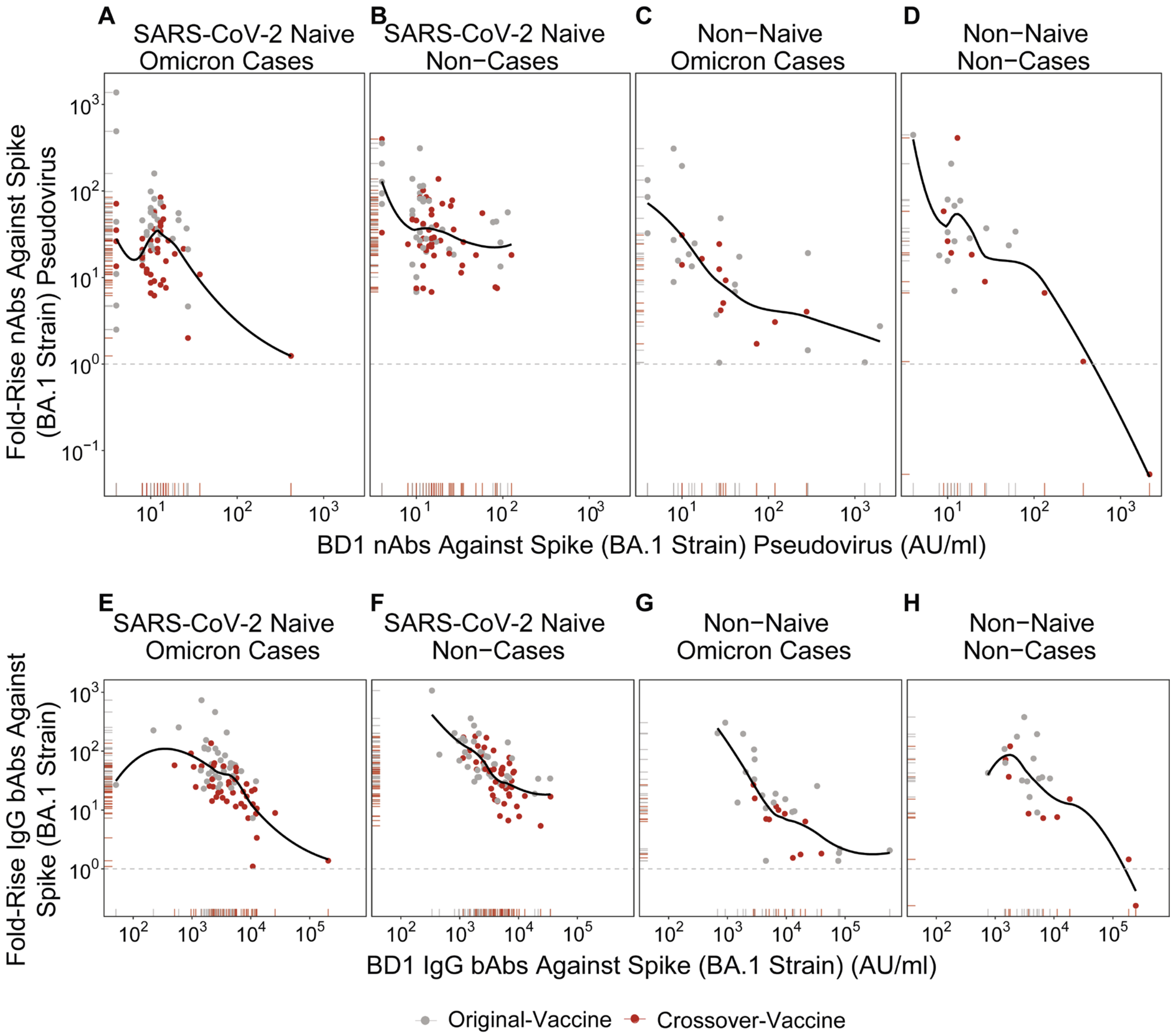

Supplementary Fig. 6. Scatterplots with rugs of BD1 and Fold-Rise (BD29/BD1) (A-D) BA.1 strain neutralizing antibody (nAb) and (E-F) Spike IgG-BA.1 strain binding antibody (bAb) level, stratified by Omicron COVID-19 case vs. non-case status and by SARS-CoV-2 naive vs. non-naive status. Data points are from per-protocol boosted participants in the original-vaccine (gray) or crossover-vaccine (red) arm (A, E: n=79; B, F: n=84; C, G: 32; D, H: n=23). The black lines are smooth curves delineating the relationship between the two variables and were fitted using the LOESS method/local regression method. Omicron Case = Omicron COVID-19 endpoint in the interval [≥ 7 days post BD29 AND ≥ December 1, 2021 to April 5, 2022 data cutoff date]. Non-case = Did not acquire COVID-19 (of any strain) in the interval [BD1 to April 5, 2022]. SARS-CoV-2 naive = No evidence of SARS-CoV-2 infection from enrollment through to BD1; Non-naive = Any evidence of SARS-CoV-2 infection in the interval [≥ 14 days after the first two doses of mRNA-1273, BD1].

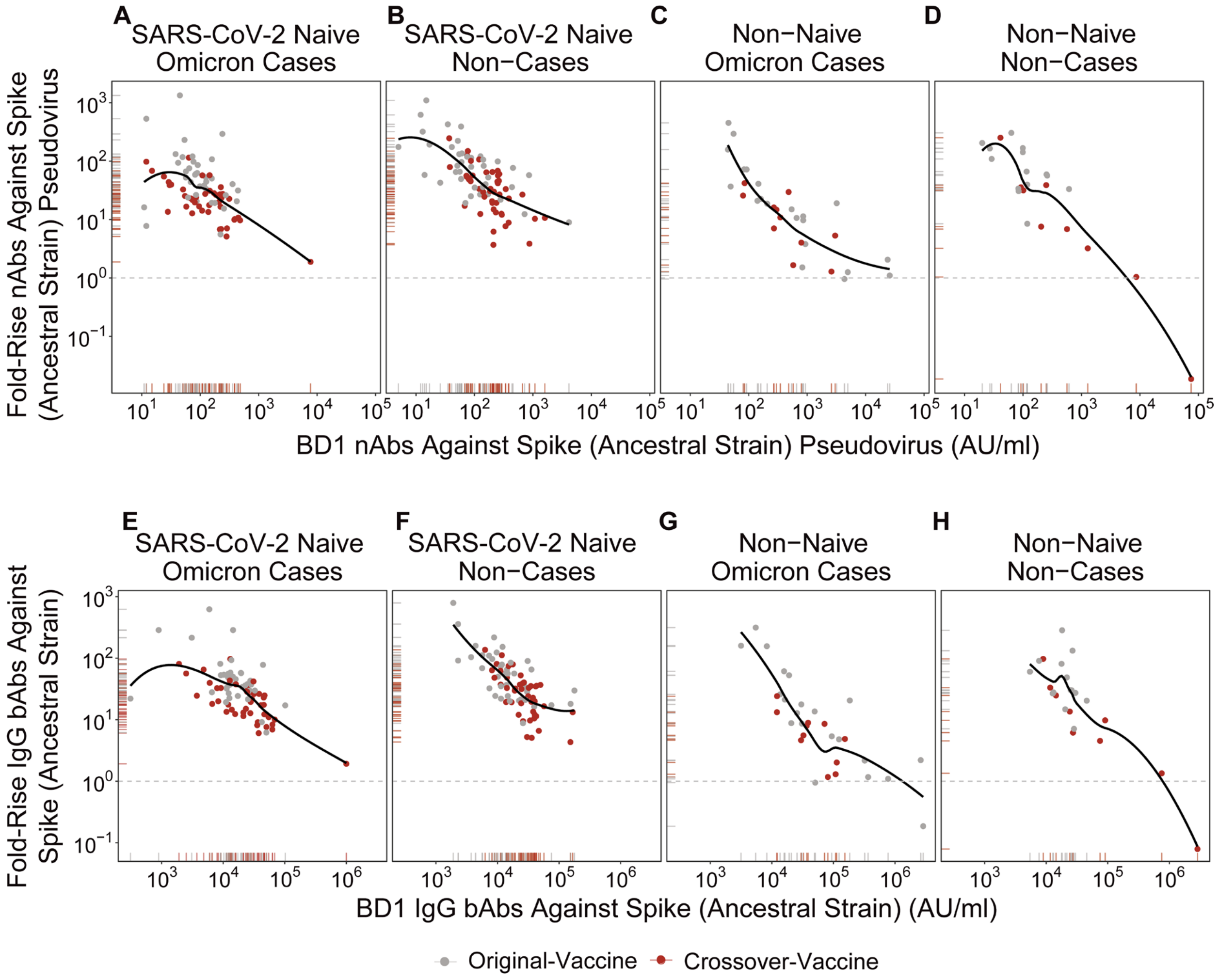

Supplementary Fig. 7. Scatterplots with rugs of BD1 and Fold-Rise (BD29/BD1) (A-D) Ancestral strain neutralizing antibody (nAb) and (E-F) Spike IgG-Ancestral strain binding antibody (bAb) level, stratified by Omicron COVID-19 case vs. non-case status and by SARS-CoV-2 naive vs. non-naive status. Data points are from per-protocol boosted participants in the original-vaccine (gray) or crossover-vaccine (red) arm (A, E: n=79; B, F: n=84; C, G: 32; D, H: n=23).. The black lines are smooth curves delineating the relationship between the two variables and were fitted using the LOESS method/local regression method. Omicron Case = Omicron COVID-19 endpoint in the interval [≥ 7 days post BD29 AND ≥ December 1, 2021 to April 5, 2022 data cutoff date]. Non-case = Did not acquire COVID-19 (of any strain) in the interval [BD1 to April 5, 2022]. SARS-CoV-2 naive (N) = No evidence of SARS-CoV-2 infection from enrollment through to BD1; Non-naive (NN) = Any evidence of SARS-CoV-2 infection in the interval [≥ 14 days after the first two doses of mRNA-1273, BD1].

Supplementary Table 6. BD29 and BD29/BD1 Fold-Rise neutralizing antibody (nAb) and binding antibody (bAb) response rates and geometric means in non-cases in the per-protocol boosted cohort, shown separately by SARS-CoV-2 naive vs. non-naive status and by study arm

Arm	Status	Marker	Visit	N	Response Rate (95% CI)	GMC or GMT (AU/ml)[1] (95% CI)
		**BA.1 Strain**				
Original-Vaccine	SARS-CoV-2 Naive	BA.1 Strain nAbs	BD29	6606	100 (100, 100)	527 (276, 1006)
Original-Vaccine	SARS-CoV-2 Naive	Spike IgG-BA.1 Strain bAbs	BD29	6606	100 (100, 100)	194468 (134566, 281036)
Original-Vaccine	SARS-CoV-2 Naive	BA.1 Strain nAbs	Fold-Rise	6606	-	37.4 (21.6, 64.8)
Original-Vaccine	SARS-CoV-2 Naive	Spike IgG-BA.1 Strain bAbs	Fold-Rise	6606	-	74.8 (58.1, 96.4)
Original-Vaccine	Non-Naive	BA.1 Strain nAbs	BD29	134	100 (100, 100)	616 (336, 1131)
Original-Vaccine	Non-Naive	Spike IgG-BA.1 Strain bAbs	BD29	134	100 (100, 100)	165136 (98711, 276261)
Original-Vaccine	Non-Naive	BA.1 Strain nAbs	Fold-Rise	134	-	40.3 (26.5, 61.4)
Original-Vaccine	Non-Naive	Spike IgG-BA.1 Strain bAbs	Fold-Rise	134	-	46.5 (32.2, 67.3)
Crossover-Vaccine	SARS-CoV-2 Naive	BA.1 Strain nAbs	BD29	6164	100 (100, 100)	455 (329, 629)
Crossover-Vaccine	SARS-CoV-2 naive	Spike IgG-BA.1 Strain bAbs	BD29	6164	100 (100, 100)	148498 (117406, 187823)
Crossover-Vaccine	SARS-CoV-2 Naive	BA.1 Strain nAbs	Fold-Rise	6164	-	30.1 (21.8, 41.6)
Crossover-Vaccine	SARS-CoV-2 Naive	Spike IgG-BA.1 Strain bAbs	Fold-Rise	6164	-	33.7 (24.7, 46.0)
Crossover-Vaccine	Non-Naive	BA.1 Strain nAbs	BD29	35	100 (100, 100)	432 (212, 879)
Crossover-Vaccine	Non-Naive	Spike IgG-BA.1 Strain bAbs	BD29	35	100 (100, 100)	98349 (58932, 164131)
Crossover-Vaccine	Non-Naive	BA.1 Strain nAbs	Fold-Rise	35	-	9.7 (1.9, 49.4)
Crossover-Vaccine	Non-Naive	Spike IgG-BA.1 Strain bAbs	Fold-Rise	35	-	9.8 (2.8, 33.9)
		**Ancestral strain**				
Original-Vaccine	SARS-CoV-2 Naive	Ancestral Strain nAbs	BD29	6606	100 (100, 100)	5859 (3101, 11067)
Original-Vaccine	SARS-CoV-2 Naive	Spike IgG-Ancestral Strain bAbs	BD29	6606	100 (100, 100)	715670 (471776, 1085650)
Original-Vaccine	SARS-CoV-2 Naive	Ancestral Strain nAbs	Fold-Rise	6606	-	77.6 (52.4, 115)

Original-Vaccine	SARS-CoV-2 Naive	Spike IgG-Ancestral Strain bAbs	Fold-Rise	6606	-	53.1 (38.8, 72.8)
Original-Vaccine	Non-Naive	Ancestral Strain nAbs	BD29	134	100 (100, 100)	6297 (3433, 11550)
Original-Vaccine	Non-Naive	Spike IgG-Ancestral Strain bAbs	BD29	134	100 (100, 100)	682910 (452891, 1029752)
Original-Vaccine	Non-Naive	Ancestral Strain nAbs	Fold-Rise	134	-	61.4 (41.6, 90.8)
Original-Vaccine	Non-Naive	Spike IgG-Ancestral Strain bAbs	Fold-Rise	134	-	35.6 (24.7, 51.2)
Crossover-Vaccine	SARS-CoV-2 Naive	Ancestral Strain nAbs	BD29	6164	100 (100, 100)	5125 (3859, 6806)
Crossover-Vaccine	SARS-CoV-2 Naive	Spike IgG-Ancestral Strain bAbs	BD29	6164	100 (100, 100)	591821 (474520, 738120)
Crossover-Vaccine	SARS-CoV-2 Naive	Ancestral Strain nAbs	Fold-Rise	6164	-	28.7 (20.3, 40.6)
Crossover-Vaccine	SARS-CoV-2 Naive	Spike IgG-Ancestral Strain bAbs	Fold-Rise	6164	-	23.5 (17.7, 31.3)
Crossover-Vaccine	Non-Naive	Ancestral Strain nAbs	BD29	35	100 (100, 100)	4092 (2534, 6608)
Crossover-Vaccine	Non-Naive	Spike IgG-Ancestral Strain bAbs	BD29	35	100 (100, 100)	425821 (283019, 640676)
Crossover-Vaccine	Non-Naive	Ancestral Strain nAbs	Fold-Rise	35	-	6.9 (1.2, 40.3)
Crossover-Vaccine	Non-Naive	Spike IgG-Ancestral Strain bAbs	Fold-Rise	35	-	6.8 (1.8, 25.9)

Fold-Rise = BD29/BD1. N is the number of cases sampled into the subcohort within baseline covariate strata. Non-case = No acquirements of COVID-19 (of any strain) in the interval [BD1, data cutoff date]. SARS-CoV-2 naive = No evidence of SARS-CoV-2 infection from enrollment through to BD1. Non-naive = Any evidence of SARS-CoV-2 infection in the interval [≥ 14 days after the original 2-dose series, BD1]

[1]For ancestral strain nAbs, the units AU/ml can be transformed to International Units (IU50)/ml (see SAP).

GMC: geometric mean concentration; GMT: geometric mean titer.

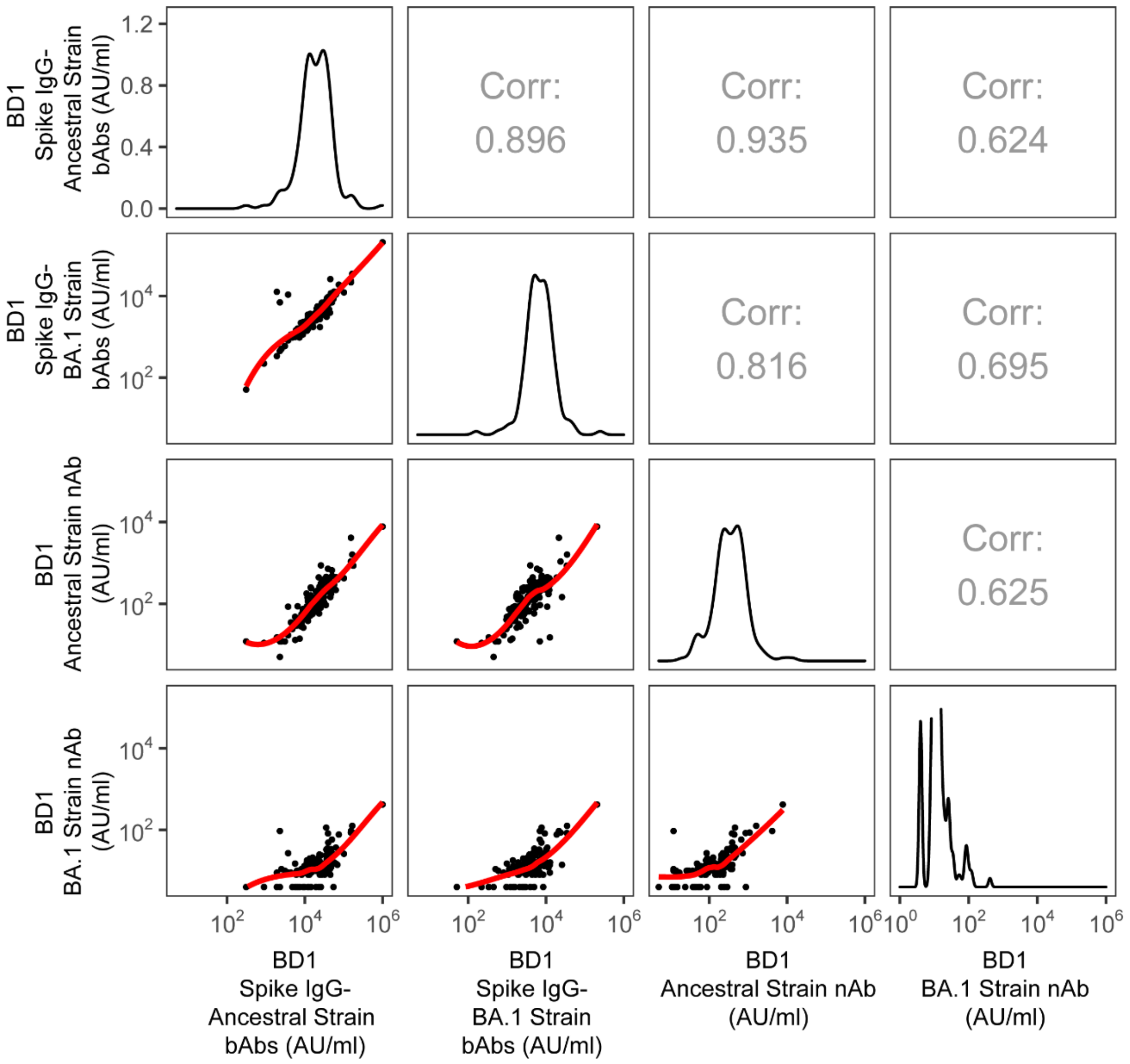

Supplementary Fig. 8. Correlations of BD1 antibody markers among SARS-CoV-2 naive participants in the per-protocol boosted cohort (n = 218). Corr = Inverse probability weight adjusted Spearman's rank correlation coefficient. P < 0.001 for all pairwise correlations. For ancestral strain nAbs, the units AU/ml can be transformed to International Units (IU50)/ml (see SAP).

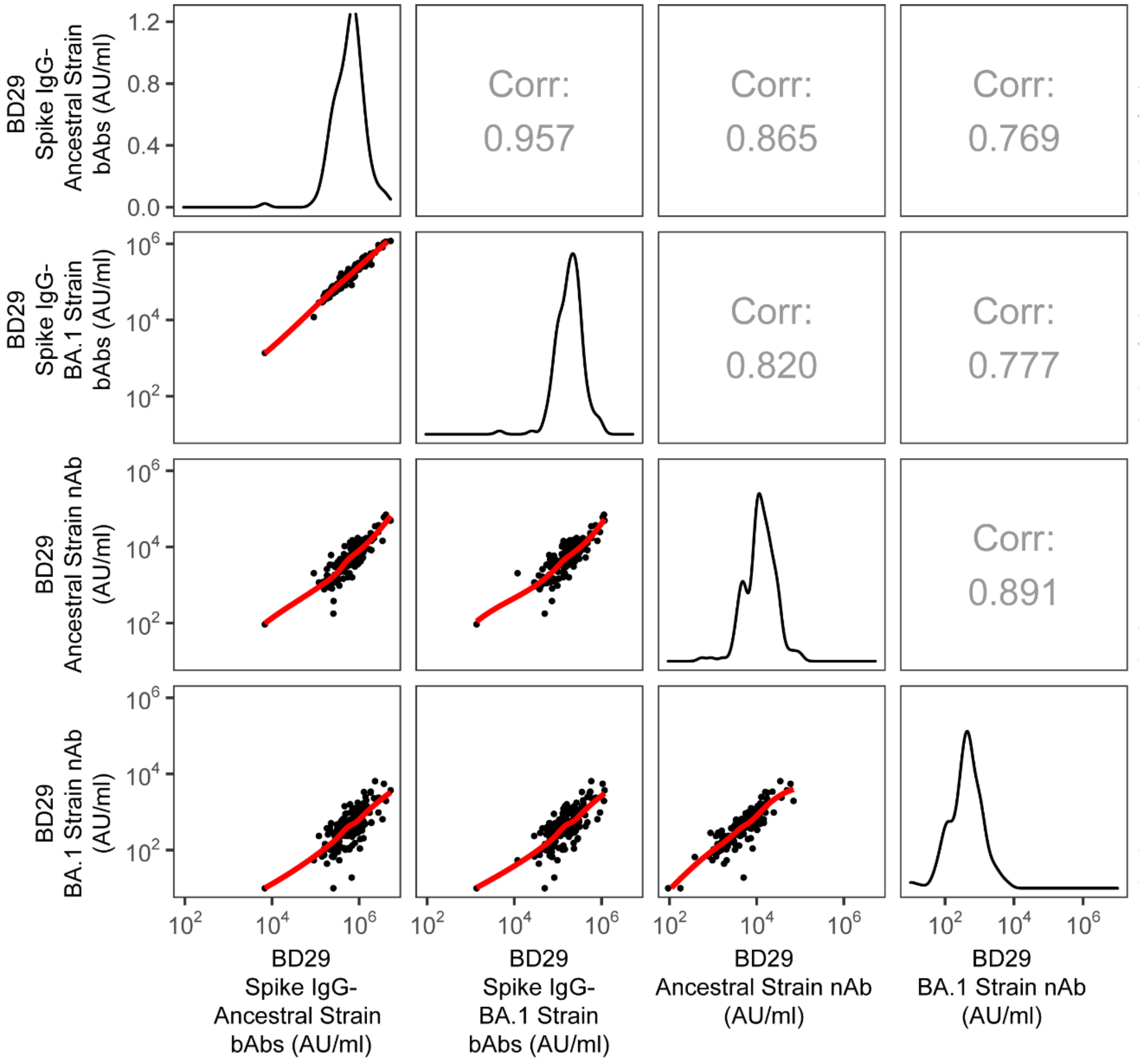

Supplementary Fig. 9. Correlations of BD29 antibody markers among SARS-CoV-2 naive participants in the per-protocol boosted cohort (n = 218). Corr = Inverse probability weight adjusted Spearman's rank correlation coefficient. P < 0.001 for all pairwise correlations. For ancestral strain nAbs, the units AU/ml can be transformed to International Units (IU50)/ml (see SAP).

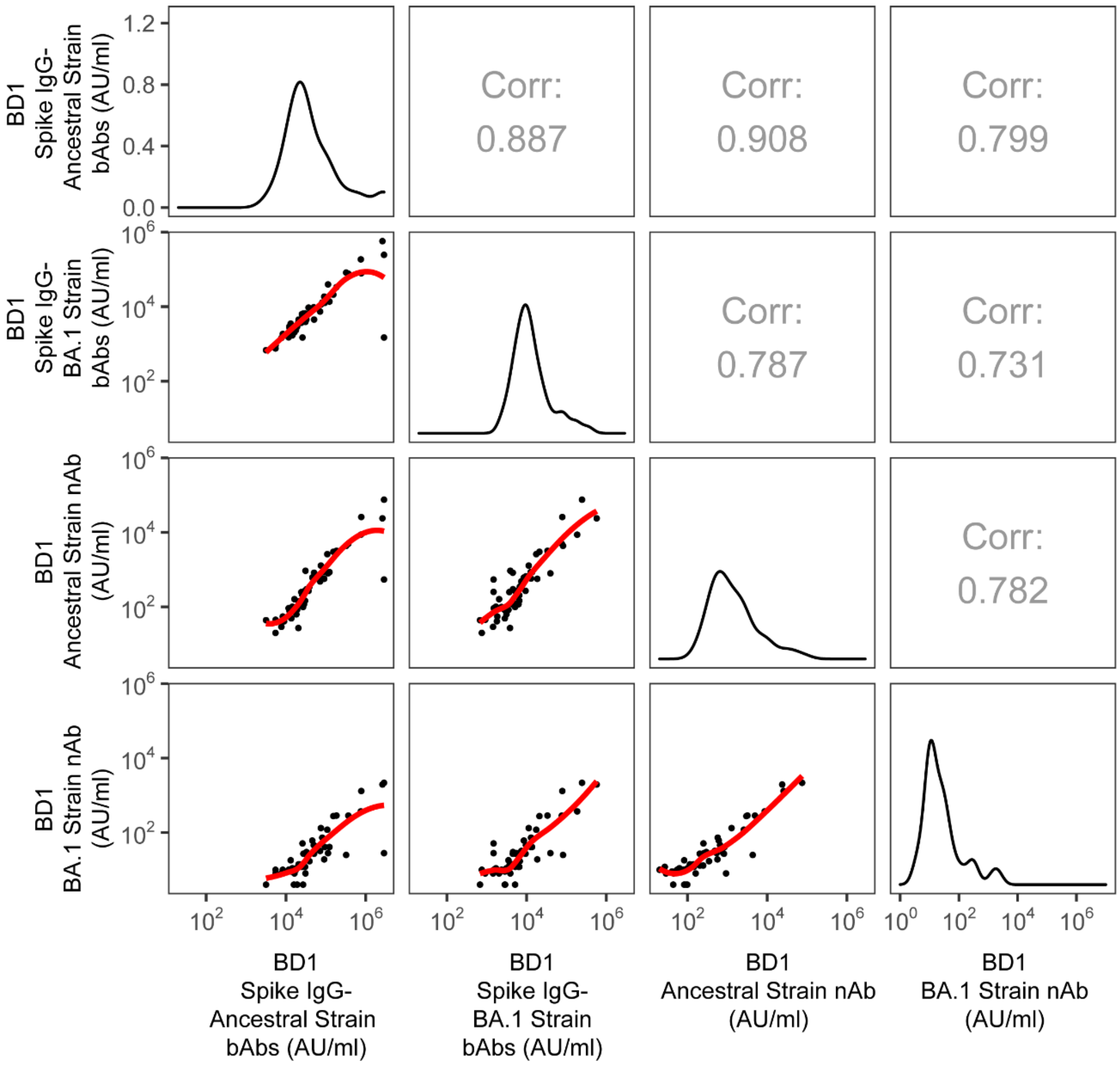

Supplementary Fig. 10. Correlations of BD1 antibody markers among non-naive participants in the per-protocol boosted cohort (n = 55). Corr = Inverse probability weight adjusted Spearman's rank correlation coefficient. P < 0.001 for all pairwise correlations. For ancestral strain nAbs, the units AU/ml can be transformed to International Units (IU50)/ml (see SAP).

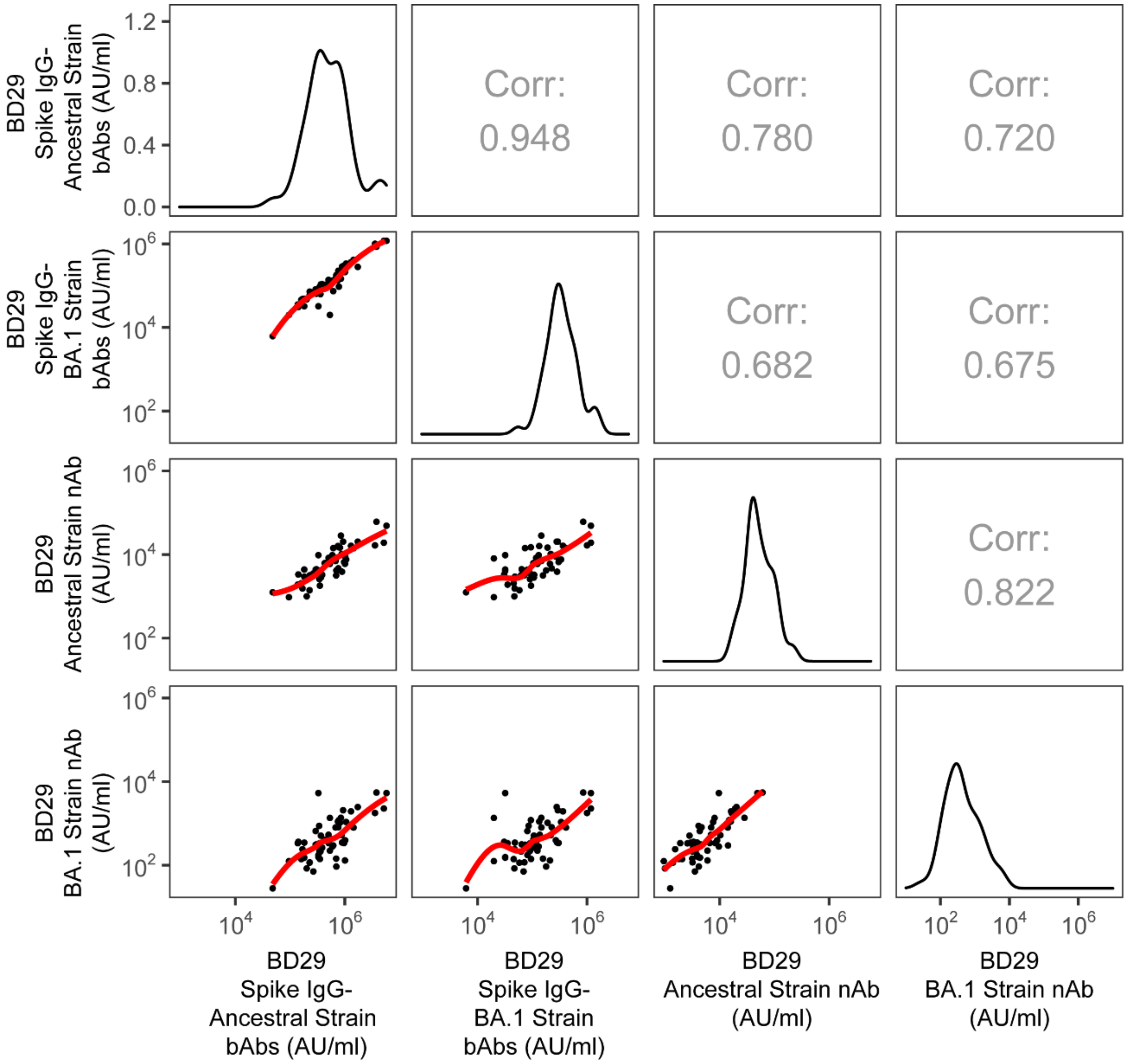

Supplementary Fig. 11. Correlations of BD29 antibody markers among non-naive participants in the per-protocol boosted cohort (n = 55). Corr = Inverse probability weight adjusted Spearman's rank correlation coefficient. $P < 0.001$ for all pairwise correlations.

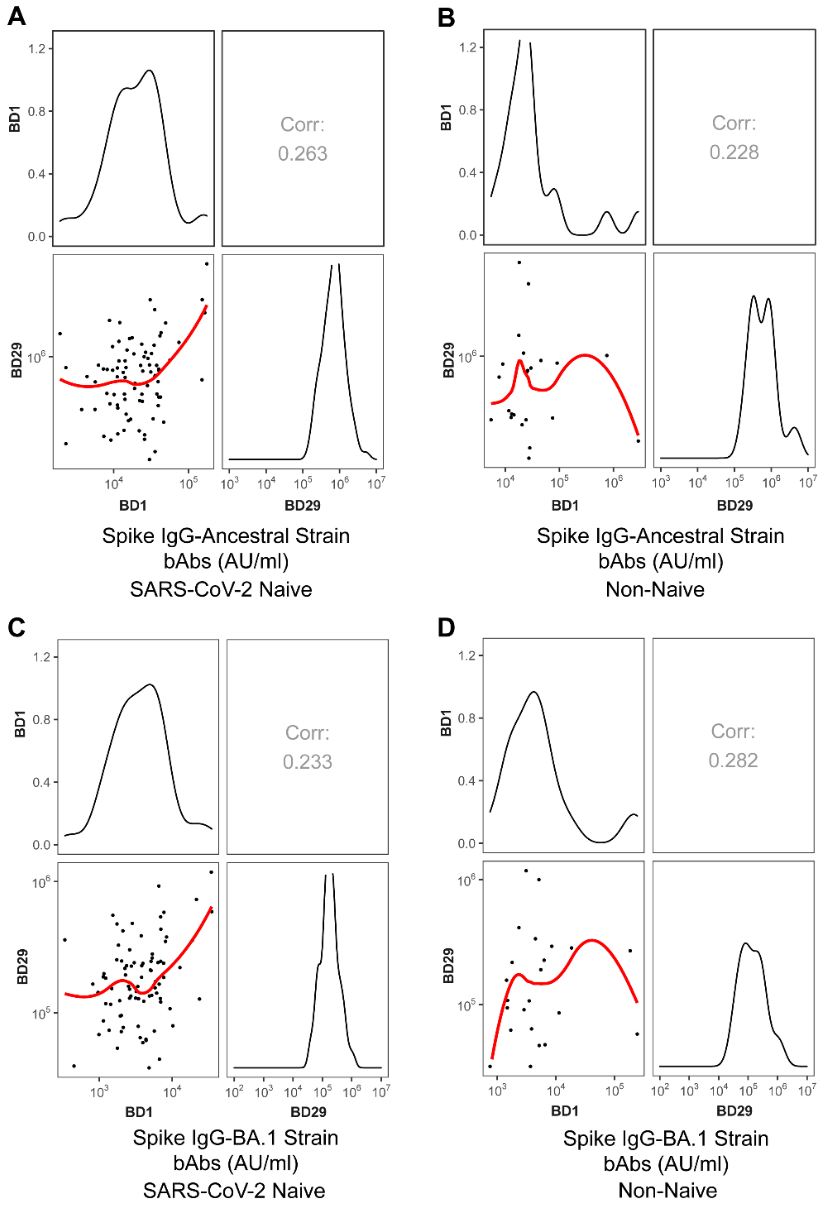

Supplementary Fig. 12. Correlations between BD1 and BD29 (A, B) Spike IgG-Ancestral strain binding antibody (bAb) and (C, D) Spike IgG-BA.1 strain bAb concentrations among (A, C) SARS-CoV-2 naive participants and (B, D) non-naive participants in the per-protocol boosted cohort (A, C: n=84; B, D: n =23). Corr = Inverse probability weight adjusted Spearman's rank correlation coefficient. $P < 0.001$ for the Ancestral strain among naive participants; $P = 0.85$ for the Ancestral strain among non-naive participants; $P = 0.002$ for the BA.1 strain among naive participants; $P = 0.34$ for the BA.1 strain among non-naive participants.

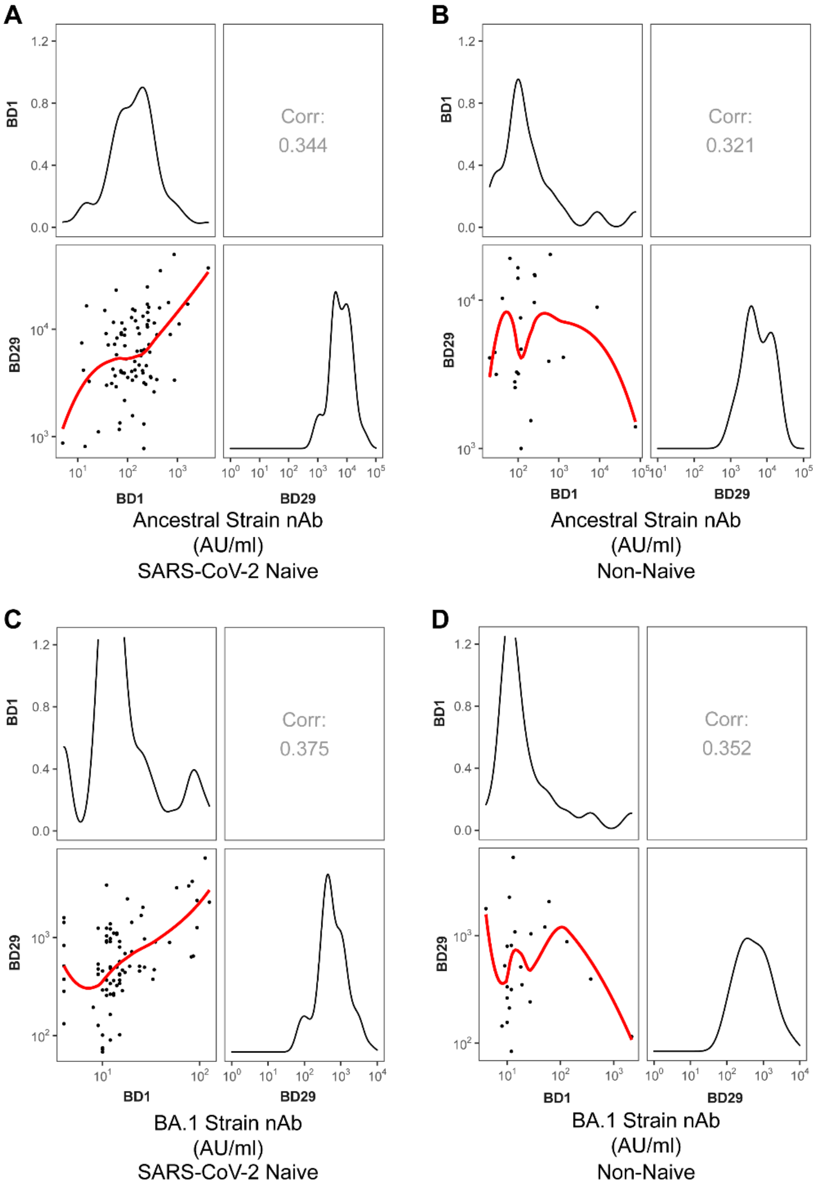

Supplementary Fig. 13. Correlations between BD1 and BD29 (A, B) Ancestral strain neutralizing antibody (nAb) and (C, D) BA.1 strain nAb titers among (A, C) SARS-CoV-2 naive participants and (B, D) non-naive participants in the per-protocol boosted cohort (A, C: n=84; B, D: n =23). Corr = Inverse probability weight adjusted Spearman's rank correlation coefficient. P < 0.001 for the Ancestral strain among naive participants; P = 0.61 for the Ancestral strain among non-naive participants; P < 0.001 for the BA.1 strain among naive participants; P = 0.80 for the BA.1 strain among non-naive participants. For ancestral strain nAbs, the units AU/ml can be transformed to International Units (IU50)/ml (see SAP).

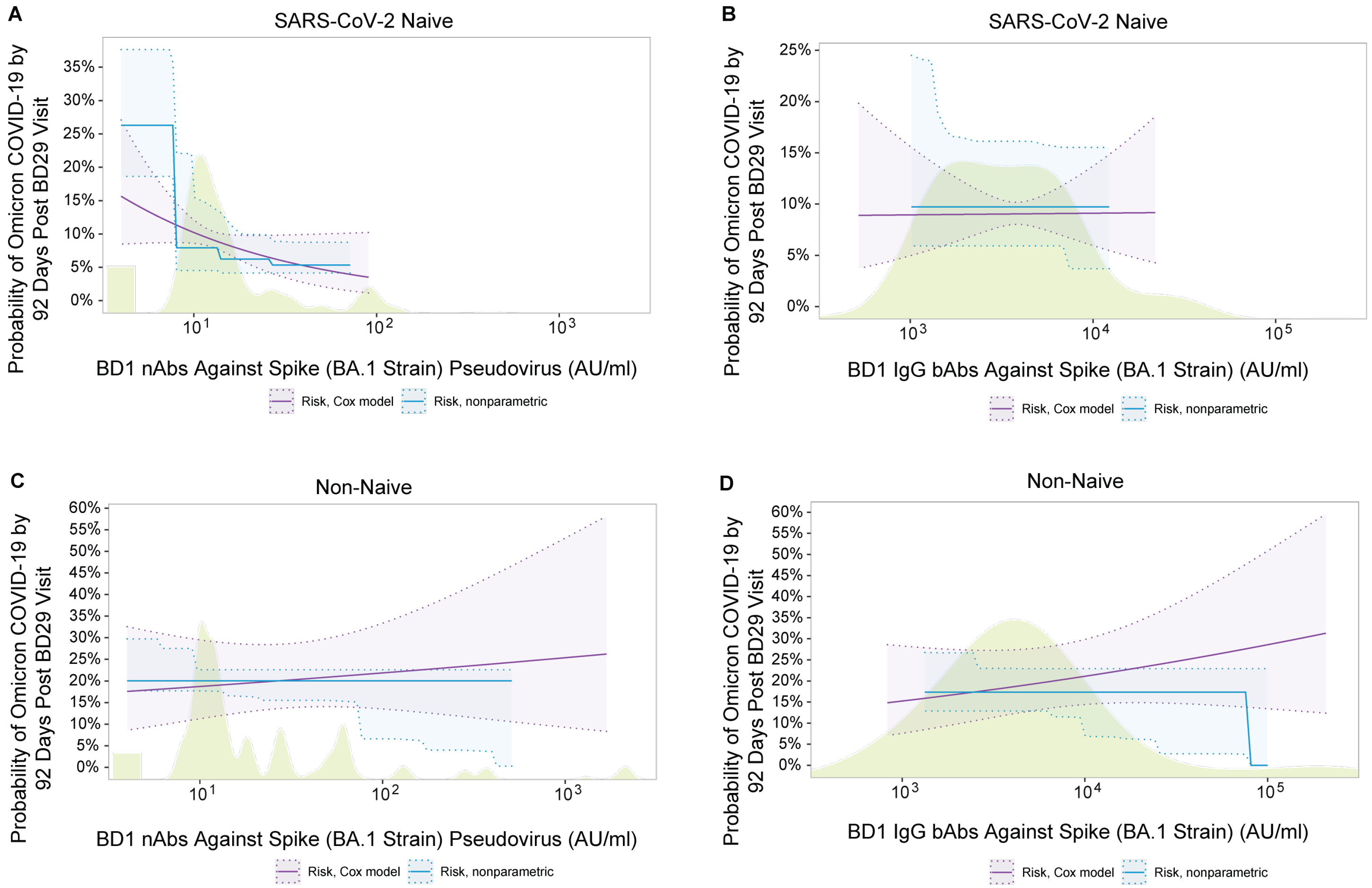

E

	Status	Marker	No. Cases / No. At-Risk*	HR per 10-Fold Increase Pt. Est. (95% CI)	P-value (2-sided)	FDR-Adj. P-value**	FWER-Adj. P-value**
BD1	SARS-CoV-2 Naive	BA.1 Strain nAb	1,277/14,047	0.31 (0.07, 1.37)	0.123	0.736	0.479
BD1	SARS-CoV-2 Naive	Spike IgG-BA.1 Strain bAb	1,277/14,047	1.02 (0.29, 3.56)	0.977	0.988	1.000
BD1	Non-Naive	BA.1 Strain nAb	35/204	1.21 (0.56, 2.61)	0.627	0.721	0.886
BD1	Non-Naive	Spike IgG-BA.1 Strain bAb	35/204	1.47 (0.71, 3.08)	0.302	0.588	0.747

* No. at-risk = estimated number in the population for analysis, i.e. per-protocol boosted participants (naive or non-naive, as designated) not experiencing the Omicron COVID-19 endpoint or SARS-CoV-2 infected through 6 days post BD29 visit; no. cases = number of this cohort with an observed Omicron COVID-19 endpoint.

** FDR (false discovery rate)-adjusted p values and FWER (family-wise error rate)-adjusted p-values were computed over the set of p-values both for quantitative markers and categorical markers using the Westfall and Young permutation method (10000 replicates).

Supplementary Fig. 14. Analyses of BD1 BA.1 strain neutralizing antibody (nAb) titer and Spike IgG-BA.1 strain binding antibody (bAb) concentration as a correlate of risk of Omicron COVID-19. Curves show cumulative incidence of Omicron COVID-19, estimated using a Cox model (purple) or a nonparametric method (blue), in per-protocol boosted (A, B) SARS-CoV-2 naive participants (N = 14,047) and (C, D) non-naive participants (N = 204) by 92 days post BD29 by BD1 antibody marker level. The solid curves indicate the mean cumulative incidences. The dotted lines and shadings in between indicate bootstrap pointwise 95% CIs. The distribution of the marker in the respective analysis population, calculated by kernel density estimation, is plotted in light green. E) Hazard ratios of Omicron COVID-19 per 10-fold increase in each BD1 BA.1 strain marker in per-protocol boosted SARS-CoV-2 naive participants or non-naive participants. Baseline covariates adjusted for: baseline risk score, at risk status, and community of color status.

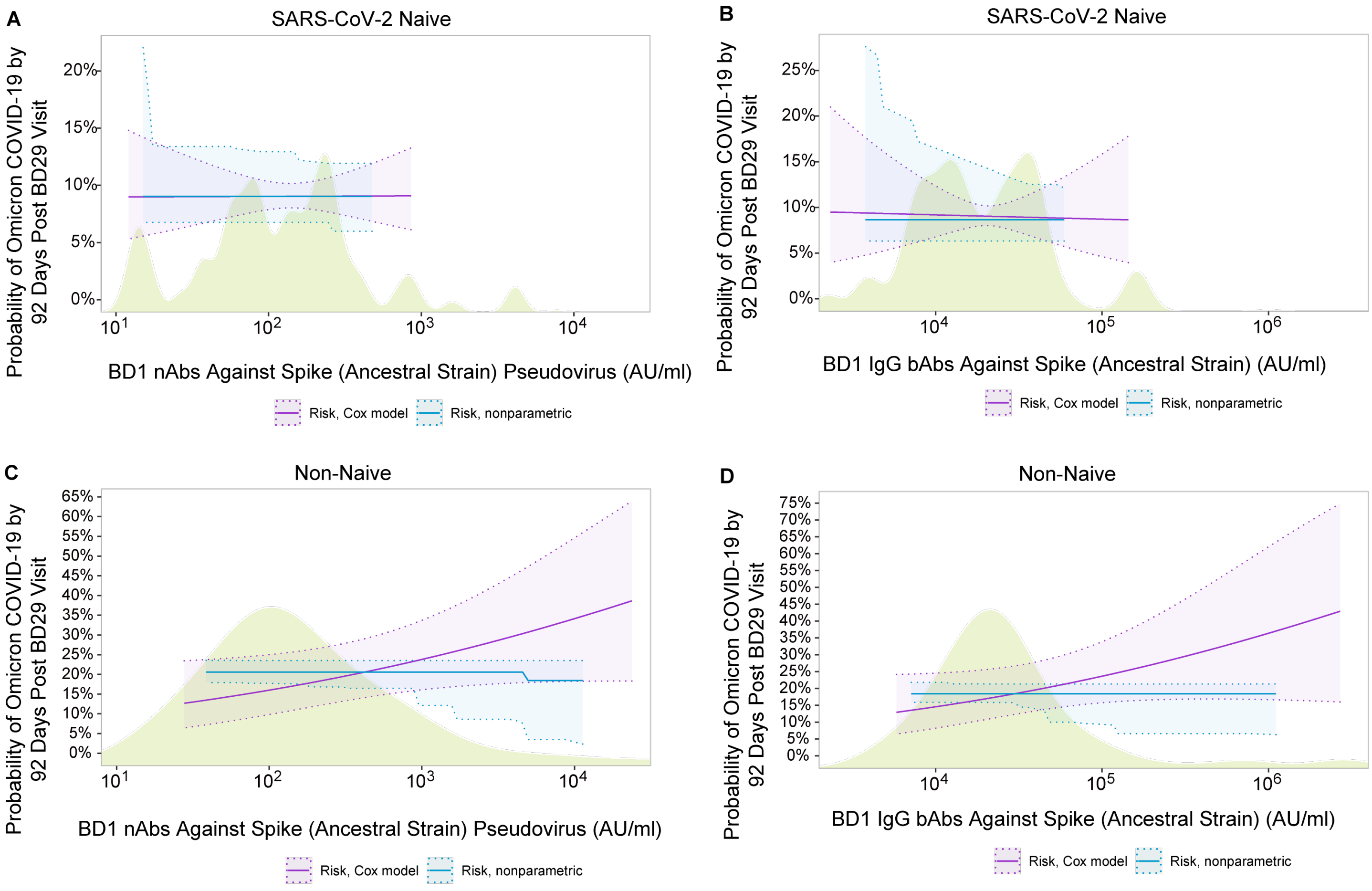

E

	Status	Marker	No. Cases / No. At-Risk*	HR per 10-Fold Increase Pt. Est. (95% CI)	P-value (2-sided)	FDR-Adj. P-value**	FWER-Adj. P-value**
BD1	SARS-CoV-2 Naive	Ancestral Strain nAb	1,277/14,047	1.01 (0.47, 2.15)	0.988	0.988	1.000
BD1	SARS-CoV-2 Naive	Spike IgG-Ancestral Strain bAb	1,277/14,047	0.95 (0.30, 2.98)	0.924	0.988	1.000
BD1	Non-Naive	Ancestral Strain nAb	35/204	1.61 (0.99, 2.63)	0.057	0.408	0.398
BD1	Non-Naive	Spike IgG-Ancestral Strain bAb	35/204	1.78 (0.90, 3.51)	0.096	0.433	0.486

* No. at-risk = estimated number in the population for analysis, i.e. per-protocol boosted participants (naive or non-naive, as designated) not experiencing the Omicron COVID-19 endpoint or SARS-CoV-2 infected through 6 days post BD29 visit; no. cases = number of this cohort with an observed Omicron COVID-19 endpoint.

** FDR (false discovery rate)-adjusted p values and FWER (family-wise error rate)-adjusted p-values were computed over the set of p-values both for quantitative markers and categorical markers using the Westfall and Young permutation method (10000 replicates).

Supplementary Fig. 15. Analyses of BD1 Ancestral strain neutralizing antibody (nAb) titer and Spike IgG-Ancestral strain binding antibody (bAb) concentration as a correlate of risk of Omicron COVID-19. Curves show cumulative incidence of Omicron COVID-19, estimated using a Cox model (purple) or a nonparametric method (blue), in per-protocol boosted (A, B) SARS-CoV-2 naive participants (N = 14,047) and (C, D) non-naive participants (N = 204) by 92 days post BD29 by BD1 antibody marker level. The solid curves indicate the mean cumulative incidences. The dotted lines and shadings in between indicate bootstrap pointwise 95% CIs. The distribution of the marker in the respective analysis population, calculated by kernel density estimation, is plotted in light green. E) Hazard ratios of Omicron COVID-19 per 10-fold increase in each BD1 Ancestral strain marker in per-protocol boosted SARS-CoV-2 naive participants or non-naive participants. Baseline covariates adjusted for: baseline risk score, at risk status, and community of color status. For ancestral strain nAbs, the units AU/ml can be transformed to International Units (IU50)/ml (see SAP).

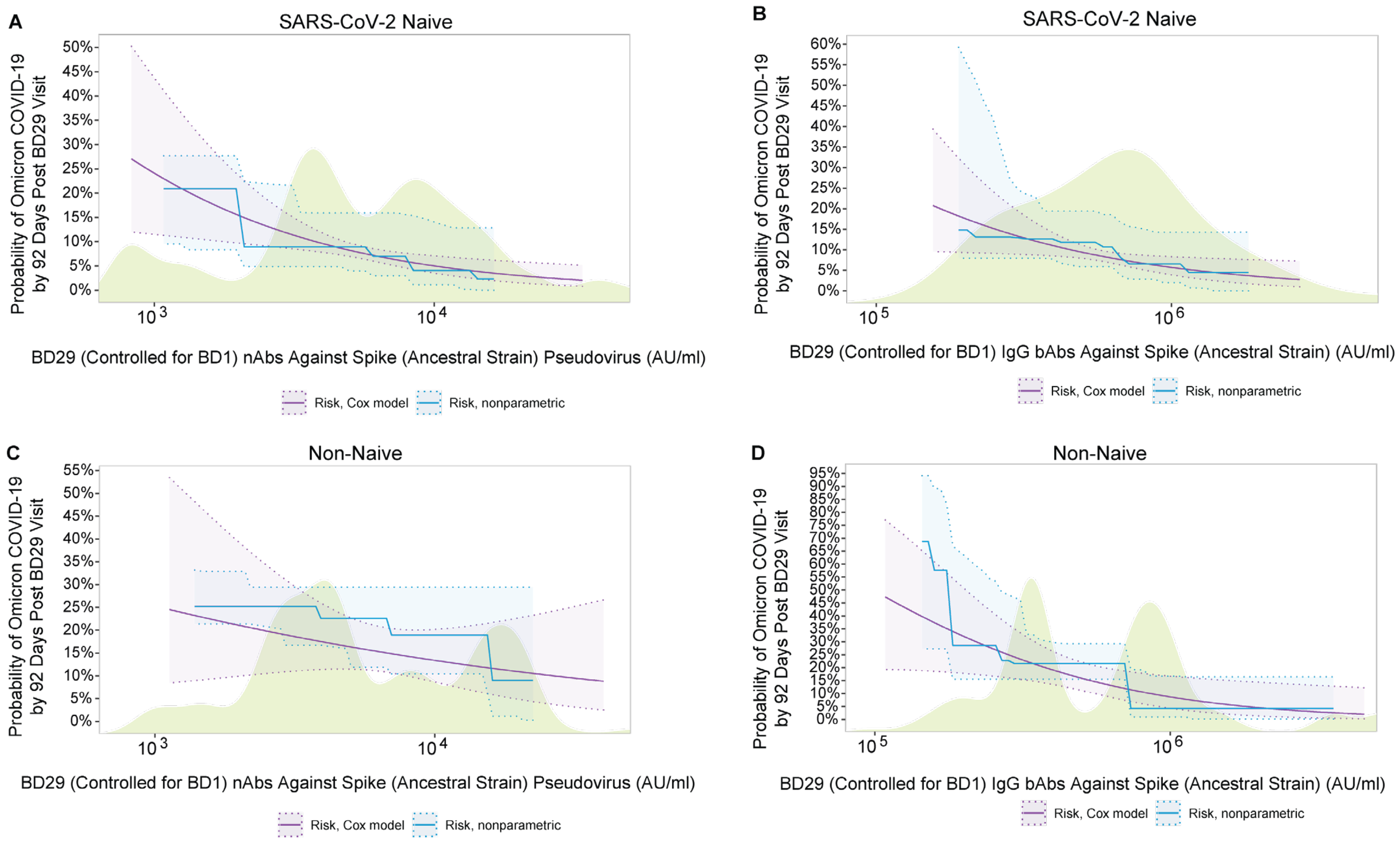

E

	Status	Marker	No. Cases / No. At-Risk*	HR per 10-Fold Increase Pt. Est. (95% CI)	P-value (2-sided)	FDR-Adj. P-value**	FWER-Adj. P-value **
BD29	SARS-CoV-2 Naive	Ancestral Strain nAb	1,277/14,047	0.33 (0.10, 1.13)	0.076	0.122	0.195
BD29	SARS-CoV-2 Naive	Spike IgG-Ancestral Strain bAb	1,277/14,047	0.23 (0.04, 1.25)	0.089	0.125	0.195
BD29	Non-Naive	Ancestral Strain nAb	35/204	0.45 (0.07, 2.95)	0.409	0.513	0.638
BD29	Non-Naive	Spike IgG-Ancestral Strain bAb	35/204	0.10 (0.01, 0.68)	0.019	0.289	0.211

* No. at-risk = estimated number in the population for analysis, i.e. per-protocol boosted participants (naive or non-naive, as designated) not experiencing the Omicron COVID-19 endpoint or SARS-CoV-2 infected through 6 days post BD29 visit; no. cases = number of this cohort with an observed Omicron COVID-19 endpoint.

** FDR (false discovery rate)-adjusted p values and FWER (family-wise error rate)-adjusted p-values were computed over the set of p-values both for quantitative markers and categorical markers using the Westfall and Young permutation method (10000 replicates).

Supplementary Fig. 16. Analyses of BD29 Ancestral strain neutralizing antibody (nAb) titer and Spike IgG-Ancestral strain binding antibody (bAb) concentration as a correlate of risk of Omicron COVID-19. Curves show cumulative incidence of Omicron COVID-19, estimated using a Cox model (purple) or a nonparametric method (blue), in per-protocol boosted (A, B) SARS-CoV-2 naive participants (N = 14,047) and (C, D) non-naive participants (N = 204) by 92 days post BD29 by BD29 antibody marker level. BD29 marker levels were controlled for BD1 marker levels. The solid curves indicate the mean cumulative incidences. The dotted lines and shadings in between indicate bootstrap pointwise 95% CIs. The distribution of the marker in the respective analysis population, calculated by kernel density estimation, is plotted in light green. E) Hazard ratios of Omicron COVID-19 per 10-fold increase in each BD29 Ancestral marker in per-protocol boosted SARS-CoV-2 naive participants or non-naive participants. Baseline covariates adjusted for: baseline risk score, at risk status, and community of color status. For ancestral strain nAbs, the units AU/ml can be transformed to International Units (IU50)/ml (see SAP).

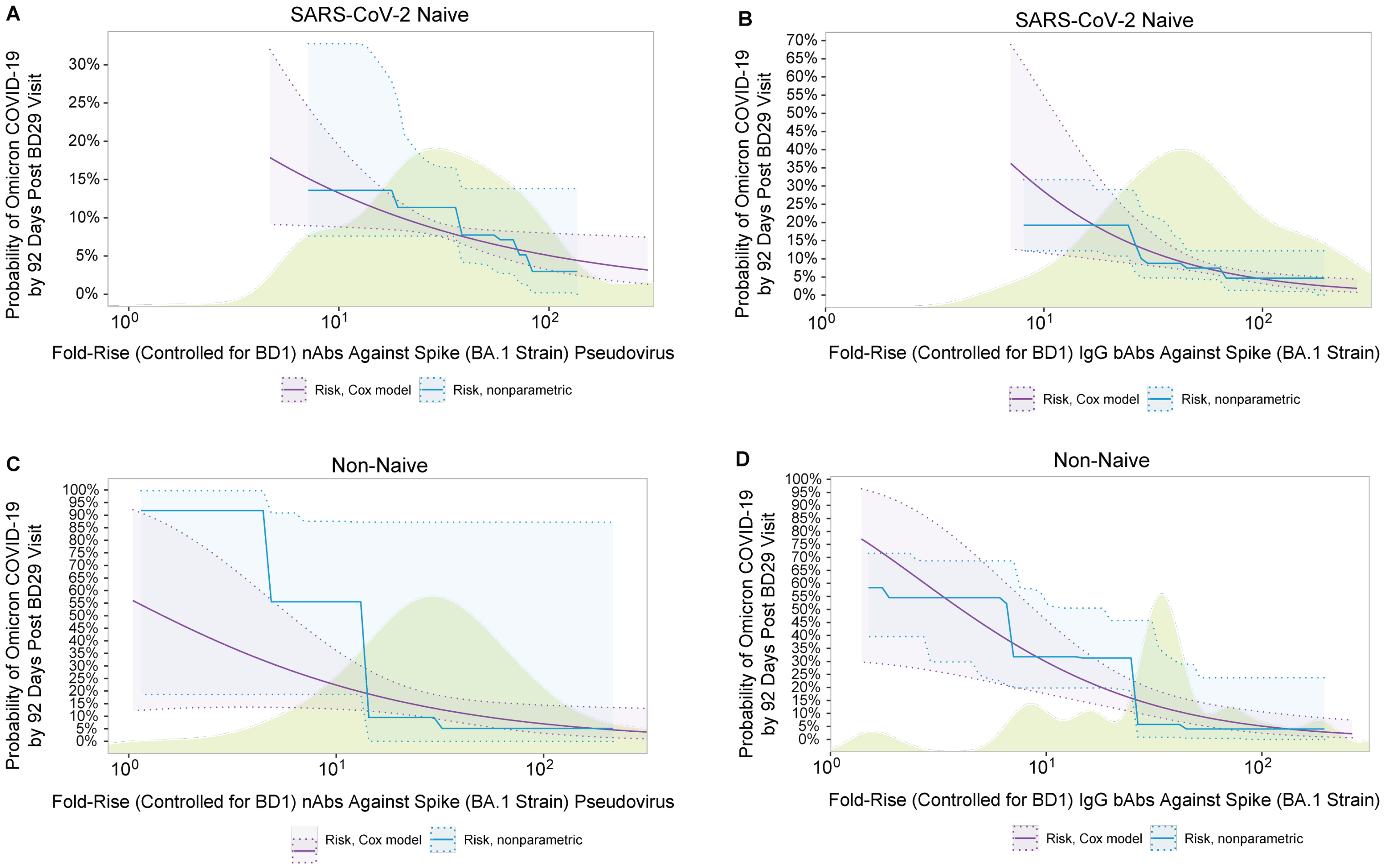

Supplementary Fig. 17. Analyses of fold-rise (BD29/BD1) BA.1 strain neutralizing antibody (nAb) titer and Spike IgG-BA.1 strain binding antibody (bAb) concentration as a correlate of risk of Omicron COVID-19. Curves show cumulative incidence of Omicron COVID-19, estimated using a Cox model (purple) or a nonparametric method (blue), in per-protocol boosted (A, B) SARS-CoV-2 naive participants (N = 14,047) and (C, D) non-naive participants (N = 204) by 92 days post BD29 by BD29/BD29 antibody marker level. BD29 marker levels were controlled for BD1 marker levels. The solid curves indicate the mean cumulative incidences. The dotted lines and shadings in between indicate bootstrap pointwise 95% CIs. The distribution of the marker in the respective analysis population, calculated by kernel density estimation, is plotted in light green. Baseline covariates adjusted for: baseline risk score, at risk status, and community of color status.

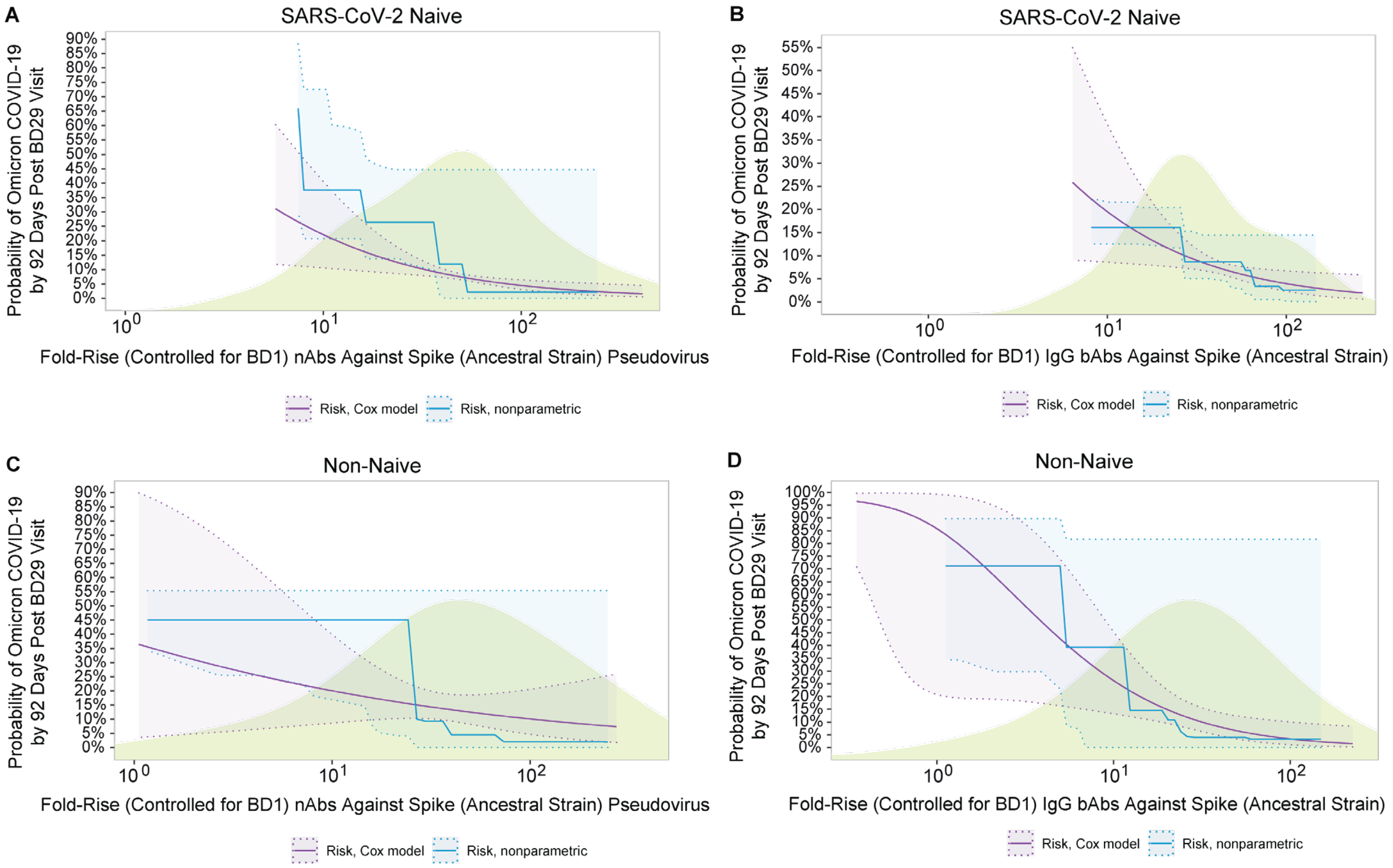

E

	Status	Marker	No. Cases / No. At-Risk*	HR per 10-Fold Increase Pt. Est. (95% CI)	P-value (2-sided)	FDR-Adj. P-value**	FWER-Adj. P-value**
Fold-Rise	SARS-CoV-2 Naive	Ancestral Strain nAb	1,277/14,047	0.38 (0.17, 0.83)	0.015	0.064	0.084
Fold-Rise	SARS-CoV-2 Naive	Spike IgG-Ancestral Strain bAb	1,277/14,047	0.41 (0.16, 1.08)	0.073	0.170	0.256
Fold-Rise	Non-Naive	Ancestral Strain nAb	35/204	0.61 (0.39, 0.95)	0.031	0.123	0.233
Fold-Rise	Non-Naive	Spike IgG-Ancestral Strain bAb	35/204	0.46 (0.25, 0.84)	0.012	0.123	0.166

* No. at-risk = estimated number in the population for analysis, i.e. per-protocol boosted participants (naive or non-naive, as designated) not experiencing the Omicron COVID-19 endpoint or SARS-CoV-2 infected through 6 days post BD29 visit; no. cases = number of this cohort with an observed Omicron COVID-19 endpoint.

** FDR (false discovery rate)-adjusted p values and FWER (family-wise error rate)-adjusted p-values were computed over the set of p-values both for quantitative markers and categorical markers using the Westfall and Young permutation method (10000 replicates).

Supplementary Fig. 18. Analyses of fold-rise (BD29/BD1) Ancestral strain neutralizing antibody (nAb) titer and Spike IgG-Ancestral strain binding antibody (bAb) concentration as a correlate of risk of Omicron COVID-19. Curves show cumulative incidence of Omicron COVID-19, estimated using a Cox model (purple) or a nonparametric method (blue), in per-protocol boosted (A, B) SARS-CoV-2 naive participants (N = 14,047) and (C, D) non-naive participants (N = 204) by 92 days post BD29 by BD29/BD1 fold-rise. BD29 marker levels were controlled for BD1 marker levels. The solid curves indicate the mean cumulative incidences. The dotted lines and shadings in between indicate bootstrap pointwise 95% CIs. The distribution of the marker in the respective analysis population, calculated by kernel density estimation, is plotted in light green. E) Hazard ratios of Omicron COVID-19 per 10-fold increase in each fold-rise (BD29/BD1) Ancestral marker in per-protocol boosted SARS-CoV-2 naive participants or non-naive participants. Baseline covariates adjusted for: baseline risk score, at risk status, and community of color status.

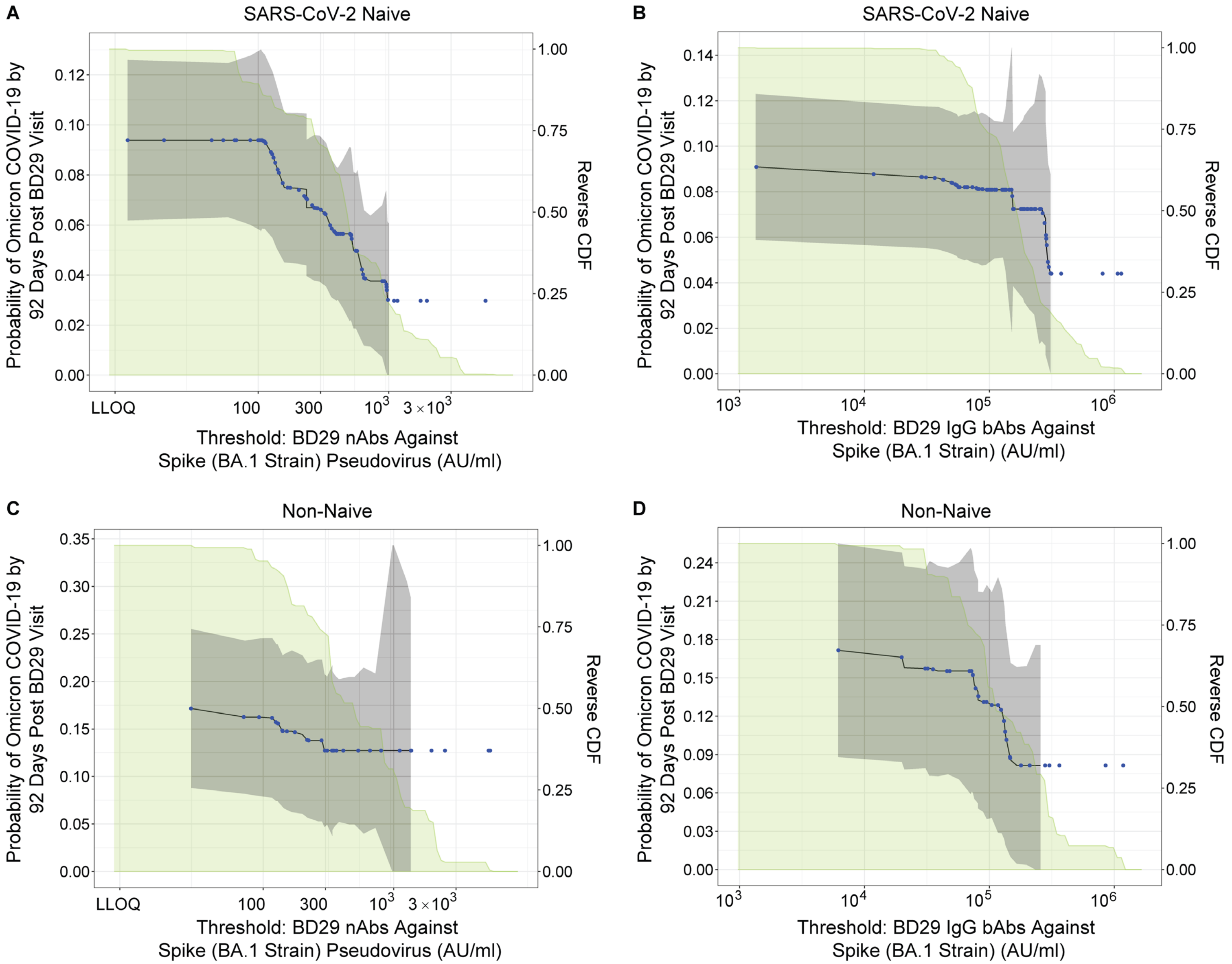

Supplementary Fig. 19. Cumulative incidence of Omicron COVID-19 by 92 days post BD29 by per-protocol boosted subgroups of (A, B) SARS-CoV-2 naive participants (N = 14,047) and (C, D) non-naive participants (N = 204) defined by (A, C) BD29 BA.1 strain neutralizing antibody (nAb) titer or (B, D) BD29 Spike IgG-BA.1 strain binding antibody (bAb) concentration above a threshold. The reverse cumulative distribution function (CDF) of each antibody marker is overlaid in green. Estimates and confidence intervals were adjusted using the assumption that the true threshold-response is nonincreasing. The blue dots correspond to marker values where an event is observed. The gray shaded area is pointwise 95% CIs.

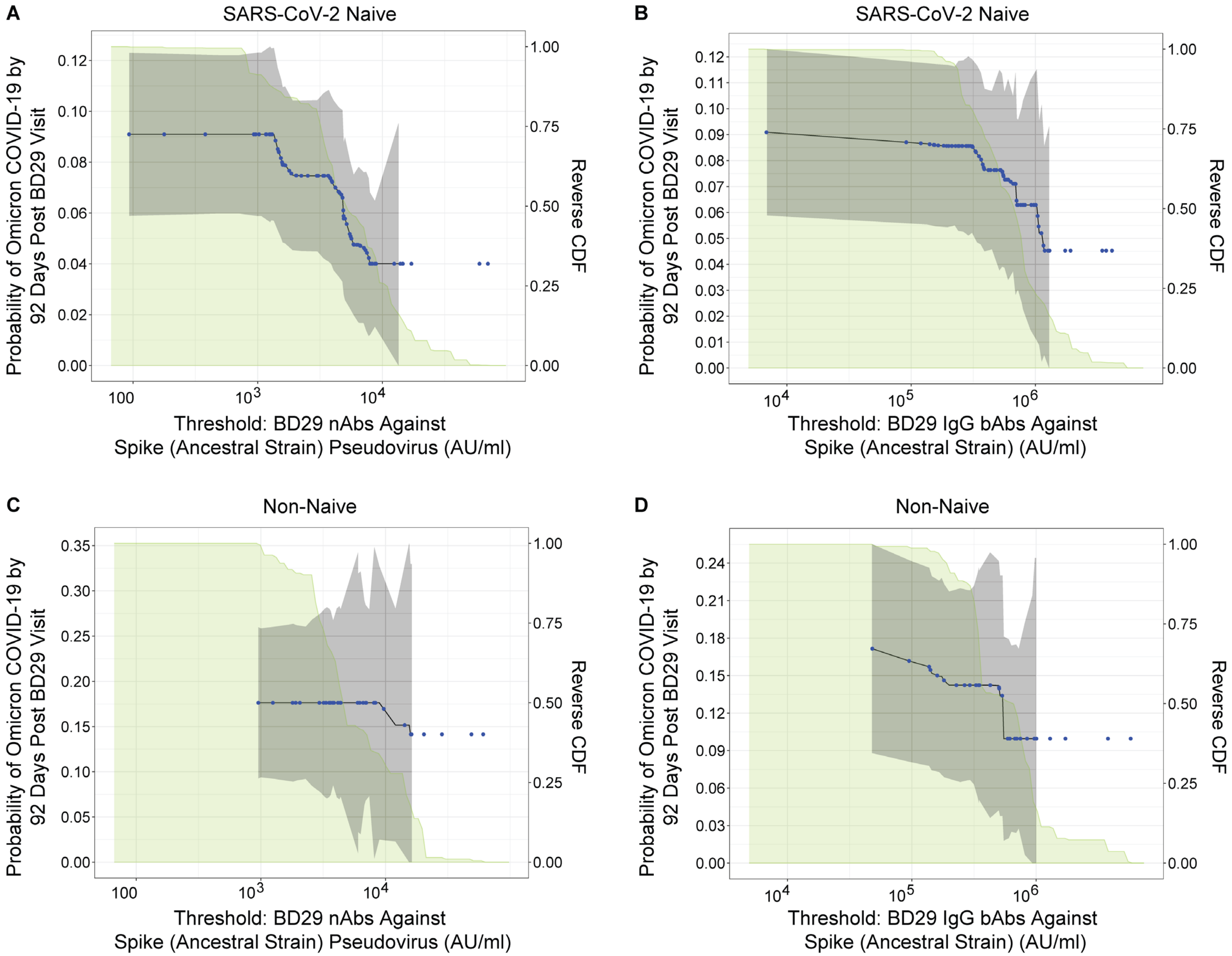

Supplementary Fig. 20. Cumulative incidence of Omicron COVID-19 by 92 days post BD29 by per-protocol boosted subgroups of (A, B) SARS-CoV-2 naive participants (N = 14,047) and (C, D) non-naive participants (N = 204) defined by (A, C) BD29 Ancestral strain neutralizing antibody (nAb) titer or (B, D) BD29 Spike IgG-Ancestral strain binding antibody (bAb) concentration above a threshold. The reverse cumulative distribution function (CDF) of each antibody marker is overlaid in green. Estimates and confidence intervals were adjusted using the assumption that the true threshold-response is nonincreasing. The blue dots correspond to marker values where an event is observed. The gray shaded area is pointwise 95% CIs. For ancestral strain nAbs, the units AU/ml can be transformed to International Units (IU50)/ml (see SAP).

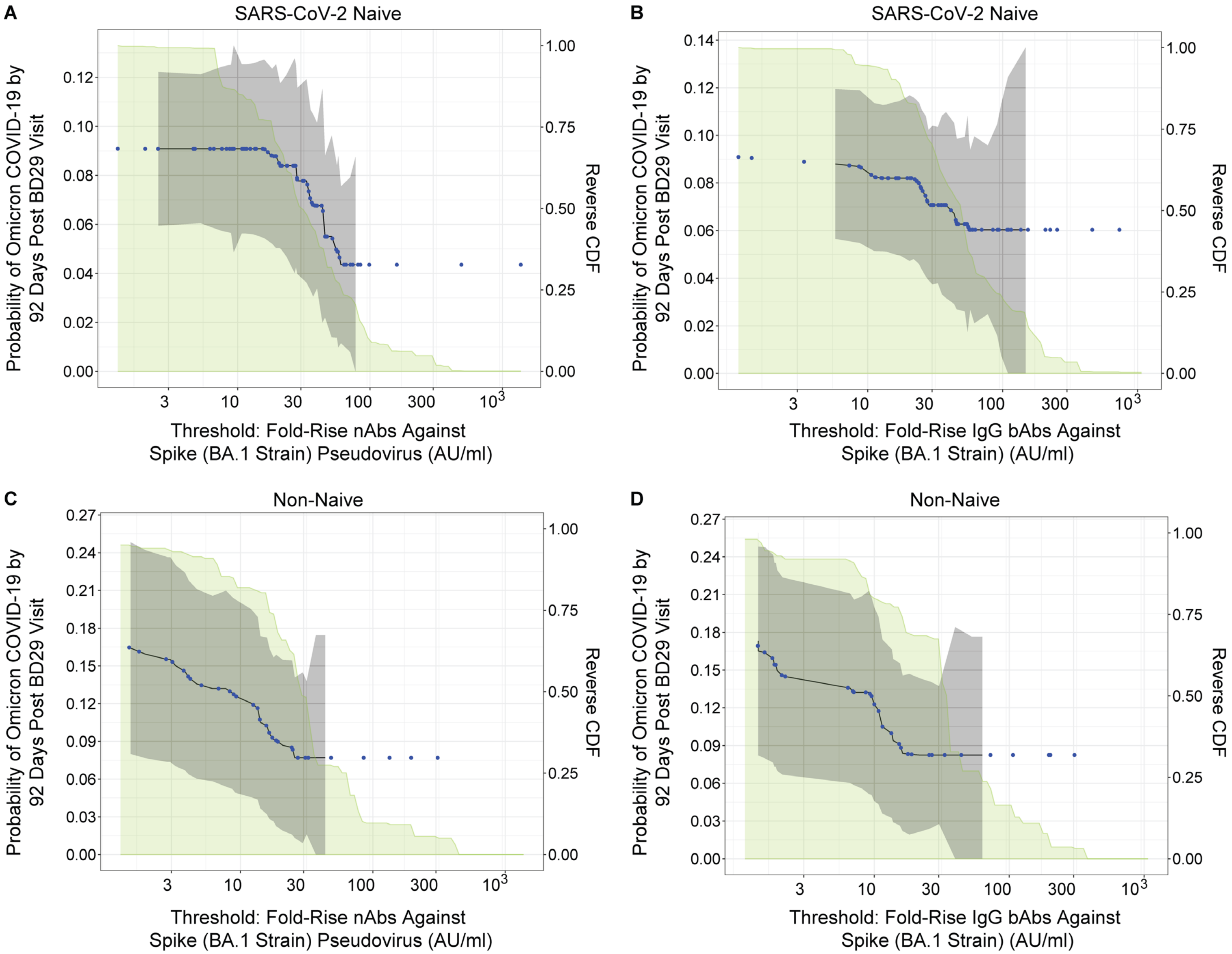

Supplementary Fig. 21. Cumulative incidence of Omicron COVID-19 by 92 days post BD29 by per-protocol boosted subgroups of (A, B) SARS-CoV-2 naive participants (N = 14,047) and (C, D) non-naive participants (N = 204) defined by (A, C) Fold-rise (BD29/BD1) BA.1 strain neutralizing antibody (nAb) titer or (B, D) Fold-rise (BD29/BD1) Spike IgG-BA.1 strain binding antibody (bAb) concentration above a threshold. The reverse cumulative distribution function (CDF) of each antibody marker is overlaid in green. Estimates and confidence intervals were adjusted using the assumption that the true threshold-response is nonincreasing. The blue dots correspond to marker values where an event is observed. The gray shaded area is pointwise 95% CIs.

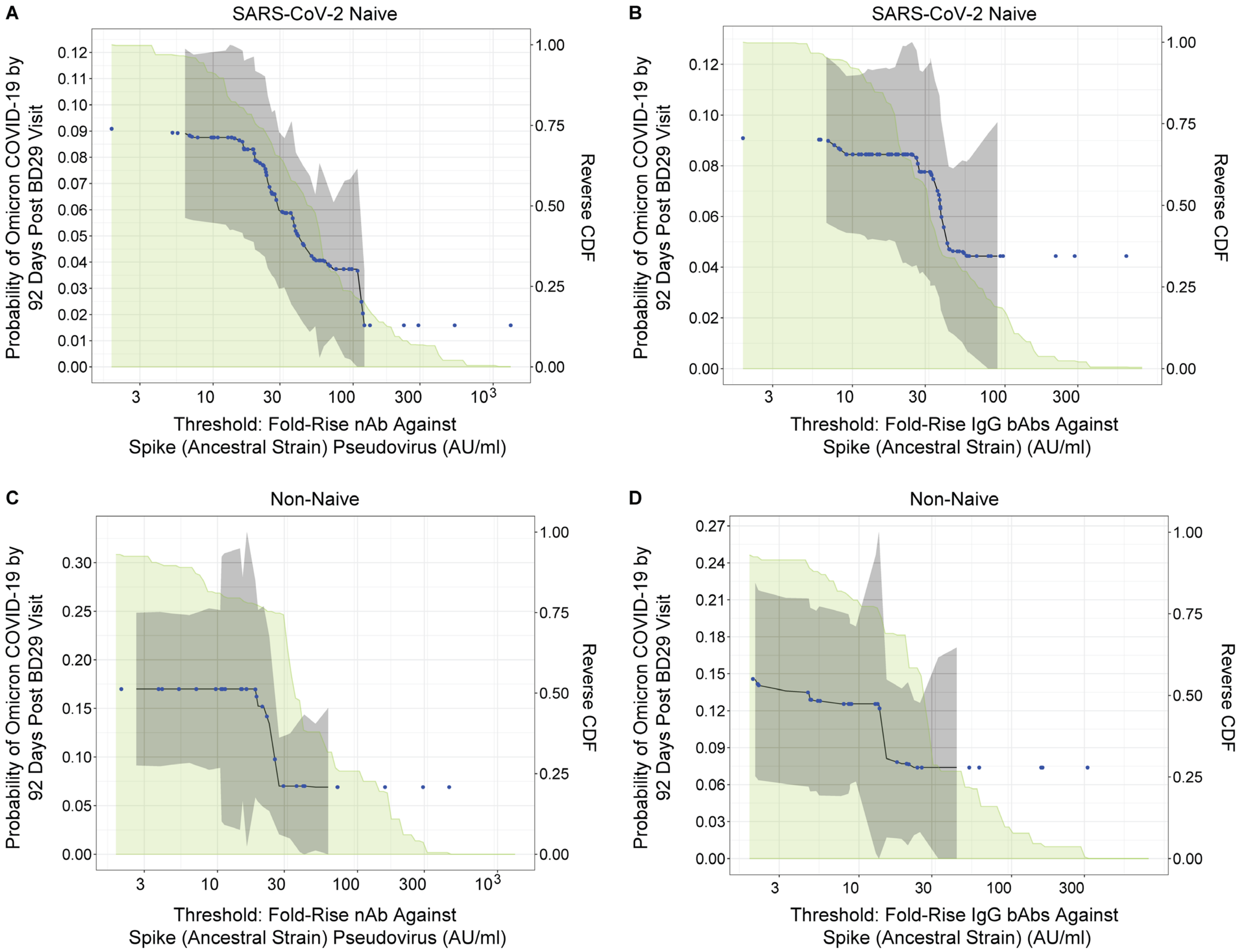

Supplementary Fig. 22. Cumulative incidence of Omicron COVID-19 by 92 days post BD29 by per-protocol boosted subgroups of (A, B) SARS-CoV-2 naive participants (N = 14,047) and (C, D) non-naive participants (N = 204) defined by (A, C) Fold-rise (BD29/BD1) Ancestral strain neutralizing antibody (nAb) titer or (B, D) Fold-rise (BD29/BD1) Spike IgG-Ancestral strain binding antibody (bAb) concentration above a threshold. The reverse cumulative distribution function (CDF) of each antibody marker is overlaid in green. Estimates and confidence intervals were adjusted using the assumption that the true threshold-response is nonincreasing. The blue dots correspond to marker values where an event is observed. The gray shaded area is pointwise 95% CIs. For ancestral strain nAbs, the units AU/ml can be transformed to International Units (IU50)/ml (see SAP).

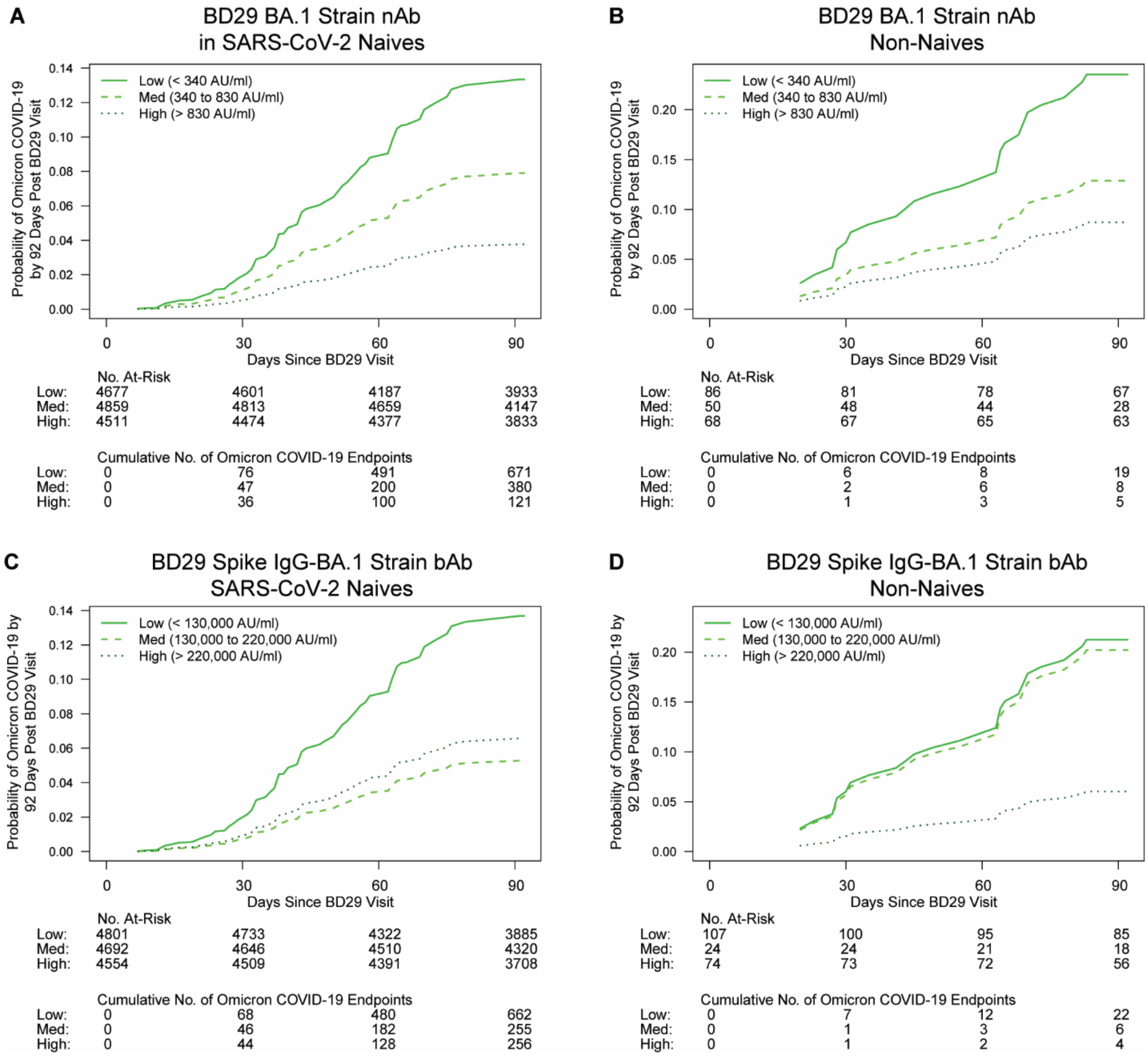

Supplementary Fig. 23. Cox-model-based marginalized Omicron COVID-19 cumulative incidence curves for subgroups of per-protocol boosted (A, C) SARS-CoV-2 naive (N = 14,047)or (B, D) non-naive participants (N = 204) defined by BD29 BA.1 strain antibody tertile. A, B: BD29 BA.1 strain neutralizing antibody (nAb); C, D: BD29 Spike IgG-BA.1 strain binding antibody (bAb). No. at risk = estimated number in the population for analysis, i.e. per-protocol (A, C) SARS-CoV-2 naive or (B, D) non-naive boosted participants not experiencing the Omicron COVID-19 endpoint or SARS-CoV-2 infected through 6 days post BD29 visit. Analyses were adjusted for baseline risk score, at-risk status, and community of color status.

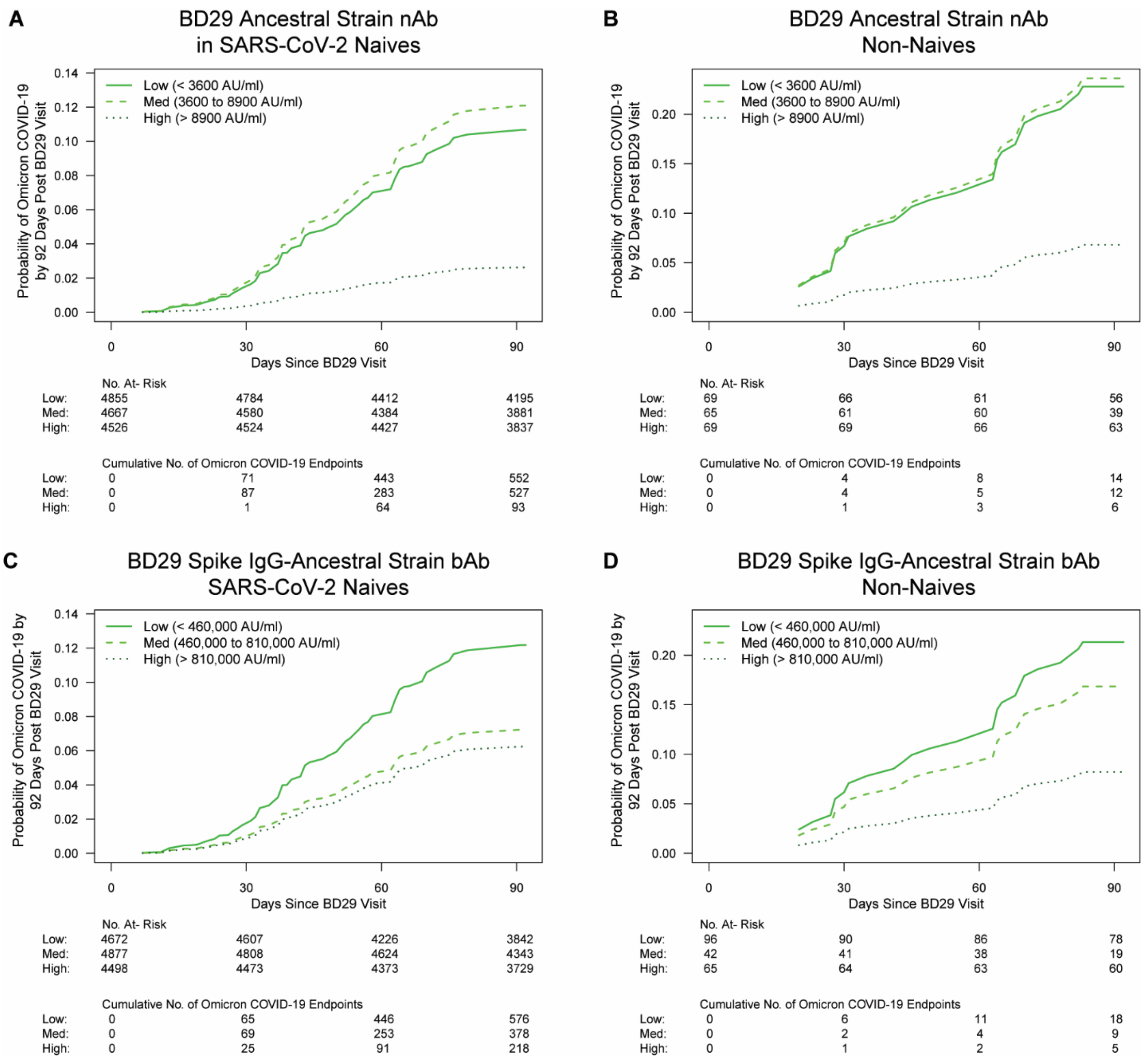

Supplementary Fig. 24. Cox-model-based marginalized Omicron COVID-19 cumulative incidence curves for subgroups of per-protocol boosted (A, C) SARS-CoV-2 naive (N = 14,047)or (B, D) non-naive (N = 204)participants defined by BD29 Ancestral strain antibody tertile. A, B: BD29 Ancestral strain neutralizing antibody (nAb); C, D: BD29 Spike IgG-Ancestral strain binding antibody (bAb). No. at risk = estimated number in the population for analysis, i.e. per-protocol (A, C) SARS-CoV-2 naive or (B, D) non-naive boosted participants not experiencing the Omicron COVID-19 endpoint or SARS-CoV-2 infected through 6 days post BD29 visit. Analyses were adjusted for baseline risk score, at-risk status, and community of color status. For ancestral strain nAbs, the units AU/ml can be transformed to International Units (IU50)/ml (see SAP).

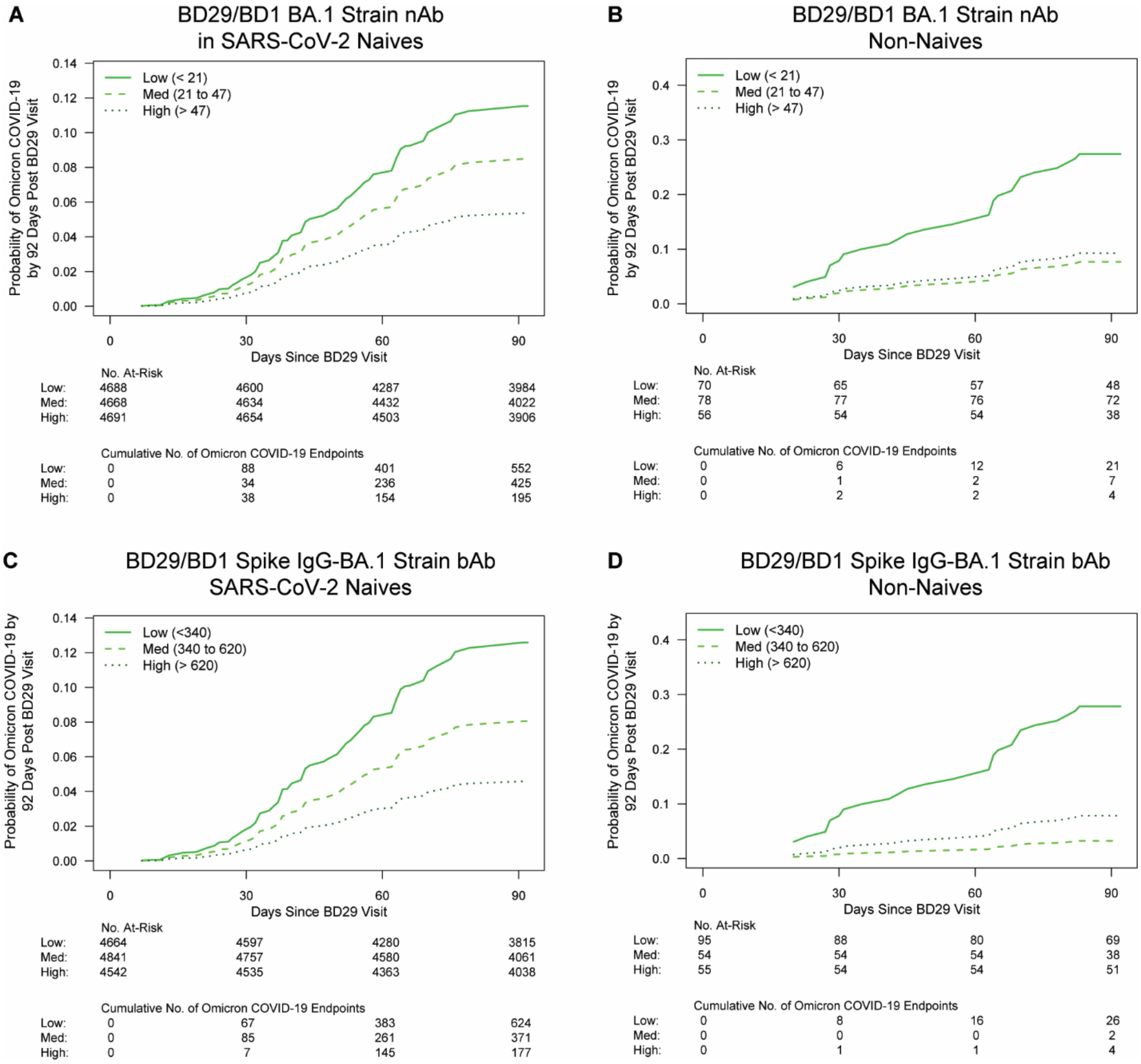

Supplementary Fig. 25. Cox-model-based marginalized Omicron COVID-19 cumulative incidence curves for subgroups of per-protocol boosted (A, C) SARS-CoV-2 naive (N = 14,047)or (B, D) non-naive (N = 204)participants defined by fold-rise (BD29/BD1) BA.1 strain antibody tertile. A, B: Fold-rise BA.1 strain neutralizing antibody (nAb); C, D: Fold-rise Spike IgG-BA.1 strain binding antibody (bAb). No. at risk = estimated number in the population for analysis, i.e. per-protocol (A, C) SARS-CoV-2 naive or (B, D) non-naive boosted participants not experiencing the Omicron COVID-19 endpoint or SARS-CoV-2 infected through 6 days post BD29 visit. Analyses were adjusted for baseline risk score, at-risk status, and community of color status.

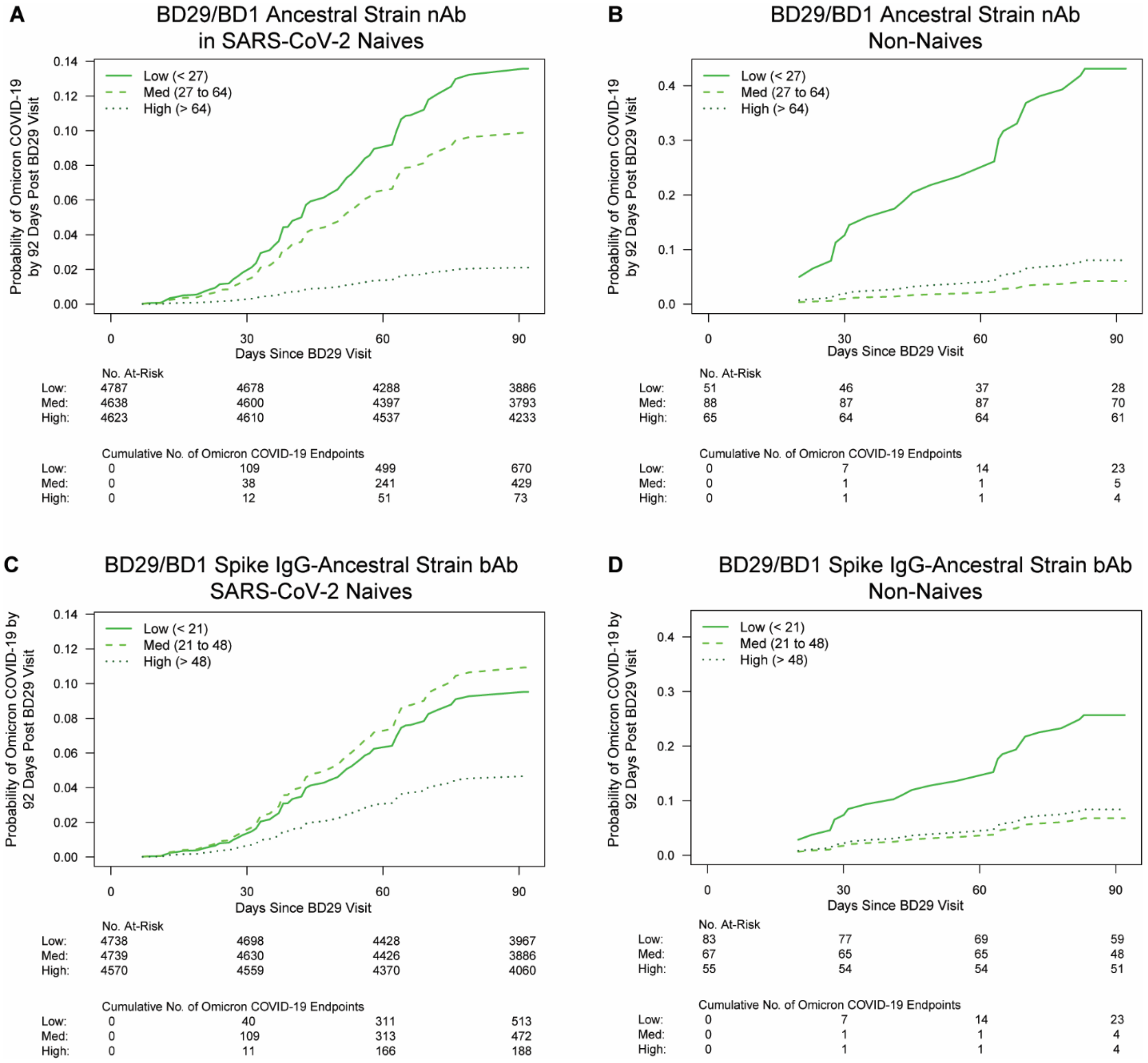

Supplementary Fig. 26. Cox-model-based marginalized Omicron COVID-19 cumulative incidence curves for subgroups of per-protocol boosted (A, C) SARS-CoV-2 naive (N = 14,047) or (B, D) non-naive (N = 204) participants defined by fold-rise (BD29/BD1) Ancestral strain antibody tertile. A, B: Fold-rise Ancestral strain neutralizing antibody (nAb); C, D: Fold-rise Spike IgG-Ancestral strain binding antibody (bAb). No. at risk = estimated number in the population for analysis, i.e. per-protocol (A, C) SARS-CoV-2 naive or (B, D) non-naive boosted participants not experiencing the Omicron COVID-19 endpoint or SARS-CoV-2 infected through 6 days post BD29 visit. Analyses were adjusted for baseline risk score, at-risk status, and community of color status.

Supplementary Table 7. Estimated hazard ratios of Omicron COVID-19 for the medium versus low and for the high versus low tertiles of the designated BD1 BA.1 strain and Ancestral strain markers, in SARS-CoV-2 naive and non-naive participants. Comparisons were made in per-protocol boosted participants. N/A, not applicable.

**BD1 Marker**	**Tertile***	**No. cases/ No. at-risk****	**Attack rate**	**Hazard Ratio Pt. Est.**	**Hazard Ratio 95% CI**	***P* value (2-sided)**	**Overall *P* value¶**	**FDR-adjusted *P* value†**	**FWER-adjusted *P* value†**
**A) SARS-CoV-2 Naive**									
Spike IgG-BA.1 strain bAbs	Low	342/4,734	0.0722	1	N/A	N/A	0.760	0.988	0.998
	Med	515/4,681	0.1100	1.44	(0.46, 4.47)	0.529			
	High	419/4,632	0.0905	1.11	(0.39, 3.18)	0.850			
BA.1 Strain nAbs	Low	667/6,196	0.1077	1	N/A	N/A	0.584	0.967	0.979
	Med	361/4,267	0.0846	0.72	(0.30, 1.77)	0.479			
	High	249/3,584	0.0695	0.60	(0.21, 1.68)	0.328			
Spike IgG-Ancestral Strain bAbs	Low	359/4,819	0.0745	1	N/A	N/A	0.775	0.988	0.998
	Med	497/4,690	0.1060	1.42	(0.50, 3.99)	0.507			
	High	420/4,538	0.0926	1.14	(0.43, 3.04)	0.791			
Ancestral Strain nAbs	Low	395/4,740	0.0833	1	N/A	N/A	0.954	0.988	1.000
	Med	413/4,681	0.0882	0.97	(0.38, 2.52)	0.953			
	High	469/4,626	0.1014	1.10	(0.40, 3.02)	0.848			
**B) Non-Naive**									
Spike IgG-BA.1 Strain bAbs	Low	4/39	0.1026	1	N/A	N/A	0.720	0.755	0.886
	Med	12/77	0.1558	1.57	(0.27, 9.01)	0.614			
	High	19/89	0.2135	1.95	(0.37, 10.24)	0.428			
BA.1 Strain nAbs	Low	12/76	0.1579	1	N/A	N/A	0.462	0.662	0.847
	Med	3/48	0.0625	0.48	(0.05, 4.35)	0.515			
	High	20/80	0.2500	1.70	(0.51, 5.72)	0.390			
Spike IgG-Ancestral Strain bAbs	Low	3/27	0.1111	1	N/A	N/A	0.059	0.408	0.399
	Med	10/115	0.0870	0.55	(0.09, 3.23)	0.507			
	High	22/62	0.3548	2.78	(0.46, 16.71)	0.263			
Ancestral Strain nAbs	Low	4/41	0.0976	1	N/A	N/A	0.198	0.499	0.664
	Med	9/87	0.1034	0.63	(0.11, 3.71)	0.613			
	High	22/76	0.2895	2.34	(0.46, 1.73)	0.303			

Baseline covariates were adjusted for baseline risk score, at risk status, and community of color status, all defined identically as in ref.[8]

*Antibody values defining the three tertiles were:

Spike IgG-BA.1 strain bAb: Low < 2000 AU/ml; Med 2000 to 5000 AU/ml; High > 5000 AU/ml.

BA.1 strain nAb: Low < 11 AU/ml; Med 11 to 15 AU/ml; High > 15 AU/ml.

Spike IgG-Ancestral strain bAb: Low < 12,000 AU/ml; Med 12,000 to 29,000 AU/ml; High > 29,000 AU/ml.

Ancestral strain nAb: Low < 73 AU/ml; Med 73 to 200 AU/ml; High > 200 AU/ml.

**No. at risk = estimated number in the population for analysis, i.e. per-protocol (A) SARS-CoV-2 naive and (B) non-naive boosted participants not experiencing the Omicron COVID-19 endpoint or SARS-CoV-2 infected through 6 days post BD29 visit; no. cases = number of this cohort with an observed Omicron COVID-19 endpoint.

¶The overall *P* value is from a generalized Wald test of whether the hazard rate of Omicron COVID-19 differed across the Low, Medium and High subgroups.
†*q*-value (false discovery rate, FDR) and family-wise error rate (FWER) were computed over the set of *P* values both for quantitative markers and categorical markers using the Westfall and Young permutation method (10,000 replicates).

Supplementary Table 8. Estimated hazard ratios of Omicron COVID-19 for the medium versus low and for the high versus low tertiles of the designated BD29 BA.1 strain and Ancestral strain markers, in SARS-CoV-2 naive and non-naive participants. Comparisons were made in per-protocol boosted participants. N/A, not applicable.

BD29 Marker	Tertile*	No. cases/ No. at-risk**	Attack rate	Hazard Ratio Pt. Est.	Hazard Ratio 95% CI	*P* value (2-sided)	Overall *P* value¶	FDR-adjusted *P* value†	FWER-adjusted *P* value†
**A) SARS-CoV-2 Naive**									
Spike IgG-BA.1 Strain bAbs	Low	693/4,801	0.1443	1	N/A	N/A	0.129	0.160	0.195
	Med	255/4,692	0.0543	0.37	(0.13, 1.02)	0.056			
	High	330/4,554	0.0725	0.46	(0.17, 1.26)	0.131			
BA.1 Strain nAbs	Low	699/4,677	0.1495	1	N/A	N/A	0.053	0.114	0.181
	Med	411/4,859	0.0846	0.57	(0.24, 1.38)	0.217			
	High	167/4,511	0.0370	0.27	(0.09, 0.78)	0.016			
Spike IgG-Ancestral Strain bAbs	Low	607/4,672	0.1299	1	N/A	N/A	0.403	0.420	0.418
	Med	378/4,877	0.0775	0.58	(0.21, 1.57)	0.282			
	High	292/4,498	0.0649	0.49	(0.17, 1.46)	0.203			
Ancestral Strain nAbs	Low	552/4,855	0.1137	1	N/A	N/A	0.018	0.114	0.101
	Med	601/4,667	0.1288	1.14	(0.47, 2.78)	0.769			
	High	123/4,526	0.0272	0.24	(0.07, 0.77)	0.017			
**B) Non-Naive**									
Spike IgG-BA.1 Strain bAbs	Low	23/107	0.2150	1	N/A	N/A	0.145	0.380	0.539
	Med	7/24	0.2917	0.94	(0.28, 3.13)	0.920			
	High	5/74	0.0676	0.24	(0.06, 1.01)	0.051			
BA.1 Strain nAbs	Low	20/86	0.2326	1	N/A	N/A	0.260	0.434	0.638
	Med	8/50	0.1600	0.49	(0.11, 2.23)	0.359			
	High	7/68	0.1029	0.32	(0.07, 1.49)	0.146			
Spike IgG-Ancestral Strain bAbs	Low	19/96	0.1979	1	N/A	N/A	0.263	0.434	0.638
	Med	9/42	0.2143	0.75	(0.24, 2.39)	0.629			
	High	7/65	0.1077	0.33	(0.09, 1.24)	0.102			
Ancestral Strain nAbs	Low	14/69	0.2029	1	N/A	N/A	0.135	0.380	0.539
	Med	13/65	0.2000	1.05	(0.35, 3.15)	0.933			
	High	8/69	0.1159	0.25	(0.05, 1.29)	0.098			

Baseline covariates were adjusted for baseline risk score, at risk status, and community of color status, all defined identically as in ref.[8]

*Antibody values defining the three tertiles are shown in Figures 2 and S22.

**No. at risk = estimated number in the population for analysis, i.e. per-protocol (A) SARS-CoV-2 naive and (B) non-naive boosted participants not experiencing the Omicron COVID-19 endpoint or SARS-CoV-2 infected through 6 days post BD29 visit; no. cases = number of this cohort with an observed Omicron COVID-19 endpoint.

¶The overall *P* value is from a generalized Wald test of whether the hazard rate of Omicron COVID-19 differed across the Low, Medium and High subgroups.

†*q*-value (false discovery rate, FDR) and family-wise error rate (FWER) were computed over the set of *P* values both for quantitative markers and categorical markers using the Westfall and Young permutation method (10,000 replicates).

Supplementary Table 9. Estimated hazard ratios of Omicron COVID-19 for the medium versus low and for the high versus low tertiles of the designated fold-rise (BD29/BD1) BA.1 strain and Ancestral strain markers, in SARS-CoV-2 naive and non-naive participants. Comparisons were made in per-protocol boosted participants. N/A, not applicable.

BD29/BD1 Marker	Tertile*	No. cases/ No. at-risk**	Attack rate	Hazard Ratio Pt. Est.	Hazard Ratio 95% CI	*P* value (2-sided)	Overall *P* value¶	FDR-adjusted *P* value†	FWER-adjusted *P* value†
**A) SARS-CoV-2 Naive**									
Spike IgG-BA.1 Strain bAbs	Low	653/4,664	0.1400	1	N/A	N/A	0.092	0.181	0.280
	Med	417/4,841	0.0861	0.62	(0.25, 1.55)	0.310			
	High	207/4,542	0.0456	0.35	(0.13, 0.90)	0.030			
BA.1 Strain nAbs	Low	581/4,688	0.1239	1	N/A	N/A	0.294	0.340	0.412
	Med	456/4,668	0.0977	0.72	(0.26, 1.98)	0.529			
	High	240/4,691	0.0512	0.45	(0.16, 1.23)	0.119			
Spike IgG-Ancestral Strain bAbs	Low	513/4,738	0.1083	1	N/A	N/A	0.178	0.251	0.412
	Med	562/4,739	0.1186	1.16	(0.47, 2.84)	0.750			
	High	203/4,570	0.0444	0.48	(0.18, 1.23)	0.125			
Ancestral Strain nAbs	Low	685/4,787	0.1431	1	N/A	N/A	0.002	0.039	0.021
	Med	489/4,638	0.1054	0.71	(0.32, 1.58)	0.406			
	High	103/4,623	0.0223	0.15	(0.05, 0.42)	<0.001			
**B) Non-Naive**									
Spike IgG-BA.1 Strain bAbs	Low	28/95	0.2947	1	N/A	N/A	0.018	0.123	0.194
	Med	2/54	0.0370	0.09	(0.01, 1.25)	0.074			
	High	5/55	0.0909	0.23	(0.07, 0.75)	0.015			
BA.1 Strain nAbs	Low	23/70	0.3286	1	N/A	N/A	0.037	0.123	0.236
	Med	7/78	0.0897	0.24	(0.07, 0.83)	0.025			
	High	5/56	0.0893	0.29	(0.07, 1.22)	0.091			
Spike IgG-Ancestral Strain bAbs	Low	26/83	0.3133	1	N/A	N/A	0.078	0.151	0.261
	Med	4/67	0.0597	0.23	(0.04, 1.21)	0.082			
	High	5/55	0.0909	0.28	(0.07, 1.08)	0.064			
Ancestral Strain nAbs	Low	25/51	0.4902	1	N/A	N/A	<0.001	0.040	0.020
	Med	5/88	0.0568	0.07	(0.02, 0.30)	<0.001			
	High	5/65	0.0769	0.14	(0.04, 0.45)	<0.001			

Baseline covariates were adjusted for baseline risk score, at risk status, and community of color status, all defined identically as in ref.[8]

*Antibody values defining the three tertiles are shown in Figures S23 and S24.

**No. at risk = estimated number in the population for analysis, i.e. per-protocol (A) SARS-CoV-2 naive and (B) non-naive boosted participants not experiencing the Omicron COVID-19 endpoint or SARS-CoV-2 infected through 6 days post BD29 visit; no. cases = number of this cohort with an observed Omicron COVID-19 endpoint.

¶The overall *P* value is from a generalized Wald test of whether the hazard rate of Omicron COVID-19 differed across the Low, Medium and High subgroups.

†*q*-value (false discovery rate, FDR) and family-wise error rate (FWER) were computed over the set of *P* values both for quantitative markers and categorical markers using the Westfall and Young permutation method (10,000 replicates).

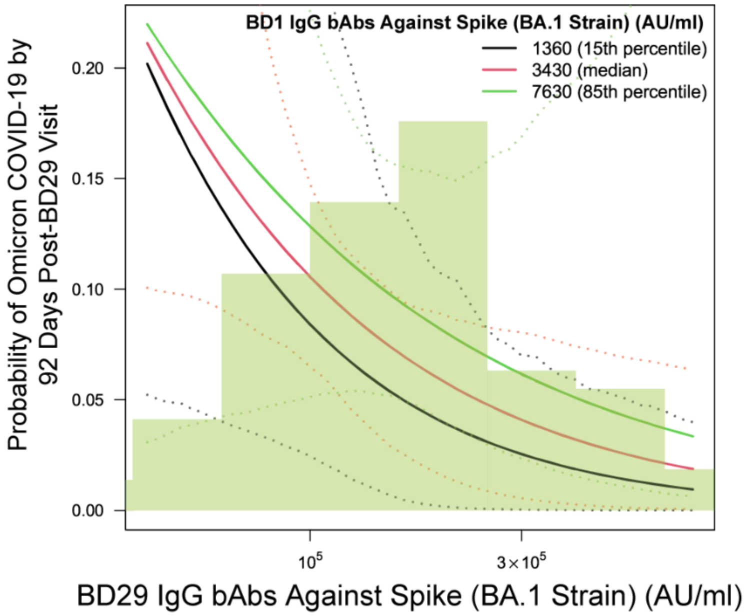

Supplementary Fig. 27. Marginalized cumulative incidence curves of Omicron COVID-19 risk across a range of BD29 Spike IgG-BA.1 strain binding antibody (bAb) levels and within each tertile of BD1 Spike IgG-BA.1 strain bAb among SARS-CoV-2 naive participants (N = 14,047). The solid lines indicate the mean probabilities. The dotted lines indicate bootstrap pointwise 95% CIs. The light green histogram plots the distribution of the BD29 marker**.** The Cox regression model adjusted for the minority indicator, heightened risk for severe COVID-19, predicted risk score, BD1 Spike IgG-BA.1 strain bAb and interaction between BD1 and BD29 Spike IgG-BA.1 strain bAb levels.

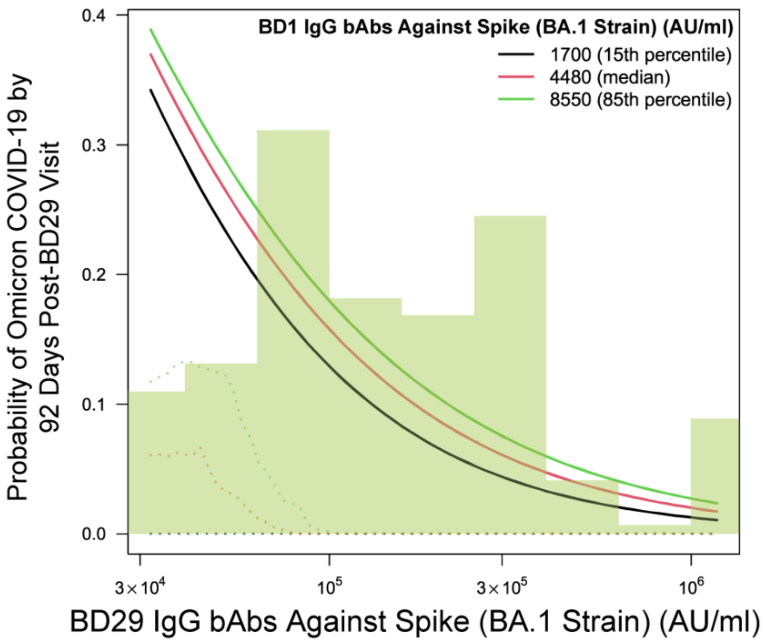

Supplementary Fig. 28. Marginalized cumulative incidence curves of Omicron COVID-19 risk across a range of BD29 Spike IgG-BA.1 strain binding antibody (bAb) levels and within each tertile of BD1 Spike IgG-BA.1 strain bAb among non-naive participants (N = 204). The solid lines indicate the mean probabilities. The dotted lines indicate bootstrap pointwise 95% CIs. The light green histogram plots the distribution of the BD29 marker**.** The Cox regression model adjusted for the minority indicator, heightened risk for severe COVID-19, predicted risk score, BD1 Spike IgG-BA.1 strain bAb and interaction between BD1 and BD29 Spike IgG-BA.1 strain bAb levels.

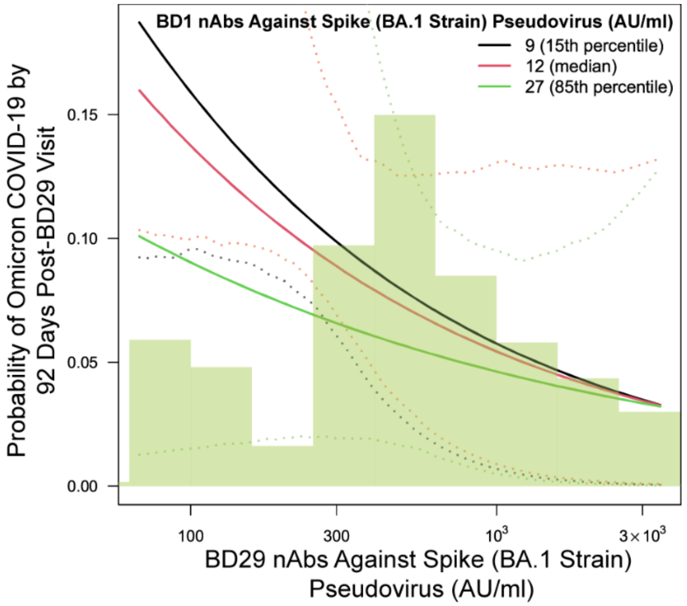

Supplementary Fig. 29. Marginalized cumulative incidence curves of Omicron COVID-19 risk across a range of BD29 nAb BA.1 levels and within each tertile of BD1 BA.1 strain neutralizing antibody (nAb) level among SARS-CoV-2 naive participants (N = 14,047). The solid lines indicate the mean probabilities. The dotted lines indicate bootstrap pointwise 95% CIs. The light green histogram plots the distribution of the BD29 marker**.** The Cox regression model adjusted for the minority indicator, heightened risk for severe COVID-19, predicted risk score, BD1 BA.1 strain nAb and interaction between BD1 and BD29 BA.1 strain nAb levels.

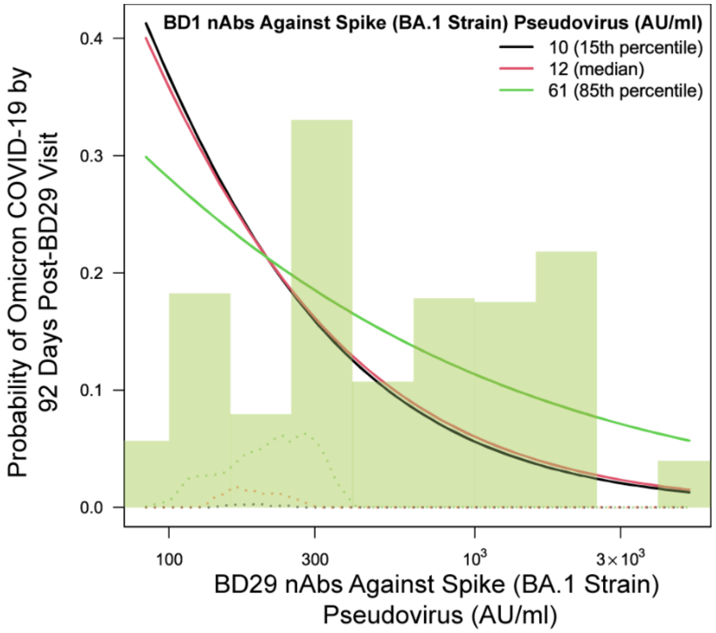

Supplementary Fig. 30. Marginalized cumulative incidence curves of Omicron COVID-19 risk across a range of BD29 BA.1 strain neutralizing antibody (nAb) levels and within each tertile of BD1 BA.1 strain nAb among non-naive participants (N = 204). The solid lines indicate the mean probabilities. The dotted lines indicate bootstrap pointwise 95% CIs. The light green histogram plots the distribution of the BD29 marker**.** The Cox regression model adjusted for the minority indicator, heightened risk for severe COVID-19, predicted risk score, BD1 BA.1 strain nAb and interaction between BD1 and BD29 BA.1 strain nAb levels.

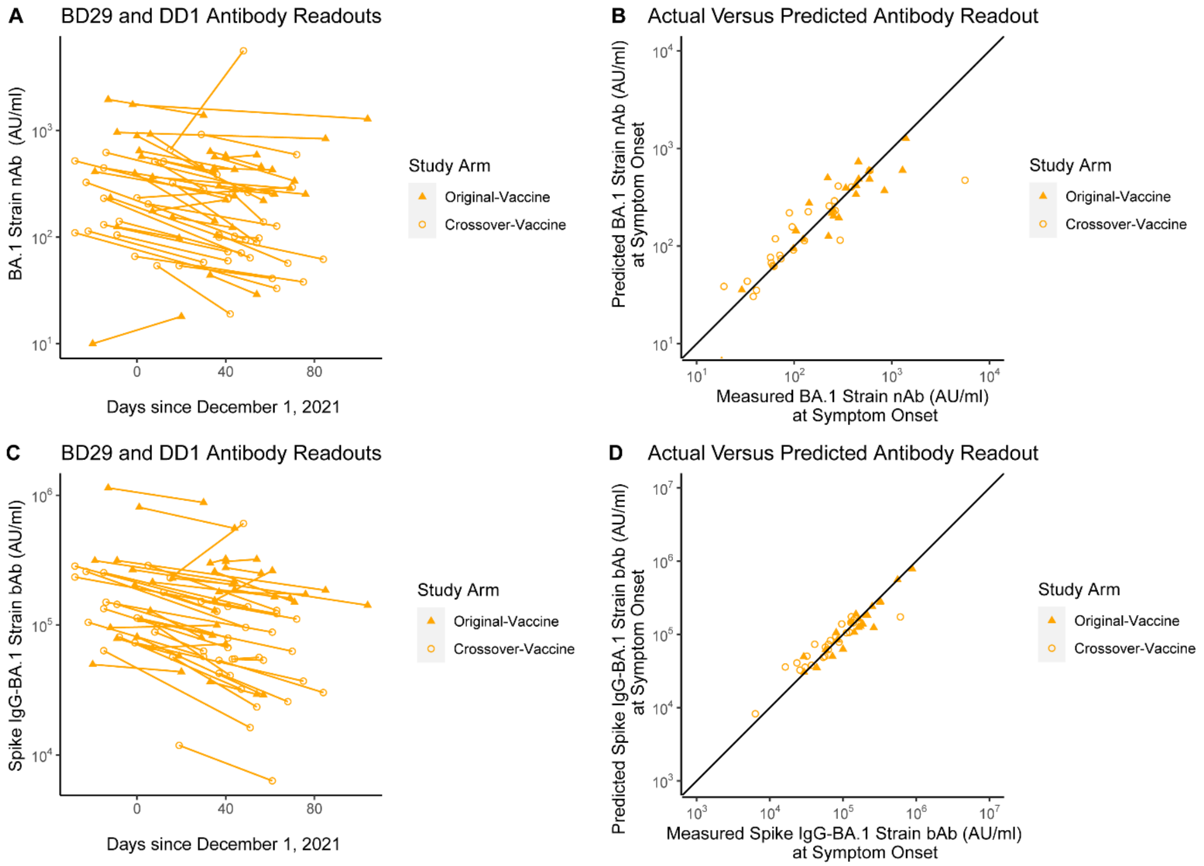

Supplementary Fig. 31. (A, C) BD29 and DD1 antibody levels and (B, D) predicted versus actual antibody levels at DD1 for (A, B) BA.1 strain neutralizing antibody (nAb) and (C, D) Spike IgG-BA.1 strain binding antibody (bAb) levels among SARS-CoV-2 naive participants. Filled orange triangles designate the original-vaccine arm; open orange circles designate the crossover-vaccine arm. The median slope for BA.1 strain nAbs was -0.0044 per day (half-life 68 days). The median slope for Spike IgG-BA.1 strain bAbs was -0.0034 per day (half-life 80 days) (N=47).

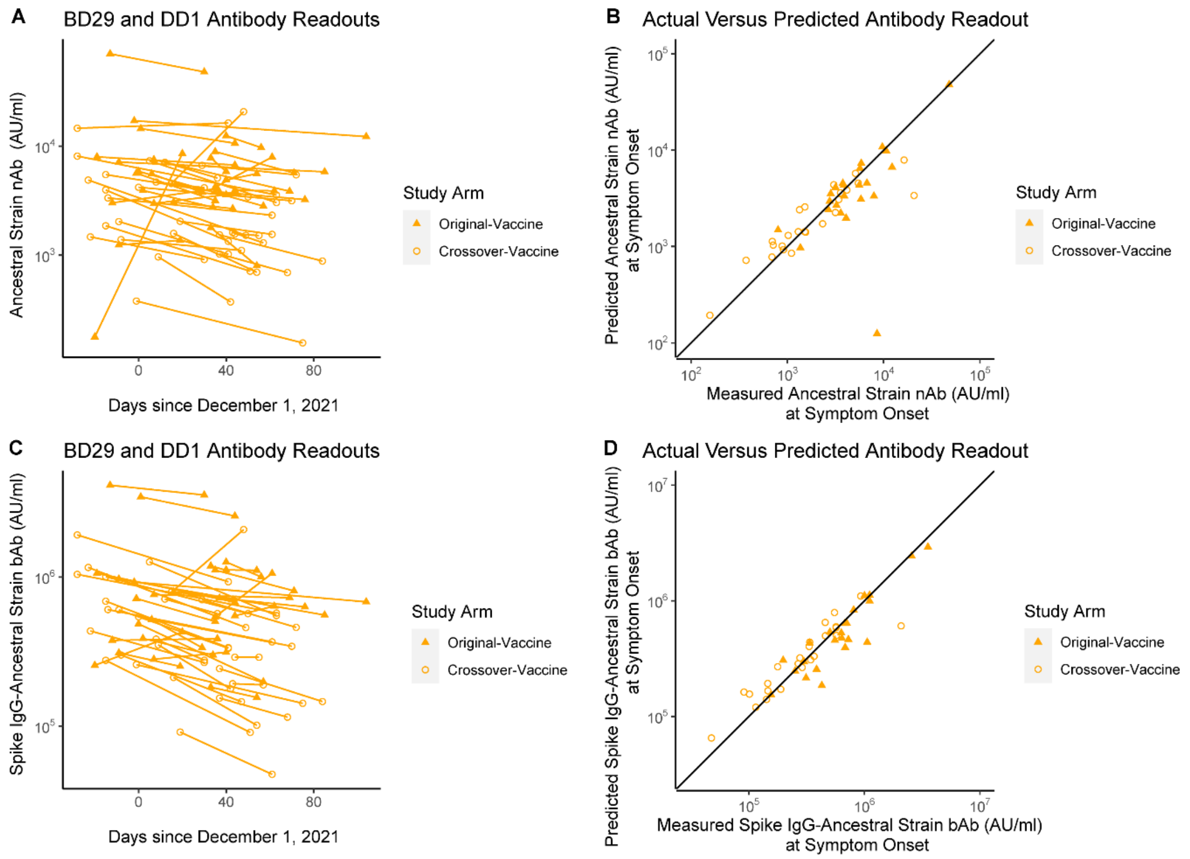

Supplementary Fig. 32. (A, C) BD29 and DD1 antibody levels and (B, D) predicted versus actual antibody levels at DD1 for (A, B) Ancestral strain neutralizing antibody (nAb) and (C, D) Spike IgG-Ancestral strain binding antibody (bAb) level among SARS-CoV-2 naive participants. Filled orange triangles designate the original-vaccine arm; open orange circles designate the crossover-vaccine arm. -0.0025. The median slope for Ancestral strain nAbs was -0.0039 per day (half-life 78 days). The median slope for Spike IgG-BA.1 strain bAbs was -0.0035 per day (half-life 86 days). (N=47)

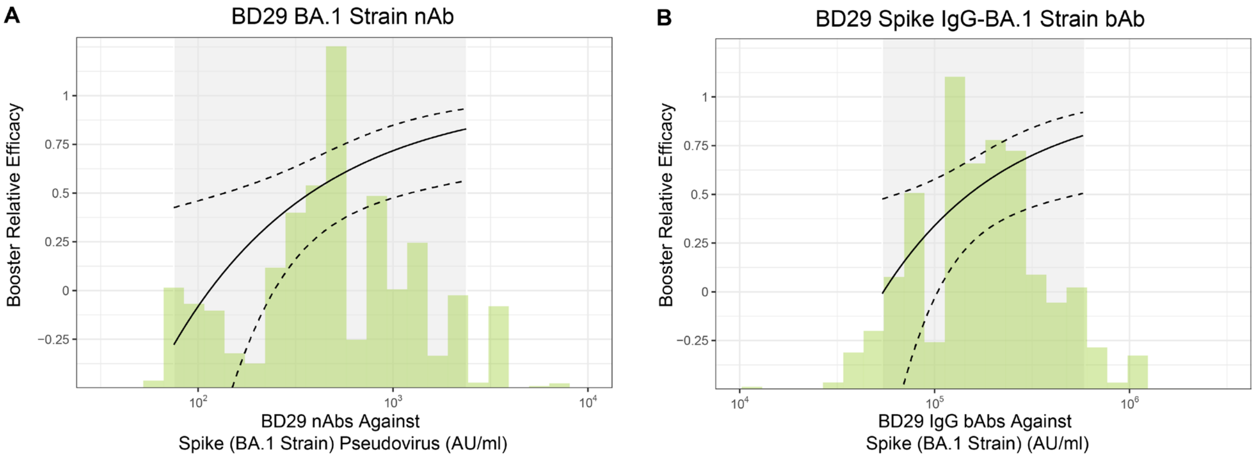

Supplementary Fig. 33. Correlate of booster relative efficacy curves against Omicron COVID-19 among SARS-CoV-2 naive participants as a function of measured antibody level at BD29. A) BD29 BA.1 strain nAb, B) BD29 Spike IgG-BA.1 strain bAb. The solid lines show the relative efficacy of three-dose mRNA-1273 vs. two-dose mRNA-1273. The dashed black lines are 95% confidence intervals. The green histograms are an estimate of the density of BD29 antibody marker level in per-protocol boosted SARS-CoV-2 naive participants. The grey shades indicate the middle 90th (5th percentile to 95th percentile) of the marker distribution.

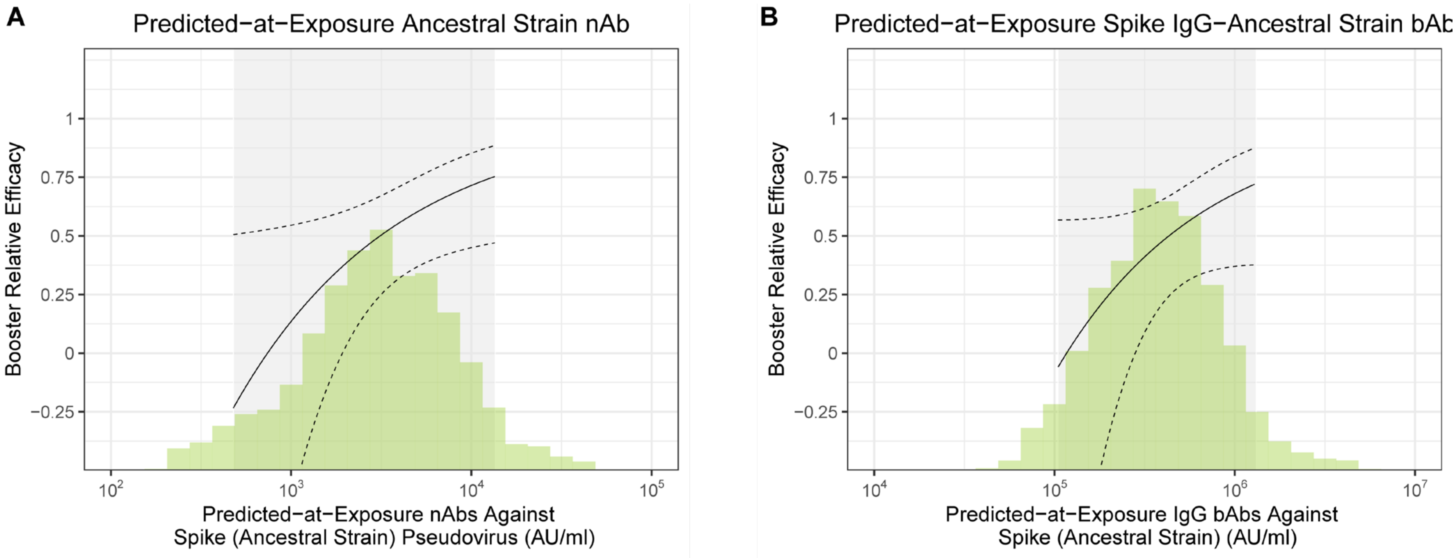

Supplementary Fig. 34. Booster relative efficacy against Omicron COVID-19 among SARS-CoV-2 naive participants (N=2464) as a function of the predicted antibody level [A: Ancestral strain neutralizing antibody (nAb), B: Spike IgG-Ancestral strain binding antibody (bAb)] at the time of exposure to SARS-CoV-2. The solid lines indicate the mean relative efficacy of three-dose mRNA-1273 vs. two-dose mRNA-1273. The dotted lines indicate bootstrap pointwise 95% CIs. The light green histogram plots the distribution of the BD29 marker. The grey shades indicate the middle 90th (5th percentile to 95th percentile) of the marker distribution.

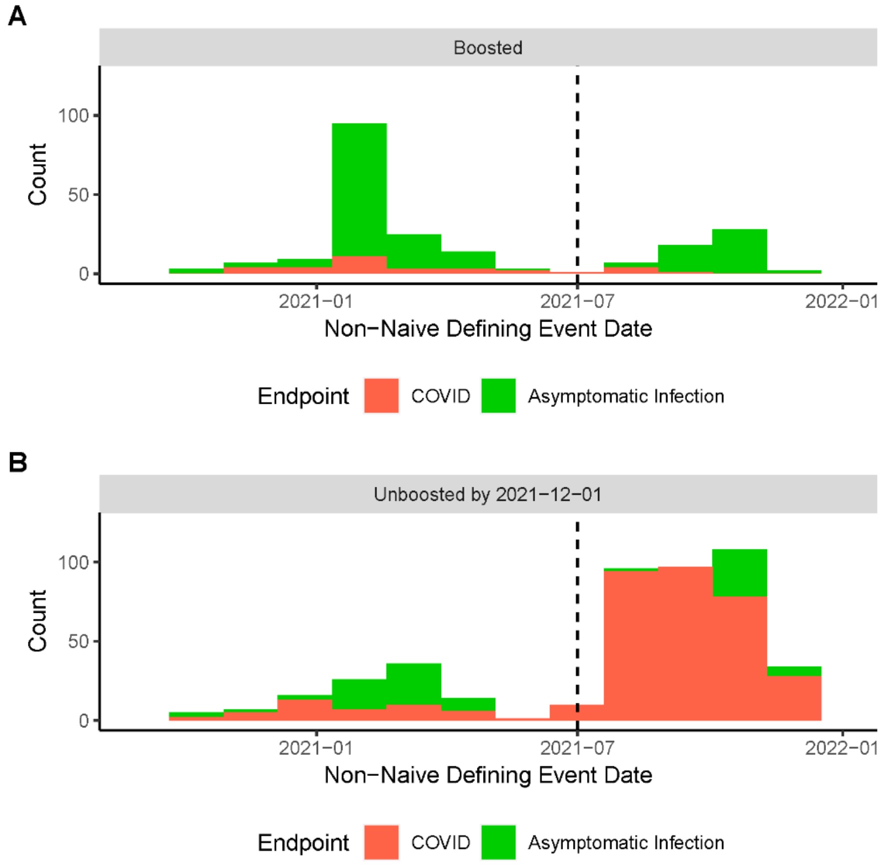

Supplementary Fig. 35. Distribution of the day of the non-naive defining event for (A) boosted (N=282) and (B) unboosted participants (N=378).

Supplementary Table 10. Discrete Super Learner performance across all 92 variable sets sorted by cross-validated area under the ROC (CV-AUC) performance for predicting occurrence of Omicron COVID-19 in SARS-CoV-2 naive per-protocol boosted participants. bAb = binding antibody; BRF = baseline risk factors; fold-rise = BD29/BD1; nAb = neutralizing antibody.

Variable Set	CV-AUC (95% CI)
BRF + BD29-bAb-Spike-BA.1 + BD29-bAb-Spike-Ancestral + BD29-bAb-RBD-Ancestral + BD29-nAb-BA.1 + BD29-nAb-Ancestral + Fold-rise-bAb-Spike-BA.1 + Fold-rise-bAb-Spike-Ancestral + Fold-rise-bAb-RBD-Ancestral + Fold-rise-nAb-BA.1 + Fold-rise-nAb-Ancestral	0.686 [0.600, 0.761]
BRF + BD1-bAb-RBD-Ancestral + BD1-nAb-BA.1 + BD1-nAb-Ancestral + Fold-rise-bAb-RBD-Ancestral + Fold-rise-nAb-BA.1 + Fold-rise-nAb-Ancestral	0.686 [0.600, 0.761]
BRF + BD1-bAb-Spike-BA.1 + BD1-bAb-Spike-Ancestral + BD1-nAb-BA.1 + BD1-nAb-Ancestral + BD29-bAb-Spike-BA.1 + BD29-bAb-Spike-Ancestral + BD29-nAb-BA.1 + BD29-nAb-Ancestral	0.686 [0.600, 0.761]
BRF + BD29-bAb-Spike-BA.1 + BD29-bAb-Spike-Ancestral + BD29-nAb-BA.1 + BD29-nAb-Ancestral	0.685 [0.599, 0.760]
BRF + BD1-bAb-Spike-BA.1 + BD1-bAb-Spike-Ancestral + BD1-bAb-RBD-Ancestral + BD1-nAb-BA.1 + BD1-nAb-Ancestral + BD29-bAb-Spike-BA.1 + BD29-bAb-Spike-Ancestral + BD29-bAb-RBD-Ancestral + BD29-nAb-BA.1 + BD29-nAb-Ancestral	0.685 [0.599, 0.760]
BRF + BD1-bAb-Spike-BA.1 + BD1-bAb-Spike-Ancestral + BD1-bAb-RBD-Ancestral + BD1-nAb-BA.1 + BD1-nAb-Ancestral + BD29-bAb-Spike-BA.1 + BD29-bAb-Spike-Ancestral + BD29-bAb-RBD-Ancestral + BD29-nAb-BA.1 + BD29-nAb-Ancestral + Fold-rise-bAb-Spike-BA.1 + Fold-rise-bAb-Spike-Ancestral + Fold-rise-bAb-RBD-Ancestral + Fold-rise-nAb-BA.1 + Fold-rise-nAb-Ancestral	0.684 [0.598, 0.760]
BRF + BD29-nAb-BA.1 + Fold-rise-nAb-BA.1	0.684 [0.598, 0.759]
BRF + BD1-bAb-RBD-Ancestral + BD1-nAb-BA.1 + BD1-nAb-Ancestral + BD29-bAb-RBD-Ancestral + BD29-nAb-BA.1 + BD29-nAb-Ancestral	0.682 [0.596, 0.758]
BRF + BD29-nAb-BA.1 + BD29-nAb-Ancestral	0.682 [0.597, 0.757]
BRF + BD29-nAb-BA.1 + BD29-nAb-Ancestral + Fold-rise-nAb-BA.1 + Fold-rise-nAb-Ancestral	0.681 [0.595, 0.756]
BRF + BD1-nAb-BA.1 + BD1-nAb-Ancestral + BD29-nAb-BA.1 + BD29-nAb-Ancestral	0.681 [0.594, 0.756]
BRF + BD29-bAb-Spike-BA.1 + BD29-bAb-Spike-Ancestral + BD29-nAb-BA.1 + BD29-nAb-Ancestral + Fold-rise-bAb-Spike-BA.1 + Fold-rise-bAb-Spike-Ancestral + Fold-rise-nAb-BA.1 + Fold-rise-nAb-Ancestral	0.681 [0.595, 0.756]
BRF + BD1-bAb-Spike-BA.1 + BD1-nAb-BA.1 + BD29-bAb-Spike-BA.1 + BD29-nAb-BA.1	0.680 [0.593, 0.755]
BRF + BD29-bAb-Spike-BA.1 + BD29-nAb-BA.1 + Fold-rise-bAb-Spike-BA.1 + Fold-rise-nAb-BA.1	0.679 [0.593, 0.755]
BRF + BD1-bAb-Spike-BA.1 + BD1-bAb-Spike-Ancestral + BD1-nAb-BA.1 + BD1-nAb-Ancestral + Fold-rise-bAb-Spike-BA.1 + Fold-rise-bAb-Spike-Ancestral + Fold-rise-nAb-BA.1 + Fold-rise-nAb-Ancestral	0.679 [0.592, 0.755]
BRF + BD1-nAb-BA.1 + BD29-nAb-BA.1	0.678 [0.592, 0.754]
BRF + BD1-bAb-Spike-BA.1 + BD1-bAb-Spike-Ancestral + BD1-bAb-RBD-Ancestral + BD1-nAb-BA.1 + BD1-nAb-Ancestral + Fold-rise-bAb-Spike-BA.1 + Fold-rise-bAb-Spike-Ancestral + Fold-rise-bAb-RBD-Ancestral + Fold-rise-nAb-BA.1 + Fold-rise-nAb-Ancestral	0.678 [0.592, 0.754]
BRF + BD1-bAb-Spike-BA.1 + BD1-nAb-BA.1 + Fold-rise-bAb-Spike-BA.1 + Fold-rise-nAb-BA.1	0.678 [0.591, 0.754]
BRF + BD29-bAb-RBD-Ancestral + BD29-nAb-BA.1 + BD29-nAb-Ancestral + Fold-rise-bAb-RBD-Ancestral + Fold-rise-nAb-BA.1 + Fold-rise-nAb-Ancestral	0.678 [0.592, 0.753]

BRF + BD29-bAb-RBD-Ancestral + BD29-nAb-BA.1 + BD29-nAb-Ancestral	0.677 [0.591, 0.753]
BRF + BD29-bAb-Spike-BA.1 + BD29-nAb-BA.1	0.677 [0.591, 0.753]
BRF + BD29-nAb-BA.1	0.677 [0.591, 0.753]
BRF + BD1-nAb-BA.1 + BD1-nAb-Ancestral + Fold-rise-nAb-BA.1 + Fold-rise-nAb-Ancestral	0.677 [0.590, 0.753]
BRF + BD1-nAb-BA.1 + Fold-rise-nAb-BA.1	0.673 [0.586, 0.750]
BRF + BD29-bAb-Spike-Ancestral + BD29-nAb-Ancestral	0.662 [0.575, 0.740]
BRF + BD29-nAb-Ancestral	0.659 [0.572, 0.737]
BRF + BD29-bAb-Spike-BA.1 + BD29-bAb-Spike-Ancestral + BD29-bAb-RBD-Ancestral	0.659 [0.571, 0.738]
BRF + BD1-bAb-Spike-Ancestral + BD1-nAb-Ancestral + BD29-bAb-Spike-Ancestral + BD29-nAb-Ancestral	0.659 [0.571, 0.737]
BRF + BD29-bAb-RBD-Ancestral + BD29-nAb-Ancestral	0.657 [0.569, 0.735]
BRF + BD1-bAb-Spike-Ancestral + BD1-bAb-RBD-Ancestral + BD1-nAb-Ancestral + BD29-bAb-Spike-Ancestral + BD29-bAb-RBD-Ancestral + BD29-nAb-Ancestral	0.657 [0.568, 0.736]
BRF + BD29-bAb-Spike-Ancestral + BD29-bAb-RBD-Ancestral + BD29-nAb-Ancestral + Fold-rise-bAb-Spike-Ancestral + Fold-rise-bAb-RBD-Ancestral + Fold-rise-nAb-Ancestral	0.656 [0.568, 0.735]
BRF + BD1-bAb-Spike-BA.1 + BD1-bAb-Spike-Ancestral + BD1-bAb-RBD-Ancestral + BD29-bAb-Spike-BA.1 + BD29-bAb-Spike-Ancestral + BD29-bAb-RBD-Ancestral	0.656 [0.568, 0.734]
BRF + BD1-nAb-Ancestral + BD29-nAb-Ancestral	0.655 [0.567, 0.734]
BRF + BD29-nAb-Ancestral + Fold-rise-nAb-Ancestral	0.654 [0.566, 0.732]
BRF + BD29-bAb-Spike-Ancestral + BD29-nAb-Ancestral + Fold-rise-bAb-Spike-Ancestral + Fold-rise-nAb-Ancestral	0.653 [0.565, 0.732]
BRF + BD29-bAb-RBD-Ancestral + BD29-nAb-Ancestral + Fold-rise-bAb-RBD-Ancestral + Fold-rise-nAb-Ancestral	0.651 [0.563, 0.730]
BRF + BD1-bAb-RBD-Ancestral + BD1-nAb-Ancestral + BD29-bAb-RBD-Ancestral + BD29-nAb-Ancestral	0.650 [0.562, 0.729]
BRF + BD1-bAb-Spike-Ancestral + BD1-bAb-RBD-Ancestral + BD29-bAb-Spike-Ancestral + BD29-bAb-RBD-Ancestral	0.647 [0.557, 0.726]
BRF + BD29-bAb-Spike-Ancestral + BD29-bAb-RBD-Ancestral	0.646 [0.557, 0.726]
BRF + BD29-bAb-Spike-Ancestral + BD29-bAb-RBD-Ancestral + Fold-rise-bAb-Spike-Ancestral + Fold-rise-bAb-RBD-Ancestral	0.645 [0.555, 0.725]
BRF + BD1-bAb-Spike-BA.1 + BD1-nAb-BA.1	0.641 [0.552, 0.721]
BRF + BD1-bAb-Spike-BA.1 + BD1-bAb-Spike-Ancestral + BD1-nAb-BA.1 + BD1-nAb-Ancestral	0.640 [0.551, 0.720]
BRF + BD1-bAb-RBD-Ancestral + BD1-nAb-BA.1 + BD1-nAb-Ancestral	0.637 [0.548, 0.718]
BRF + BD1-nAb-Ancestral + Fold-rise-nAb-Ancestral	0.637 [0.548, 0.717]
BRF + BD29-bAb-Spike-BA.1 + BD29-bAb-Spike-Ancestral + BD29-bAb-RBD-Ancestral + Fold-rise-bAb-Spike-BA.1 + Fold-rise-bAb-Spike-Ancestral + Fold-rise-bAb-RBD-Ancestral	0.635 [0.546, 0.716]
BRF + BD1-nAb-BA.1 + BD1-nAb-Ancestral	0.633 [0.544, 0.714]
BRF + BD1-nAb-BA.1	0.632 [0.543, 0.713]
BRF + BD1-bAb-Spike-Ancestral + BD1-nAb-Ancestral + Fold-rise-bAb-Spike-Ancestral + Fold-rise-nAb-Ancestral	0.629 [0.540, 0.710]
BRF + BD29-bAb-RBD-Ancestral + Fold-rise-bAb-RBD-Ancestral	0.625 [0.537, 0.706]
BRF + BD1-bAb-Spike-Ancestral + BD1-bAb-RBD-Ancestral + BD1-nAb-Ancestral + Fold-rise-bAb-Spike-Ancestral + Fold-rise-bAb-RBD-Ancestral + Fold-rise-nAb-Ancestral	0.623 [0.534, 0.704]

BRF + BD29-bAb-Spike-BA.1 + BD29-bAb-Spike-Ancestral	0.622 [0.533, 0.703]
BRF + BD1-bAb-RBD-Ancestral + BD29-bAb-RBD-Ancestral	0.621 [0.533, 0.702]
BRF + BD1-bAb-Spike-Ancestral + BD1-bAb-RBD-Ancestral + Fold-rise-bAb-Spike-Ancestral + Fold-rise-bAb-RBD-Ancestral	0.620 [0.530, 0.702]
BRF + BD29-bAb-RBD-Ancestral	0.620 [0.531, 0.701]
BRF + BD29-bAb-Spike-BA.1 + BD29-bAb-Spike-Ancestral + Fold-rise-bAb-Spike-BA.1 + Fold-rise-bAb-Spike-Ancestral	0.618 [0.529, 0.699]
BRF + BD1-bAb-RBD-Ancestral + BD1-nAb-Ancestral + Fold-rise-bAb-RBD-Ancestral + Fold-rise-nAb-Ancestral	0.616 [0.528, 0.698]
BRF + BD1-bAb-Spike-BA.1 + BD1-bAb-Spike-Ancestral + BD29-bAb-Spike-BA.1 + BD29-bAb-Spike-Ancestral	0.612 [0.523, 0.694]
BRF + BD1-bAb-RBD-Ancestral + Fold-rise-bAb-RBD-Ancestral	0.611 [0.522, 0.693]
BRF + BD29-bAb-Spike-BA.1 + Fold-rise-bAb-Spike-BA.1	0.605 [0.516, 0.688]
BRF + Fold-rise-bAb-RBD-Ancestral + Fold-rise-nAb-BA.1 + Fold-rise-nAb-Ancestral	0.603 [0.514, 0.686]
BRF + Fold-rise-bAb-Spike-Ancestral + Fold-rise-bAb-RBD-Ancestral	0.603 [0.513, 0.686]
BRF + BD1-bAb-Spike-BA.1 + BD29-bAb-Spike-BA.1	0.603 [0.514, 0.685]
BRF + Fold-rise-bAb-Spike-BA.1 + Fold-rise-bAb-Spike-Ancestral + Fold-rise-nAb-BA.1 + Fold-rise-nAb-Ancestral	0.601 [0.512, 0.685]
BRF + Fold-rise-nAb-BA.1 + Fold-rise-nAb-Ancestral	0.597 [0.508, 0.681]
BRF + BD1-bAb-Spike-BA.1 + BD1-bAb-Spike-Ancestral + BD1-bAb-RBD-Ancestral + Fold-rise-bAb-Spike-BA.1 + Fold-rise-bAb-Spike-Ancestral + Fold-rise-bAb-RBD-Ancestral	0.597 [0.507, 0.680]
BRF + Fold-rise-bAb-Spike-BA.1 + Fold-rise-bAb-Spike-Ancestral + Fold-rise-bAb-RBD-Ancestral	0.596 [0.506, 0.680]
BRF + BD29-bAb-Spike-BA.1	0.594 [0.505, 0.677]
BRF + Fold-rise-bAb-Spike-BA.1 + Fold-rise-nAb-BA.1	0.593 [0.504, 0.676]
BRF + BD29-bAb-Spike-Ancestral + Fold-rise-bAb-Spike-Ancestral	0.590 [0.501, 0.674]
BRF + BD1-bAb-Spike-BA.1 + Fold-rise-bAb-Spike-BA.1	0.587 [0.498, 0.670]
BRF + Fold-rise-bAb-RBD-Ancestral + Fold-rise-nAb-Ancestral	0.586 [0.496, 0.670]
BRF + Fold-rise-nAb-BA.1	0.582 [0.493, 0.666]
BRF + BD1-bAb-Spike-Ancestral + BD29-bAb-Spike-Ancestral	0.579 [0.490, 0.664]
BRF + BD29-bAb-Spike-Ancestral	0.579 [0.489, 0.664]
BRF + BD1-bAb-Spike-BA.1 + BD1-bAb-Spike-Ancestral + Fold-rise-bAb-Spike-BA.1 + Fold-rise-bAb-Spike-Ancestral	0.579 [0.490, 0.664]
BRF + Fold-rise-bAb-Spike-BA.1 + Fold-rise-bAb-Spike-Ancestral	0.578 [0.488, 0.663]
BRF + Fold-rise-bAb-Spike-Ancestral + Fold-rise-nAb-Ancestral	0.575 [0.484, 0.661]
BRF + Fold-rise-nAb-Ancestral	0.573 [0.482, 0.660]
BRF + BD1-bAb-Spike-Ancestral + Fold-rise-bAb-Spike-Ancestral	0.573 [0.483, 0.657]
BRF + Fold-rise-bAb-RBD-Ancestral	0.570 [0.481, 0.655]
BRF + Fold-rise-bAb-Spike-BA.1	0.565 [0.475, 0.650]
BRF + Fold-rise-bAb-Spike-Ancestral	0.540 [0.451, 0.627]
BRF + BD1-bAb-Spike-Ancestral + BD1-nAb-Ancestral	0.534 [0.438, 0.627]
BRF + BD1-nAb-Ancestral	0.527 [0.432, 0.620]
BRF + BD1-bAb-Spike-BA.1 + BD1-bAb-Spike-Ancestral + BD1-bAb-RBD-Ancestral	0.524 [0.417, 0.629]
BRF + BD1-bAb-Spike-Ancestral + BD1-bAb-RBD-Ancestral	0.523 [0.416, 0.629]
BRF + BD1-bAb-Spike-BA.1	0.522 [0.415, 0.628]

BRF + BD1-bAb-RBD-Ancestral + BD1-nAb-Ancestral	0.522 [0.424, 0.618]
BRF + BD1-bAb-Spike-BA.1 + BD1-bAb-Spike-Ancestral	0.518 [0.406, 0.630]
BRF + BD1-bAb-Spike-Ancestral	0.518 [0.405, 0.629]
BRF + BD1-bAb-RBD-Ancestral	0.517 [0.404, 0.629]
BRF (baseline risk factors)	0.517 [0.404, 0.628]

Supplementary Table 11. Discrete Super Learner performance across all 92 variable sets sorted by cross-validated area under the ROC (CV-AUC) performance for predicting occurrence of Omicron COVID-19 in non-naive per-protocol boosted participants. bAb = binding antibody; BRF = baseline risk factors; fold-rise = BD29/BD1; nAb = neutralizing antibody.

Variable Set	CV-AUC (95% CI)
BRF + BD29-bAb-RBD-Ancestral + BD29-nAb-Ancestral	0.712 [0.558, 0.829]
BRF + Fold-rise-bAb-RBD-Ancestral + Fold-rise-nAb-Ancestral	0.683 [0.530, 0.804]
BRF + BD1-bAb-RBD-Ancestral + BD1-nAb-Ancestral + Fold-rise-bAb-RBD-Ancestral + Fold-rise-nAb-Ancestral	0.681 [0.526, 0.804]
BRF + Fold-rise-bAb-RBD-Ancestral + Fold-rise-nAb-BA.1 + Fold-rise-nAb-Ancestral	0.680 [0.527, 0.802]
BRF + BD1-bAb-RBD-Ancestral + BD1-nAb-BA.1 + BD1-nAb-Ancestral + Fold-rise-bAb-RBD-Ancestral + Fold-rise-nAb-BA.1 + Fold-rise-nAb-Ancestral	0.673 [0.516, 0.799]
BRF + BD1-bAb-Spike-Ancestral + BD1-bAb-RBD-Ancestral + BD1-nAb-Ancestral + Fold-rise-bAb-Spike-Ancestral + Fold-rise-bAb-RBD-Ancestral + Fold-rise-nAb-Ancestral	0.666 [0.510, 0.792]
BRF + BD1-bAb-Spike-Ancestral + BD1-bAb-RBD-Ancestral + Fold-rise-bAb-Spike-Ancestral + Fold-rise-bAb-RBD-Ancestral	0.657 [0.499, 0.787]
BRF + BD29-bAb-Spike-Ancestral + BD29-nAb-Ancestral	0.656 [0.500, 0.784]
BRF + Fold-rise-bAb-Spike-Ancestral + Fold-rise-bAb-RBD-Ancestral	0.654 [0.497, 0.784]
BRF + Fold-rise-bAb-RBD-Ancestral	0.651 [0.496, 0.780]
BRF + BD1-bAb-RBD-Ancestral + Fold-rise-bAb-RBD-Ancestral	0.650 [0.493, 0.780]
BRF + BD1-bAb-Spike-BA.1 + BD1-bAb-Spike-Ancestral + BD1-bAb-RBD-Ancestral + BD1-nAb-BA.1 + BD1-nAb-Ancestral + Fold-rise-bAb-Spike-BA.1 + Fold-rise-bAb-Spike-Ancestral + Fold-rise-bAb-RBD-Ancestral + Fold-rise-nAb-BA.1 + Fold-rise-nAb-Ancestral	0.646 [0.491, 0.775]
BRF + BD1-bAb-Spike-BA.1 + BD1-bAb-Spike-Ancestral + BD1-bAb-RBD-Ancestral + Fold-rise-bAb-Spike-BA.1 + Fold-rise-bAb-Spike-Ancestral + Fold-rise-bAb-RBD-Ancestral	0.643 [0.478, 0.782]
BRF + Fold-rise-bAb-Spike-BA.1 + Fold-rise-bAb-Spike-Ancestral + Fold-rise-bAb-RBD-Ancestral	0.640 [0.475, 0.780]
BRF + Fold-rise-bAb-Spike-Ancestral + Fold-rise-nAb-Ancestral	0.639 [0.481, 0.772]
BRF + BD1-bAb-RBD-Ancestral + BD1-nAb-Ancestral + BD29-bAb-RBD-Ancestral + BD29-nAb-Ancestral	0.636 [0.478, 0.770]
BRF + BD29-bAb-RBD-Ancestral + Fold-rise-bAb-RBD-Ancestral	0.633 [0.475, 0.767]
BRF + BD1-bAb-Spike-Ancestral + BD1-nAb-Ancestral + Fold-rise-bAb-Spike-Ancestral + Fold-rise-nAb-Ancestral	0.625 [0.468, 0.760]
BRF + Fold-rise-bAb-Spike-BA.1 + Fold-rise-bAb-Spike-Ancestral	0.624 [0.464, 0.761]
BRF + BD1-bAb-Spike-BA.1 + BD1-bAb-Spike-Ancestral + BD1-nAb-BA.1 + BD1-nAb-Ancestral + Fold-rise-bAb-Spike-BA.1 + Fold-rise-bAb-Spike-Ancestral + Fold-rise-nAb-BA.1 + Fold-rise-nAb-Ancestral	0.622 [0.463, 0.759]
BRF + Fold-rise-bAb-Spike-BA.1 + Fold-rise-bAb-Spike-Ancestral + Fold-rise-nAb-BA.1 + Fold-rise-nAb-Ancestral	0.622 [0.465, 0.756]
BRF + BD1-bAb-RBD-Ancestral + BD1-nAb-BA.1 + BD1-nAb-Ancestral	0.621 [0.465, 0.756]
BRF + BD1-nAb-Ancestral + Fold-rise-nAb-Ancestral	0.621 [0.466, 0.755]
BRF + BD1-bAb-Spike-BA.1 + BD1-bAb-Spike-Ancestral + Fold-rise-bAb-Spike-BA.1 + Fold-rise-bAb-Spike-Ancestral	0.619 [0.459, 0.757]
BRF + BD29-bAb-RBD-Ancestral + BD29-nAb-Ancestral + Fold-rise-bAb-RBD-Ancestral + Fold-rise-nAb-Ancestral	0.619 [0.460, 0.756]

BRF + BD1-nAb-BA.1 + BD1-nAb-Ancestral	0.617 [0.460, 0.753]
BRF + BD29-bAb-RBD-Ancestral + BD29-nAb-BA.1 + BD29-nAb-Ancestral	0.616 [0.460, 0.752]
BRF + BD29-bAb-Spike-Ancestral + BD29-bAb-RBD-Ancestral + Fold-rise-bAb-Spike-Ancestral + Fold-rise-bAb-RBD-Ancestral	0.615 [0.455, 0.753]
BRF + Fold-rise-bAb-Spike-Ancestral	0.614 [0.457, 0.751]
BRF + BD29-bAb-Spike-Ancestral + BD29-bAb-RBD-Ancestral + BD29-nAb-Ancestral + Fold-rise-bAb-Spike-Ancestral + Fold-rise-bAb-RBD-Ancestral + Fold-rise-nAb-Ancestral	0.614 [0.456, 0.751]
BRF + Fold-rise-bAb-Spike-BA.1	0.611 [0.454, 0.748]
BRF + BD1-bAb-Spike-Ancestral + Fold-rise-bAb-Spike-Ancestral	0.609 [0.452, 0.747]
BRF + BD29-nAb-BA.1 + BD29-nAb-Ancestral	0.607 [0.443, 0.752]
BRF + BD1-bAb-Spike-BA.1 + Fold-rise-bAb-Spike-BA.1	0.606 [0.452, 0.741]
BRF + BD1-bAb-RBD-Ancestral + BD1-nAb-Ancestral	0.603 [0.443, 0.744]
BRF + BD1-bAb-RBD-Ancestral + BD29-bAb-RBD-Ancestral	0.603 [0.444, 0.742]
BRF + BD1-nAb-BA.1 + BD1-nAb-Ancestral + Fold-rise-nAb-BA.1 + Fold-rise-nAb-Ancestral	0.602 [0.443, 0.742]
BRF + BD1-bAb-Spike-Ancestral + BD1-nAb-Ancestral + BD29-bAb-Spike-Ancestral + BD29-nAb-Ancestral	0.601 [0.444, 0.738]
BRF + BD1-bAb-Spike-BA.1 + BD1-bAb-Spike-Ancestral + BD1-nAb-BA.1 + BD1-nAb-Ancestral	0.599 [0.440, 0.739]
BRF + Fold-rise-bAb-Spike-BA.1 + Fold-rise-nAb-BA.1	0.598 [0.441, 0.736]
BRF + Fold-rise-nAb-BA.1 + Fold-rise-nAb-Ancestral	0.597 [0.423, 0.752]
BRF + BD29-bAb-Spike-BA.1 + BD29-bAb-Spike-Ancestral + BD29-bAb-RBD-Ancestral	0.596 [0.441, 0.734]
BRF + BD29-bAb-Spike-Ancestral + Fold-rise-bAb-Spike-Ancestral	0.595 [0.427, 0.745]
BRF + BD29-bAb-Spike-Ancestral + BD29-nAb-Ancestral + Fold-rise-bAb-Spike-Ancestral + Fold-rise-nAb-Ancestral	0.595 [0.415, 0.756]
BRF + BD1-bAb-Spike-Ancestral + BD1-bAb-RBD-Ancestral + BD1-nAb-Ancestral + BD29-bAb-Spike-Ancestral + BD29-bAb-RBD-Ancestral + BD29-nAb-Ancestral	0.594 [0.434, 0.737]
BRF + BD1-bAb-Spike-BA.1 + BD1-nAb-BA.1 + Fold-rise-bAb-Spike-BA.1 + Fold-rise-nAb-BA.1	0.593 [0.438, 0.731]
BRF + BD1-bAb-Spike-Ancestral + BD1-nAb-Ancestral	0.591 [0.432, 0.732]
BRF + BD1-bAb-RBD-Ancestral + BD1-nAb-BA.1 + BD1-nAb-Ancestral + BD29-bAb-RBD-Ancestral + BD29-nAb-BA.1 + BD29-nAb-Ancestral	0.590 [0.432, 0.732]
BRF + BD29-bAb-RBD-Ancestral + BD29-nAb-BA.1 + BD29-nAb-Ancestral + Fold-rise-bAb-RBD-Ancestral + Fold-rise-nAb-BA.1 + Fold-rise-nAb-Ancestral	0.590 [0.434, 0.730]
BRF + BD29-bAb-Spike-BA.1 + BD29-bAb-Spike-Ancestral + BD29-bAb-RBD-Ancestral + Fold-rise-bAb-Spike-BA.1 + Fold-rise-bAb-Spike-Ancestral + Fold-rise-bAb-RBD-Ancestral	0.590 [0.432, 0.731]
BRF + BD1-nAb-BA.1 + BD1-nAb-Ancestral + BD29-nAb-BA.1 + BD29-nAb-Ancestral	0.590 [0.425, 0.737]
BRF + BD1-nAb-Ancestral + BD29-nAb-Ancestral	0.586 [0.423, 0.733]
BRF + Fold-rise-nAb-Ancestral	0.586 [0.428, 0.728]
BRF + BD29-bAb-Spike-BA.1 + BD29-bAb-Spike-Ancestral + Fold-rise-bAb-Spike-BA.1 + Fold-rise-bAb-Spike-Ancestral	0.585 [0.409, 0.745]
BRF + BD1-bAb-Spike-Ancestral + BD29-bAb-Spike-Ancestral	0.584 [0.419, 0.733]
BRF + BD29-bAb-Spike-BA.1 + Fold-rise-bAb-Spike-BA.1	0.584 [0.415, 0.736]
BRF + BD1-bAb-Spike-BA.1 + BD1-bAb-Spike-Ancestral + BD1-bAb-RBD-Ancestral + BD29-bAb-Spike-BA.1 + BD29-bAb-Spike-Ancestral + BD29-bAb-RBD-Ancestral	0.583 [0.418, 0.733]
BRF + BD1-nAb-Ancestral	0.582 [0.426, 0.724]

BRF + BD29-bAb-Spike-Ancestral + BD29-bAb-RBD-Ancestral	0.580 [0.417, 0.729]
BRF + BD1-bAb-Spike-BA.1 + BD29-bAb-Spike-BA.1	0.580 [0.405, 0.739]
BRF + BD29-bAb-Spike-BA.1 + BD29-bAb-Spike-Ancestral + BD29-nAb-BA.1 + BD29-nAb-Ancestral	0.578 [0.418, 0.725]
BRF + BD29-bAb-RBD-Ancestral	0.577 [0.411, 0.728]
BRF + BD29-nAb-Ancestral + Fold-rise-nAb-Ancestral	0.576 [0.413, 0.725]
BRF + Fold-rise-nAb-BA.1	0.575 [0.408, 0.727]
BRF + BD1-bAb-Spike-Ancestral + BD1-bAb-RBD-Ancestral + BD29-bAb-Spike-Ancestral + BD29-bAb-RBD-Ancestral	0.574 [0.404, 0.731]
BRF + BD1-bAb-Spike-BA.1 + BD1-bAb-Spike-Ancestral	0.573 [0.407, 0.726]
BRF + BD1-bAb-Spike-BA.1 + BD1-bAb-Spike-Ancestral + BD1-bAb-RBD-Ancestral + BD1-nAb-BA.1 + BD1-nAb-Ancestral + BD29-bAb-Spike-BA.1 + BD29-bAb-Spike-Ancestral + BD29-bAb-RBD-Ancestral + BD29-nAb-BA.1 + BD29-nAb-Ancestral	0.573 [0.403, 0.728]
BRF + BD29-bAb-Spike-Ancestral	0.573 [0.410, 0.722]
BRF + BD1-bAb-Spike-Ancestral + BD1-bAb-RBD-Ancestral	0.570 [0.406, 0.721]
BRF + BD29-bAb-Spike-BA.1 + BD29-bAb-Spike-Ancestral + BD29-bAb-RBD-Ancestral + BD29-nAb-BA.1 + BD29-nAb-Ancestral + Fold-rise-bAb-Spike-BA.1 + Fold-rise-bAb-Spike-Ancestral + Fold-rise-bAb-RBD-Ancestral + Fold-rise-nAb-BA.1 + Fold-rise-nAb-Ancestral	0.569 [0.412, 0.713]
BRF + BD1-bAb-Spike-BA.1 + BD1-bAb-Spike-Ancestral + BD1-bAb-RBD-Ancestral + BD1-nAb-BA.1 + BD1-nAb-Ancestral + BD29-bAb-Spike-BA.1 + BD29-bAb-Spike-Ancestral + BD29-bAb-RBD-Ancestral + BD29-nAb-BA.1 + BD29-nAb-Ancestral + Fold-rise-bAb-Spike-BA.1 + Fold-rise-bAb-Spike-Ancestral + Fold-rise-bAb-RBD-Ancestral + Fold-rise-nAb-BA.1 + Fold-rise-nAb-Ancestral	0.569 [0.412, 0.713]
BRF + BD29-nAb-BA.1 + BD29-nAb-Ancestral + Fold-rise-nAb-BA.1 + Fold-rise-nAb-Ancestral	0.568 [0.380, 0.742]
BRF + BD1-nAb-BA.1 + Fold-rise-nAb-BA.1	0.567 [0.388, 0.733]
BRF + BD1-bAb-Spike-BA.1 + BD1-bAb-Spike-Ancestral + BD1-bAb-RBD-Ancestral	0.566 [0.402, 0.718]
BRF + BD29-bAb-Spike-BA.1 + BD29-nAb-BA.1 + Fold-rise-bAb-Spike-BA.1 + Fold-rise-nAb-BA.1	0.565 [0.390, 0.728]
BRF + BD1-bAb-Spike-BA.1 + BD1-bAb-Spike-Ancestral + BD29-bAb-Spike-BA.1 + BD29-bAb-Spike-Ancestral	0.565 [0.403, 0.715]
BRF + BD29-bAb-Spike-BA.1 + BD29-bAb-Spike-Ancestral	0.564 [0.404, 0.713]
BRF + BD1-bAb-Spike-BA.1	0.563 [0.391, 0.724]
BRF + BD1-bAb-Spike-Ancestral	0.563 [0.400, 0.715]
BRF + BD29-nAb-BA.1 + Fold-rise-nAb-BA.1	0.563 [0.393, 0.720]
BRF + BD1-bAb-Spike-BA.1 + BD1-bAb-Spike-Ancestral + BD1-nAb-BA.1 + BD1-nAb-Ancestral + BD29-bAb-Spike-BA.1 + BD29-bAb-Spike-Ancestral + BD29-nAb-BA.1 + BD29-nAb-Ancestral	0.562 [0.397, 0.716]
BRF + BD1-bAb-Spike-BA.1 + BD1-nAb-BA.1	0.562 [0.394, 0.718]
BRF + BD1-bAb-RBD-Ancestral	0.561 [0.398, 0.713]
BRF (baseline risk factors)	0.561 [0.389, 0.720]
BRF + BD29-bAb-Spike-BA.1	0.560 [0.397, 0.712]
BRF + BD29-nAb-BA.1	0.559 [0.389, 0.719]
BRF + BD1-bAb-Spike-BA.1 + BD1-nAb-BA.1 + BD29-bAb-Spike-BA.1 + BD29-nAb-BA.1	0.559 [0.386, 0.721]
BRF + BD29-nAb-Ancestral	0.558 [0.377, 0.727]
BRF + BD29-bAb-Spike-BA.1 + BD29-nAb-BA.1	0.557 [0.394, 0.710]

BRF + BD1-nAb-BA.1 + BD29-nAb-BA.1	0.557 [0.385, 0.718]
BRF + BD29-bAb-Spike-BA.1 + BD29-bAb-Spike-Ancestral + BD29-nAb-BA.1 + BD29-nAb-Ancestral + Fold-rise-bAb-Spike-BA.1 + Fold-rise-bAb-Spike-Ancestral + Fold-rise-nAb-BA.1 + Fold-rise-nAb-Ancestral	0.556 [0.370, 0.731]
BRF + BD1-nAb-BA.1	0.555 [0.384, 0.715]

Supplementary Table 12. Duke and PPD Assays Concordance Analysis: Descriptive statistics between Duke (ID50) and PPD (AU/mL) assays and by VOC (analysis sample subset: full analysis)

	D614G (n=250)		Omicron (BA.1) (n=250)	
**Parameters**	**Duke**	**PPD**	**Duke**	**PPD**
Geometric mean	2086.4	2739.3	639.8	324.4
SD (log 10 scale)	0.60	0.56	0.86	0.68
Geometric SD	3.98	3.59	7.28	4.79
Geometric CV	239.8%	203.5%	710.2%	325.6%
Median	2519.9	2986.5	816.5	408.5
Minimum	75.6	78.0	5	10
Maximum	88352.6	65395.0	99474.7	18761.0
LLOD	10	10	10	10
Percentage below the LLOD	0.0%	0.0%	1.2%	0.0%
ULOQ	45118	111433	15502.7	24503
Percentage above the ULOQ	1.6%	0.0%	4.0%	0.0%

CV, coefficient of variation; LLOD, lower limit of detection; SD, standard deviation; ULOQ, upper limit of quantitation.

Supplementary Table 13. Duke and PPD Assays Concordance Analysis: Correlations Between Duke (ID50) and PPD (AU/mL) Assays (Analysis Sample Subset: Full Analysis Set; N = 250)

VOC	Pearson Correlation* (95% CI)	Spearman Correlation (95% CI)	Raw CCC* (95% CI)	Calted CCC* (95% CI)	Meet concordance Criteria**
**D614G**	0.93 (0.911, 0.945)	0.92 (0.900, 0.944)	0.91 (0.885, 0.927)	0.93 (0.909, 0.943)	Yes
**Omicron (BA.1)**	0.95 (0.931, 0.957)	0.95 (0.935, 0.964)	0.85 (0.819, 0.875)	0.94 (0.925, 0.953)	Yes

*Pearson and CCC are based on log-transformed titers excluding 3 cases having < LLOD

Calted CCC = Calibrated CCC by linear regression approach.

**The predefined concordance criterion is lower bound of the 95% CI for CCC is greater than 0.65.

CCC, concordance correlation coefficient; CI, confidence interval; LLOD, lower limit of detection; SD, standard deviation; ULOQ, upper limit of quantitation.

Supplementary Table 14: Linear Regression and Deming Regression of PPD (AU/mL) on Duke (ID50) by VOC

	Linear Regression		Deming Regression by Wicklin 2019 with lambda = 1	
**VOC**	**Intercept (SE)**	**Slope (SE)**	**Intercept (95% CI)**	**Slope (95% CI)**
**D614G**	-0.095 (0.087)	0.993 (0.025)	-0.371 (-0.560, -0.182)	1.073 (1.019, 1.127)
**Omicron (BA.1)**	-0.103 (0.066)	1.156 (0.025)	-0.303 (-0.419, -0.188)	1.235 (1.186, 1.284)

Supplementary Fig. 36. Scatter Plots of Duke (ID50) and PPD (AU/mL) Assays by VOC with and without Calibration* (N = 250)

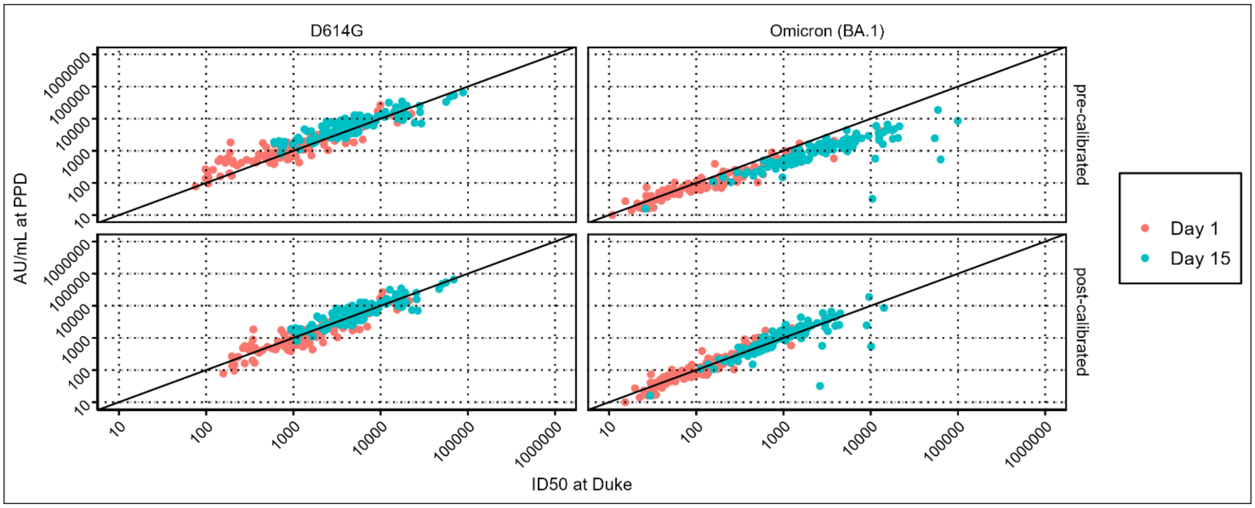

Supplementary Fig. 37. Violin Plots of Duke (ID50) and PPD (AU/mL) Assays by VOC with and without Calibration* (N = 250)

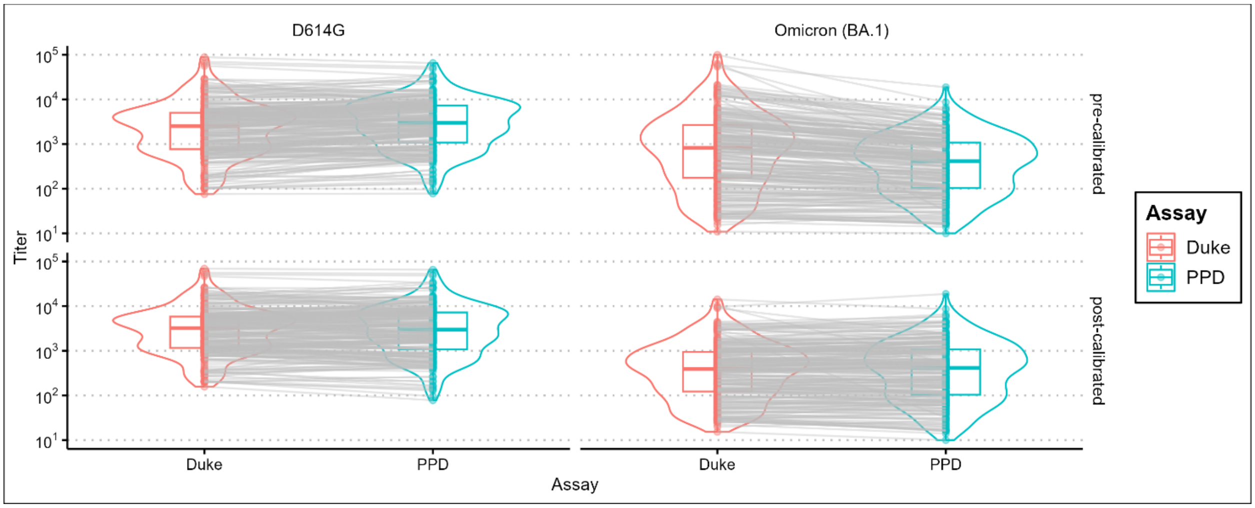

*The data was calibrated using linear regression approach, see Supplementary Table 14 for the calibration equation parameters. Only samples with titers above the lower limit of detection (LLOD) from both laboratories are included in plots and above ULOQ values were changed to be equal to ULOQ. VOC: Variant of concern.

Supplementary Table 15. Glossary of Terms, Abbreviations, and Acronyms

Term	Definition/Explanation/Further Information
AU	Arbitrary units, i.e., assay readouts that are not calibrated to any standard or scale.
bAb	Binding antibodies
BD1	Booster Dose 1 = the day on which the third (booster) dose of mRNA-1273 was administered. See Supplementary Fig. 1.
BD29	Booster Dose 29 = 28 days (allowable window: 19 to 45 days) post-administration of third (booster) dose. See Supplementary Figs. 1, 2.
CoP	Correlate of Protection = an immune marker that statistically associates with post-vaccination protection against a given clinical endpoint. May or may not be a mechanistic CoP. A validated CoP can expedite decisions on vaccine approval and use and hence is highly sought. Further reading on CoPs: refs.[1-4]
CoR	Correlate of Risk = an immune marker that statistically associates with the incidence of a given clinical endpoint in the population of interest receiving a regimen.[5,6] Assessment of CoRs is a first step in identifying a correlate of protection (CoP).
Correlate of booster relative efficacy	Given crossover of the original placebo arm, correlates could not be assessed against a placebo group. The correlate of booster relative efficacy analyses therefore contrast the hazard of Omicron COVID-19 in three-dose vaccine to that in two-dose vaccine recipients.
DD1	Disease Day 1 = Date of Omicron COVID-19 endpoint diagnosis. See Supplementary Fig. 1.
IU	International Units for neutralizing antibody titer. Ancestral nAb titer readouts can be calibrated to the WHO 20/136 International Standard[7] to express in IU (for 50% inhibitory dilution nAb titers, this is IU50/ml) as detailed in the Supplementary Material of Gilbert et al.[8]
LLOQ	Lower limit of quantitation. The LLOQ defines the lower readout of the range over which the assay is accurate and precisely quantitates samples.
nAb	Neutralizing antibodies. Evidence supporting nAbs as a CoP against symptomatic COVID-19 is summarized in ref.[9]
PDV	Participant decision visit. After issuance of an EUA for the mRNA-1273 vaccine (December 2020), the COVE protocol was amended to include an open-label phase. At the PDV, participants were informed of their original treatment assignment and those in the placebo group were offered the opportunity to receive mRNA-1273.
Predicted-at-exposure antibody level	To estimate Ab levels at each day post peak, paired antibody measurements on BD1 and DD1 were used to calculate the slope of antibody waning for each participant. The median slope was used to predict antibody levels at each following day, for each participant with BD29 measurement, using the formula described in Methods. The Ab

	level at each following day was then associated with the COVID-19 hazard using a calendar time-based Cox model.
Surrogate endpoint	A validated CoP can be used as a surrogate endpoint for a clinical outcome of interest and thus be used as a primary endpoint for vaccine authorization or approval. The use of a surrogate endpoint can thus circumvent resource-intensive randomized, controlled phase 3 trials. Example applications could include serving as a basis for approval of a vaccine in populations not included in the original phase 3 trial (e.g. children,[10] the elderly) or for approving e.g. variant-adapted versions.

Supplementary References

1 Gilbert, P. B. *et al.* Four statistical frameworks for assessing an immune correlate of protection (surrogate endpoint) from a randomized, controlled, vaccine efficacy trial. *Vaccine* **42**, 2181-2190, doi:10.1016/j.vaccine.2024.02.071 (2024).

2 Plotkin, S. A. Correlates of protection induced by vaccination. *Clin Vaccine Immunol* **17**, 1055-1065, doi:10.1128/CVI.00131-10 (2010).

3 Plotkin, S. A. & Gilbert, P. B. Nomenclature for immune correlates of protection after vaccination. *Clin Infect Dis* **54**, 1615-1617, doi:10.1093/cid/cis238 (2012).

4 Plotkin, S. A. & Gilbert, P. B. Correlates of Protection. In: Vaccines, Eighth Edition, Editors Walter Orenstein, Paul Offit, Kathryn Edwards, Stanley Plotkin. Pages 35-40. Elsevier Inc., New York. (2022).

5 Qin, L., Gilbert, P. B., Corey, L., McElrath, M. J. & Self, S. G. A framework for assessing immunological correlates of protection in vaccine trials. *J Infect Dis* **196**, 1304-1312, doi:10.1086/522428 (2007).

6 Gilbert, P. B., Qin, L. & Self, S. G. Evaluating a surrogate endpoint at three levels, with application to vaccine development. *Stat Med* **27**, 4758-4778, doi:10.1002/sim.3122 (2008).

7 National Institute for Biological Standards and Control (NIBSC). Instructions for use of First WHO International Standard for anti-SARS-CoV-2 Immunoglobulin (Version 3.0, Dated 17/12/2020) NIBSC code: 20/136 https://www.nibsc.org/science_and_research/idd/cfar/covid-19_reagents.aspx Access date Jul 29, 2021.

8 Gilbert, P. B. *et al.* Immune correlates analysis of the mRNA-1273 COVID-19 vaccine efficacy clinical trial. *Science* **375**, 43-50, doi:10.1126/science.abm3425 (2022).

9 Gilbert, P. B. *et al.* A Covid-19 Milestone Attained — A Correlate of Protection for Vaccines. *New England Journal of Medicine* **387**, 2203-2206 (2022).

10 Fleming-Dutra, K. E. Interim recommendations of the Advisory Committee on Immunization Practices for use of Moderna and Pfizer-BioNTech COVID-19 vaccines in children aged 6 months–5 years—United States, June 2022. *MMWR. Morbidity and Mortality Weekly Report* **71** (2022).

Statistical Analysis Plan for Study of Post Dose 3 and Exposure-Proximal Omicron Antibody as Immune Correlates for Omicron COVID-19 in the P301 COVE Study

USG COVID-19 Response Team / Coronavirus Prevention Network (CoVPN) Biostatistics Team

October 15, 2023

Contents

List of Tables 3

List of Figures 4

1 Outline 5

2 Stage 1 correlates sampling design 5

3 Objectives of this post booster dose Omicron correlates study 5

4 Stage 2 sampling design for addressing the objectives 6

5 Statistical analysis plan by objectives 7

5.1 Descriptive statistics . . . 7
5.2 Details on planned figures and tables for the first manuscript . . . 14
5.3 Assessing Objectives 1–4 (≈peak Ab and pre-booster Correlates of Risk) . . . 15
5.3.1 Covariates adjusted for in CoR and CoP analyses . . . 16
5.3.2 Machine learning analysis to estimate best models for predicting COVID-19 . 16
5.4 Assessing Objectives 5 and 6 (≈peak Ab as controlled risk Correlates of Protection) 18
5.4.1 Primary controlled risk CoP analysis . . . 18
5.4.2 Exploratory controlled CoP analysis . . . 19
5.5 Controlled VE CoP analysis of Objectives 5 and 6 based on boosted vs. not-yet boosted . . . 20
5.6 Assessing Objectives 7-9 (Exposure-Proximal Correlates of Risk and Correlates of Protection) . . . 21
5.7 Addressing Objective 10 on mediation of the effect of dose 2 to 3 interval on COVID-19 mediated through BD29 antibody . . . 22

6 Specifications for general issues faced for most analyses 22

6.1 Computation of inverse probability of sampling weights . . . 22
6.2 Imputation of demographics variables for stratification and merging of sparse strata for weights computation . . . 23

7 Additional data analysis issues 23

7.1 Exclude participants reporting being HIV positive from the correlates analysis . . . 23
7.2 Missing lineages . . . 23

List of Tables

1 Stratified sampling design for measuring Omicron antibody at (BD1, BD29) for non-cases and at (BD1, BD29, DD1) for cases* 8

List of Figures

1 Flow-chart of the stage 2 correlates study that evaluates Omicron antibody as a correlates of risk and of protection of Omicron COVID-19. 7

1 Outline

First, this document recapitulates the sampling design that was used for assessment of Stage 1 correlates (Gilbert et al., 2022b). Second, it states the study objectives to assess post dose 3 Omicron BA.1 antibody titer, and exposure-proximal antibody titer, as immune correlates for Omicron COVID-19. Third, it describes the sampling plan for enabling the immune correlates statistical analyses. Fourth, it specifies the statistical analysis plan that details how to assess each objective.

Three assays VAC62, VAC122, VAC123 have been selected for this study:

VAC62– PsVNA against ancestral D614G strain (PPD Vaccines)

VAC122– PsVNA against BA.1 (B.1.529) VAC122 (PPD Vaccines)

VAC123– MSD multiplex: S, RBD, N +S (D614, Gamma, Alpha, Beta, Delta AY4, Omicron BA.1) (PPD Vaccines)

2 Stage 1 correlates sampling design

A two-phase stratified case-cohort sampling design was applied for measuring D1, 29, 57 antibody levels after the two-dose primary series in per-protocol participants sampled into the immunogenicity subcohort and for all baseline negative per-protocol vaccine recipient COVID-19 endpoint cases occurring at least 7 days post D29 visit or at least 7 days post D57 visit. The implemented sampling design is described in the Supplementary Material of Gilbert et al. (2022b). The sampling design sought balanced numbers of baseline negative per-protocol participants in each of the six demographic strata defined by (Minority, Non-Minority) $\times$ (Age ≥ 65, Age < 65 and 'at risk', Age < 65 and Not 'at risk'), within each of the naïve and non-naïve populations. For sampling of non-cases for Stage 2 correlates, balance in these factors will also be pursued.

3 Objectives of this post booster dose Omicron correlates study

The following objectives are assessed separately in SAR-CoV-2 naïve and SAR-CoV-2 non-naïve individuals, as defined below. The study endpoint for all objectives is adjudicated "Omicron COVID-19" counted starting 7 days after the post-booster Day 29 (BD29) visit and starting December 1, 2021 or later. For Objectives 7 and 9, "instantaneous Omicron COVID-19" refers to the instantaneous hazard rate of Omicron COVID-19, i.e., the rate of Omicron COVID-19 over the next day of follow-up. The objectives are assessed primarily for two BA.1 markers: bAb to Spike BA.1 in the MSD multiplex (VAC123), and pseudovirus nAb-ID50 titer to BA.1 (VAC122), based on assays at PPD. In addition, some of the objectives will be repeated for the same markers measured against D614 (binding assay) or D614G (pseudovirus neutralization) instead of against BA.1.

Objectives

1. To assess BD29 Omicron Ab as a correlate of risk (CoR) against Omicron COVID-19
2. To assess fold-rise in Omicron Ab from BD1/pre-booster to BD29 as a CoR against Omicron COVID-19

3. To assess whether the CoR in 1. or 2. is modified by SARS-CoV-2 naïve/non-naïve status
4. To assess whether the CoR in 1. is modified by the BD1 antibody value
5. To assess BD29 Omicron Ab as a correlate of protection (CoP) against Omicron COVID-19
6. To assess fold-rise in Omicron Ab from BD1 to BD29 as a CoP against Omicron COVID-19
7. To assess Omicron Ab as an exposure-proximal CoR of instantaneous Omicron COVID-19
8. To assess whether the exposure-proximal CoR in 7. is modified by the BD1 antibody value
9. To assess Omicron Ab as an exposure-proximal CoP against instantaneous Omicron COVID-19
10. To assess mediation of the effect of the interval between dose 2 and dose 3 on Omicron COVID-19 through BD29 Omicron Ab value

4 Stage 2 sampling design for addressing the objectives

Figure 1 shows the blood sampling schedule that enables the correlates studies. This correlates study is a stratified case-control study of post-dose 3 Omicron Ab in 3-dose vaccine recipients. The sampling approach samples Omicron COVID-19 endpoint cases starting 7 days after BD29 from each of the original vaccine and cross-over vaccine arms. Sampling stratified by randomization arm creates useful variability in the time between the two-dose vaccination series and the booster dose. The primary study endpoint is Omicron COVID-19 occurring at least 7 days post dose 3 Ab measurement at BD29 through to the data base lock in May, 2022. The sampling is done separately in the "naïve" cohort with no evidence of SARS-CoV-2 infection from enrollment through to BD1 and in the "non-naïve" cohort with any evidence of SARS-CoV-2 infection from ≥14 days after the second dose of mRNA-1273 vaccine through to BD1. Here, prior infection is defined inclusively based on any results of previous RT-PCR+, N-seroconversion, or a symptomatic COVID-19 endpoint with positive confirmatory testing. Stratifying the sampling by naïve/non-naïve status enables study of immune correlates in each of the naïve and non-naïve cohorts given the importance of understanding immune correlates in non-naïve populations as well as in naïve populations, and addressing whether and how prior infection modifies immune correlates (Objective 3). Figure 1 shows the schema of blood sample storage for potential antibody measurement in the COVE study.

The sample size of the correlates study in terms of participants with new antibody measurements is as follows:

1. Stratified random sample of N=256 three-dose vaccine recipients
2. 640 total samples/assays (Omicron BA.1 Ab measured at BD1, BD29 for all participants, and also at disease-day-one (DD1) that is the date of COVID-19 endpoint diagnosis for all cases)

Each of the correlates studies (in naïve and non-naïve individuals) is based on 64 vaccine cases with antibody data; in comparison ≈peak Ab correlates were defined based on 36 vaccine cases in the Stage 1 correlates analyses (Gilbert et al., 2022b) and exposure-proximal correlates were defined

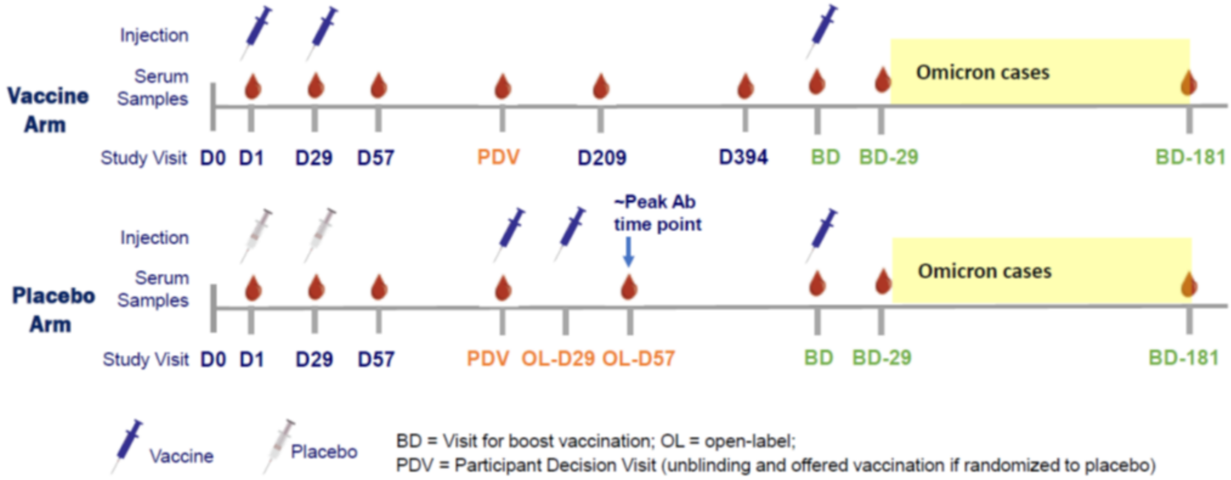

Figure 1: Flow-chart of the stage 2 correlates study that evaluates Omicron antibody as a correlates of risk and of protection of Omicron COVID-19.

based on 39 vaccine cases for an observational study of Pfizer's mRNA vaccine (Bergwerk et al., 2021).

Table 1 presents the sampling strata, where for eligibility participants must qualify per-protocol during the original follow-up period, received the first booster dose, have blood samples at BD1 and BD29, and cases are also required to have DD1 sample availability. Appendix A provides complete details of the Stage 2 sampling design that includes a prioritization of sampling of eligible participants and a dependency of sampling on demographic factors, which allows computation of inverse probability of sampling weights for all participants included in the correlates study.

For peak time correlates analyses of BD29 markers, in addition to requiring cases to have failure time starting 7 days after BD29 for inclusion, it is also required that the time interval between BD1 and BD29 falls in 19 to 45 days; otherwise the case is excluded from analysis.

5 Statistical analysis plan by objectives

5.1 Descriptive statistics

Tables of immunogenicity will be reported separately by assay, which amounts to the following variables:

1. $\log_{10}$ nAb titer to D614G
2. $\log_{10}$ nAb titer to BA.1
3. $\log_{10}$ anti-Spike IgG to D614

Table 1: Stratified sampling design for measuring Omicron antibody at (BD1, BD29) for non-cases and at (BD1, BD29, DD1) for cases*

	Boosted Sep23-Oct15 2021		Boosted Oct16-Oct31 2021		Boosted Nov 2021		Boosted Dec 2021		Total (Samples)
Original Vx Omicron case	8N	8NN	8N	8NN	8N	8NN	8N	8NN	64 (192)
Original Vx non-case	8N	8NN	8N	8NN	8N	8NN	8N	8NN	64 (192)
Crossover Vx Omicron case	8N	8NN	8N	8NN	8N	8NN	8N	8NN	64 (192)
Crossover Vx non-case	8N	8NN	8N	8NN	8N	8NN	8N	8NN	64 (192)

*Case = COVID-19 endpoint in the interval [$\geq$ 7 days post BD29 AND $\geq$ Dec 1 2021, May 2022 data base lock date]. As described in the appendix the COVID-19 endpoint is documented to be Omicron BA.1 if possible whereas for some non-naïve COVID-19 endpoints there was not lineage data available to document the case to be Omicron BA.1.
Non-case = Did not acquire COVID-19 (of any strain) in the interval [BD1, data base lock date].
naïve = No evidence of SARS-CoV-2 infection from enrollment through to BD1;
Non-naïve = Any evidence of SARS-CoV-2 infection in the interval [$\geq$ 14 days after the second dose of mRNA-1273, BD1], operationalized as a COVID-19 endpoint or seroconversion from a blood sample up to the BD1 visit (evidence of infection by RNA PCR from a BD1 sample was not included as a qualifier for the Non-naïve group).

4. $\log_{10}$ anti-Spike IgG to Gamma
5. $\log_{10}$ anti-Spike to Alpha
6. $\log_{10}$ anti-Spike to Beta
7. $\log_{10}$ anti-Spike to Delta AY4
8. $\log_{10}$ anti-Spike to BA.1
9. $\log_{10}$ anti-RBD IgG to D614

Note that while descriptives are provided for all of the assay variables, the correlates analyses focus on four assay variables: $\log_{10}$ nAb ID50 titer to D614G, $\log_{10}$ nAb ID50 titer to BA.1, anti-Spike IgG to D614, and anti-Spike IgG to BA.1.

Inverse-probability weighting will be used in summarizing immunogenicity in order that estimates and inferences are for the population from which the whole study cohort was drawn. This whole population and the sample weights are defined in Section 6.1.

Assay readouts accounting for assay limits (before multiplying the readouts by constants)

The antibody markers have readouts in units defined by PPD reports, and the readouts account for the LOD, LLOQ, ULOQ assay limits derived by PPD for each assay.

The readout for the analysis of the two nAb ID50 titer markers is serum antibody concentration Ab[C], with labeling for plots "ID50 (AU/ml)." For D614G, the LLOQ for Ab[C] is 10 AU/ml. For D614G the ULOQ for Ab[C] is 281,600 AU/ml. Values > ULOQ are assigned ULOQ. These LLOQs and ULOQs were derived for the D614G antigen in the PPD assay report "PPD Project

ID: RPJX. Assessment of Equivalency of Neutralization Antibodies Between the PPD VSDVAC 62 Microneutralization Assay and Historical ID50 Results Provided by Moderna That Were Generated Using the Duke Microneutralization Assay and the D614G Microneutralization Assay Version 1.0.” PPD amended the ULOQ of 281,600 AU/ml based on FDA feedback that precision, relative accuracy and dilutional linearity of the SARS CoV-2 MN assay as well as ULOQ for the assay be based on the highest measurable sample that shows acceptable precision and accuracy.

For the Omicron BA.1 antigen, the PPD assay report “PPD Project Code: RVUJ2. Validation of A Microneutralization Assay for the Detection of SARS CoV-2 Neutralizing Antibodies (SARS CoV-2-NAb) for the Omicron Spike BA.1 Variant in Human Serum (SARS CoV-2 MN O) Version 1.0” yielded the LLOQ for Ab[C] of 8 AU/ml and a ULOQ of 24,503 AU/ml. Values > ULOQ are assigned ULOQ.

The LLOQ for PsV nAb ID50 is 10 for D614G and 8 for BA.1, respectively. PsV nAb ID50 D614G values below LLOQ = 10 are assigned the value of LLOQ/2 = 5 and PsV nAb ID50 BA.1 values below LLOQ = 8 are assigned the value of LLOQ/2 = 4.

Multiplying nAb ID50 titer assay readouts by constants to place readouts on a comparable scale to units used in the previous immune correlates publications for Moderna COVE

The PPD assay report RPJX cited above showed that Ab[C] is on the same AU/ml scale as the Duke ID50 titer readout with no need for multiplying Ab[C] by a constant, that is, analyses would be acceptable if they treat Ab[C] to have the same unitage as the Duke ID50 biomarker. That report estimated a scaling factor of 1.04 between the PPD Ab[C] readout and the Duke ID50 readout, and therefore we do apply this scaling factor, even though it has little impact.

In addition, we multiply PPD Ab[C] readouts by 0.242, which was the conversion factor used by Duke to convert their ID50 readouts to the IU50/ml scale. It might seem better to divide the PPD Ab[C] readouts by 1.275, as this was the conversion factor estimated by PPD in its calibration report “PPD Project Code: RQHQ. Calibration of the V62RS-X132-CVMN Reference Standard Used in the SARS CoV-2 Neutralizing Antibodies (SARS CoV-2-Nab) in Human Serum (SARS CoV-2 MN) Method to the WHO International Standard for anti-SARS-CoV-2 Immunoglobulin Lot 20/136 Version 1.0.” However, to meet our greater objective to be able to compare readouts to those previously used in the blinded-phase Moderna COVE correlates studies (Gilbert et al., 2022b; Benkeser et al., 2023), we apply the Duke conversion factor. This means we can interpret anti-D614G ID50 titer readouts at BD1 and BD29 in the current correlates study on an apples vs. apples scale compared to the readouts used in the previous correlates studies. A section in the Supplemental Material of the booster correlates manuscript will explain the reasoning of this choice in greater detail. In sum, the original PPD Ab[C] ID50 readouts received from PPD are multiplied by 0.242 and then they are divided by 1.04, to constitute the reported ID50 (AU/ml) readouts. Then, the PPD anti-BA.1 Ab[C] ID50 readouts received from PPD are also multiplied by 0.242 and then they are divided by 1.04, for placing the readouts on a comparable scale to readouts against the D614G strain. Statistical reports are generated both using the un-scaled PPD assay units for each of D614G ID50 and BA.1 ID50, as well as using the scaled units for each of D614G ID50 and BA.1 ID50 (multiplying un-scaled readouts by 0.242/1.04).

In addition, it is of interest to consider D614G ID50 readouts scaled to be predicted ID50 values against BA.1; the advantage of doing this is the anchoring to the Duke/PPD D614G assay concordance study, as the Duke/PPD BA.1 concordance study is still ongoing. Based on data from the Duke assay on 26 3-dose mRNA-1273 participants, the geometric mean ratio of ID50 readouts to BA.1 vs. to D614G was 0.225. Therefore, it is of interest to scale D614G readouts by multiplying them by 0.225, which gives the readouts interpretations in terms of predicted ID50 against BA.1. Multiplying original PPD D614G ID50 units by (0.242/1.04)*0.225 = 0.052 creates the Predicted BA.1 units that can be quantitatively interpreted in comparison to the anti-D614G IU50/ml units, where for example a result of Predicted BA.1 ID50 is 2-fold lower than D614G IU50/ml can be properly interpreted as 2-fold lower titer against BA.1 than against D614G. The following data analysis will be included:

The blinded phase correlates study (Gilbert et al. 2022) estimated how two-dose vs. placebo vaccine efficacy varied by D614G nAb ID50 titer at 4 weeks post dose 2, with ID50 titer calibrated to the WHO 20/136 International Standard and reported in IU50/ml units. It is of interest to compare this ancestral antibody, ancestral COVID-19 vaccine efficacy curve with the BA.1 antibody, Omicron BA.1 COVID-19 booster vaccine efficacy curve, to ascertain whether a different amount of variant-matched antibody is needed for high-level booster protection than for high-level two dose vs. placebo protection. To do this, we defined a Predicted BA.1 ID50 biomarker at BD29 scaled such that it can be absolutely quantitatively interpreted vs. D614G IU50/ml units. This scaling was accomplished in two steps. First, the Duke/PPD D614G assay concordance study (BARDA, 2021) and the Duke assay International Standard calibration study (Huang et al., 2021) showed that multiplying D614G PPD nAb ID50 readouts by (0.242/1.04) transforms units to the IU50/ml scale previously used (Gilbert et al., 2022c). Second, based on data from 26 three-dose mRNA-1273 participants with Duke assay ID50 measured 4 weeks post dose 3 against both D614G and BA.1, the geometric mean ratio of ID50 against BA.1 vs. against D614G was 0.225 (Lyke et al., 2022; Atmar et al., 2022). Therefore, we multiplied the PPD D614G nAb IU50/ml values by 0.225, attaining the Predicted BA.1 ID50 values (thus original PPD BA.1 ID50 units are multiplied by (0.242/1.04)*0.225 = 0.052 to generate Predicted BA.1 ID50 units). The BD29 booster vaccine efficacy curve analysis was repeated for this biomarker, and results overlaid with the original Day 57 vaccine efficacy curve analysis, providing a means for absolute comparison of variant-matched titer levels associated with efficacy.

Note that for plotting labeling, IU50/ml and BAU/ml labeling is never used, because antibody responses to BA.1 are of primary interest, and international units do not exist for these readouts. For the responses against D614G or D614 the readouts indeed are in international units IU50/ml and BAU/ml; however, for consistency with BA.1 readouts plotting labels AU/ml are used, and footnotes of captions note that these units equate to international units.

The assay limits for the PPD VAC123 MSD multiplex assay are listed below. In particular, the LLOQs are taken as the LLOQs for the lowest dilution 1:500, and are as follows by antigen:

- Spike D614: 69
- B.1.1.529/BA.1: 102
- B.1.617.2/Delta: 150

- P.1/Gamma: 143
- B.1.1.7/Alpha: 52
- B.1.351/Beta: 111
- RBD D614: 79

In addition, the ULOQs are taken as the ULOQs for the highest dilution 1:500,000, and are as follows by antigen:

- Spike D614: 14,400,000
- B.1.1.529/BA.1: 1,180,000
- B.1.617.2/Delta: 8,000,000
- P.1/Gamma: 5,800,000
- B.1.1.7/Alpha: 8,800,000
- B.1.351/Beta: 5,000,000
- RBD D614: 5,800,000

The LLOQ for bAb Spike is 69 AU/ml for D614 and 102 AU/ml for BA.1. bAb spike D614 readouts below LLOQ = 69 AU/ml are assigned the value LLOQ/2 = 34.5 AU/ml, and bAb spike BA.1 readouts below LLOQ = 102 AU/ml are assigned the value LLOQ/2 = 51 AU/ml.

Reporting units for MSD binding antibody readouts in tables and figures are AU/ml. PPD did not develop a conversion factor from AU/mL to International Units (BAU/ml) for any of the MSD assays. There is no equivalency study of the PPD VAC123 MSD assay compared to the VRC MSD assay that was used in the first correlates study Gilbert et al. (2022b).

Definition of participants with a positive response

- Participants with a positive (quantifiable) pseudovirus neutralization response at each pre-defined timepoint are defined as participants who had ID50 value at the time point greater than or equal to the antigen-specific LLOQ; otherwise the response is not detectable. This definition is the same for both nAb D614G and nAb BA.1.
- Participants with a positive antigen-specific binding antibody response at each pre-defined timepoint are defined as participants who had a antigen-specific bAb measurement at the time point greater than or equal to the antigen-specific LLOQ (specified above); otherwise the response is negative.

Tabular output

- Average duration of follow-up post BD29 for cases and non-cases, stratified by naïve/non-naïve status
- Number (%) positive responses (including denominator that is the estimated number of participants in the population in the cell) with 95% CI at each time point (columns) by original randomization arm x case-control status x naïve/non-naïve status (rows). 95% CI calculated

based on Clopper-Pearson method. Table pools participants over the four boosting intervals listed in Table 1. The time points are BD1, BD29, and disease-day 1 (DD1) (only cases are included for DD1).

- Number (%) positive responses with 95% CI at BD1 by boosting interval (columns) and original randomization arm x case-control status x naïve/non-naïve status (rows). 95% CI calculated based on Clopper-Pearson method.
- Number (%) positive responses with 95% CI at BD29 by boosting interval (columns) and original randomization arm x case-control status x naïve/non-naïve status (rows). 95% CI calculated based on Clopper-Pearson method.
- Number (%) positive responses with 95% CI at DD1 by boosting interval (columns) and original randomization arm x case-control status x naïve/non-naïve status (rows). 95% CI calculated based on Clopper-Pearson method.
- Geometric mean (95% CI) of quantitative marker at each time point (columns) by original randomization arm x case-control status x naïve/non-naïve status (rows). 95% CIs using the t-distribution approximation of $\log_{10}$-transformed marker (base 10 of the logarithm is always used). Table pools participants over boosting interval. The time points are BD1, BD29, and DD1 (only cases are included for DD1).
- Geometric mean (95% CI) of quantitative marker at BD1 by boosting interval (columns) and original randomization arm x case-control status x naïve/non-naïve status (rows). 95% CIs using the t-distribution approximation of log-transformed marker
- Geometric mean (95% CI) of quantitative marker at BD29 by boosting interval (columns) and original randomization arm x case-control status x naïve/non-naïve status (rows). 95% CIs using the t-distribution approximation of log-transformed marker
- Geometric mean (95% CI) of quantitative marker at DD1 by boosting interval (columns) and original randomization arm x case-control status x naïve/non-naïve status (rows). 95% CIs using the t-distribution approximation of log-transformed marker.
- Geometric mean ratio (95% CI) of quantitative marker at BD29 and DD1 time points relative to BD1 time point (columns) by original randomization arm x case-control status x naïve/non-naïve status (rows). (i.e., geometric mean of fold-rise values from BD1 to BD29 and from BD1 to DD1.) 95% CIs using the t-distribution approximation of log-transformed marker at each time point. Table pools participants over boosting interval.
- Geometric mean ratio (95% CI) of quantitative marker at BD29 relative to BD1 time point by boosting interval (columns) and original randomization arm x case-control status x naïve/non-naïve status (rows). 95% CIs using the t-distribution approximation of log-transformed marker.
- Geometric mean ratio (95% CI) of quantitative marker at DD1 relative to BD1 time point by boosting interval (columns) and original randomization arm x case-control status x naïve/non-naïve status (rows). 95% CIs using the t-distribution approximation of log-transformed marker.

- Differences in positive response rates (95% CI) between cases and controls at each time point (columns) by original randomization arm x naïve/non-naïve status (rows). 95% CI the Wilson-Score method without continuity correction (Newcombe, 1998). Table pools participants over boosting interval. The time points are BD1 and BD29.
- Differences in positive response rates (95% CI) between cases and controls at BD1 by boosting interval (columns) and original randomization arm x naïve/non-naïve status (rows). 95% CI the Wilson-Score method without continuity correction (Newcombe, 1998).
- Differences in positive response rates (95% CI) between cases and controls at BD29 by boosting interval (columns) and original randomization arm x naïve/non-naïve status (rows). 95% CI the Wilson-Score method without continuity correction (Newcombe, 1998).
- Geometric mean ratio (95% CI) of quantitative marker between cases and controls at each time point (column) by original randomization arm x naïve/non-naïve status (rows). Table pools participants over boosting interval. The time points are BD1 and BD29.
- Geometric mean ratio (95% CI) of quantitative marker between cases and controls at BD1 by boosting interval (columns) and original randomization arm x naïve/non-naïve status (rows).
- Geometric mean ratio (95% CI) of quantitative marker between cases and controls at BD29 by boosting interval (columns) and original randomization arm x naïve/non-naïve status (rows).

Graphical Output

Set 1 plots: BD1 and BD29 Ab distributions by case/non-case and naive-non-naive status

1. BD1 antibody for the 2 $\log_{10}$ nAb ID50 titer markers (to D614G and to BA.1), 8 panels of violin/boxplots defined by 4 rows (cross-classification of original randomization arm with naïve/non-naïve) and 2 columns defined by D614G and BA.1 strain, where within each panel there are side-by-side violin/boxplots for cases and non-cases. These plots pool over the four boosting intervals.
2. Repeat 1. for BD29 antibody
3. Repeat 1. for BD29 - BD1 antibody
4. Repeat 1.–3. for the 2 $\log_{10}$ IgG anti-Spike markers (to D614 and to BA.1)
5. Repeat 1.–3. for the 2 $\log_{10}$ IgG anti-RBD markers (to D614)
6. BD1 antibody for the 6 $\log_{10}$ IgG anti-Spike markers (to D614, Gamma, Alpha, Beta, Delta AY4, BA.1), 24 panels of violin/boxplots defined by 4 rows (cross-classification of original randomization arm with naïve/non-naïve) and 6 columns defined by strain, where within each panel there are side-by-side violin/boxplots for cases and non-cases. These plots pool over boosting intervals.
7. Repeat 7. for BD29 antibody
8. Repeat 7. for BD29 - BD1 antibody

Set 2 plots: Longitudinal plots BD1 to BD29 (and to DD1)

1. For $\log_{10}$ nAb ID50 titer to D614G, for each of 4 rows (cross-classification of original randomization arm with naïve/non-naïve), plot 5 side-by-side violin/box plots, the the first 2 for BD1 non-cases, BD29 non-cases, with lines connecting individual's data points, and the last 3 for BD1 cases, BD29 cases, DD1 cases, with lines connecting individual's data points. To the right of this plot, place the parallel results for $\log_{10}$ nAb ID50 titer to BA.1. These plots pool over the four boosting intervals.
2. Repeat 1. for $\log_{10}$ anti-Spike IgG (for D614 and BA.1)
3. Repeat 1. for $\log_{10}$ anti-RBD IgG (for D614)

Set 3 plots: Correlation plots across markers at a given time point

1. For all 15 markers at BD1, a pairs plot similar to those in Gilbert et al. (2022b), pooling over boosting intervals, original randomized arm, case/non-case status, and naïve/non-naïve status. Spearman rank correlation coefficients are included (including IPS weights).
2. Repeat 1. for the 15 markers at BD29
3. Repeat 1. for the 15 difference markers BD29 - BD1 (i.e., $\log_{10}$ fold-rise markers)
4. Repeat 1.–3. restricting to the 6 markers of focus as defined in Section 5.1.

Set 4 plots: Correlation plots for a given marker across time points

1. For each of the 15 markers, a figure with 8 panels, with 4 rows (cross-classification of original randomization arm with naïve/non-naïve) of pairs plots, pooling over boosting intervals, for the marker measured over the time points BD1 and BD29 for non-cases (column 1) and over BD1, BD29, and DD1 for cases (column 2). Spearman rank correlation coefficients are included.

5.2 Details on planned figures and tables for the first manuscript

Proposed Figure 1 of the manuscript: Include the nAb ID50 BA.1 marker and the IgG Spike BA.1 marker. 8 panels, 2 rows, 4 columns. Each panel shows the violin plots for BD1 and BD29 marker distributions, with lines connecting the BD1 and BD29 data points so the paired data/fold-rises are visible. The 2 rows are for (1) nAb marker and for (2) IgG Spike (the logic here is the y-axis range can always be the same). The 4 columns are for (1) Naive Omicron Cases; (2) Naive Non-Cases; (3) Non-naive Omicron Cases; (4) Non-naive Non-Cases Plotting symbols distinguish Original-Vaccine and Crossover-Vaccine.

Then a supp figure would do the same thing for nAb ID50 D614G and IgG Spike D614. And 2 other supp figures would do the same thing except replace BD29 marker with Fold-rise marker.

Proposed Table 1 of the manuscript: Like Table 1 in the 2022 Science paper, for the same 2 BA.1 markers of Figure 1 (nAb ID50, IgG Spike), focusing only on the BD29 time point, showing BD29 absolute level markers and fold-rise markers as separate rows, and separately for Naive and Non-Naive. So the rows would be (1) Naive, ID50 BA.1, BD29; (2) Naive, IgG Spike BA.1, BD29; (3) Naive, ID50 BA.1, Fold-rise; (4) Naive, IgG Spike BA.1, Fold-rise; (5) Non-Naive, ID50 BA.1,

BD29; (6) Non-Naive, IgG Spike BA.1, BD29; (7) Non-Naive, ID50 BA.1, Fold-rise; (8) Non-Naive, IgG Spike BA.1, Fold-rise.

Then a supp table that is the same except it is for nAb ID50 against D614G and IgG Spike against D614.

Proposed Figure 2 of the manuscript: Of the 'identical' structure/layout of Figure 2 in the 2022 Science paper, with Panel A for Naive, ID50 BA.1, BD29 and Panel B for Non-Naive, ID50 BA.1, BD29. Panel C would include results for 8 markers, the same 8 listed above for Table 1.

5.3 Assessing Objectives 1–4 (≈peak Ab and pre-booster Correlates of Risk)

For the CoR Objectives 1.–4., the planned analysis is similar to the originally published Stage 1 CoR analysis, implementing baseline-covariate marginalized Cox regression and nonparametric monotone-constrained analysis in the stratified random sample of three-dose vaccine recipients, who were per-protocol during the original follow-up period, received the first booster dose, and have blood samples at BD1, BD29, and also at DD1 for cases. The Cox regression modeling is done using study time to be consistent with what was done originally for COVE; this approach could have reduced precision compared to using calendar time if calendar time predicts COVID-19. If calendar time does strongly predict COVID-19, the analyses may be repeated using the calendar time scale. Cox modeling for CoP objectives uses the calendar time scale (see Section 5.5).

For analyses of markers defined at BD29, the Cox model uses BD29 as the time origin, whereas for analyses of markers defined at BD1, the Cox model uses BD1 as the time origin. Specifically, output for the six analyzed markers listed in Section 5.1 is as follows, where the analyses are done separately for the naïve and non-naïve cohorts, as well as for pooling over the naïve and non-naïve cohorts.

1. (Obj. 1,2) Univariable Cox model results for each quantitative marker (hazard ratio, 95% CI, 2-sided p-value)
2. (Obj. 1,2) Univariable Cox model results for each tertilized marker (hazard ratios, 95% CIs, 2-sided p-values, Generalized Wald p-values)
3. (Obj. 1,2) Univariable Cox model marginalized marker-conditional mean cumulative incidence curves over time through to the last time point t_0, for Low, Medium, High tertile marker subgroups.
4. (Obj. 1,2) Univariable Cox model marginalized marker-conditional mean cumulative incidence curve over time through to the last time point t_0, with marker subgroups defined by the continuous value of the marker.
5. (Obj. 1,2) Univariable nonparametric monotonic-regression model (Kenny PhD dissertation) marginalized marker-conditional mean cumulative incidence curve over time through to the last time point t_0, with marker subgroups defined by the continuous value of the marker.
6. (Obj. 1,2) Multivariable Cox model for the two quantitative markers (anti-Spike IgG to BA.1, nAb ID50 titer to BA.1) (hazard ratios, 95% CIs, 2-sided p-values, generalized Wald test p-value)

7. (Obj. 1,2) Multivariable Cox model for the two tertilized markers (anti-Spike IgG to BA.1, nAb ID50 titer to BA.1) (hazard ratios, 95% CIs, 2-sided p-values, generalized Wald test p-values)

8. (Obj. 3,4) Multivariable Cox model for each of the two quantitative markers including an interaction term for naïve/non-naïve status (Obj. 3, 6) or for the BD1 antibody marker (Obj.4): A Wald p-value for interaction/effect modification is calculated

9. (Obj. 1, 2) Nonparametric threshold TMLE analysis the same as done in Gilbert et al. (2022b) with the method of Van der Laan and Gilbert (2022).

5.3.1 Covariates adjusted for in CoR and CoP analyses

The following covariates are adjusted for in all CoR and CoP analyses: baseline behavioral risk score, heightened at-risk indicator, and indicator of White Non-Hispanic (same three variables as adjusted for in Gilbert et al. (2022b)). Analyses pooling over naive and non-naive include adjustment for naive status. Moreover, for the pooled analysis the multivariable superlearning CoR analyses also adjust for the interaction of heightened at-risk indicator with naive status.

In addition to the baseline covariates $\mathbf{X}$, controlled risk CoP analysis that imagines "intervening" on a post-randomization event like BD29 antibody titer will also adjust for covariates measured after baseline but prior to BD29 and are associated with both the BD29 antibody titer and the endpoint; see Section 5.4 for details. Such covariates will include tertiles of BD1 antibody titer. For the analyses that pool over naïve and non-naïve, the analyses also adjust for naïve/non-naïve status. This is done because naïve/non-naïve status is strongly predictive of COVID-19, and likely will also be quite predictive of the BD29 antibody markers, such that it is likely a confounder of the effects of BD29 antibody markers on COVID-19.

The last time point t_0 for analysis is defined taking into account the smallest of the two latest COVID-19 endpoint failure times for naňe and non-naňe individuals, which is similar to as in Gilbert et al. (2022b) except only naïve individuals were studied previously. For the overall analysis of booster vaccine efficacy against Omicron, t_0 was selected as PENDING/TBD days, as the latest time point with reasonable precision in estimation.

5.3.2 Machine learning analysis to estimate best models for predicting COVID-19

This analysis will only be pursued if the lower-dimensional CoR analyses of Objectives 1–4 generate substantial signal and motivate a machine-learning multivariable CoR analysis. The analysis will be conducted in the same way as done in Benkeser et al. for the multivariable Moderna correlates analysis of two-dose vaccine recipients (Benkeser et al., 2023), except 1) the markers involved and the baseline covariates involved are different and data from all participants are included, 2) to identify interactions between markers, SL.step.interaction will be added to the learner library, These variables are listed below, in the different sets for which a superlearner model is built. Cross-validated area under the ROC curve (CV-AUC) and variable importance analysis will also be conducted in the same way as done in (Benkeser et al., 2023).

1. Baseline demographics (= variables described in Section 5.3.1) and for analyses pooling over

naive and non-naive also include naive status and the interaction of heightened at-risk indicator with naive status.

2. Possible antibody marker variable sets accounting for assay type (bAb, nAb) where bAb refers to anti-Spike (D614, BA.1), anti-RBD (D614 only), and time point (BD1, BD29, BD29-fold-rise, which includes 2FR and 4FR variables – indicators of two-fold and four-fold rise)
    - BD1 bAb all BA.1
    - BD29 bAb all BA.1
    - BD29-fold-rise bAb all BA.1
    - BD1 nAb all BA.1
    - BD29 nAb all BA.1
    - BD29-fold-rise nAb all BA.1
    - BD1 bAb, BD29 bAb all BA.1
    - BD1 bAb, BD29-fold-rise bAb all BA.1
    - BD29 bAb, BD29-fold-rise bAb all BA.1
    - BD1 nAb, BD29 nAb all BA.1
    - BD1 nAb, BD29-fold-rise nAb all BA.1
    - BD29 nAb, BD29-fold-rise nAb all BA.1
    - BD1 (bAb, nAb) all BA.1
    - BD29 (bAb, nAb) all BA.1
    - BD29-fold-rise (bAb, nAb) all BA.1
    - BD1 (bAb, nAb,) BD29 (bAb, nAb) all BA.1
    - BD1 (bAb, nAb), BD29-fold-rise (bAb, nAb) all BA.1
    - BD29 (bAb, nAb), BD29-fold-rise (bAb, nAb) all BA.1
    - Repeat the above 18 sets for all D614 / D614G
    - Repeat the above 18 sets for all BA.1 and D614 / D614G

As for other analyses, the analysis is done separately for naïves, non-naïves, and naïves + non-naïves pooled.

5.4 Assessing Objectives 5 and 6 (≈peak Ab as controlled risk Correlates of Protection)

5.4.1 Primary controlled risk CoP analysis

Each of the BD29 and BD29 fold-rise markers is assessed as a controlled risk CoP as defined in Gilbert et al. (2022a), which is based on boosted participants only without a contrast of controlled risk in not-yet-boosted participants. This is analogous to CoR analysis of the vaccine arm only in the original blinded vaccine vs. placebo stage of the trial. This analysis reports E-values for each marker analyzed in tertiles and reports ignorance intervals and 95% estimated uncertainty intervals around the controlled risk curve estimate as a function of continuous immune marker value via the Cox modeling approach, the same as was done in the first Moderna CoP analysis (Gilbert et al., 2022b). As described in Gilbert et al. (2022a, Section 2.1), the objective of a controlled risk CoP analysis is to estimate the controlled risk parameter that assesses the causal effect of the antibody marker on COVID-19 risk. A controlled risk CoP analysis is different from a controlled vaccine efficacy CoP analysis (see Section 5.5), whose goal is to contrast participants in the vaccination arm and that in the not-yet-boosted arm.

Our primary interest is to assess the BD29 biomarker as a controlled risk CoP for the population who received the 3rd dose of mRNA-1273 vaccine in the COVE cohort. We will pursue this goal in the naïve and non-naïve populations, separately.

To be more specific, we will study the following causal estimand. Let $T(Ab1)$ denote the time to Omicron BA.1 COVID-19 after receiving the booster under assignment of all participants to $BD29 = Ab1$. For a fixed time t_0 after receiving the booster shot, define

$$r_M(Ab1) := \mathbb{E}_{\mathcal{P}_{\mathbf{L}}}[P(T(BD29 = Ab1) \leq t_0)],$$

where $\mathbf{L}$ denotes a vector of pre-treatment covariates ('pre-treatment' with respect to BD29) and $\mathcal{P}_{\mathbf{L}}$ is the distribution of $\mathbf{L}$ in the "per-protocol" naïve or non-naïve populations who received a booster.

Identification of $\mathbb{E}_{\mathcal{P}_{\mathbf{L}}}\{P(T(BD29 = Ab1) \leq t_0)\}$ from observed data depends on the ignorability assumption. One version of the ignorability assumption states that the BD29 titer level is independent of potential outcomes $T(Ab1)$ conditional on baseline covariates $\mathbf{X}$, including the minority indicator, high risk indicator and risk score, and covariates collected at BD1, including the matching biomarker level (or its tertiles) at BD1.

Under this ignorability assumption, the quantity $\mathbb{E}_{\mathcal{P}_{\mathbf{L}}}\{P(T(BD29 = Ab1) \leq t_0)\}$ is identified from observed data via the following g-computation formula (Gilbert et al., 2022a):

$$r_M(Ab1) := \mathbb{E}_{\mathcal{P}_{\mathbf{L}}}\{P(T(BD29 = Ab1) \leq t_0)\} = \mathbb{E}_{\mathcal{P}_{\mathbf{L}}}\{P(T \leq t_0 \mid BD29 = Ab1, \mathbf{L})\}, \qquad (1)$$

where $\mathbf{L}$ is specified above. To faciliate interpretation, for a fixed BD1 Ab tertile, the controlled risk curve $r_M(Ab1)$ will be plotted against $Ab1$ and used to assess the BD29 biomarker of interest as a controlled risk CoP.

Analyses outlined above will be done with BD29 titer replaced by fold-increase from BD1 to BD29 (Objective 6).

5.4.2 Exploratory controlled CoP analysis

We may pursue the following exploratory analyses. First, in addition to assessing the controlled risk CoP in the boosted population, we could also assess BD29 as a controlled risk CoP in the COVE trial population. Let $\mathcal{P}_{\mathbf{X}}$ denote the distribution of baseline covariates $\mathbf{X}$ in COVE. The parameter of interest would be $\mathbb{E}_{\mathcal{P}_{\mathbf{X}}}\{P(T(BD29 = Ab1) \leq t_0)\}$. Identification of $\mathbb{E}_{\mathcal{P}_{\mathbf{X}}}[P(T(BD29 = Ab1) \leq t_0 \mid \mathbf{X})]$ from observed data depends on the sequential ignorability assumption; see, e.g., Joffe and Greene (2009, Section 2.3) and Gilbert et al. (2022a, Supplementary Material B). One version of the sequential ignorability assumption states that the BD29 titer level is independent of potential outcomes $T(Ab1)$ conditional on baseline covariates $\mathbf{X}$, tertiles of BD1 marker level, and a person's naïve/non-naïve status as discussed in the primary controlled risk CoP analysis.

Under this version of sequential ignorability assumption, the quantity $\mathbb{E}_{\mathcal{P}_{\mathbf{X}}}\{P(T(BD29 = Ab1) \leq t_0 \mid \mathbf{X})\}$ is identified as follows (Gilbert et al., 2022a, Supplementary Material B):

$$\begin{aligned} &P(T(BD29 = Ab1) \leq t_0 \mid \mathbf{X}) \\ =& \sum_{a \in l,m,h; b \in 0,1} P(T \leq t_0 \mid BD29 = Ab1, BD1 = a, \text{Naïve} = b, \mathbf{X}) \times P(BD1 = a, \text{Naïve} = b \mid \mathbf{X}). \end{aligned} \tag{2}$$

where $a \in \{l, m, h\}$ denotes the the low, medium and high tertiles of the matching BD1 biomarker. In practice, the conditional probability $P(BD1 = a, \text{Naïve} = b \mid \mathbf{X})$ can be estimated via a multinomial regression. Finally, we standardize $P(T(BD29 = Ab1) \leq t_0 \mid \mathbf{X})$ to the COVE trial $\mathcal{P}_{\mathbf{X}}$ and obtain a controlled risk curve.

As a second exploratory analysis, we will study the controlled risk CoP in each randomization arm. Let $A = 1 =$ Vaccine if a participant was assigned to the vaccine arm and $A = 0 =$ Crossover if assigned to the placebo arm (and later crossed over to the vaccine arm) in the original COVE study; see Figure 1 for an illustration. Let $T(a, Ab1)$ denote the time to Omicron BA.1 COVID-19 after receiving the booster under assignment of all participants to $A = a$ and $BD29 = Ab1$. For a fixed time t_0 after receiving the booster shot, let $r_M(a, Ab1) := \mathbb{E}_{\mathcal{P}_{\mathbf{X}}}[P(T(A = a, BD29 = Ab1) \leq t_0 \mid \mathbf{X})]$, where a = Vaccine or Crossover, $\mathbf{X}$ denotes a vector of baseline covariates, and $\mathcal{P}_{\mathbf{X}}$ is the distribution of $\mathbf{X}$ in the COVE trial population.

Identification of $\mathbb{E}_{\mathcal{P}_{\mathbf{X}}}[P(T(A = a, BD29 = Ab1) \leq t_0)]$ from observed data depends is analogous to the two-stage g-computation discussed previously. Separate estimates of the curves $r_M(a, Ab1)$ in $Ab1$ for each $a = 0, 1$ will be produced. The contrast $r_M(\text{Vaccine}, Ab1)/r_M(\text{Crossover}, Ab1')$ will also be reported. This contrast characterizes the "joint effect" of being assigned to the vaccine versus crossover (which had an implication for the interval time and could potentially have an effect on the clinical outcome via a causal pathway not mediated by the BD29 antibody titer) and different levels of BD29 antibody titer.

In a third exploratory analysis, the potential outcome of interest is:

$$T(\Delta, Ab1) := T(\text{receiving booster } \Delta \text{ days after the 2nd vaccine}, BD29 = Ab1).$$

The identification of the controlled risk based on the potential outcome $T(\Delta, Ab1)$ will be based on a two-stage generalization g-computation discussed previously if the target population is the entire

COVE population and a single-stage g-computation if the population of interest is the boosted population. For selected values of Δ, a controlled risk curve could be plotted as a function of BD29 titer level. In addition, controlled vaccine efficacy can be estimated and plotted for two distinct values of Δ, e.g., the 10th and 90th percentiles.

Analyses outlined above will be done with BD29 titer replaced by fold-increase from BD1 to BD29 (Objective 6).

5.5 Controlled VE CoP analysis of Objectives 5 and 6 based on boosted vs. not-yet boosted

In addition to the controlled risk CoP analysis, for assessing the BD29 antibody marker as a CoP against Omicron COVID-19, another approach measures the booster VE, defined as the hazard rate of COVID-19 for boosted vs. not-yet boosted individuals, or alternatively by the cumulative probability of COVID-19 by a given fixed time point for boosted vs. not-yet boosted individuals. We will study how the booster VE varies as a function of BD29 antibody level Ab1, through the stepped-wedge methodology designed by Fintzi and Follmann (2021).

To be more specific, at any time t, the risk set would consist of not-infected-by-Omicron participants who are at least 7 days post BD29 and not-yet boosted participants. Each boosted participant is associated with a BD29 antibody level and the hazard rate conditional on the BD29 Ab level, $\lambda_{\text{boost}}(t, Ab1)$, will be estimated. On the other hand, the hazard among the not-yet boosted participants, $\lambda_{\text{not-yet-boost}}(t)$, will also be estimated. The contrast $1 - \lambda_{\text{boost}}(t, Ab1)/\lambda_{\text{not-yet-boost}}(t)$ or boost efficacy by Ab1, will be reported and plotted as a function of of the BD29 antibody marker Ab1.

An estimate of the overall booster VE against Omicron COVID-19 provides a way to scale the controlled risk curve (marginalized Omicron COVID-19 risk vs. BD29 antibody level Ab1) to be a booster-controlled VE curve; see Gilbert et al. (2022d, Section 2.1) for the distinction between a controlled risk CoP analysis discussed in Section 5.3 and the controlled VE CoP analysis outlined in this section.

The cohort for the CoP analysis will be comprised of everyone who is unboosted as of 1 December 2021, plus the stratified case-control cohort (SCCC). Separate datasets and analyses will be constructed for the non-naive and naive cohorts. An illustrative version of the hazard function for peak antibody CoP analysis is given by

$$h(t) = h_0(t)\exp\{Z_i(t)[\beta_0 + \beta_1 Ab1] + X_i\theta\}w_i(t)I(t \in R_i)$$

where R_i is defined to remove person i from the risk set after event, censoring, or the 34 days postboost interval, t is days since 1 December 2021, $Z_i(t)$ is 0 before boost and 1 after boost and X_i is a vector of covariates. The weight $w_i(t)$ is a little complicated. There are three categories.

1. For those in the SCCC BD cohorts for period 1-3, $w_i(t)$ is the IPS weight.
2. For those in the SCCC BD period 4 cohort (boosted 1 December 2021 to 31 December 2021), $w_i(t) = 1$ prior to boosting and is the IPS weight after boosting.

3. For those not in the SCCC and unboosted/prior to boosting on 1 December 2021, $w_i(t)$ is 1 prior to boosting/COVID-19 event and 0 after boosting or COVID-19 event.

As an alternative, we will avoid weighting by imputing Ab1 in all vaccinees by empirical sampling with replacement from the distribution of BD29 antibody or either cases or controls, as appropriate. This should result in a much reliable estimate of β_0.

The same analysis will be conducted with the BD29 titer replaced by fold-increase from BD1 to BD29 (Objective 6).

Repeating correlates analyses in the early period of follow-up in acknowledgment of waning vaccine efficacy

Several studies have shown that mRNA booster vaccines have waning protection over time, including analysis of the COVE trial itself. Correlates of protection may be strongest and most interpretable during periods of substantial vaccine protection. Therefore, the ≈peak time point CoR and CoP analyses may be repeated using as the final time point $t_0 = 91$ days post Day 1 visit. This cut-point of 91 days is chosen in part to harmonize with the COVAIL immune correlates study that also assesses immune correlates restricting to COVID-19 endpoints occurring 91 days post booster. In addition, the immune correlates analyses may also be repeated restricting to COVID-19 endpoints occurring starting 92 days post Day 1 visit through to the final time point t_0 that was selected for the main correlates analyses.

5.6 Assessing Objectives 7-9 (Exposure-Proximal Correlates of Risk and Correlates of Protection)

For assessing antibody as an exposure-proximal CoR (Objective 7), we use the below Cox model

$$h(t) = h_0(t)\exp\{Z_i(t)[\beta_0 + \beta_1 Ab_i(t-b)] + X_i\theta\}w_i(t)I(t \in R_i),$$

where t is days since 1 December 2021 and $Ab_i(t-b)$ is the predicted antibody level for person i at time $(t-b)$ post boost with other terms as defined in section 5.4. A CoR analysis will draw estimated curves with confidence bands of $exp\{\beta_1 Ab\}$ as a function of Ab ranging over the middle 95% of the distribution of predicted Ab. Assessing the Objective 8 will include a term for BD1 dichtomized at the median of the BD1 distribution and an interaction of dichtomized BD1 with Ab_i. Objective 9 will use the same model and provide CoP curves analogous to the CoR curves using $1 - exp(\beta_0 + \beta_1 Ab)$. Below we describe how we will impute $Ab(t)$.

In select cases the BD29 and DD1 antibody readouts will be used to calculate individual slopes using the form (BD29-DD1)/d where BD29 and DD1 are the antibody readouts and d the difference in days between BD29 and DD1. Denote the median slope as $\hat{\theta}$ which will be used to calculate individual antibody decay curves for *all* cases and non-cases using the formula

$$\text{Ab}(d) = \text{BD29} + \hat{\theta} \times d,$$

where d is the number of day post BD29. This imputation will be performed for all individuals in the risk set at all event times. This approach has been applied to the Stage 1 blinded-phase COVE

data, though the slope of decay there was estimated using data from Doria-Rose et al. (2021). Note that even though we have DD1 antibody value for the cases we don't use it in this approach in order to treat cases and controls the same way. It's bad if a covariate is measured one way for cases and another way for controls and the above symmetric imputation avoids this problem. Another reason not to impute DD1 is that the interval between BD29 and DD1 is random, which makes imputation problematic.

If the total variance is large relative to the within person variance, the above regression calibration approach may result in bias and to reduce such bias, an expected partial likelihood estimator may be considered.

The above analyses will be run separately for the naive and non-naive cohorts.

5.7 Addressing Objective 10 on mediation of the effect of dose 2 to 3 interval on COVID-19 mediated through BD29 antibody

This question will be analyzed by a new method described in a manuscript under preparation (Hejazi et al., 2023). The exposure variable of interest A must be dichotomous, so it will be defined as above vs. below the median number of days between dose 2 and dose 3. The putative mediator to study is BD29 $\log_{10}$ PsV nAb ID50 titer against BA.1, and the outcome is COVID-19, both variables defined the same as for the other ≈peak antibody correlates objectives. Covariates to adjust for W will also be the same as used for the other ≈peak antibody correlates analyses. The analysis will be done with and without V defined as the BD1 $\log_{10}$ PsV nAb ID50 titer against BA.1. The data set up fits the method of Hejazi et al. (2023) where V is a likely confounder of the exposure-mediator relationship given that V predicts both A and the putative mediator.

The data analysis will be repeated for $\log_{10}$ PsV nAb ID50 titer against D614G as well as for each of the other markers $\log_{10}$ anti-Spike BA.1 IgG, $\log_{10}$ anti-Spike D614, IgG $\log_{10}$, and $\log_{10}$ anti-RBD D614 IgG.

This data analysis is based on a novel statistical method that is still being developed, which will be submitted as part of a statistical methods manuscript. Consequently, the results of this method will likely come later than results from the other analyses, and hence will likely be included in sequel manuscripts rather than in the first correlates manuscript resulting from this SAP.

6 Specifications for general issues faced for most analyses

6.1 Computation of inverse probability of sampling weights

Define six demographic categories, which were used in the stratified sampling design: Age ≥ 65 minority; Age 18-64 'at risk' minority; Age 18-64 'not at risk' minority; Age ≥ 65 non-minority; Age 18-64 'at risk' non-minority; Age 18-64 'not at risk' non-minority, i.e., to enrich/over-sample those Age ≥ 65]. For each sampled participant, the inverse probability sampling weight is computed as numerator / denominator, where the numerator is the total number of per-protocol participants in the participant's cell (among the 32 of Table 1) that also have membership in the participant's demographic category (among the 6 listed above). The denominator is the total number of participants included in the numerator that were sampled for stage 2 correlates.

6.2 Imputation of demographics variables for stratification and merging of sparse strata for weights computation

Wstratum depends upon the demo variables (age, at risk, minority), CalendarBD1Interval, naive, and trt. Controls with missing Wstratum won't be sampled, hence not part of ph1. On the other hand, cases with missing Wstratum are part of ph1 because they may be sampled. If cases have missing demo variables, we want to impute them so that we can assign weights, otherwise it gets too complicated to assign weights to cases. Imputation is performed over all cases and controls without missing demo variables. The latter are included to improve imputation performance. Imputation is performed for demo variables only, but can be enlarged if there are additional variables that provide info on the three demo variables. Due to the limited missingness, a single hard imputation is performed.

When there are strata with empty ph2 sample set, collapsing strata is performed in three steps. First, do it across demo strata within each of 32 sampling buckets. Second, if there are still empty strata, do it across the 4 calendar periods. Specifically, merge a period with the next period if not the last, and merge with the last period with the previous if needed. Third, do it across demo strata within each of 32 sampling buckets one more time because it is possible that collapsing across time periods in step 2 introduced empty demo strata. Assuming that DD1 may not be available for all cases with BD1 and BD29 markers, we will compute a different set of weights for DD1, which may be used for, e.g. computing positive response rates at DD1. We will first attempt to compute weights for DD1 using the Wstratum derived for computing BD29 weights. If it turns out that there are empty cells, we will re-collapse sampling strata to compute weights for DD1.

7 Additional data analysis issues

7.1 Exclude participants reporting being HIV positive from the correlates analysis

Because the lentivirus-based pseudovirus neutralization assay uses an HIV backbone, the presence of anti-retroviral drugs in serum can give a false positive neutralization signal. For this reason, the original immune correlates analysis Gilbert et al. (2022b) excluded participants who self-reported being HIV positive, because they would likely be taking anti-retroviral drugs. Consistent with the previous correlates analysis, this SAP also excludes participants who self-reported being HIV positive.

7.2 Missing lineages

Some endpoint cases will likely have missing lineage/spike sequence. If the COVID-19 endpoint diagnosis date is $\geq$ January 15, 2022, then the lineage will be hard-imputed to be Omicron BA.1. If the COVID-19 diagnosis date is less than January 15, 2022, the lineage will be recorded as NA. Note that attempts were made to measure the lineage/sequence for 100% of selected cases, enabling addressing this issue in the data analysis. Data analyses will restrict to Omicron BA.1 cases, although if the number of non-naïve cases has more than 10% of missing lineages, then missing data methods may be used that account for missing lineage. A separate SAP describes an

approach to doing this using hotdeck multiple imputation, similar to as in Sun et al. (2020), which may be added to this SAP if needed.

References

Atmar, R.L., Lyke, K.E., Deming, M.E., Jackson, L.A., Branche, A.R., El Sahly, H.M. et al (2022), "Homologous and heterologous Covid-19 booster vaccinations," *New England Journal of Medicine*, 386, 1046–1057.

BARDA (2021), "04Nov2021_VSDVAC_62_V300_AES_VAC_62toHistorical_Assays," .

Benkeser, D., Montefiori, D., McDermott, A., Fong, Y., Janes, H., Deng, W. et al (2023), "Comparing antibody assays as correlates of protection against COVID-19 in the COVE mRNA-1273 vaccine efficacy trial," *Science Translational Medicine*.

Bergwerk, M., Gonen, T., Lustig, Y., Amit, S., Lipsitch, M., Cohen, C. et al (2021), "Covid-19 breakthrough infections in vaccinated health care workers," *New England Journal of Medicine*, 385, 1474–1484.

Doria-Rose, N., Suthar, M.S., Makowski, M., Oâ€™Connell, S., McDermott, A.B., Flach, B. et al (2021), "Antibody persistence through 6 months after the second dose of mRNA-1273 vaccine for Covid-19," *New England Journal of Medicine*, 384, 2259–2261.

Fintzi, J. and Follmann, D. (2021), "Assessing vaccine durability in randomized trials following placebo crossover," *Statistics in Medicine*, 40, 5983–6007.

Gilbert, P., Fong, Y., Kenny, A. and Carone, M. (2022a), "A Controlled Effects Approach to Assessing Immune Correlates of Protection," *Biostatistics*.

Gilbert, P.B., Montefiori, D.C., McDermott, A.B., Fong, Y., Benkeser, D., Deng, W. et al (2022b), "Immune correlates analysis of the mRNA-1273 COVID-19 vaccine efficacy clinical trial," *Science*, 375, 43–50.

Gilbert, P.B., Montefiori, D.C., McDermott, A.B., Fong, Y., Benkeser, D., Deng, W. et al (2022c), "Immune correlates analysis of the mRNA-1273 COVID-19 vaccine efficacy clinical trial," *Science*, 375, 43–50.

Gilbert, P.B., Isbrucker, R., Andrews, N., Goldblatt, D., Heath, P.T., Izu, A. et al (2022d), "Methodology for a correlate of protection for group B Streptococcus: Report from the Bill & Melinda Gates Foundation workshop held on 10 and 11 February 2021," *Vaccine*, 40, 4283–4291.

Huang, Y., Borisov, O., Kee, J.J., Carpp, L.N., Wrin, T., Cai, S. et al (2021), "Calibration of two validated SARS-CoV-2 pseudovirus neutralization assays for COVID-19 vaccine evaluation," *Scientific reports*, 11, 23921.

Joffe, M. and Greene, T. (2009), "Related causal frameworks for surrogate outcomes." *Biometrics*, 65, 530–538.

Lyke, K.E., Atmar, R.L., Islas, C.D., Posavad, C.M., Szydlo, D., Chourdhury, R.P. et al (2022), "Rapid decline in vaccine-boosted neutralizing antibodies against SARS-CoV-2 Omicron variant," *Cell Reports Medicine*, 3.

Sun, Y., Qi, L., Heng, F. and Gilbert, P.B. (2020), "A hybrid approach for the stratified mark-specific proportional hazards model with missing covariates and missing marks, with application to vaccine efficacy trials," *Journal of the Royal Statistical Society: Series C (Applied Statistics)*, 69, 791–814.

Van der Laan, L. and Gilbert, P. (2022), "Targeted semiparametric estimation of the relative strain-specific conditional vaccine-efficacy in observational studies with cases-only data," .

Appendix A: Stage 2 Sampling: Stratified Case-Control Samples in 3-dose vaccine recipients

Appendix A.1 Stratified case-control sampling

Participants in P301 started receiving booster dose in Sep-2021 (first subject first booster dose 23-Sep-2021 in P301 Part C), and a total of 19,609 participates received a booster dose in Part C (Part C Safety Set, data cutoff date: 05-Apr-2022). Part C Safety Set will be used as the source dataset to sample for the stage 2 sampling. In this sampling plan, Omicron case, is approximated by adjudicated COVID-19 case (positive RT-PCR for SARS-CoV-2 with eligible symptoms) $\geq$ 7 days post BD29 AND $\geq$ 01-Dec-2021 given the emergence of Omicron (BA.1) wave. Primary endpoint COVID-19 cases with known Omicron BA.1 lineage are prioritized for sampling. The sampling of cases and controls are further stratified by the following:

1. Originally randomized to mRNA-1273 (mRNA-1273, original vaccine arm) vs. placebo recipients in the blinded phase (Part A) who received mRNA-1273 primary series in Part B (Placebo-mRNA-1273, cross-over vaccine arm), these two groups/arms create useful variability in the time between the two-dose vaccination series and the booster dose.

2. Calendar period a participant received a booster (23-Sep to 15-Oct-2021, 16-Oct to 31-Oct-2021, Nov-2021, Dec-2021).

3. naïve vs. non-naïve cohorts, where naïve participants are those with no evidence of SARS-CoV-2 infection through the day of receiving booster (BD-Day 1, or pre-booster, or BD1); and non-naïve participants are those with evidence of infection in [date of 2nd dose of the primary series + 14 days, BD1]. Infection is defined by either a positive RT-PCR for SARS-CoV-2, or conversion from non-positive to positive by Roche Elecsys assay (NP).

An equal number of cases vs. non-cases, 8 within each of the stratum defined by the above cross-classification will be sampled, as presented in Table 1 below. Primary endpoint COVID-19 cases with known Omicron BA.1 lineage are prioritized for sampling If fewer than 8 such eligible cases are available for sampling, then the remainder of the cell is filled with eligible cases with unknown lineage. For cases, antibodies at 3 timepoints: pre-booster Day 1 (BD1), 1 month/28 days after booster (BD29) and illness Day 1 (DD1) will be measured; for non-cases, antibodies at 2 timepoints: BD 1 and BD29 will be measured.

Appendix A.2 Specifications for Sampling

Participants who are in Per-protocol Primary Series analysis set and received booster dose (ADSL.PPPSFL = ‘Y’ AND ADSL.TR03SDT > .) are used for sampling. Eligible participants to be sampled also requires Case/Non-case, naïve/non-naïve, received booster during [23-Sep-2021, 31-Dec-2021] as defined in section 2.1.

For cases, severe Omicron COVID-19 cases (onset $\geq$ 01-Dec-2021) will be sampled first. Within each stratum, a random number generator function with seed of 1273 is used. Sampled participants who did not have sufficient serum samples available at the planned timepoints (BD1 and BD29 for non-cases, BD1, BD29, and DD1 for cases) will be replaced. Based on preliminary review of data, the number of non-naïve Omicron COVID-19 cases is very limited. Thus, for non-naïve Omicron cases,

eligible primary endpoint COVID-19 cases will be sampled first. The remaining non-naïve cases will be sampled from infection cases (positive RT-PCR not necessarily with eligible symptom(s)), first from those with known Omicron BA.1 lineage, then from those likely to be Omicron BA.1. For these non-naïve Omicron COVID-19 cases and infections, every effort will be made to sample up to 8 participants, even if serum samples are not available at all 3 preferred timepoints (BD1, BD29, and DD1). If there are still not sufficient non-naïve cases, to fill out the 8 cases additional adjudicated COVID-19 cases 7 days post BD29 with onset $\geq$ 01-Dec-2021. In summary, in the situation available non-naïve cases are <8 in a cell, effort will be made: to sample a total of 16 cases for each arm per boosting calendar period as described above. Effort will be made to maintain 1:1 ratio between case: non-case for naïve and non-naïve cohort. In the situation when there are only x (<8) non-naïve cases to be sampled, 16-x naïve cases will be sampled to reach a total of 16 cases. Correspondingly, 16-x naïve non-cases and x non-naïve non-cases will be sampled be maintain 1:1 between cases: non-cases, as illustrated as an example in the table below:

Omicron case	16-x	x
	N	NN
non-case	16-x	x
	N	NN

When feasible, for each set of 8N (naïve) or 8NN (non-naïve) in a cell, sample 2:1:1:2:1:1 from baseline demographic strata: Age $\geq$ 65 minority; Age 18-64 'at risk' minority; Age 18-64 'not at risk' minority; Age $\geq$ 65 non-minority; Age 18-64 'at risk' non-minority; Age 18-64 'not at risk' non-minority, i.e. to enrich/over-sample those Age $\geq$ 65].

Appendix B: Notes for construction of a mock data set

1. Need the mock data set to include all of the variables used for sampling as described in Table 1, which means adding a variable coding the four calendar boosting intervals, and adding a new variable to indicate naïve vs. Non-naïve. This is needed for computing sampling weights as well as for other purposes such as covariate adjustment.

2. The markers that need to be simulated are BD1, BD29 values (and DD1 values for cases) of:

    - $\log_{10}$ nAb titer to D614G
    - $\log_{10}$ nAb titer to BA.1
    - $\log_{10}$ anti-Spike IgG to D614
    - $\log_{10}$ anti-Spike IgG to Gamma
    - $\log_{10}$ anti-Spike to Alpha
    - $\log_{10}$ anti-Spike to Beta
    - $\log_{10}$ anti-Spike to Delta AY4

- $\log_{10}$ anti-Spike to BA.1
- $\log_{10}$ anti-RBD IgG to D614

For the naïve cohort, the BD1, BD29, and DD1 values could all be taken to be like D29, D57, and D29 values against D614/D614G of baseline negatives from the original COVE study, respectively, by assay type MSD/binding and pseudovirus neutralization, using BAU/ml and IU50/ml. For readouts to strains other than D614/D614G, will re-sample from the D614/D614G strain data. For the Non-naïve cohort, the BD1 and BD29 values could be taken from the D29 and D57 values against D614/D614G of baseline positives from the original COVE study, respectively, by assay type MSD/binding and pseudovirus neutralization. For DD1 values, could be taken from the D29 values against D614/D614G of baseline positives from the original COVE study. After readouts are calculated, for the MSD binding antibody data, values below the constant/non-strain-specific LLOQ (on the BAU/ml scale) are set to LLOQ/2, and for the nAb ID50 titer data, values below the constant/non-strain-specific LOD (on the IU50/ml scale) are set to LOD/2.

Appendix C: Miscellaneous

tfinal.tpeak is the minimum of tfinal.tpeak for each of four quadrants (2 trt * 2 naive status) and no larger than 105 (Dean et al's analyses). Within each qudrant, it is defined as smaller of the two: 1) time of the last case, 2) last time to have 15 ph2 samples at risk.

Appendix D: Definition of Stage-2 Per-Protocol Population

Booster dose correlates studies will be restricted to the "stage-2 per-protocol" (BDPerprotocol) population where the flag BDPerprotocol == TRUE if the participant satisfies the following two criteria:

1. The participant was in the original blinded-phase per-protocol cohort as in Gilbert et al. (2022b);
2. The participant received the booster dose (third mRNA-1273 dose) before and including December 31, 2021;

and belongs to one of the following 4 sampling strata:

1. "Case/Naïve"
2. "Case/Non-Naïve"
3. "Non-Case/Naïve"
4. "Non-Case/Non-Naïve"

where "naïve," "non-navë," "case," and "non-case" are defined as follows:

Naïve No evidence of SARS-CoV-2 infection detected by elecsys or RT-PCR from enrollment through BD1;

Non-Naïve Any evidence of SARS-CoV-2 infection in the interval [14 days after the second dose of mRNA-1273 vaccine, BD1];

Case 1) If a participant is naïve, then case is Omicron COVID-19 event in the interval [max(7 days post BD29, 01 Dec2021), 16 May 2022 database lock date]; 2) If a participant is non-naïve, then case is SARS-CoV-2 infection detected by Elecsys or RT-PCR in the interval [max(7 days post BD29, 01 Dec2021), 16 May 2022] and the Elecsys test was not positive at BD-D1 pre-booster;

Non-Case No evidence of SARS-CoV-2 infection detected by Elecsys or RT-PCR in the interval (BD1, 16 May 2022 database lock date].

For all analyses of the peak immune correlates (i.e., BD29 response), the "per-protocol" status further requires that

1. The participant did not miss a BD29 visit;
2. The participant had a BD29 measurement (approximate peak measurement) that was between 19 and 45 days, both inclusive, of the BD1 visit;
3. The participant was not censored or had any evidence of infection before 7 days post BD29 visit.
4. The participant did not live with HIV.
5. The participant did not acquire a non-adjudicated Omicron endpoint.
6. The participant did not test SARS-CoV-2 RT-PCR positive at the BD1 visit.

In the correlates studies of the "per-protocol" population, a study participant would be considered "naïve" by BD1 if there was no evidence of SARS-CoV-2 infection (RT-PCR+, Roche Elecsys seropositive, or a symptomatic COVID-19 endpoint followed by positive confirmatory testing) from enrollment to BD1 (including the BD1 visit). A study participant is considered "non-naïve" if the participant showed any evidence of COVID-19 starting 14 days post the second immunization in the primary series and through the BD1 visit.

Because a participant who tested PCR+ at BD1 was excluded from the per-protocol cohort, the person would not be associated with a naive/non-naive status.
