# Supplementary material for: Omicron COVID-19 immune correlates analysis of a third dose of mRNA-1273 in the COVE trial
Source: Nat Commun. 2024 Sep 11;15:7954. doi: 10.1038/s41467-024-52348-9 (PMC11390939; doi:10.1038/s41467-024-52348-9)
Supplement: Supplementary file 3 — Description of Additional Supplementary Files [file 41467_2024_52348_MOESM3_ESM.pdf]

Description of Additional Supplementary Files

File Name: Supplementary Software 1
Description: The zip file includes an RMD file, “exposure_proximal_code”, which helps walk through all code and produce all results/figures; a mock dataset, “dat_full_mock.csv”, a README txt file, and a data dictionary. The running time is < 2 mins.
