# Supplementary material for: Omicron COVID-19 immune correlates analysis of a third dose of mRNA-1273 in the COVE trial
Source: Nat Commun. 2024 Sep 11;15:7954. doi: 10.1038/s41467-024-52348-9 (PMC11390939; doi:10.1038/s41467-024-52348-9)
Supplement: Supplementary file 4 — Supplementary Software 1 [file 41467_2024_52348_MOESM4_ESM.zip › DataDictionaryModernaCOVEBoost.docx]

| Variable Name | Description | Possible Values | Notes |
| --- | --- | --- | --- |
| Ptid | Participant identifier | US3xxx no NAs allowed |  |
| Trt | Booster assignment | 1=Booster  0=Not receiving booster | NA if a participant is not included in the Stage 2 analysis |
| **Ethnicity Information** |  |  |  |
| EthnicityHispanic | Indicator ethnicity = Hispanic | 1=Hispanic 0=Else no NAs allowed | Participants with NA might receive the booster dose as the stage 2 analysis data didn’t include all booster recipients |
| EthnicityNotreported | Indicator ethnicity = Not reported | 1=Ethnicity not reported 0=Else no NAs allowed) | Definition of BD1 naïve/non-naïve can be found in the manuscript |
| EthnicityUnknown | Indicator ethnicity = Unknown | 1=Ethnicity unknown 0=Else no NAs allowed | Built by machine learning |
| **Race Information** |  |  | Naïve = 1 - nnaive |
| Black | Indicator race = Black | 1=Black 0=Else no NAs allowed |  |
| Asian | Indicator race = Asian | 1=Asian 0=Else no NAs allowed |  |
| NatAmer | Indicator race = American Indian or Alaska Native | 1=American Indian or Alaska Native 0=Else no NAs allowed | All in 2021 |
| PacIsl | Indicator race = Native Hawaiian or Other Pacific Islander | 1=Native Hawaiian or Other Pacific Islander 0=Else no NAs allowed |  |
| Other | Indicator race = Other | 1=Other 0=Else no NAs allowed |  |
| Multiracial | Indicator race = Multiracial | 1=Multiracial 0=Else no NAs allowed |  |
| Notreported | Indicator race = Not reported | 1=Not reported 0=Else, no NAs allowed |  |
| Unknown | Indicator race = Unknown | 1=Unknown 0=Else, no NAs allowed |  |
| **Minority status** |  |  |  |
| MinorityInd | Indicator of under-represented minority | 1=Minority 0=Else no NAs allowed | This variable is the same as URMforsubcohortsampling except missing minority status is assigned value 0 (i.e., missing defaults to non-Minority) |
| URMforsubcohortsampling | Indicator of under-represented minority | 1=Minority 0=Non-minority NA | This variable matches the immunogenicity subcohort sampling design that was implemented. Minority includes Blacks or African Americans, Hispanics or Latinos, American Indians or Alaska Natives, Native Hawaiians, and other Pacific Islanders. Non-Minority includes all other races with observed race (Asian, Multiracial, White, Other) and observed ethnicity Not Hispanic or Latino. Participants not classifiable as Minority or Non-Minority because of unknown, unreported or missing were not included and have value NA. Data analysis below describe how this variable is calculated. |
| **Baseline demographics** |  |  |  |
| HighRiskInd | Indicator of At-risk for COVID-19 condition, a randomization factor | 1=At-risk 0=Not At-Risk NA | At-risk status was a factor in the stratified randomization* |
| Age | Age at enrollment in years | 18 and above NA | Age 18-64, Age >= 65 was a factor in the stratified randomization* |
| Sex | Sex assigned at birth | 1=Female 0=Male NA |  |
| BMI | BMI at enrollment (kg/m^2) | Positive numeric value NA |  |
| HIVinfection | HIV status at enrollment | 1 = Live with HIV  0 = Not live with HIV  NA if not in the stage 2 cohort | Participants with NA might receive the booster dose as the stage 2 analysis data didn’t include all booster recipients |
| nnaive | BD1 naïve status of the participant | 1 = BD1 non-naïve  0 = BD1 naive | Definition of BD1 naïve/non-naïve can be found in the manuscript |
| **Visit interval Information** |  |  |  |
| CaldendarBD1Date | Calendar date of booster dose visit (BD1) | Calendar date of the form yyyy-mm-dd;  NA if not in the stage 2 cohort |  |
| CalendarBD1Interval | Time period of the BD1 visit | 1 = 9/23– 10/15  2= 10/16-10/31  3= 11/1-11/30  4= 12/1-12/31 | All in 2021 |
| NumberdaysBD1toBD29 | Number of days between BD1 visit and BD29 visit | Numeric count NA if missing BD29 visit or not in the stage 2 cohort |  |
| NumberdaysBD1toDD1 | Number of days between BD1 visit and DD1 visit | Numeric count NA if not primary endpoint case or not in the stage 2 cohort |  |
| NumberdaysBD1toBD181 | Number of days between BD1 visit and BD181 visit | Numeric count NA if missing BD181 visit or not in stage 2 cohort |  |
| BD29window | Indicator of if the BD29 visit is within a valid window | 1= the number of days between BD1 and BD29 is within [19, 45];  0= the number of days between BD1 and BD29 < 19 or > 45;  NA if missing BD29 visit or not in the stage 2 cohort |  |
| NumberdaysDS2toDS3 | Number of days between the 2^nd^ dose and the 3^rd^ dose | Numeric count NA if not in the stage 2 cohort | Used for deriving risk_score |
| BeforeMedianUnblind | Indicator of if the unblinding date was prior to the median unblinding date | 1 = unblinding date <= median(unbliding date of Stage 1 Baseline Negative PP participants from the Stage 1 processed data);  0 = otherwise;  NA if not in the stage 2 cohort | Used for deriving risk_score |
| **Per-protocol status** |  |  |  |
| Perprotocol | Indicator of qualifying per-protocol | 1=per-protocol 0=not per-protocol no  NA if not in the stage 2 cohort | Perprotocol = 1 means all following criteria was met during the primary series:  1. participant is in the mITT cohort,  2. The number of days between dose 1 and dose 2 >= 21 and <= 42,  3. There is no severe protocol violation  4. Received treatment as planned.  0 = any of the criteria was not met; |
| BDPerprotocol | Indicator of qualifying BD per-protocol | 1=BD per-protocol 0=not BD per-protocol  NA if not in the stage 2 cohort | BDPerprotocolIncludePos = 1 means all following criteria was met:  1. PPROTFL='Y';  2. in the BD safety population.  3. BD Baseline SARS-CoV-2 Negative  4. Vaccine recipients in the primary series did not received treatments in the OL phase; or Placebo recipients in the primary series were not OL Baseline SARS-CoV-2 positive and the 2 doses in OL were within [21, 42] days  5. no dosing error in BD phase,  6. There is no severe protocol violation during BD phase |
| BDPerprotocolIncludeSeroPos | Indicator of qualifying BD per-protocol (including sero positive) | 1=BD per-protocol and  0=not per-protocol no  NA if not in the stage 2 cohort | BDPerprotocolIncludePos = 1 means the participant met all the BDPerprotocol criteria except being seropositive at BD baseline |
| Stage2SamplingInd | Indicator of being sampled in the case-control cohort | 1 = sampled in the case-control cohort  0 = not sampled in the case-control cohort  NA if not in the stage 2 cohort |  |
| **Early SARS-CoV-2 infection information** |  |  |  |
| EarlyOmicronBD29 | Indicator a participant has Omicron infection < 7 days post BD29 visit | 1=Early infection 0=Else  NA if missing BD29 visit or not in the stage 2 cohort | Individuals with EarlyOmicronBD29==1 are excluded from correlates analyses of Day 29 markers. |
| EarlyPrimaryOmicronBD29 | Indicator a participant has an adjudicated Omicron infection < 7 days post BD29 visit | 1=Early infection 0=Else  NA if missing BD29 visit or not in the stage 2 cohort | Individuals with EarlyPrimaryOmicronBD29==1 are excluded from correlates analyses of Day 29 markers. |
| AnyInfectionBD1 | Indicator a participant has any evidence of previous SARS-CoV-2 infection since BD1 | 1 = any evidence of infection since BD1  0 = no evidence of infection since BD1 |  |
| **Failure time information** |  |  |  |
| EventIndOmicronBD1 | Indicator that the EventTimeOmicronBD1 failure time is <= the right-censoring time | 1=Omicron endpoint 0=right-censored  NA if not in the stage 2 cohort | Includes both adjudicated and non-adjudicated Omicron |
| EventTimeOmicronBD1 | Minimum of the time from BD1 until the Omicron endpoint or right-censoring | Integer in days  NA if not in the stage 2 cohort |  |
| EventIndOmicronBD29 | Indicator that the EventTimeOmicronBD29 failure time is <= the right-censoring time | 1=Omicron endpoint  0=right-censored NA if EventTimeOmicronBD29 is <= 0 or not in the stage 2 cohort | Omicron (both adjudicated and not adjudicated) endpoints are only counted starting 7 days post Day 29 visit, because endpoints occurring during the first 6 days may have already been infected with Omicron before endpoint occurrence. |
| EventTimeOmicronBD29 | Minimum of the time from BD29 visit (antibody marker measurement) until the Omicron endpoint or right-censoring | Integer in days <= 0 if the event occurred before or at BD29 NA if missed the BD29 visit or not in the stage 2 cohort | The BD29 visit is the time origin for studying Day 29 antibody markers as CoRs and as controlled risk CoPs |
| EventIndPrimaryOmicronBD1 | Indicator that the EventTimePrimaryOmicronBD1 failure time is <= the right-censoring time | 1=Adjudicated Omicron endpoint  0=right-censored  NA if not in the stage 2 cohort |  |
| EventTimePrimaryOmicronBD1 | Minimum of the time from BD1 until the adjudicated Omicron endpoint or right-censoring | Integer in days  NA if not in the stage 2 cohort |  |
| EventIndPrimaryOmicronBD29 | Indicator that the EventTimePrimaryOmicronBD29 failure time is <= the right-censoring time | 1=Adjudicated Omicron endpoint  0=right-censored NA if EventTimePrimaryOmicronBD29 is <= 0 or not in the stage 2 cohort |  |
| EventTimePrimaryOmicronBD29 | Minimum of of the time from BD29 visit (antibody marker measurement) until the adjudicated Omicron endpoint or right-censoring | Integer in days <= 0 if the event occurred before or at BD29 NA if missed the BD29 visit or not in the stage 2 cohort |  |
| lgroup | Indicator if the infection is Omicron or not | Omicron = lineage is Omicron  Blank if no endpoint or the variant type is missing  NA if not in the stage 2 cohort |  |
| lineage | Lineage of endpoint | BA.1; BA.1.1; BA.2, etc  Blank if no endpoint or the lineage is missing  NA if not in the stage 2 cohort |  |
| **Antibody marker data** |  |  |  |
| BD1pseudoneutid50 | BD1 visit value of the PPD pseudo-neutralizing antibody readout against the ancestral strain, reported as log10 scale. | Numeric value in the log10 scale  NA iff the participant is not sampled or missed the value | This is the raw value not calibrated to the WHO international unit |
| BD1pseudoneutid50_scaled | Scaled BD1 visit value of the PPD pseudo-neutralizing antibody readout against the ancestral strain, reported as log10 scale. | Numeric value in the log10 scale  NA iff the participant is not sampled or missed the value | This is calibrated to the WHO international unit. The scaling is done by multiplying the PPD level (before taking log) by 0.242/1.04. |
| BD29pseudoneutid50 | BD29 visit value of the PPD pseudo-neutralizing antibody readout against the ancestral strain, reported as log10 scale. | Numeric value in the log10 scale  NA iff the participant is not sampled or missed the value | This is the raw value not calibrated to the WHO international unit |
| BD29pseudoneutid50_scaled | Scaled BD29 visit value of the PPD pseudo-neutralizing antibody readout against the ancestral strain, reported as log10 scale. | Numeric value in the log10 scale  NA iff the participant is not sampled or missed the value | This is calibrated to the WHO international unit. The scaling is done by multiplying the PPD level (before taking log) by 0.242/1.04. |
| DD1pseudoneutid50 | DD1 visit value of the PPD pseudo-neutralizing antibody readout against the ancestral strain, reported as log10 scale. | Numeric value in the log10 scale  NA iff the participant is not sampled or missed the value | This is the raw value not calibrated to the WHO international unit |
| DD1pseudoneutid50_scaled | Scaled DD1 visit value of the PPD pseudo-neutralizing antibody readout against the ancestral strain, reported as log10 scale. | Numeric value in the log10 scale  NA iff the participant is not sampled or missed the value | This is calibrated to the WHO international unit. The scaling is done by multiplying the PPD level (before taking log) by 0.242/1.04. |
| BD1pseudoneutid50_BA.1 | BD1 visit value of the PPD pseudo-neutralizing antibody readout against the BA.1 strain, reported as log10 scale. | Numeric value in the log10 scale  NA iff the participant is not sampled or missed the value | This is the raw value; there is no calibrated WHO international unit for neuts against BA.1 |
| BD1pseudoneutid50_BA.1_scaled | Scaled BD1 visit value of the PPD pseudo-neutralizing antibody readout against the BA.1 strain, reported as log10 scale. | Numeric value in the log10 scale  NA if the participant is not sampled or missed the value | This is calibrated to the WHO international unit. The scaling is done by multiplying the PPD level (before taking log) by 0.242/1.04. |
| BD29pseudoneutid50_BA.1 | BD29 visit value of the PPD pseudo-neutralizing antibody readout against the BA.1 strain, reported as log10 scale. | Numeric value in the log10 scale  NA iff the participant is not sampled or missed the value | This is the raw value; there is no calibrated WHO international unit for neuts against BA.1 |
| BD29pseudoneutid50_BA.1_scaled | Scaled BD29 visit value of the PPD pseudo-neutralizing antibody readout against the BA.1 strain, reported as log10 scale. | Numeric value in the log10 scale  NA if the participant is not sampled or missed the value | This is calibrated to the WHO international unit. The scaling is done by multiplying the PPD level (before taking log) by 0.242/1.04. |
| DD1pseudoneutid50_BA.1 | DD1 visit value of the PPD pseudo-neutralizing antibody readout against the BA.1 strain, reported as log10 scale. | Numeric value in the log10 scale  NA iff the participant is not sampled or missed the value | This is the raw value; there is no calibrated WHO international unit for neuts against BA.1 |
| DD1pseudoneutid50_BA.1_scaled | Scaled DD1 visit value of the PPD pseudo-neutralizing antibody readout against the BA.1 strain, reported as log10 scale. | Numeric value in the log10 scale  NA if the participant is not sampled or missed the value | This is calibrated to the WHO international unit. The scaling is done by multiplying the PPD level (before taking log) by 0.242/1.04. |
| BD1bindRBD | BD1 value of log10 IgG binding antibody concentration to RBD | Numeric value in the log10 scale  NA iff the participant is not sampled or missed the value |  |
| BD29bindRBD | BD29 value of the same marker as BD1bindRBD | Numeric value in the log10 scale  NA iff the participant is not sampled or missed the value |  |
| DD1bindRBD | DD1 value of the same marker as BD1bindRBD | Numeric value in the log10 scale  NA iff the participant is not sampled or missed the value |  |
| BD1bindSpike | BD1 value of log10 IgG binding antibody concentration to Spike protein of the ancestral strain | Numeric value in the log10 scale  NA iff the participant is not sampled or missed the value |  |
| BD29bindSpike | BD29 value of the same marker as BD1bindSpike | Numeric value in the log10 scale  NA iff the participant is not sampled or missed the value |  |
| DD1bindSpike | DD1 value of the same marker as BD1bindSpike | Numeric value in the log10 scale  NA iff the participant is not sampled or missed the value |  |
| BD1bindSpike_Delta | BD1 value of log10 IgG binding antibody concentration to Spike protein of the Delta variant | Numeric value in the log10 scale  NA iff the participant is not sampled or missed the value |  |
| BD29bindSpike_Delta | BD29 value of the same marker as BD1bindSpike_Delta | Numeric value in the log10 scale  NA iff the participant is not sampled or missed the value |  |
| DD1bindSpike_Delta | DD1 value of the same marker as BD1bindSpike_Delta | Numeric value in the log10 scale  NA iff the participant is not sampled or missed the value |  |
| BD1bindSpike_Alpha | BD1 value of log10 IgG binding antibody concentration to Spike protein of the Alpha variant | Numeric value in the log10 scale  NA iff the participant is not sampled or missed the value |  |
| BD29bindSpike_Alpha | BD29 value of the same marker as BD1bindSpike_Alpha | Numeric value in the log10 scale  NA iff the participant is not sampled or missed the value |  |
| DD1bindSpike_Alpha | DD1 value of the same marker as BD1bindSpike_Alpha | Numeric value in the log10 scale  NA iff the participant is not sampled or missed the value |  |
| BD1bindSpike_Beta | BD1 value of log10 IgG binding antibody concentration to Spike protein of the Beta variant | Numeric value in the log10 scale  NA iff the participant is not sampled or missed the value |  |
| BD29bindSpike_Beta | BD29 value of the same marker as BD1bindSpike_Beta | Numeric value in the log10 scale  NA iff the participant is not sampled or missed the value |  |
| DD1bindSpike_Beta | DD1 value of the same marker as BD1bindSpike_Beta | Numeric value in the log10 scale  NA iff the participant is not sampled or missed the value |  |
| BD1bindSpike_BA.1 | BD1 value of log10 IgG binding antibody concentration to Spike protein of the BA.1 variant | Numeric value in the log10 scale  NA iff the participant is not sampled or missed the value |  |
| BD29bindSpike_BA.1 | BD29 value of the same marker as BD1bindSpike_BA.1 | Numeric value in the log10 scale  NA iff the participant is not sampled or missed the value |  |
| DD1bindSpike_BA.1 | DD1 value of the same marker as BD1bindSpike_BA.1 | Numeric value in the log10 scale  NA iff the participant is not sampled or missed the value |  |
| BD1bindSpike_Gamma | BD1 value of log10 IgG binding antibody concentration to Spike protein of the Gamma variant | Numeric value in the log10 scale  NA iff the participant is not sampled or missed the value |  |
| BD29bindSpike_Gamma | BD29 value of the same marker as BD1bindSpike_Gamma | Numeric value in the log10 scale  NA iff the participant is not sampled or missed the value |  |
| DD1bindSpike_Gamma | DD1 value of the same marker as BD1bindSpike_Gamma | Numeric value in the log10 scale  NA iff the participant is not sampled or missed the value |  |
|  |  |  |  |
|  |  |  |  |
|  |  |  |  |
|  |  |  |  |
|  |  |  |  |
|  |  |  |  |
|  |  |  |  |
|  |  |  |  |
|  |  |  |  |
|  |  |  |  |
|  |  |  |  |
| Derived variables |  |  |  |
| risk_score | Baseline risk score for COVID-19 | Numeric value in [0,1] | Built by machine learning |
| naive | BD1 naïve status of the participant | 1 = BD1 naïve  0 = BD1 non-naive | Naïve = 1 - nnaive |
| ph1.BD29 | Indicator if the participant is in the booster stage analysis | TRUE = included in the booster stage analysis  FALSE= not included |  |
| ph2.BD29 | Indicator if the participant is in the booster stage case-control correlates cohort and have BD29 marker data | TRUE = included in the case-control correlates cohort  FALSE= not included |  |
| ph2.DD1 | Indicator if the participant is in the booster stage case-control correlates cohort and have DD1 marker data | TRUE = included in the case-control correlates cohort and have DD1 data  FALSE= not included |  |
| sampling_bucket | Coarsened sampling stratum indicator | 0-31 |  |
| sampling_bucket_formergingstrata | Further coarsened sampling stratum indicator | 0-7 |  |
| wt.BD29 | Inverse probability of sampling weight for participants with BD29 marker data | Numeric value;  NA if the participant is not in the booster phase analysis or not sampled |  |
| WstratumDD1 | Stratification variable defining the case-control sampling design | Counting numbers denoting the strata for case-control sampling |  |
| wt.DD1 | Inverse probability of sampling weight for participants with DD1 marker data | Numeric value;  NA if the participant is not in the booster phase analysis or not sampled |  |
| DeltaBD29overBD1bindSpike | Fold-rise from BD1 to BD20 of the same marker as BD1bindSpike | Numeric value |  |
| DeltaBD29overBD1bindSpike_BA.1 | Fold-rise from BD1 to BD20 of the same marker as BD1bindSpike_BA.1 | Numeric value |  |
| DeltaBD29overBD1bindSpike_Gamma | Fold-rise from BD1 to BD20 of the same marker as BD1bindSpike_Gamma | Numeric value |  |
| DeltaBD29overBD1bindSpike_Alpha | Fold-rise from BD1 to BD20 of the same marker as BD1bindSpike_Alpha | Numeric value |  |
| DeltaBD29overBD1bindSpike_Beta | Fold-rise from BD1 to BD20 of the same marker as BD1bindSpike_Beta | Numeric value |  |
| DeltaBD29overBD1bindSpike_Delta | Fold-rise from BD1 to BD20 of the same marker as BD1bindSpike_Delta | Numeric value |  |
| DeltaBD29overBD1bindRBD | Fold-rise from BD1 to BD20 of the same marker as BD1bindRBD | Numeric value |  |
| DeltaBD29overBD1pseudoneutid50 | Fold-rise from BD1 to BD20 of the same marker as BD1pseudoneutid50 | Numeric value |  |
| DeltaBD29overBD1pseudoneutid50_BA.1 | Fold-rise from BD1 to BD20 of the same marker as BD1pseudoneutid50_BA.1 | Numeric value |  |
| DeltaBD29overBD1pseudoneutid50_scaled | Fold-rise from BD1 to BD20 of the same marker as BD1pseudoneutid50_scaled | Numeric value |  |
| DeltaBD29overBD1pseudoneutid50_BA.1_scaled | Fold-rise from BD1 to BD20 of the same marker as BD1pseudoneutid50_BA.1_scaled | Numeric value |  |
| **Additional derived variables for exposure-proximal analyses** | | | |
| Exposure_proximal_group | Variable indicating when each participant entered the risk set, applies to boosted participants only. | 1 = boosted after 12/01/2021  2 = fully boosted prior to 12/01/2021 (i.e., BD29 + 6 days <= 12/01/2021)  3 = In blackout period on 12/01/2021 |  |
| tstart.1 | Calendar date of entry into the risk set | 12/01/2021 if Exposure_proximal_group = 1 or 2  Date of BD29 + 6 days if Exposure_proximal_group = 3 |  |
| tstop.1 | Calendar date of DD1, end of exposure proximal analysis period, or boost | The earlier of EventTimePrimaryOmicronBD29 and BD1 if Exposure_proximal_group = 1, otherwise EventTimePrimaryOmicronBD29 |  |
| covid.1 | Event indicator for COVID-19 endpoint between tstart.1 and tstop.1 | If Exposure_proximal_group = 1, then 0 EventTimePrimaryOmicronBD29 > BD1 and 1 otherwise  If Exposure_proximal_group = 2 or 3 then equal to EventIndPrimaryOmicronBD29 |  |
| tstart.2 | Calendar date of entry into the risk set following boost if Exposure_proximal_group = 1 and EventTimePrimaryOmicronBD29 > BD29 + 6 days | BD29 + 6 days |  |
| tstop.2 | Calendar date of DD1, end of exposure proximal analysis period | EventTimePrimaryOmicronBD29 |  |
| covid.2 | Event indicator for COVID-19 endpoint between tstart.2 and tstop.2 | EventIndPrimaryOmicronBD29 |  |
| Exposure_proximal_weight | Time-varying weight depending on booster status between tstart.1 – tstop.1, and tstart.2 – tstop.2 | If Exposure_proximal_group = 1, then 1 prior to BD1 and wt.BD29 after tstart.2  If Exposure_proximal_group = 2 or 3, then wt.BD29. |  |
