# Supplementary material for: Omicron COVID-19 immune correlates analysis of a third dose of mRNA-1273 in the COVE trial
Source: Nat Commun. 2024 Sep 11;15:7954. doi: 10.1038/s41467-024-52348-9 (PMC11390939; doi:10.1038/s41467-024-52348-9)
Supplement: Supplementary file 5 — Reporting Summary [file 41467_2024_52348_MOESM5_ESM.pdf]

nature portfolio

Corresponding author(s): Dean Follmann

Last updated by author(s): Aug 27, 2024

Reporting Summary

Nature Portfolio wishes to improve the reproducibility of the work that we publish. This form provides structure for consistency and transparency in reporting. For further information on Nature Portfolio policies, see our Editorial Policies and the Editorial Policy Checklist.

Statistics

For all statistical analyses, confirm that the following items are present in the figure legend, table legend, main text, or Methods section.

n/a	Confirmed	
☐	☒	The exact sample size (*n*) for each experimental group/condition, given as a discrete number and unit of measurement
☐	☒	A statement on whether measurements were taken from distinct samples or whether the same sample was measured repeatedly
☐	☒	The statistical test(s) used AND whether they are one- or two-sided<br>*Only common tests should be described solely by name; describe more complex techniques in the Methods section.*
☐	☒	A description of all covariates tested
☐	☒	A description of any assumptions or corrections, such as tests of normality and adjustment for multiple comparisons
☐	☒	A full description of the statistical parameters including central tendency (e.g. means) or other basic estimates (e.g. regression coefficient) AND variation (e.g. standard deviation) or associated estimates of uncertainty (e.g. confidence intervals)
☐	☒	For null hypothesis testing, the test statistic (e.g. *F*, *t*, *r*) with confidence intervals, effect sizes, degrees of freedom and *P* value noted<br>*Give P values as exact values whenever suitable.*
☒	☐	For Bayesian analysis, information on the choice of priors and Markov chain Monte Carlo settings
☒	☐	For hierarchical and complex designs, identification of the appropriate level for tests and full reporting of outcomes
☐	☒	Estimates of effect sizes (e.g. Cohen's *d*, Pearson's *r*), indicating how they were calculated

Our web collection on statistics for biologists contains articles on many of the points above.

Software and code

Policy information about availability of computer code

Data collection	VAC123 utilized the software associated with the Meso Scale Discovery (MSD) Plate Reader (MPR) Model No. 600 Discovery Workbench software version 4.0.12.1 to generate ECL responses. VAC62 and VAC122 utilized the Gen5 Microplate Reader and Imager Software, Version 3.08.
Data analysis	For VAC62, VAC122 and VAC123, a four-parameter logistic function is used to fit the reference standard using Statistical Analysis Software (SAS) version 9.2, and the sample antibody concentrations are determined by interpolating the sample responses off the fitted reference standard curve.<br><br>All immune correlates analyses were done reproducibly on the basis of publicly available R scripts. A portion of these are hosted on the GitHub collaborative programming platform.35 The rest of these are contained in the Supplementary Software file.<br><br>Reference 35 =<br>35 Zhang, B. et al. Omicron COVID-19 Immune Correlates Analysis of a Third Dose of mRNA-1273 in the COVE Trial. Github. https://doi.org/10.5281/zenodo.13381578. (2024).

For manuscripts utilizing custom algorithms or software that are central to the research but not yet described in published literature, software must be made available to editors and reviewers. We strongly encourage code deposition in a community repository (e.g. GitHub). See the Nature Portfolio guidelines for submitting code & software for further information.

Data

Policy information about availability of data

All manuscripts must include a data availability statement. This statement should provide the following information, where applicable:

- Accession codes, unique identifiers, or web links for publicly available datasets
- A description of any restrictions on data availability
- For clinical datasets or third party data, please ensure that the statement adheres to our policy

Access to patient-level data presented in this article and supporting clinical documents by qualified external researchers who provide methodologically sound scientific proposals will be available upon reasonable request. Such requests can be made to Moderna Inc., 200 Technology Square, Cambridge, MA 02139, email: datasharing@modernatx.com. A materials transfer and/or data access agreement with the sponsor will be required for accessing shared data. All other relevant data are presented in the paper. The protocol is available online at ClinicalTrials.gov: NCT04470427.

Research involving human participants, their data, or biological material

Policy information about studies with human participants or human data. See also policy information about sex, gender (identity/presentation), and sexual orientation and race, ethnicity and racism.

Reporting on sex and gender	Cell lines: The VAC62 and VAC122 microneutralization assays both used a 293T-ACE2 cell line, based on the HEK293 cell line that was originally derived from a female donor. It is unknown whether alternative neutralization assays would provide different results if based on a HEK cell line derived from a male donor.<br>Human research participants: Information on participant sex assigned at birth (male, female) was self-reported. Information on the distribution of participants by sex assigned at birth in the per-protocol boosted cohort and in the per-procotol three-dose correlates cohort is provided in Supplementary Table 2.<br>Participant sex was not a stratum in the stage 2 sampling design (stratified case-control sampling in 3-dose vaccine recipients); see Appendix A.1 in the SAP.<br>It was not a prespecified objective of this work to perform sex-based immune correlates analyses, and post-hoc inferences by sex are not presented because the validity of statistical inferences depends on pre-specification. However, post-hoc descriptive analyses (without inferences) are presented separately for participants assigned male and female sex assigned at birth (Supplementary Fig. 4).
Reporting on race, ethnicity, or other socially relevant groupings	As described in the Statistical Analysis Plan, the sampling design sought balanced numbers of baseline negative per-protocol participants in each of the six demographic strata defined by (Minority, Non-Minority) × (Age ≥ 65, Age < 65 and 'at risk', Age < 65 and Not 'at risk'), within each of the naive and non-naive populations. For the sampling, Minority includes Blacks or African Americans, Hispanics or Latinos, American Indians or Alaska Natives, Native Hawaiians, and other Pacific Islanders. Non-Minority includes all other races with observed race (Asian, Multiracial, White, Other) and observed ethnicity Not Hispanic or Latino. Therefore Unknown and Not reported have missing values for this sampling stratum variable.
Population characteristics	Demographic and clinical information for the per-protocol boosted cohort and the three-dose correlates cohort subset are provided in Supplementary Table 2. Compared to the per-protocol correlates analysis cohort for the blinded-phase COVE correlates analyses (Gilbert et al. Science 2022), the per-protocol boosted cohort was similar in age and sex, but lower in baseline risk (24% vs. 40%).
Recruitment	To enhance the diversity of the trial population in accordance with Food and Drug Administration Draft Guidance, site-selection and enrollment processes were adjusted to increase the number of persons from racial and ethnic minorities in the trial, in addition to the persons at risk for SARS-CoV-2 infection in the local population (from Baden et al. 2021 NEJM).
Ethics oversight	The mRNA-1273-P301 study was conducted in accordance with the International Council for Harmonisation of Technical Requirements for Pharmaceuticals for Human Use, Good Clinical Practice guidelines, and applicable government regulations. The Central Institutional Review Board approved the mRNA-1273-P301 protocol and the consent forms. All participants provided written informed consent before enrollment. Central IRB services for the mRNA-1273-P301 study were provided by Advarra, Inc., 6100 Merriweather Dr., Suite 600, Columbia, MD 21044.

Note that full information on the approval of the study protocol must also be provided in the manuscript.

Field-specific reporting

Please select the one below that is the best fit for your research. If you are not sure, read the appropriate sections before making your selection.

☒ Life sciences ☐ Behavioural & social sciences ☐ Ecological, evolutionary & environmental sciences

For a reference copy of the document with all sections, see nature.com/documents/nr-reporting-summary-flat.pdf

Life sciences study design

All studies must disclose on these points even when the disclosure is negative.

Sample size	Table 7 in the Statistical Analysis Plan (SAP) for the Stage 1 COVE immune correlates analysis (Gilbert et al. 2022 Science, https://www.science.org/doi/suppl/10.1126/science.abm3425/suppl_file/science.abm3425_statistical_analysis_plan.pdf) provided guidelines on the

minimum number of vaccine endpoints needed to conduct Day 57 (~peak antibody) marker correlate of risk (CoR) and correlate of protection (CoP) analyses. For the CoR analyses, these ranged from 25-35, and for the CoP analyses, 50 was the minimum number. These numbers were chosen to ensure there are enough endpoint cases to achieve worthwhile precision in the analyses. The HVTN 505 trial serves as a precedent where 25 evaluable vaccine recipient cases provided enough data to reasonably characterize correlates of risk for a preventive candidate HIV vaccine (Janes et al., 2017; Fong et al., 2018; Neidich et al., 2019; Gilbert et al., 2020). In addition, simulation studies show that correlates analyses at 20 endpoints have notably lower precision.

In Gilbert et al., ~peak antibody correlates were assessed based on 36 vaccine cases with antibody data. While this is lower than the target of 50, it was nevertheless decided to proceed as the scientific question of whether and how neutralizing and binding antibody markers correlate with protection was of timely relevance.

In the present study, antibody markers measured on the day of boost, 28 days later, and fold-rise of these two values were assessed as correlates of Omicron COVID-19 based on 79 Omicron cases in SARS-CoV-2 naïve participants with post-booster antibody data and 32 Omicron cases in SARS-CoV-2 non-naïve participants with post booster antibody data, all in in the per-protocol boosted cohort and pooled across the original-vaccine and crossover-vaccine arms.

The number 79 exceeds the number of 50 needed for CoP analyses defined in the Stage 1 SAP, and the number of 32 cases is similar to that used in the Stage 1 correlates analysis of the Moderna COVE study (36).

Data exclusions	For the VAC62 microneutralization assay, if the antibody concentration (Ab[C]) associated the lowest dilution with a determinate Ab[C] was invalid (due to extravariability or other issues), the sample was excluded from the analyses. Samples invalidated due to extravariability or non-parallelism were not assigned a final Ab[C] and were excluded from the analysis. Moreover, plates that failed system suitability criteria (related to standard curve parameters: max parameter, slope, EC50, min parameter) were excluded from all analyses.<br><br>For the VAC122 microneutralization assay, samples that failed the extravariability criteria and samples with inherent antibody concentrations that exceeded the spike concentration were excluded from subsequent analyses. If the plate did not meet the valid standard curve points criteria, blank well criteria, or control sample criteria, all data associated with the plate were excluded from subsequent analyses.<br><br>For VAC123, if the antibody concentrations (Ab[C]) between replicate wells >1.30 ratio, the result was deemed "extravariable" (EXV) and would be repeated. If the same sample produced two more EXVs deemed valid per the VSDVAC 123 method, the result was set to "Unable to Process" (UTP) and no result was reported. Moreover, plates that failed system suitability criteria (e.g. >2 reference standard curve points invalid, root mean squared error limit exceeded, blank limit exceeded, ≥2 QC samples exceed 2σ limit, etc.) were excluded from all analyses.<br><br>For the correlates analyses, because the lentivirus-based pseudovirus neutralization assay uses an HIV backbone, the presence of anti-retroviral drugs in serum can give a false positive neutralization signal. For this reason, the original immune correlates analysis Gilbert et al. (2022) excluded participants who self-reported being HIV positive, because they would likely be taking anti-retroviral drugs. Consistent with the previous correlates analysis, this SAP also excludes participants who self-reported being HIV positive.
Replication	VAC62: As part of assay validation, the assay's quantifiable range, limit of detection, ruggedness and precision, relative accuracy, dilutional linearity, selectivity, specificity, and quality control specifications were all established. Each assay plate included duplicate wells of each experimental sample dilution; and each assay plate included duplicate wells of each control sample to generate the standard curve. Specifically, an extravariability rule was applied to replicate foci forming units for the reference standard, blank wells, test samples, and quality control samples. A replicate pair of foci forming unit values was designated as extravariable if (1) the range of the untransformed foci forming units exceeded 70, (2) the %CV (Stdev/Mean) of the replicate foci forming units exceeded 50% (Ratio = 2.09), and (3) at least one of the two replicates fell within the foci forming units corresponding to the quantifiable range of the assays (for test samples, quality control samples, and blank wells only).<br><br>VAC122: As part of assay validation, the assay's quantifiable range, limit of detection, ruggedness and precision, relative accuracy, dilutional linearity, selectivity, specificity, and quality control specifications were all established. Each assay plate included duplicate wells of each experimental sample dilution; and each assay plate included duplicate wells of each control sample to generate the standard curve. As a quality control measure for each experimental sample, the replicate readings were assessed for comparability. Specifically, an extravariability rule was applied to replicate foci forming units for the reference standard, blank wells, test samples, and quality control samples. A replicate pair of foci forming unit values was designated as extravariable if (1) the range of the untransformed foci forming units exceeded 70, (2) the % CV (Stdev/Mean) of the replicate foci forming units exceeded 50% (Ratio = 2.09), and (3) at least one of the two replicates fell within the foci forming units corresponding to the quantifiable range of the assays (for test samples, quality control samples, and blank wells only).<br><br>VAC123: One replicate (two wells) are analyzed each time a sample is assayed in VSDVAC 123. An Extravariability (EXV) rule is applied for VAC123. If the antibody concentrations (Ab[C]) between replicate wells is >1.30 ratio, the result is deemed "extravariable" (EXV) and will not generate a result.
Randomization	In the COVE trial, participants were randomly assigned in a 1:1 ratio, through the use of a centralized interactive response technology system, to receive vaccine or placebo. However, after issuance of Emergency Use Authorization to three COVID-19 vaccines by the FDA (between Dec 2020 and Feb 2021), the protocol was amended to offer participants the option of having the group assignments unblinded and, for those who had received placebo, the option to receive the mRNA-1273 vaccine. Thus, participants were not randomized to receive a third dose or not.
Blinding	For assessment of equivalency of neutralizing antibodies between the VAC62 microneutralization assay and the Duke microneutralization assay, the lab was blinded to the historical results until after testing was performed.<br><br>The VAC62, VAC122, and VAC123 assays were performed blinded to assignment to original vaccine vs. crossover vaccine arm status.

Reporting for specific materials, systems and methods

We require information from authors about some types of materials, experimental systems and methods used in many studies. Here, indicate whether each material, system or method listed is relevant to your study. If you are not sure if a list item applies to your research, read the appropriate section before selecting a response.

Materials & experimental systems

n/a	Involved in the study	
☐	☒	Antibodies
☐	☒	Eukaryotic cell lines
☒	☐	Palaeontology and archaeology
☒	☐	Animals and other organisms
☐	☒	Clinical data
☒	☐	Dual use research of concern
☒	☐	Plants

Methods

n/a	Involved in the study	
☒	☐	ChIP-seq
☒	☐	Flow cytometry
☒	☐	MRI-based neuroimaging

Antibodies

Antibodies used: The VSDVAC 123 MSD assay only utilized materials and reagents provided in the commercially available MSD kit, which included the MSD SULFO-TAG™ Anti-human IgG Antibody (Meso Scale Discovery, Cat# D21ADF-3, Lot#s D00V0003, D00V0024, D00V0019) and a human serum sample as QCS4 (BioIVT, Cat# HMSRM-COVIDREC, Lot# HMN374129).

Validation: From MSD's website: "The assays are analytically validated according to the principles outlined in "Fit-for-Purpose Method Development and Validation for Successful Biomarker Measurement" by J. W. Lee, et al. (Pharm Res. 2006;23:312-28). Product validation of every V-PLEX panel includes manufacture of a minimum of three kit lots, using independent raw material lots when available. These are tested in multiple runs, across multiple days, and by multiple analysts. Parameters such as dynamic range, sensitivity, precision, specificity, recovery, linearity, and accuracy are optimized, and data from these lots are used to define specifications."
(https://www.mesoscale.com/en/products_and_services/assay_kits/v-plex/v-plex_quality)

Eukaryotic cell lines

Policy information about cell lines and Sex and Gender in Research

Cell line source(s): Both the VAC62 and VAC122 microneutralization assays used 293T-ACE2 cells. The HEK293 cells was isolated from a human embryonic kidney (HEK) with "293" referring to the experiment used to immortalize the cell line. Two derivatives of HEK293 cells are maintained for laboratory use: HEK 293T (also referred to as 293T) and 293T-ACE2. The 293T cell line is a popular derivative as the cell line contains the SV40-T antigen allowing the cells to be highly transfectectable. 293T-ACE2 cells are monoclonal cell line derived from the 293T transfected with angiotensin I converting enzyme 2 (ACE2) linked with a puromycin resistance gene. The 293T-ACE2 cells are used as a primary effector cell for the SARS-CoV-2 reporter virus particles. 293T-ACE2 cells were sourced from Integral Molecular.

Authentication: This information is captured in the Certificate of Analysis from vendor in which cells were sourced.

Mycoplasma contamination: Yes, cell vials from the working cell bank created at PPD were sent to a 3rd party vendor for mycoplasma testing; the results were negative.

Commonly misidentified lines (See ICLAC register): NA

Clinical data

Policy information about clinical studies

All manuscripts should comply with the ICMJE guidelines for publication of clinical research and a completed CONSORT checklist must be included with all submissions.

Clinical trial registration	COVE trial ClinicalTrials.gov number, NCT04470427
Study protocol	The full COVE trial protocol can be accessed with the publication of the efficacy results at completion of the blinded phase: El Sahly, Hana M., et al. "Efficacy of the mRNA-1273 SARS-CoV-2 vaccine at completion of blinded phase." New England Journal of Medicine 385.19 (2021): 1774-1785.<br>Protocol can be accessed at: https://www.nejm.org/doi/suppl/10.1056/NEJMoa2113017/suppl_file/nejmoa2113017_protocol.pdf
Data collection	Participants were recruited from July 27 to October 23, 2020 at 99 sites in the United States. The present study includes follow-up through April 5, 2022.
Outcomes	In the COVE trial, as described in El Sahly et al. NEJM 2021, "For the primary end point, mRNA-1273 vaccine efficacy in preventing a first occurrence of Covid-19 with onset at least 14 days after the second injection, Covid-19 cases were defined by at least two systemic symptoms (temperature ≥38°C, chills, myalgia, headache, sore throat, or new olfactory or taste disorders), or at least one respiratory sign or symptom (cough, shortness of breath, or clinical or radiologic evidence of pneumonia), and were confirmed by positive SARS-CoV-2 reverse-transcriptase–polymerase-chain-reaction (RT-PCR) assay of nasopharyngeal swab, nasal, or saliva samples. Participants were monitored daily for at least 14 days after diagnosis or until symptoms resolved. " For the Omicron COVID-19 endpoints in the present analysis, as we state in "Omicron COVID-19 Endpoints" in Methods: "COVID-19 cases in COVE were sequenced and we prioritized sampling cases with BA.1 lineage based on sequencing. Of the 79 naïve cases, 41 were identified as BA.1 by sequencing, 26 were identified as BA.1.1 by sequencing, and 12 were inferred to be BA.1 based on COVID-19 occurring after January 15, 2022. Of the 32 non-naïve cases, 16 were identified as BA.1 by sequencing, 3 were identified as BA.1.1 by sequencing, and 13 were inferred to be BA.1 based on COVID-19 occurring after January 15, 2022."

Plants

Seed stocks	NA
Novel plant genotypes	NA
Authentication	NA
